# Movement Intensity in the Minutes Before a Fall: A Lag-Controlled, Activity-Matched Time-Stratified Case-Crossover Analysis of Home Monitoring in Multiple Sclerosis

**DOI:** 10.3390/jcm15218323

**Published:** 2026-10-26

**Authors:** Irina Naskinova, Mikhail Kolev, Meglena Lazarova, Hristo Kalinov, Velin Klisurov

**Affiliations:** 1 Department of Mathematics, University of Architecture, Civil Engineering and Geodesy (UACEG), 1 Hristo Smirnenski Blvd., 1164 Sofia, Bulgaria; inaskinova_fte@uacg.bg (I.N.); mikhail.kolev@uwm.edu.pl (M.K.); 2 Department of Applied Computer Science and Mathematical Modelling, Faculty of Mathematics and Computer Science, University of Warmia and Mazury in Olsztyn, Słoneczna 54, 10-710 Olsztyn, Poland; 3 Department of Mathematical Modelling and Numerical Methods, Faculty of Applied Mathematics and Informatics, Technical University of Sofia, 8 “Kliment Ohridski” Blvd., 1000 Sofia, Bulgaria; 4 Faculty of Mathematics and Informatics, Sofia University “St. Kliment Ohridski”, 5 James Bourchier Blvd., 1164 Sofia, Bulgaria; hkalinov@uni-sofia.bg (H.K.); klisurovi4@gmail.com (V.K.)

**Keywords:** conditional logistic regression, wearable sensors, inertial measurement, accelerometry, within-person design, referent selection, ambulation, signal processing

## Abstract

**Background:** Fall-risk research in multiple sclerosis usually asks who falls and does not address what changes in the minutes before a fall. **Methods:** We analyzed continuous home inertial monitoring of 26 people with multiple sclerosis using a time-stratified case-crossover design. Each fall was compared with referent windows from the same participant on other days at the same clock time. **Results:** After one participant whose accelerometer was not on a 1 g scale was excluded, 48 falls and 285 referents remained in 12 informative strata. Per standard deviation, gravity-removed dynamic acceleration had an odds ratio of 3.05 (95% CI 1.66–5.63). The estimate persisted when the window ended 10, 30, or 60 s before the annotated fall, although these designs overlap the primary one and are not independent tests. Referents from pre-fall days only gave 3.46 (1.67–7.16). When activity was held comparable, the estimate fell to 2.11 (1.17–3.79) within ambulatory windows and to 1.38 (0.86–2.22, *p* = 0.183) against referents from walking periods. **Conclusions:** These results suggest that much of the association reflects the fact that people fall while they are moving. No released fall lies in unrecorded time, which indicates that the public dataset retains sensor-captured falls.

## 1. Introduction

Multiple sclerosis (MS) carries a substantial and rising global burden [1], and falls are among its most consequential everyday complications. Falls in MS are frequent, and their consequences extend beyond injury. Fear of falling and the activity restriction that follows it contribute to deconditioning and reduced participation [2,3]. Identifying who is at risk has therefore been a sustained research priority. A substantial literature relates clinical, neuropsychological, and balance measures to prospective fall counts [4,5], increasingly with machine-learning models [6] and wearable sensing [7,8].

Almost all of this work shares a design. Participants are characterized once, by clinical scales, laboratory gait assessment, or a summary of sensor-derived mobility, and that characterization is related to how often they subsequently fall. The question is *who falls*, and the unit of analysis is the person. Between-person comparisons are confounded by everything that differs between people: disease phenotype and duration, medication, cognition, footwear, home layout, comorbidity, and how much a person walks at all. Some of these can be measured and adjusted for, but most cannot. A between-person model also answers a different question from the one an intervention would act on. Knowing that a person has an elevated annual fall risk does not indicate *when* that person is at risk, and a monitoring system that could warn or intervene requires an answer to this latter question.

The complementary question is within-person and temporal: what distinguishes the minutes immediately before a fall from ordinary minutes in the same person’s life. Continuous home monitoring makes this question answerable, because it records the ordinary minutes as well as the exceptional ones.

This question has been addressed before, in the recordings analyzed here. Kushner and colleagues [7], from the group that collected them, reported higher movement complexity and longer traveled path length in the 10 min before falls than in other periods (entropy 1.15 ± 0.47 against 0.96 ± 0.24, *p* = 0.020; path length 151.3 ± 156.1 m against 95.0 ± 157.2 m, *p* = 0.003). The direction of the present finding therefore agrees with an established result. The same study reports an observation that governs how any such result must be read. Of the falls it recorded, 52% happened while the participant was walking, although participants walked only about 1.5% of the time they were at home. On those figures, movement before a fall largely reflects the fact that falls happen to people who are in motion.

Three gaps remain, and they define what this analysis adds. First, the existing comparison is not matched within person and clock time, so it does not separate the pre-fall interval from ordinary daily variation in a person’s own activity. Second, a window that ends at an annotated fall time cannot distinguish an antecedent state, that is, a condition that precedes the fall, from the beginning of the event itself, and no published analysis of these recordings moves the window to test this. Third, and most important given the 52% figure, no analysis holds activity comparable between the compared periods. The reported contrasts therefore cannot show whether anything beyond being in motion distinguishes the pre-fall interval.

The design that addresses the first gap is the time-stratified case-crossover design [9]. Each case, here each fall, serves as its own control and contributes matched windows from the same person on other days at the same clock time. Case and referent windows come from the same individual, so every stable between-person characteristic is removed by design rather than by adjustment, including characteristics that were never measured. Referent selection in this design has a well-developed literature on the biases that poor choices introduce [10,11], and the time-stratified variant used here was developed to avoid them. The data also favor this design. Fall counts are highly concentrated, with 3 of the 12 participants with falls contributing 70.8% of all analyzed events, so any between-person model on this cohort is effectively a model of three people.

This study makes three contributions, one for each gap. It applies a time-stratified case-crossover design to these recordings, so that every comparison is within participant and within clock time. It reports a lag analysis that moves the window away from the annotated fall time, which a single-window design cannot do. It also reports activity-matched contrasts, including referents drawn from the participants’ own walking periods, which test whether the association is more than motion. Finally, it shows how two signal-processing decisions, the handling of the sampling time base and the definition of gravity removal affect which features reach significance.

**Primary objective.** Estimate which inertial features of the minutes preceding a fall distinguish that interval from matched referent intervals in the same participant at the same clock time.

**Secondary objective.** Establish how much of any such estimate survives displacing the window from the annotated fall time, restricting the referents to days that precede the fall, and holding the participant’s activity comparable across the two arms.

## 2. Materials and Methods

### 2.1. Data Source, Provenance and Label Ascertainment

The data come from a continuous home-monitoring study of people with MS at Oregon Health & Science University. Recordings were made with the MotioWear system (MotioSens, Inc., Portland, OR, USA), which combines a body-worn inertial measurement unit (IMU) sampled at 50 Hz with ambient beacon-based time-of-flight (ToF) position sensing sampled at 4 Hz. The present authors did not operate any device, and this is a secondary analysis of the released recordings. The recordings were released publicly with the study that developed automated fall detection from them [12], which describes how the recordings were collected and how falls were ascertained.

**Parent study.** The monitoring was not a stand-alone observational study. It was a sub-study nested in the Free From Falls randomized controlled trial of a group education and exercise program for reducing falls in people with MS [13], and the monitored sample was recruited from the trial’s participants. The sub-study enrolled 34 individuals and tracked each for eight weeks [7]. Everyone whose recordings are analyzed here was therefore enrolled in a fall-prevention trial while being monitored and may have been receiving the intervention. This affects generalizability and is discussed in Section 4.4.

**Provenance and label independence.** Fall events are supplied by the depositing investigators as a ground-truth file of fall timestamps and participant identifiers. The file was established under the original study’s protocol and was not derived here. The original study used the same recordings to develop a fall detector, so the labels were ascertained by investigators with access to the sensor stream, and the public release does not show whether ascertainment was blind to the inertial signal. The labels are not an output of any model fitted here, no feature used in this analysis was used to define an event, and the case windows exclude the impact. The remaining risk is that timestamps were refined against the signal, which would tie the annotation more closely to the sensor trace than a clinically ascertained fall time would be. The lag analysis of Section 2.7 addresses this risk directly.

**Which falls the public release contains.** The release is a subset of the monitoring program, and the subsetting is not documented. The archive holds recordings for 26 participants and a ground-truth file with 54 fall records in 12 of them, whereas the parent analysis of the same program reports 105 falls in 17 of the 34 enrolled participants [7]. Two components of the difference can be seen in the release. Four participant identifiers within the range documented in the dataset readme, va_27, va_29, va_31, and va_32, are absent from the archive. The release does not state whether these participants account for the missing falls, so this count cannot explain the difference between 17 and 12. In addition, the retained falls are not a random sample of monitored time. Of the case windows, 98.0% meet the coverage gate of Section 2.5, against 72.0% of the 882 clock-matched referent candidates from the same participants on other days (binomial *p* = 2.0 × 10^−6^). No case window contains zero inertial samples, whereas 23.1% of referent candidates do. The released falls are therefore falls that occurred while the sensor was recording. Wearing and recording the device are linked to being up and active, so this selection probably enriches the release for falls during movement. This limits every estimate reported here and is discussed in Section 4.4.

The cohort was enumerated from the archive index rather than from the dataset documentation, and the two disagree. Table 1 gives both, and Table 2 describes each participant.

Fall counts are highly concentrated. Three participants contribute 13, 12, and 9 of the 48 analyzed events, or 70.8% of the total, while 13 of the 25 analyzed participants contribute none. This concentration shapes every design decision below.

### 2.2. Participant Characteristics

The public release carries inertial and position signals and a fall-timestamp file, and nothing else. It contains no age, sex, disease duration, phenotype, Expanded Disability Status Scale score, medication, or assistive-device information, and none of these can be recovered from the recordings. A characteristics table for this cohort can therefore describe only what the monitoring itself observed, and Table 2 is built on that basis. Clinical characteristics of the parent trial population are reported by the studies that collected the data [7,13]. They describe the 34 enrolled participants rather than the 25 analyzed here and are therefore not reproduced.

### 2.3. Case-Crossover Design and Window Construction

Each fall anchors one case window, the 300 s ending at the fall timestamp in the primary specification. The window therefore covers the interval leading up to the fall but not the impact itself.

Referent windows are drawn from the same participant on other monitored days at the same clock time, which is the time-stratified referent scheme [9,10,11]. Matching on clock time controls for daily rhythms in activity, and matching within participant controls for everything stable about the person, the referent windows and the case windows form the two arms of the comparison.

**How the referent days were chosen.** The rule is mechanical and is stated in full, because referent selection is where this design is most easily compromised. For a given fall, the eligible days are the participant’s monitored days, excluding the calendar days touched by the widest window anchored at that fall and the days touched by any other fall of the same participant. Each eligible day supplies one candidate referent, obtained by shifting the case window by a whole multiple of 86,400 s, so the referent replicates the case’s clock-time span exactly in the archive’s UTC time base. It does not do so in local clock time. A referent on the other side of a daylight-saving change from its case lies one hour away in local time, which applies to 69 of the 285 primary referents, and Section 3.4 refits the model without them. A candidate is dropped if the shifted window falls within two hours before or 24 h after any fall of that participant. The washout is deliberately asymmetric. Two hours keep a referent clear of another fall’s own lead-in, whereas the aftermath of a fall, including any injury, rest, or medical attention, can be expected to last for the rest of that day and would otherwise pull the referent arm toward the profile of the case arm. The surviving days are shuffled with a fixed seed, up to three times the target count is retained, and the selection step keeps the first six that pass the inertial-coverage gate. Referent days are therefore drawn at random from the eligible set rather than by proximity to the fall, and the six retained for a given case may lie on either side of it.

This leaves one bias open, and it is tested here. The washout clears only the 24 h after a fall, so days from day +2 onward remain eligible. A participant with many falls can therefore contribute referents that follow a fall, and activity after a fall is often reduced by injury or by fear of falling [2,3]. A referent arm with lower activity for that reason would inflate a movement odds ratio without any antecedent state. Section 3.4 tests whether referent activity differs by side of the fall and refits the model on pre-fall referents only.

Two alternative window lengths, 2 and 8 min, and four lagged specifications (Section 2.7) were analyzed as separate complete designs.

### 2.4. Features

Eight IMU features summarize each window: mean acceleration magnitude, gravity-removed dynamic acceleration root mean square (RMS), mean and peak jerk, dominant frequency, spectral entropy, gait-band power fraction, and posture-transition rate. Four ToF features describe movement through the home: mean speed, path length, tortuosity, and room-transition rate. The archive does not contain room labels. The room proxy is a per-participant *k*-means discretization of tracked positions sampled through the analysis windows, and it is not a ground-truth room identity.

**Ambulation marker.** One derived binary variable is used only to hold activity comparable between the arms (Section 2.8). A window is *ambulatory* if its posture-transition rate exceeds zero, that is, if the participant changed posture at least once during the window. A posture transition cannot occur without the person moving. This is why the marker is preferred to a threshold on the gait-band power fraction, which is scale-free, so that a motionless window with slight low-frequency tremor can score highly on it. In the analysis set, the marker flags 95.8% of case windows and 44.6% of referent windows (Table S44).

Two aspects of how the features are computed affect the results, and both are reported.

**Uniform time base.** The archive’s sampling is not perfectly regular. The observed interval varies around the nominal 20 ms, and intervals shorter than nominal are common enough that the coverage fraction exceeds 1 in 148 of the 1267 primary candidate windows, reaching 1.36. If jerk is computed by dividing the first difference of the acceleration magnitude by the observed interval, a single anomalously short interval can determine a feature that is a maximum over roughly 15,000 samples. Every amplitude, jerk, and spectral feature was therefore computed on a uniform 50 Hz grid obtained by linear interpolation, so that no observed interval enters a denominator. Grid points that would be interpolated across a gap longer than 1 s were marked invalid and excluded from all derived features, so that interpolation does not create smooth, low-jerk data. The uniform grid also gives the Welch spectral estimates the evenly spaced input they assume. For comparison, peak jerk was also computed with the observed interval in the denominator, and the model was refitted with it (Section 3.6).

**Gravity removal.** Removing gravity requires working per axis, because the magnitude of the acceleration vector discards the direction in which the device accelerated [14,15]. Each axis was low-pass filtered at 0.3 Hz with a zero-phase fourth-order Butterworth filter to estimate the gravity component. That estimate was subtracted, and the RMS of the norm of the residual vector was taken. Zero-phase filtering matters because a causal filter would shift the estimated gravity in time relative to the signal it is subtracted from, and this analysis concerns the seconds before an event. The RMS of the mean-removed acceleration *magnitude* is a different measurement: it is algebraically the standard deviation of that magnitude. It is retained as an alternative feature, and the model is refitted with it as a sensitivity analysis (Section 3.6).

### 2.5. Exclusions

Three screens were applied before the model was fitted: one to participants, one to windows, and one to predictors.

A stationary tri-axial accelerometer reads 1 g. One participant’s record has a median window-mean magnitude of 4.06 g, a scale anomaly across the whole record rather than a scatter of bad windows. Participants whose median window-mean magnitude falls outside 0.8 to 1.3 g were excluded whole, and the observed value is reported (Table S4). This excludes va_4 and no one else, which restores the cohort used by the source study (Section 2.1).

**Coverage, applied symmetrically.** A window was accepted if its IMU coverage fraction was at least 0.9. The gate was applied alike to case windows, referent windows, and the randomly timed windows of participants who never fell. A gate applied to only one arm would make the arms differ in more than the timing of the window, and coverage plausibly depends on wear and movement. The gate removes one event, and its matched set is dropped with it, because a matched set without its case contributes nothing to a conditional likelihood. Among accepted primary windows, the Spearman correlation between coverage and each feature is at most 0.057 in absolute value (all *p* ≥ 0.240), and coverage does not differ between arms (Mann-Whitney *p* = 0.740).

**Predictors.** Two predictors were excluded from the conditional model. Mean acceleration magnitude is nearly constant, because gravity dominates it, and it destabilized the fit. Mean jerk nearly duplicates the dynamic-acceleration term. This screen was post hoc: it was carried out after an implausible odds ratio was observed in a model containing both dynamic-acceleration terms. The full pairwise correlation matrix is given in Table S14. In it, the magnitude fluctuation measure correlates with the acceleration-magnitude standard deviation at *r* = 1.0000, which confirms that the two are the same quantity.

### 2.6. Statistical Analysis

The primary model is conditional logistic regression stratified on participant, using the exact conditional likelihood rather than a partial-likelihood approximation [16]. Each participant forms one stratum. Only strata that contain both a case and a referent, called informative strata, contribute to the conditional likelihood; the others are dropped by the estimator. Odds ratios (ORs) are reported with 95% confidence intervals (CIs) from Wald standard errors.

Features were standardized so that ORs are expressed per standard deviation (SD). The standardization was performed after strata without a case had been removed, so the mean and SD that define “per SD” come from exactly the rows that enter the likelihood. Standardizing over every row passed to the estimator, including windows from fall-free participants whose strata are then discarded, would let 94 rows influence every effect size without contributing to the fit. Both counts are reported for every model, and the primary model was also refitted with the wider standardization population (Section 3.6).

Three checks follow. A leave-one-participant-out analysis refitted the model with each of the 12 case-contributing participants excluded in turn, reporting the shift in log OR and any change in significance. A permutation control shuffled case labels within stratum 500 times to give a null distribution for the model likelihood-ratio statistic, together with a leave-one-feature-out check that no single feature carries the result. A leakage audit checked that the case labels in the feature table agree with the window index.

A gradient-boosted model [17] was fitted with participant-grouped cross-validation as a descriptive check on feature ranking. It is not independent confirmation, because it uses the same recordings, features, and labels, so any timing or feature-construction artifact would affect it equally. Its window set differs from that of the conditional model, and Section 3.7 gives both counts. This difference is what makes a fold without cases possible. Discrimination is summarized by the area under the receiver operating characteristic curve (AUROC). Its CI comes from a participant-level cluster bootstrap in which the entire grouped cross-validation is repeated in every replicate, so that the interval includes model-fitting and fold-assignment variability.

### 2.7. Lag Analysis: Displacing the Window from the Annotation

A window that ends exactly at the annotated fall timestamp cannot distinguish an antecedent state from the beginning of the fall. Window lengths that share a right edge cannot test the distinction either, because they differ only in how far back the left edge reaches. Four additional designs were therefore fitted, with case windows ending 10, 30, 60, and 120 s before the annotated time and the primary 5 min length held fixed. The same lag was applied to the matched referent windows, so each lagged analysis is a complete case-crossover design rather than a shifted case arm compared with an unshifted referent arm.

These designs are nested. A window displaced by 10 s shares 96.7% of its span with the primary window, one displaced by 30 s shares 90.0%, one displaced by 60 s shares 80.0%, and one displaced by 120 s shares 60.0%. The lag estimates are therefore repeated measurements on largely the same data, not independent replications, and differences among them carry no evidential weight. A higher estimate at one lag cannot be read as the association strengthening. The family supports only the weaker statement that the association does not depend on the seconds closest to the annotation. The expectation, stated before the lagged models were fitted, was one-sided. If the association reflects detection of the fall or of its immediate lead-in, it should move toward the null as soon as the right edge is moved off the annotation. A state occupying the minutes before the event should persist while the windows still overlap that interval.

### 2.8. Holding Activity Comparable

Falls occur disproportionately while people are in motion, as the walking figures from the parent study show (Section 1) [7]. A contrast between pre-fall windows and referent windows drawn without regard to activity therefore risks measuring little more than the difference between moving and sitting still. The lag analysis cannot resolve this, because moving the window by a few tens of seconds leaves the participant in the same activity state.

Three specifications hold activity comparable, in increasing severity. All three are reported because they balance bias and interpretability differently.

The first adds the binary ambulation marker of Section 2.4 to the model as a covariate, so that the movement-intensity term is estimated within levels of the marker. The second restricts both arms to ambulatory windows and refits the model, with the posture-transition rate dropped from the predictor set because it defines the restriction. The third replaces the referents themselves. For each case, the eligible referent candidates that pass the coverage gate are ranked by gait-band power fraction, and the six most walking-like are retained, so that each fall is compared with the participant’s own walking periods at the same clock time. The third is the most severe test available in these data, but it has a cost that the second does not. Its referents are selected on two of the model’s own predictors, which leaves those predictors almost no within-stratum contrast and attenuates the movement term through their collinearity. For that reason, the single-predictor fit on the same referent set is reported beside the full model.

A fourth specification applies the activity control and the lag control together. Separately, the lag family asks whether the association reflects detection of the impact, and the activity contrast asks whether it merely reflects motion. Neither answers the other question, because windows displaced by a few tens of seconds leave the participant in the same activity state. The lag designs of Section 2.7 were therefore refitted with both arms restricted to ambulatory windows. As in the second specification, the posture-transition rate was dropped from the predictor set because it defines the restriction, so these estimates come from a five-predictor model and are not directly comparable with the primary model.

### 2.9. Software and Data Integrity

Analyses used Python 3.13.12 with statsmodels 0.14.6 (https://www.statsmodels.org, accessed on 1 August 2026), XGBoost 3.2.0 (https://xgboost.readthedocs.io, accessed on 1 August 2026) [17], NumPy 2.4.2, pandas 2.2.3, SciPy 1.16.3, scikit-learn 1.6.1, and Matplotlib 3.10.8. Random seeds are fixed. The analysis code is provided in Supplementary File S1.

One of the 1279 archive members referenced by the analysis failed its checksum verification and could not be read, and all others were verified (Table S2). The single referent window that depended on it was excluded by the coverage gate.

## 3. Results

### 3.1. Analytic Sample

Feature extraction produced 7525 candidate windows across all 26 archived participants, with IMU coverage of at least 0.9 in 68.7% of rows. One participant was excluded by the calibration audit (Section 2.5). After the symmetric coverage gate and referent matching, the primary analysis comprised 48 case events with 285 matched referent windows, or 5.94 referents per case, from 25 participants. The conditional model was fitted on 333 windows (48 cases and 285 referents) in 12 informative strata. A further 94 windows from the 13 fall-free participants were in the table but contributed no term to the conditional likelihood and were excluded before standardization.

### 3.2. Primary Model

The primary specification compares each case window with the referent windows matched to it in the same participant at the same time of day. Odds ratios for the six retained predictors are given in Table 3.

**Figure 1:**
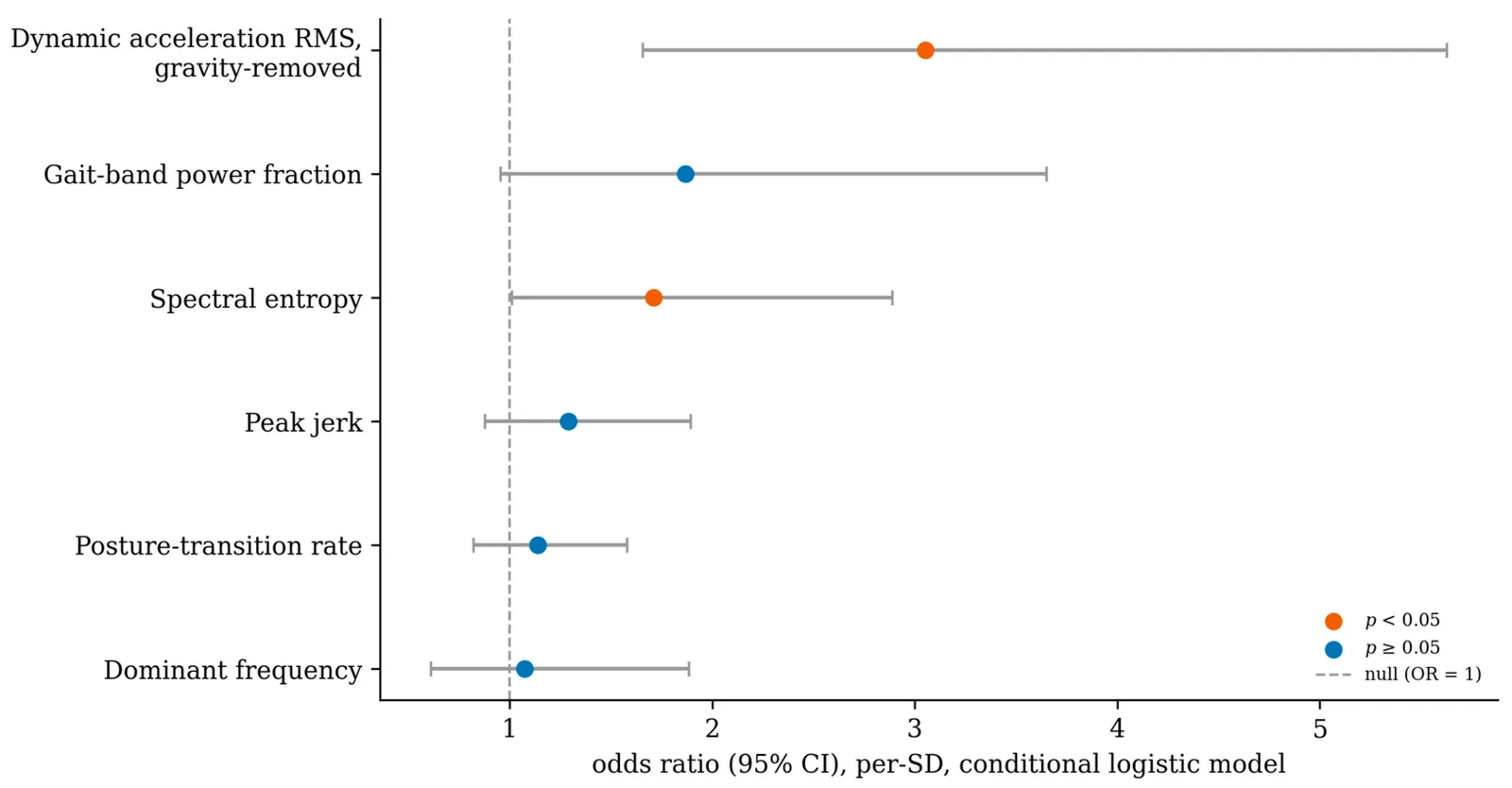
Primary conditional logistic regression model. Five-minute windows ending at the annotated fall time. Odds ratio per standard deviation with 95% confidence interval for each of the six retained predictors. The dashed vertical reference line is labeled null (OR = 1). Predictors reaching *p* < 0.05 are drawn in orange and labeled *p* < 0.05 in the legend, and the others are drawn in blue and labeled *p* ≥ 0.05. Estimates are from the model in Table 3, fitted on 333 windows across 12 informative participant strata.

The five minutes preceding a fall carried greater movement intensity than matched referent intervals in the same participant at the same time of day. Figure 1 shows the same estimates as a forest plot.

Peak jerk did not reach significance. Spectral entropy was nominally significant at *p* = 0.045, but Section 3.5 shows that it is not stable, and with six predictors tested it is not interpreted as a finding.

### 3.3. Displacing the Window from the Annotation

**Figure 2:**
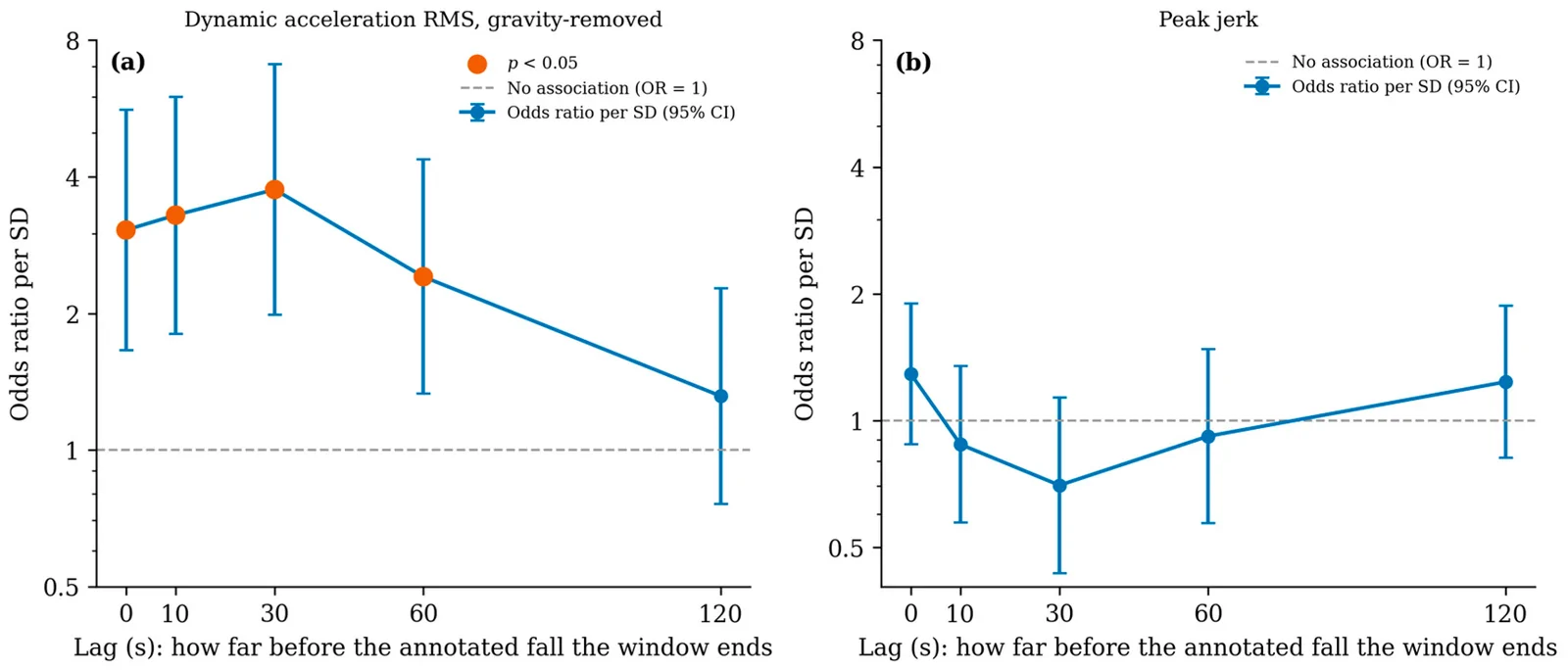
Movement term and peak jerk under displacement of the window. (**a**) Odds ratio per standard deviation, with 95% confidence interval for the gravity-removed dynamic acceleration RMS term, labeled Odds ratio per SD (95% CI) in the legend and plotted against the lag, that is, how far before the annotated fall the window ends. Markers reaching *p* < 0.05 are drawn in orange and labeled *p* < 0.05, and the dashed horizontal reference line is labeled No association (OR = 1). The vertical axis is logarithmic. (**b**) The same plot for peak jerk, which reaches significance at no lag. Each point is a separate complete case-crossover design in which the stated lag was applied to the case and the matched referent windows alike. The designs are nested rather than independent, so the panels show whether each interval excludes 1, not an ordering of the point estimates.

The primary windows end exactly at the annotated fall time, so the estimate above could in principle rest on the event itself. To separate the two, the same design was refitted, with the right edge of every window, case and referent alike, moved back from the annotation. Table 4 and Figure 2 report the five lag specifications.

The association persisted when the window was moved away from the annotation. An estimate produced by capturing the impact, or the first moments of the fall, would move toward the null as soon as the right edge was moved back by a few seconds and would not survive a 30 s displacement. The association persisted to 60 s and was lost only at 120 s, as the window moved out of the pre-fall interval. The lagged windows overlap the primary one (Table 4), so the variation among their point estimates is not interpreted, and the association cannot be said to strengthen at intermediate lags.

This is a limited safeguard against annotation error. The 0 s and 10 s windows differ by less than the plausible error in a fall timestamp, so the analysis cannot show that the primary estimate is free of any contribution from the event itself. It shows that an association confined to the annotated instant would not still be present, at a similar magnitude, half a minute earlier.

Peak jerk behaved differently (Figure 2b). It was not significant at any lag, and its point estimate fell below 1 at 10, 30, and 60 s. These data show no antecedent peak-jerk signal.

### 3.4. Referent Selection and Activity

**Figure 3:**
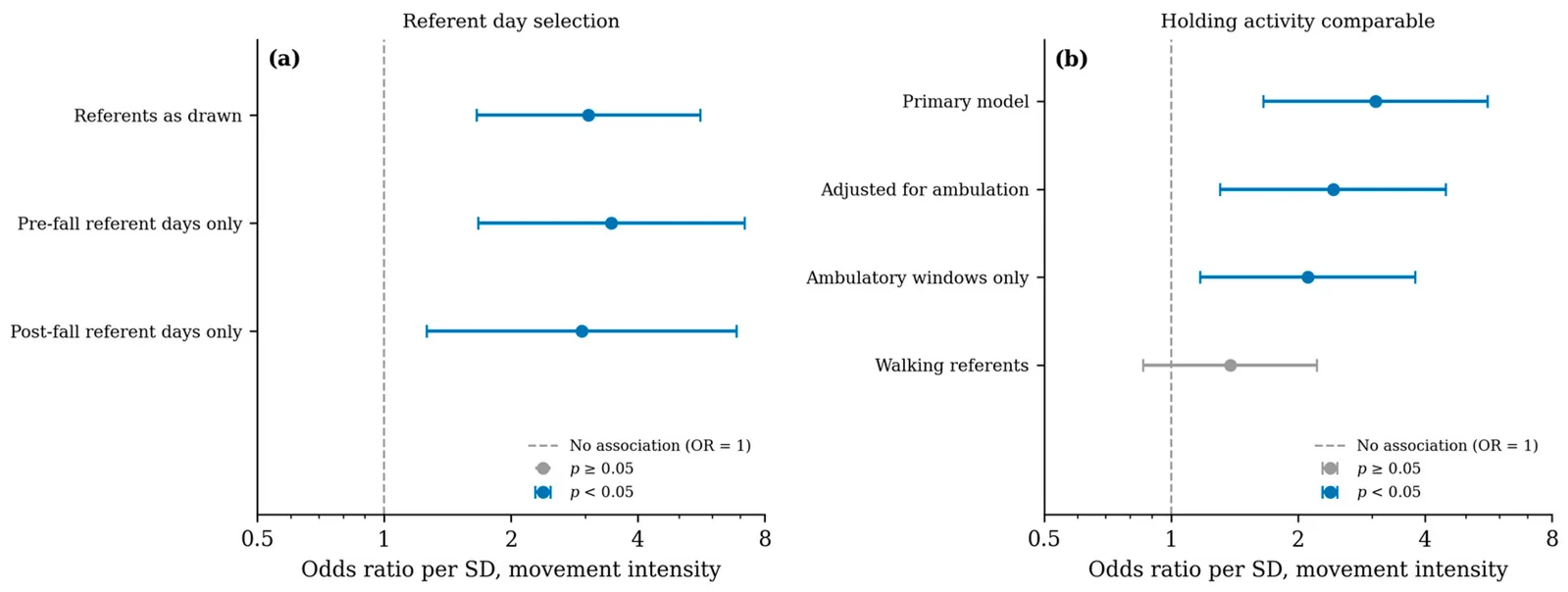
Referent-selection and activity contrasts. The movement association under two explanations that do not require a distinctive pre-fall state. (**a**) Referent day selection: odds ratio per standard deviation with 95% confidence interval for the movement term, with the referents as drawn, with pre-fall referent days only, and with post-fall referent days only. (**b**) Holding activity comparable: the same estimate for the primary model, adjusted for the ambulation marker, restricted to ambulatory windows in both arms and against referents drawn from walking periods. In both panels, estimates reaching *p* < 0.05 are drawn in blue and labeled *p* < 0.05, estimates not reaching it are drawn in gray and labeled *p* ≥ 0.05, the dashed vertical reference line is labeled No association (OR = 1), and the horizontal axis is logarithmic. In panel (**b**), the estimate declines as activity is held more nearly constant, and in the final row it no longer excludes 1.

Two explanations would let the primary estimate arise without any distinctive pre-fall state. Under the first, the referent arm is quieter because much of it is drawn from days after a fall. Under the second, the contrast reflects no more than the difference between moving and sitting still. Both are tested in Table 5 and Figure 3. A third specification, reported at the end of this section and in the last five rows of Table 5, applies the activity control and the displacement control of Section 3.3 together.

**Referent days.** The referents divided almost evenly, 141 falling before their anchoring fall and 144 after it, and three participants contributed no pre-fall referent. Referents on post-fall days were quieter. Within participant, they carried lower gravity-removed dynamic acceleration than referents on pre-fall days (mean within-participant *z* −0.175 against +0.178, Mann–Whitney *p* = 0.003) and lower peak jerk (*p* = 0.004). The gait-band power fraction and the posture-transition rate did not differ (*p* = 0.460 and *p* = 0.290). The premise of this bias therefore holds, but it does not produce the result. Refitting on pre-fall referents alone, which cost four events and three strata, gave an OR of 3.46 (1.67–7.16), slightly larger than the primary estimate rather than smaller. Post-fall referents alone gave 2.94 (1.26–6.85). Here, “pre-fall” is defined against the anchoring fall, so for a frequent faller a pre-fall referent can still follow an earlier fall. Two stricter definitions close that gap. Referents preceding the participant’s first released fall gave 4.61 (1.61–13.23; 31 events in eight strata), and referents at least seven days after any fall gave 3.78 (1.85–7.72). Dropping the 69 referents that lie across a daylight-saving change from their case gave 2.95 (1.51–5.77). Neither referents from the aftermath of a fall nor the time base used to match them explain the estimate.

**Activity.** The activity contrast changes how the primary estimate should be read. The ambulation marker, entered on its own, carried an OR of 5.69 (2.76–11.75). This is larger than the continuous movement term in any specification, so most of what separates a pre-fall window from a referent window is that the participant was up and moving at all. Holding activity comparable attenuated the movement term at every level of severity. Adjusted for the marker in the full model, it fell from 3.05 to 2.42 (1.31–4.49). Restricted to ambulatory windows in both arms, it was 2.11 (1.17–3.79). Against referents drawn from the participants’ own walking periods, the full model gave 1.38 (0.86–2.22, *p* = 0.183), which does not exclude no association. On the same walking referents, the movement term fitted alone retained 1.73 (1.23–2.43). This difference between the two fits is expected, for the reason given in Section 2.8: the walking referents were selected on two of the full model’s predictors.

**The two controls together.** Restricting the lag family to ambulatory windows holds activity comparable and moves the window at the same time, so it shows what survives both controls. The movement term was 2.11 (1.17–3.79) with the window ending at the annotation, 2.37 (1.29–4.37) at a 10 s displacement and 2.51 (1.34–4.70) at 30 s. It was no longer distinguishable from no association at 60 s (1.53, 0.82–2.86) or at 120 s (0.89, 0.48–1.64). The displacement result of Section 3.3 therefore does not depend on the arms differing in activity. Within ambulatory windows, the association was still present half a minute before the annotated time, which detection of the impact cannot produce. The analysis also shows that the remaining association is confined to a shorter interval than in the unrestricted lag family, fading by 60 s rather than by 120 s. The nesting caveat of Section 2.7 applies to these five designs as it does to Table 4.

Taken together, the three contrasts support a narrower reading than the primary model alone. A substantial part of the association between pre-fall windows and movement intensity reflects the fact that falls happen while people are in motion, as the parent study’s walking figures already implied [7]. What survives holding activity comparable is a smaller graded association, of the order of two per SD. It holds in two of the three specifications and cannot be separated from the null in the most stringent one.

### 3.5. Stability and Controls

The primary estimate was then put under three controls: removing participants one at a time, permuting the labels within strata, and auditing the labels themselves.

**Figure 4:**
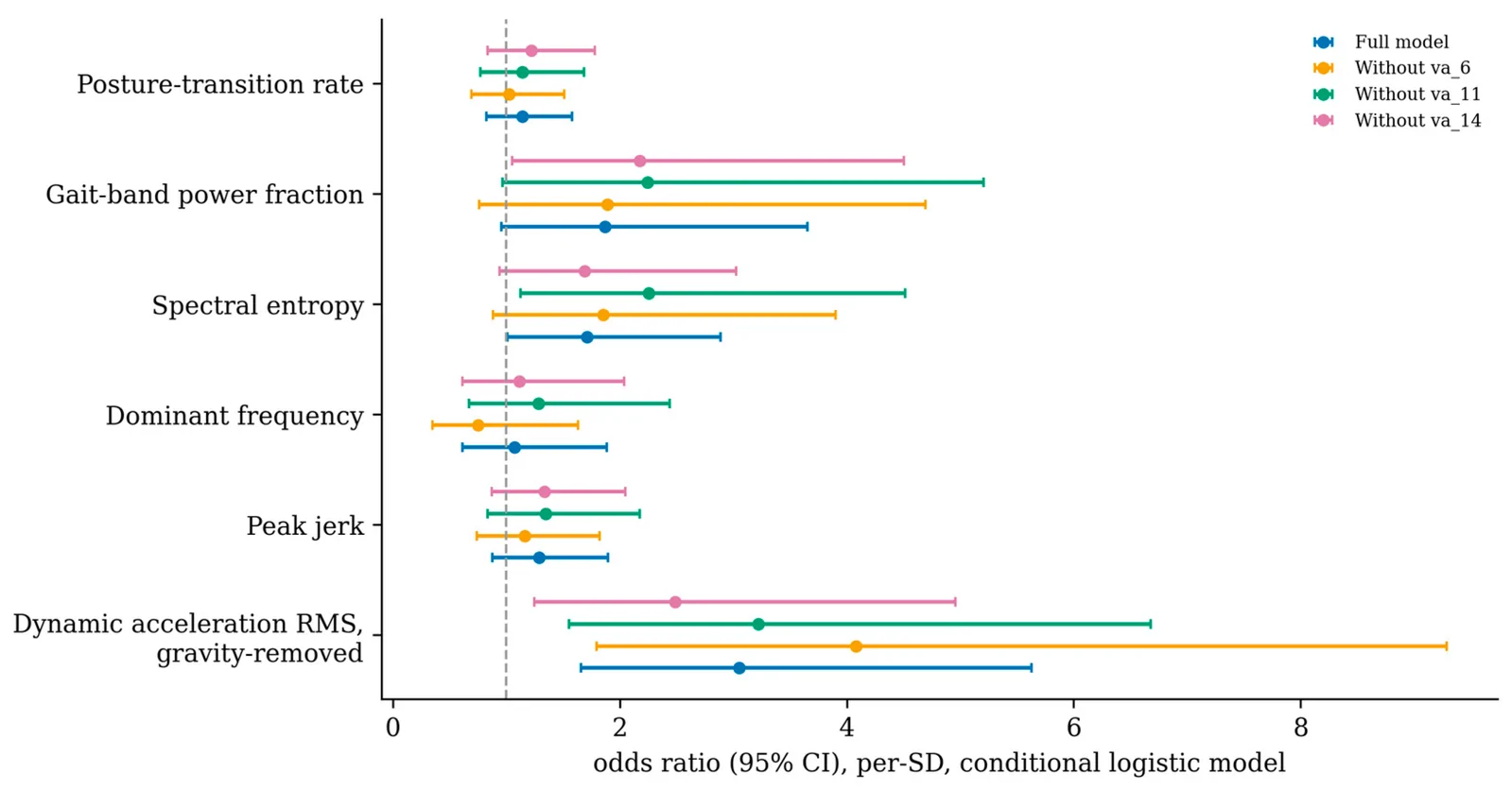
Leave-one-participant-out sensitivity. Odds ratio per standard deviation with 95% confidence interval for each predictor in the full model (labeled Full model) and after removing, in turn, each of the three participants contributing the most fall events (labeled Without va_6, Without va_11, and Without va_14). The dashed vertical line marks OR = 1. The gravity-removed movement term does not cross OR = 1 under any removal.

**Leave-one-participant-out.** Across all 12 case-contributing participants, no single removal changed the significance of the gravity-removed movement term, whose largest shift in log OR was 0.29. Two other predictors were not stable. Spectral entropy changed significance when any of five different participants was removed, including participants contributing as few as one event, and the gait-band power fraction changed when the participant contributing nine events was removed. Neither is treated as a finding. Figure 4 shows the shift for every predictor.

The observed model likelihood-ratio statistic was 67.69, against a within-stratum permutation null with a mean of 6.23 (SD 3.61, maximum 24.25 over 500 permutations), *p* = 0.002. No single feature removal reduced the statistic to the range of the permutation null. Removing the movement term reduced it most, to 53.75.

A leakage audit found no label mismatches between the feature table and the window index across all 7525 rows.

### 3.6. Sensitivity to Processing, Standardization, and Window Length

Five processing choices were varied in turn, each with the rest of the design held fixed, to show which of them the findings depend on.

**The sampling time base.** Across the 333 windows of the analysis set, dividing by the observed sampling interval inflated peak jerk by a median factor of 1.21, by more than twofold in 7.8% of windows and by up to 6.0-fold. With this definition, peak jerk reached significance (OR 1.56, 1.07–2.28, *p* = 0.022), whereas on the uniform time base it did not (Table 3). The movement term remained significant, at 2.60 (1.43–4.74). The time base therefore decides whether peak jerk appears as a finding (Tables S42 and S43).

Refitting the primary model with features standardized over all 427 windows, rather than over the 333 in informative strata, changed the ORs by at most 4% (dynamic acceleration 3.00 against 3.05, peak jerk 1.27 against 1.29). The conclusions do not depend on this choice.

Refitting with the acceleration-magnitude standard deviation in place of the gravity-removed term gave an OR of 2.39 (1.35–4.23, *p* = 0.003) for that term and left peak jerk non-significant (*p* = 0.102). The direction and significance of the movement finding therefore do not depend on which of the two acceleration measures is used, but its magnitude does.

The movement association held at all three window lengths, with an OR of 3.89 (1.98–7.65) at 2 min, 3.05 (1.66–5.63) at 5 min and 1.77 (1.01–3.12) at 8 min. The decline with window length is consistent with a state concentrated in the minutes immediately before the event and diluted as the window widens.

Restricting the analysis to windows with usable ToF coverage, which excludes two participants entirely, left the movement term significant with a much larger and far less precise estimate, an OR of 15.46 (3.65–65.38). No ToF feature reached significance. The complete-case subset is too small for the magnitude to be interpreted.

### 3.7. Secondary Boosted Model

The participant-grouped gradient-boosted model reached an AUROC of 0.797. Its cluster-bootstrap interval was 0.693–0.865, obtained by repeating the entire grouped cross-validation in each of 200 replicates.

Its window set differs from that of the conditional model, which matters for reading the folds. The boosted model was fitted on 427 windows from all 25 analyzed participants, including the 94 windows contributed by the 13 who never fell, whereas the conditional model used the 333 windows in the 12 informative strata. A participant-grouped split over 25 participants, 13 of whom have no case, can therefore leave a fold without any case, and one of the five folds contained none. That fold has no defined AUROC, so the model yields four fold estimates, which range from 0.648 to 0.825. These folds contain between 14 and 211 windows each, so their spread mainly reflects the size of the test sets and is not compared with the interval for the pooled estimate.

**Figure 5:**
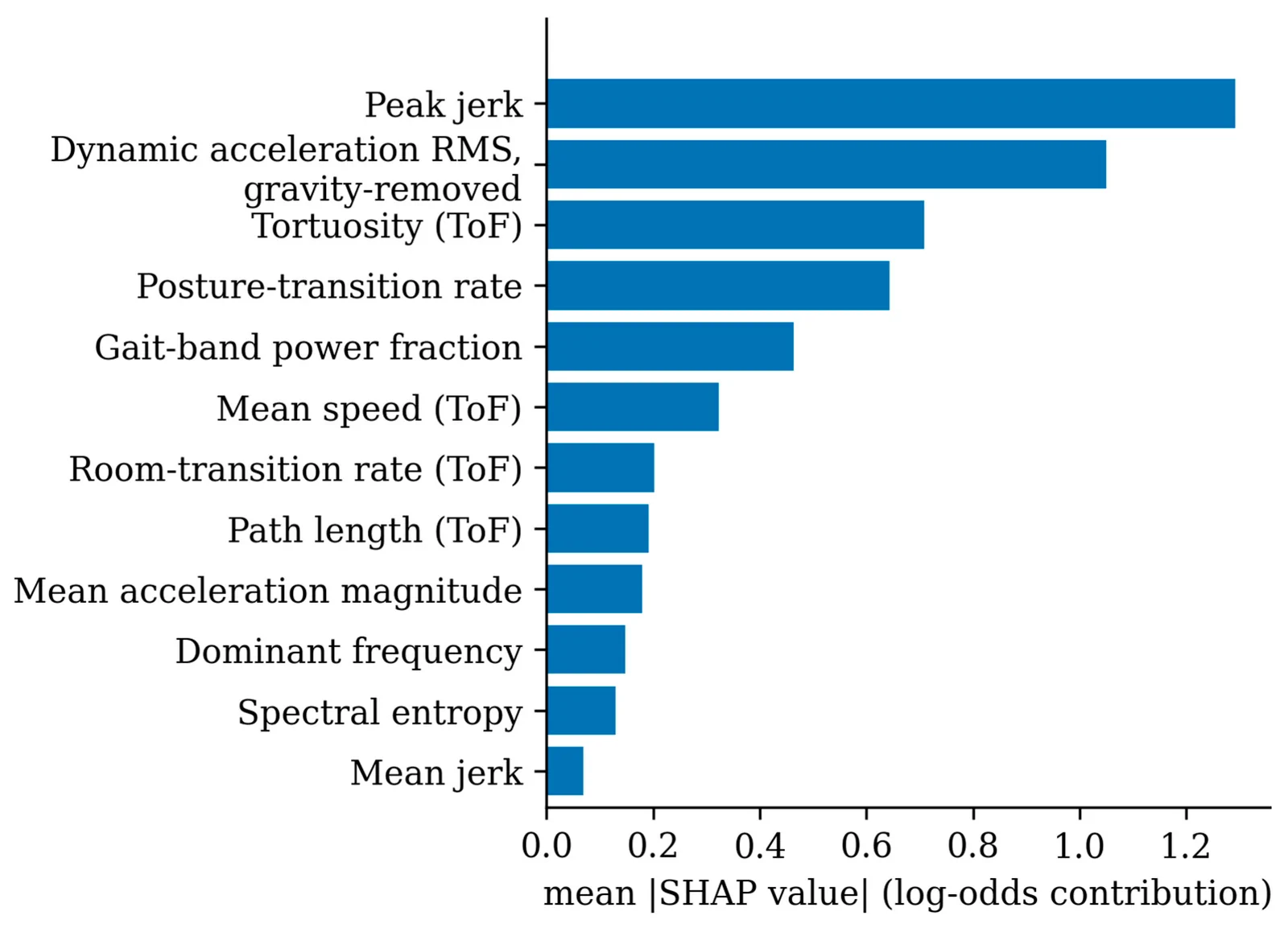
Feature importance in the gradient-boosted model. TreeSHAP mean absolute attribution per feature for the participant-grouped gradient-boosted model, ordered by magnitude. Each bar is the mean |SHAP value| of one feature, and all bars are drawn in a single color.

Its TreeSHAP ranking placed peak jerk first and the gravity-removed movement term second (Figure 5). The two models disagree about peak jerk, which the conditional model does not find significant, and because they share recordings, features, and labels, their agreement on the movement term is not independent evidence (Section 2.6).

## 4. Discussion

### 4.1. The Pre-Fall Interval and the Role of Motion

The pre-fall interval is characterized by elevated movement intensity, and the direction of that result is not new. Kushner and colleagues [7], analyzing the same monitoring program, already reported greater movement complexity and longer traveled path length in the 10 min preceding falls. The present analysis agrees with them. Its contribution concerns three aspects of the interpretation rather than the existence of the association: the association holds within person and within clock time under matched referents, it survives moving the window away from the annotation, and most of it is attributable to the participant being in motion at all.

The last point should govern how the result is read. It comes from a comparison that the published analysis of these recordings did not make. A binary marker of whether the participant moved at all carries a larger OR, 5.69, than the continuous movement-intensity term achieves in any specification. Once activity is held comparable, the intensity term falls to about two per SD, and against referents taken from the participants’ own walking periods the full model no longer distinguishes it from no association. Falls in this cohort happen while people are moving, as the parent study’s figures suggested. Beyond this, there is a weaker graded tendency for the pre-fall minutes to be more vigorous than comparable moving minutes. These data support that tendency in two of three specifications and cannot establish it in the most stringent one.

This conclusion makes no claim about a distinct antecedent state. No mechanism is identified. The design cannot separate a pre-fall state from the ordinary consequences of walking, nor exclude that both the movement and the fall follow from an unmeasured third factor such as fatigue or a fluctuation in symptoms. The result also does not support a fall-prediction alarm. Within person, an OR near two per SD cannot produce a usable positive predictive value when the probability of a fall in any given five minutes is very small.

The analysis does establish three narrower, methodological points. Within-person antecedents of real-world falls can be estimated from continuous monitoring. The time-stratified case-crossover design is workable for them at this event count. The activity-matched contrast separates a claim about the pre-fall interval from a restatement of when people fall.

This distinction matters when the result is compared with the wearable fall-sensing literature, which is mostly concerned with two other tasks. Fall *detection*, recognizing that a fall has occurred from the impact signature, is comparatively tractable, but its accuracy falls sharply when models trained on simulated falls are applied to real-world recordings [18]. Fall-*risk assessment*, a score for a person’s standing propensity to fall, is the wearable analogue of the between-person designs discussed above and has been reviewed extensively [19,20]. Real-world walking in MS differs substantially from walking observed in the laboratory [21], which is a further reason to estimate antecedents in the free-living setting. A growing body of work also applies machine learning to fall risk in MS within the between-person framing [22].

### 4.2. Two Signal-Processing Decisions That Affect the Results

Two choices that would ordinarily pass without comment in a Methods section turned out to determine which features reached significance, and both are worth generalizing.

**Dividing by an observed sampling interval is unsafe when a feature is a maximum.** The archive’s sampling is irregular enough that coverage fractions above one occur in 11.7% of primary candidate windows. Computing jerk as a first difference divided by the observed interval gives any anomalously short interval unbounded influence over a feature defined as a maximum across 15,000 samples. In these data, that inflates peak jerk by a median factor of 1.21 and by up to 6.0-fold, which is enough to make the feature significant. On a uniform time base it is not significant at any lag. A peak-jerk antecedent of falls is therefore not supported by these data.

**Gravity was removed per axis, and the choice changes the effect size.** Gravity is a vector, so removing it requires low-pass filtering each axis and subtracting the estimated gravity vector before taking a norm [14,15], which is the procedure Section 2.4 describes. The magnitude-based alternative is a different measurement. The RMS of the mean-removed acceleration *magnitude* is algebraically the standard deviation of that magnitude, and the two correlate at *r* = 1.0000 in these data because they are the same quantity. Both measures agree on the direction and the significance of the movement finding but not on its size, 3.05 against 2.39. Studies that report gravity-removed acceleration should therefore state whether gravity was removed per axis or from the magnitude, because the choice changes effect sizes.

### 4.3. Why the Within-Person Design Suits This Cohort

Three participants contribute 70.8% of events. A between-person model fitted here would in effect describe three people, and its apparent precision would be an artifact of treating their many falls as independent observations. In the case-crossover design, these three participants instead contribute many within-person contrasts, each internally controlled, and the estimate does not depend on comparing them with anyone else.

The design also has a cost. The 13 participants who never fell contribute nothing to the primary model. This follows from the within-person design, but it means that the primary estimate rests on 12 people. The leave-one-participant-out analysis showed that the movement effect survives the removal of any one of them, including each of the three dominant contributors. The two features that are unstable here, spectral entropy and gait-band power fraction, are reported as unstable and are not claimed.

### 4.4. Limitations

**The released falls are a selected set.** The archive carries 54 fall records in 12 participants where the parent analysis of the same program reports 105 in 17 [7], and the selection rule is not documented. What can be measured is that the retained falls sit on recorded time far more often than matched referent candidates do (Section 2.1, Table 5). Falls that occurred while the device was not recording are therefore largely absent, and because device wear is not independent of being up and active, the release is plausibly enriched for falls during movement. This bears directly on the activity contrast of Section 3.4: the residual association there is estimated within a fall sample already tilted toward ambulation, and the direction of the resulting bias cannot be assigned from the public release.

**Activity is not fully separable from the exposure.** The movement-intensity term and any marker of activity measure overlapping things, so adjusting or restricting on activity removes part of the exposure by construction. The attenuated estimates in Section 3.4 should be read as lower bounds rather than as unbiased estimates of a residual effect, and the walking-referent specification, which is selected on two model predictors, is the most affected.

**The lag family is nested.** The four lagged designs share most of their data with the primary one (Table 4). They bound the annotation problem only in the weak sense that an association confined to the annotated instant would not persist across a 30 s displacement. They cannot quantify how much of the primary estimate reflects timestamp imprecision, and no ordering among their point estimates is interpreted. The same limitation applies to the lag family refitted within ambulatory windows (Section 3.4), which shows that displacement and activity matching survive together but inherit the same nesting.

**Label ascertainment may not have been blind to the sensor stream.** The original study used these recordings to develop a fall detector, so timestamps may have been refined against the signal. This is stated in Section 2.1, and it is the reason the lag analysis is reported as a primary result rather than a sensitivity check.

**Few events and no clinical characterization.** Everything above rests on 48 falls in 12 people, and the public release carries no age, sex, disease duration, phenotype, or disability score (Section 2.2), so the analytic sample cannot be described clinically and no subgroup analysis is possible. The participants were also enrolled in a fall-prevention trial while being monitored [13], which further limits how far the estimates extend to an unselected MS population.

Two features are unstable—spectral entropy and gait-band power fraction change significance under single-participant removal—and neither is claimed as a finding.

The room proxy is not rooms: ToF room labels are a per-participant clustering of tracked positions sampled through analysis windows, not ground-truth room identity, and no ToF feature reached significance in any model.

Validation is needed. The next step is a continuous home-monitoring cohort in which falls are ascertained independently of the sensor stream, by diary or caregiver report, and in which wear time is recorded separately so that unrecorded falls can be counted rather than inferred. The method transfers directly. The window construction, referent rule, and conditional model apply unchanged to any archive with per-day inertial files and timestamped events, and the ambulation marker requires only a posture-transition estimate. The threshold that matters clinically has not yet been reached: an activity-matched OR whose lower confidence bound stays above one in the most stringent referent specification. In these data, that bound is 0.86. Reaching it would need either an independently ascertained fall set or additional events roughly equivalent to an order of magnitude.

## 5. Conclusions

In continuous home monitoring of people with MS, the five minutes before a fall carried more dynamic movement than matched referent intervals in the same person at the same time of day (OR 3.05 per SD, 95% CI 1.66–5.63). The estimate did not depend on where the window ended or which days supplied the referents. Moving the right edge back by 10, 30, or 60 s left it intact, and referents from days before the fall gave 3.46 (1.67–7.16).

Most of that association is motion itself. A binary marker of whether the participant moved at all carried a larger OR than the continuous term. Holding activity comparable reduced the movement term to 2.11 (1.17–3.79) within ambulatory windows and to 1.38 (0.86–2.22) against referents drawn from walking periods. Falls in this cohort therefore occur while people are in motion, with a weaker graded tendency for the pre-fall minutes to be more vigorous than comparable moving minutes.

Three practical points follow. A conditional logistic model on matched within-person windows is the appropriate estimator at this event count. A gradient-boosted model on the same recordings adds a feature ranking but not independent confirmation. A monitoring system would need to start from an ambulation gate rather than from a movement-intensity threshold, because most of the discrimination lies there. An intensity threshold added to such a gate is worth at most a factor of two in odds and cannot support an alarm on its own. Before any such system is evaluated clinically, it should be validated in the independently ascertained cohort described in Section 4.4, with an activity-matched lower confidence bound above one as the entry criterion.

Two methodological points apply beyond this dataset. First, a case window that ends at an annotated event time cannot by itself distinguish an antecedent state from the event, and window-length sensitivity analyses do not test the distinction because they share a right edge. Moving the window does test it at little cost. It should be standard wherever an antecedent claim rests on an annotation of uncertain precision, provided that the resulting designs are reported as the nested family they are. Second, an antecedent claim about a rare event needs referents matched on activity. Otherwise, events that require being in motion will reproduce the activity contrast and present it as a finding about the interval.

## Figures and Tables

**Table 1:** Cohort as stated in the dataset documentation and as measured from the archive.

Quantity	Dataset Readme	As Measured
Participants in the archive	25	26
Participants after the calibration audit (Section 2.5)	—	25
Fall records	not stated	54 (49 after removing 5 exact duplicates)
Fall events analyzed (Section 2.6)	—	48
Monitored days per participant	—	22–74 (median 49)
Participants with ≥1 analyzed fall	—	12 of 25

All analyses use the measured values. The readme states that the source study used participants va_5 to va_33, or 25 in total. The archive also contains va_4. The calibration audit of Section 2.5 excludes va_4 on measured grounds, which returns the analysis to exactly the cohort the original investigators used. Five fall records were exact duplicates by participant and timestamp and were removed.

**Table 2:** Participant characteristics derived from the public release.

Participant	Analyzed	Monitored Days	Calendar Span (Days)	Fall Records	Analyzed Falls	Matched Referents	Usable-Coverage Fraction	Ambulatory Fraction
va_4	no	25	32	0	0	0	0.292	0.429
va_5	yes	60	63	0	0	0	0.292	0.857
va_6	yes	49	62	16	12	72	0.636	0.497
va_7	yes	44	61	0	0	0	0.500	0.417
va_8	yes	40	46	0	0	0	0.583	0.429
va_9	yes	56	57	2	2	12	0.868	0.333
va_10	yes	66	66	0	0	0	0.333	0.500
va_11	yes	63	64	15	13	77	0.850	0.624
va_12	yes	60	60	0	0	0	0.583	0.429
va_13	yes	57	67	2	2	12	0.842	0.688
va_14	yes	55	65	9	9	52	0.737	0.270
va_15	yes	49	65	0	0	0	0.250	0.333
va_16	yes	22	29	1	1	6	0.684	0.385
va_17	yes	24	34	0	0	0	0.250	0.667
va_18	yes	53	62	0	0	0	0.292	0.429
va_19	yes	32	63	0	0	0	0.375	0.111
va_20	yes	47	65	1	1	6	0.632	0.583
va_21	yes	37	55	2	2	12	0.500	0.632
va_22	yes	41	65	0	0	0	0.500	0.250
va_23	yes	56	57	0	0	0	0.667	0.188
va_24	yes	41	75	1	1	6	0.526	0.300
va_25	yes	57	66	0	0	0	0.500	0.250
va_26	yes	63	78	2	2	12	0.684	0.885
va_28	yes	46	64	0	0	0	0.167	0.000
va_30	yes	45	68	1	1	6	0.737	0.286
va_33	yes	74	74	2	2	12	0.711	0.593

One row per archived participant. “Analyzed” is “no” only for va_4, who was excluded by the calibration audit of Section 2.5. The usable-coverage fraction is the proportion of the participant’s candidate windows that meet the 0.9 inertial-coverage gate. The ambulatory fraction is the proportion of usable windows that contain at least one posture transition, the activity marker defined in Section 2.4. Participants with no analyzed fall contribute no matched referents and no term to the conditional likelihood. Their 4 to 8 windows each, 94 in all, are drawn at random clock times and enter only the gradient-boosted model (Section 3.1 and Section 3.7). They are counted separately in Table S33. The release carries no clinical variables, so none appear here.

**Table 3:** Primary conditional logistic regression model.

Predictor (per 1 SD)	OR	95% CI	*p*
Dynamic acceleration RMS, gravity-removed	3.05	1.66–5.63	<0.001
Gait-band power fraction	1.87	0.96–3.65	0.068
Spectral entropy	1.71	1.01–2.89	0.045
Peak jerk	1.29	0.88–1.89	0.193
Posture-transition rate	1.14	0.82–1.58	0.434
Dominant frequency	1.07	0.61–1.88	0.807

Conditional logistic regression stratified on participant, with 5 min windows ending at the annotated fall time and odds ratios per standard deviation; 333 windows, 48 cases, 12 informative strata.

**Table 4:** Movement term across the five lag specifications.

Lag Before the Annotated Fall	Overlap with Primary Window	Cases	OR per SD	95% CI	*p*
0 s (primary)	100.0%	48	3.05	1.66–5.63	<0.001
10 s	96.7%	48	3.29	1.81–6.01	<0.001
30 s	90.0%	48	3.75	1.98–7.09	<0.001
60 s	80.0%	48	2.41	1.33–4.37	0.004
120 s	60.0%	47	1.32	0.76–2.28	0.327

Gravity-removed dynamic acceleration RMS per standard deviation. Each row is a complete case-crossover design, with the stated lag applied to both arms. The overlap column gives the share of each window’s span that it holds in common with the primary window. The 120 s row loses one further event to the coverage gate, because its window reaches further back into the record. The designs share most of their data, so the differences among the first four rows are not interpretable as the association varying with lag.

**Table 5:** Referent-selection and activity contrasts for the movement term.

Specification	Windows	Cases	Strata	OR per SD	95% CI	*p*
Primary model	333	48	12	3.05	1.66–5.63	<0.001
Pre-fall referent days only	185	44	9	3.46	1.67–7.16	<0.001
Post-fall referent days only	192	48	12	2.94	1.26–6.85	0.012
Adjusted for ambulation marker	333	48	12	2.42	1.31–4.49	0.005
Restricted to ambulatory windows	173	46	12	2.11	1.17–3.79	0.013
Walking referents, full model	266	48	12	1.38	0.86–2.22	0.183
Walking referents, movement term alone	266	48	12	1.73	1.23–2.43	0.002
Ambulation marker, fitted on its own	333	48	12	5.69	2.76–11.75	<0.001
Lag 0 s, ambulatory windows	173	46	12	2.11	1.17–3.79	0.013
Lag 10 s, ambulatory windows	175	43	12	2.37	1.29–4.37	0.006
Lag 30 s, ambulatory windows	180	41	12	2.51	1.34–4.70	0.004
Lag 60 s, ambulatory windows	180	40	11	1.53	0.82–2.86	0.182
Lag 120 s, ambulatory windows	162	33	11	0.89	0.48–1.64	0.705

All rows report the gravity-removed dynamic acceleration term per standard deviation except the ambulation-marker row, which reports the marker itself. The five lag rows restrict both arms to ambulatory windows and drop the posture-transition rate from the predictor set, since it defines the restriction. These are five-predictor models and are not directly comparable with Table 3. They are nested in one another exactly as the designs in Table 4 are. The fall-capture comparison of Section 2.1, from the same candidate pool, shows that 98.0% of 49 case windows meet the 0.9 coverage gate against 72.0% of 882 clock-matched referent candidates (binomial *p* = 2.0 × 10^−6^), and 0.0% against 23.1% contain no inertial samples at all.

## Data Availability

All data analyzed in this study are publicly available and were not generated by the authors. The recordings are the open-access dataset Real-World Falls in Multiple Sclerosis (MS), deposited on IEEE DataPort under a Creative Commons Attribution 4.0 license (https://doi.org/10.21227/xcnr-nz43, accessed on 1 August 2026) [23]. The dataset accompanies the study of Mosquera-Lopez and colleagues [12], which describes the recording protocol, the participants, the ascertainment of the fall timestamps used here as labels, and the ethics approval under which the recordings were collected. The dataset has three components, all used here. The inertial directory ms_ohsu_raw_imu_data_04152018 holds one compressed file of 50 Hz tri-axial accelerometry per participant-day. The tracking directory ms_ohsu_raw_tof_data_09042019 holds 4 Hz time-of-flight position and velocity states in the same layout. The ground-truth file falls_groundtruth.csv maps fall timestamps to participant identifiers. Derived results generated in this study, namely the cohort manifest, participant characteristics, window tables, feature tables, model tables, and audit tables, are provided in Supplementary Files S2 and S3. No restricted-access or identifiable data were used. The analysis code is provided in Supplementary File S1.

## Supplementary Materials

The following supporting information can be downloaded at: https://www.mdpi.com/article/10.3390/jcm15218323/s1. Supplementary File S1: Reproducible Analysis Code, a formatted listing of the analysis pipeline. Supplementary File S2: Supplementary Tables, comprising the cohort manifest; the participant characteristics; the duplicate-event report; the participant calibration audit and the windows it excludes; column summaries of the candidate and final window tables; the selection accounting for every window specification, including the events lost to the symmetric coverage gate; the coverage-versus-feature association check; the fall-capture comparison; the referent-day balance, the pre-fall referent refits, and the referent-timing refits; the archive identifier coverage; the ambulation marker by arm; the activity-matched contrasts; the excluded-predictor audit and the full post hoc collinearity screen; the complete conditional-model estimates for every specification, with variance-inflation factors; the standardization-population comparison; the sampling time-base comparison; the full leave-one-participant-out analysis and its stability summary; the permutation null distribution and the leave-one-feature-out check; the archive-integrity verification; the leakage audit; the descriptive statistics by case and referent status; and the gradient-boosted model’s cross-validation folds, TreeSHAP importances, and feature-ranking comparison. Supplementary File S3: Window Tables, a spreadsheet with the complete candidate window index and the candidate and final window feature tables.

## Abbreviations

The following abbreviations are used in this manuscript:

AUROC	area under the receiver operating characteristic curve
CI	confidence interval
IMU	inertial measurement unit
MS	multiple sclerosis
OR	odds ratio
RMS	root mean square
SD	standard deviation
ToF	time of flight
