# Supplementary material for: Movement Intensity in the Minutes Before a Fall: A Lag-Controlled, Activity-Matched Time-Stratified Case-Crossover Analysis of Home Monitoring in Multiple Sclerosis
Source: J Clin Med. 2026 Oct 26;15(21):8323. doi: 10.3390/jcm15218323 (PMC13764606; doi:10.3390/jcm15218323)
Supplement: Supplementary file 1 [file jcm-15-08323-s001.zip › Supplement_S1_Reproducible_Analysis_Code.pdf]

Movement Intensity in the Minutes Before a Fall: A Lag-Controlled, Activity-Matched Time-Stratified Case-Crossover Analysis of Home Monitoring in Multiple Sclerosis

Supplementary File S1: Reproducible Analysis Code

This document contains the complete, formatted source code accompanying the article above, organized by file. Each listing is reproduced verbatim from the reproducible analysis pipeline; line numbers are added for reference only.

Contents

1 src/config.py 2

2 src/archive.py 3

3 src/features.py 3

4 src/conditional_logit.py 8

5 src/plotting.py 9

6 src/build_manifest.py 12

7 src/deduplicate_falls.py 14

8 src/build_window_index.py 15

9 src/verify_archive_integrity.py 19

10 src/extract_features.py 22

11 src/select_windows.py 27

12 src/leakage_audit.py 30

13 src/fit_conditional_models.py 32

14 src/jerk_time_base_comparison.py 35

15 src/leave_one_participant_out.py 37

16 src/permutation_control.py 40

17 src/fit_boosted_model.py 42

18 src/referent_and_activity_analyses.py 49

19 src/descriptive_tables.py 58

20 src/make_lag_figure.py 60

21 src/make_referent_activity_figure.py 62

22 scripts/run_all.py 63

1 src/config.py

```python
from __future__ import annotations

import logging
import os
from pathlib import Path

PAPER_DIR = Path(__file__).resolve().parents[1]

ARCHIVE = Path(
    os.environ.get(
        "MS_FALL_ARCHIVE",
        str(PAPER_DIR / "data" / "raw" / "ohsu-ms-fall-data.zip"),
    )
)
WORK_DIR = Path(
    os.environ.get("MS_FALL_WORK_DIR", str(PAPER_DIR / "data" / "interim"))
)

PROCESSED_DIR = PAPER_DIR / "data" / "processed"
TABLES_DIR = PAPER_DIR / "results" / "tables"
METRICS_DIR = PAPER_DIR / "results" / "metrics"
FIGURES_DIR = PAPER_DIR / "results" / "figures"

SEED = 42
SECONDS_PER_DAY = 86400
Z_95 = 1.959963985

IMU_HZ = 50.0
TOF_HZ = 4.0

COVERAGE_MIN = 0.9
TARGET_CONTROLS_PER_CASE = 6
NEG_PARTICIPANT_TARGET = 8

PRIMARY_LABEL = "primary_5min"
MOVEMENT_FEATURE = "accel_dynamic_rms_gravity_removed"
LAG_LABELS = [
    "primary_5min",
    "lag10_5min",
    "lag30_5min",
    "lag60_5min",
    "lag120_5min",
]
LENGTH_LABELS = ["primary_5min", "alt_2min", "alt_8min"]
LAG_SECONDS = {
    "primary_5min": 0,
    "lag10_5min": 10,
    "lag30_5min": 30,
    "lag60_5min": 60,
    "lag120_5min": 120,
}

IMU_FEATURES = [
    "accel_dynamic_rms_gravity_removed",
    "jerk_max",
    "dom_freq_hz",
    "spectral_entropy",
    "gait_band_power_frac",
    "posture_transition_rate",
]
FEATURE_LABELS = {
    "accel_mag_mean": "Mean acceleration magnitude",
    "accel_dynamic_rms_gravity_removed": (
        "Dynamic acceleration RMS,\ngravity-removed"
    ),
    "jerk_mean": "Mean jerk",
    "jerk_max": "Peak jerk",
    "dom_freq_hz": "Dominant frequency",
    "spectral_entropy": "Spectral entropy",
    "gait_band_power_frac": "Gait-band power fraction",
    "posture_transition_rate": "Posture-transition rate",
    "tof_speed_mean": "Mean speed (ToF)",
    "tof_path_length": "Path length (ToF)",
    "tof_tortuosity": "Tortuosity (ToF)",
```

```
    "tof_room_transition_rate": "Room-transition rate (ToF)",
}
TOF_FEATURES = [
    "tof_speed_mean",
    "tof_path_length",
    "tof_tortuosity",
    "tof_room_transition_rate",
]


def configure_logging() -> None:
    logging.basicConfig(level=logging.INFO, format="%(message)s")


def ensure_output_dirs() -> None:
    for directory in (PROCESSED_DIR, TABLES_DIR, METRICS_DIR, FIGURES_DIR):
        directory.mkdir(parents=True, exist_ok=True)
```

2 src/archive.py

```
from __future__ import annotations

import re
import zipfile
from datetime import UTC, datetime
from pathlib import Path

MEMBER_NAME = re.compile(r"(?P<date>\d{6})_va_(?P<pid>\d+)_", re.IGNORECASE)


def day_string(timestamp: float) -> str:
    return datetime.fromtimestamp(timestamp, tz=UTC).strftime("%y%m%d")


def member_kind(name: str) -> str | None:
    if "raw_imu_data" in name:
        return "imu"
    if "raw_tof_data" in name:
        return "tof"
    return None


def participant_and_day(name: str) -> tuple[str, str] | None:
    match = MEMBER_NAME.search(Path(name).name)
    if not match:
        return None
    return match.group("pid"), match.group("date")


def sensor_file_index(
    archive: zipfile.ZipFile,
) -> dict[tuple[str, str, str], str]:
    index: dict[tuple[str, str, str], str] = {}
    for info in archive.infolist():
        if info.is_dir():
            continue
        parsed = participant_and_day(info.filename)
        kind = member_kind(info.filename)
        if parsed and kind:
            index[(kind, *parsed)] = info.filename
    return index
```

3 src/features.py

```
from __future__ import annotations

import numpy as np
from scipy.signal import butter, filtfilt, welch
from sklearn.cluster import KMeans

```

```python
from config import IMU_HZ, SEED, TOF_HZ

GAIT_BAND = (0.5, 3.0)
SPECTRAL_BAND = (0.3, 10.0)
POSTURE_SMOOTH_SECONDS = 1.0
POSTURE_ANGLE_DEGREES = 30.0
ROOM_CLUSTERS = 6
GRAVITY_CUTOFF_HZ = 0.3
RESAMPLE_MAX_GAP_SECONDS = 1.0
MIN_IMU_SAMPLES = 10
MIN_TOF_SAMPLES = 4
MIN_NET_DISPLACEMENT_M = 0.25
WELCH_SEGMENT_SECONDS = 8
MIN_WELCH_SEGMENT = 8

IMU_SIGNAL_KEYS = (
    "accel_mag_mean",
    "accel_mag_sd",
    "accel_mag_iqr",
    "accel_mag_fluctuation_rms",
    "accel_dynamic_rms_gravity_removed",
    "jerk_mean",
    "jerk_sd",
    "jerk_max",
    "dom_freq_hz",
    "spectral_entropy",
    "gait_band_power_frac",
    "posture_transition_rate",
)
SPECTRAL_KEYS = ("dom_freq_hz", "spectral_entropy", "gait_band_power_frac")
TOF_KEYS = (
    "tof_speed_mean",
    "tof_speed_sd",
    "tof_path_length",
    "tof_net_displacement",
    "tof_tortuosity",
    "tof_room_transition_rate",
)


def imu_window_features(
    times: np.ndarray,
    acceleration: np.ndarray,
    window_start: float,
    window_end: float,
) -> dict:
    in_window = (times >= window_start) & (times < window_end)
    n_samples = int(in_window.sum())
    expected = max(1, round((window_end - window_start) * IMU_HZ))
    features: dict = {
        "imu_n_samples": n_samples,
        "imu_coverage_frac": n_samples / expected,
    }
    if n_samples < max(MIN_IMU_SAMPLES, 0.5 * expected):
        features.update(dict.fromkeys(IMU_SIGNAL_KEYS, np.nan))
        features["jerk_max_observed_interval"] = np.nan
        return features

    order = np.argsort(times[in_window])
    window_acceleration = acceleration[in_window][order]
    window_times = times[in_window][order]
    features.update(_interval_diagnostics(window_times))
    features["jerk_max_observed_interval"] = _observed_interval_jerk_max(
        window_times, window_acceleration
    )

    grid, resampled, valid = _uniform_resample(
        window_times, window_acceleration, window_start, window_end
    )
    if grid is None or valid.sum() < max(MIN_IMU_SAMPLES, 0.5 * len(grid)):
        features.update(dict.fromkeys(IMU_SIGNAL_KEYS, np.nan))
        features["resampled_valid_frac"] = (
            float(valid.mean()) if valid is not None else 0.0
        )
        return features
    features["resampled_valid_frac"] = float(valid.mean())
```

```python
    features.update(_amplitude_features(resampled, valid))
    features.update(_jerk_features(resampled, valid))
    magnitude = np.linalg.norm(resampled[valid], axis=1)
    features.update(_spectral_features(magnitude - magnitude.mean()))
    features["posture_transition_rate"] = _posture_transition_rate(
        resampled[valid], window_end - window_start
    )
    return features


def _interval_diagnostics(window_times: np.ndarray) -> dict:
    intervals = np.diff(window_times)
    nominal = 1.0 / IMU_HZ
    if not intervals.size:
        return {
            "imu_dt_median_s": np.nan,
            "imu_dt_min_s": np.nan,
            "imu_dt_frac_below_half_nominal": np.nan,
            "imu_dt_frac_nonpositive": np.nan,
        }
    return {
        "imu_dt_median_s": float(np.median(intervals)),
        "imu_dt_min_s": float(intervals.min()),
        "imu_dt_frac_below_half_nominal": float(
            np.mean(intervals < 0.5 * nominal)
        ),
        "imu_dt_frac_nonpositive": float(np.mean(intervals <= 0)),
    }


def _observed_interval_jerk_max(
    window_times: np.ndarray, window_acceleration: np.ndarray
) -> float:
    magnitude = np.linalg.norm(window_acceleration, axis=1)
    intervals = np.diff(window_times)
    keep = intervals > 0
    jerk = np.diff(magnitude)[keep] / intervals[keep]
    return float(np.max(np.abs(jerk))) if jerk.size else np.nan


def _uniform_resample(
    times: np.ndarray,
    acceleration: np.ndarray,
    window_start: float,
    window_end: float,
) -> tuple:
    n_grid = int(round((window_end - window_start) * IMU_HZ))
    if n_grid < 2 or times.size < 2:
        return None, None, None
    grid = window_start + np.arange(n_grid) / IMU_HZ
    resampled = np.column_stack(
        [
            np.interp(grid, times, acceleration[:, axis])
            for axis in range(acceleration.shape[1])
        ]
    )
    valid = np.ones(n_grid, dtype=bool)
    gaps = np.diff(times)
    for index in np.flatnonzero(gaps > RESAMPLE_MAX_GAP_SECONDS):
        valid &= ~((grid >= times[index]) & (grid <= times[index + 1]))
    valid &= (grid >= times[0]) & (grid <= times[-1])
    return grid, resampled, valid


def _amplitude_features(resampled: np.ndarray, valid: np.ndarray) -> dict:
    magnitude = np.linalg.norm(resampled[valid], axis=1)
    upper, lower = np.percentile(magnitude, [75, 25])
    fluctuation = magnitude - magnitude.mean()
    features = {
        "accel_mag_mean": float(np.mean(magnitude)),
        "accel_mag_sd": float(np.std(magnitude)),
        "accel_mag_iqr": float(upper - lower),
        "accel_mag_fluctuation_rms": float(np.sqrt(np.mean(fluctuation**2))),
    }
    dynamic = _gravity_removed(resampled)
    if dynamic is None:
```

```python
        features["accel_dynamic_rms_gravity_removed"] = np.nan
    else:
        dynamic_norm = np.linalg.norm(dynamic[valid], axis=1)
        features["accel_dynamic_rms_gravity_removed"] = float(
            np.sqrt(np.mean(dynamic_norm**2))
        )
    return features


def _gravity_removed(resampled: np.ndarray) -> np.ndarray | None:
    numerator, denominator = butter(
        4, GRAVITY_CUTOFF_HZ / (IMU_HZ / 2.0), btype="low"
    )
    if resampled.shape[0] <= 3 * max(len(numerator), len(denominator)):
        return None
    gravity = np.column_stack(
        [
            filtfilt(numerator, denominator, resampled[:, axis])
            for axis in range(resampled.shape[1])
        ]
    )
    return resampled - gravity


def _jerk_features(resampled: np.ndarray, valid: np.ndarray) -> dict:
    jerk_all = np.diff(np.linalg.norm(resampled, axis=1)) * IMU_HZ
    jerk = jerk_all[valid[:-1] & valid[1:]]
    if jerk.size == 0:
        return dict.fromkeys(("jerk_mean", "jerk_sd", "jerk_max"), np.nan)
    return {
        "jerk_mean": float(np.mean(np.abs(jerk))),
        "jerk_sd": float(np.std(jerk)),
        "jerk_max": float(np.max(np.abs(jerk))),
    }


def _spectral_features(fluctuation: np.ndarray) -> dict:
    frequencies, power = _welch_psd(fluctuation, IMU_HZ)
    if frequencies is None:
        return dict.fromkeys(SPECTRAL_KEYS, np.nan)
    band = (frequencies >= SPECTRAL_BAND[0]) & (
        frequencies <= SPECTRAL_BAND[1]
    )
    if band.sum() < 2 or power[band].sum() <= 0:
        return dict.fromkeys(SPECTRAL_KEYS, np.nan)
    band_frequencies, band_power = frequencies[band], power[band]
    shares = band_power / band_power.sum()
    entropy = -np.sum(shares * np.log(shares + 1e-300))
    gait = (band_frequencies >= GAIT_BAND[0]) & (
        band_frequencies <= GAIT_BAND[1]
    )
    return {
        "dom_freq_hz": float(band_frequencies[np.argmax(band_power)]),
        "spectral_entropy": float(entropy / np.log(len(shares))),
        "gait_band_power_frac": float(
            band_power[gait].sum() / band_power.sum()
        ),
    }


def _welch_psd(signal: np.ndarray, sampling_hz: float) -> tuple:
    segment = min(len(signal), int(sampling_hz * WELCH_SEGMENT_SECONDS))
    if segment < MIN_WELCH_SEGMENT:
        return None, None
    return welch(signal, fs=sampling_hz, nperseg=segment, detrend="constant")


def _posture_transition_rate(
    valid_samples: np.ndarray, window_seconds: float
) -> float:
    width = max(1, int(round(POSTURE_SMOOTH_SECONDS * IMU_HZ)))
    if len(valid_samples) < width * 2:
        return np.nan
    kernel = np.ones(width) / width
    smooth = np.column_stack(
        [
```

```
            np.convolve(valid_samples[:, axis], kernel, mode="valid")
            for axis in range(3)
        ]
    )
    norms = np.linalg.norm(smooth, axis=1, keepdims=True)
    norms[norms == 0] = 1.0
    orientation = smooth / norms
    threshold = float(np.cos(np.radians(POSTURE_ANGLE_DEGREES)))
    transitions = 0
    reference = 0
    while reference + 1 < len(orientation):
        dots = orientation[reference + 1 :] @ orientation[reference]
        beyond = np.flatnonzero(dots < threshold)
        if beyond.size == 0:
            break
        reference = reference + 1 + int(beyond[0])
        transitions += 1
    minutes = window_seconds / 60.0
    return float(transitions / minutes) if minutes > 0 else np.nan


def tof_window_features(
    times: np.ndarray,
    states: np.ndarray,
    window_start: float,
    window_end: float,
) -> dict:
    in_window = (times >= window_start) & (times < window_end)
    n_samples = int(in_window.sum())
    expected = max(1, round((window_end - window_start) * TOF_HZ))
    features: dict = {
        "tof_n_samples": n_samples,
        "tof_coverage_frac": n_samples / expected,
    }
    if n_samples < max(MIN_TOF_SAMPLES, 0.5 * expected):
        features.update(dict.fromkeys(TOF_KEYS, np.nan))
        return features
    order = np.argsort(times[in_window])
    window_states = states[in_window][order]
    position, velocity = window_states[:, :3], window_states[:, 3:]
    speed = np.linalg.norm(velocity, axis=1)
    path_length = float(
        np.sum(np.linalg.norm(np.diff(position, axis=0), axis=1))
    )
    net_displacement = float(np.linalg.norm(position[-1] - position[0]))
    features.update(
        {
            "tof_speed_mean": float(np.mean(speed)),
            "tof_speed_sd": float(np.std(speed)),
            "tof_path_length": path_length,
            "tof_net_displacement": net_displacement,
            "tof_tortuosity": (
                float(path_length / net_displacement)
                if net_displacement > MIN_NET_DISPLACEMENT_M
                else np.nan
            ),
            "tof_room_transition_rate": np.nan,
        }
    )
    return features


def fit_room_model(positions: np.ndarray) -> KMeans | None:
    if len(positions) < ROOM_CLUSTERS:
        return None
    model = KMeans(n_clusters=ROOM_CLUSTERS, random_state=SEED, n_init=10)
    model.fit(positions)
    return model
```

4 src/conditional_logit.py

```
from __future__ import annotations

import warnings

import numpy as np
import pandas as pd
from scipy import stats
from statsmodels.discrete.conditional_models import ConditionalLogit
from statsmodels.stats.outliers_influence import variance_inflation_factor

from config import Z_95


def zscore(frame: pd.DataFrame, columns: list[str]) -> pd.DataFrame:
    standardised = frame.copy()
    for column in columns:
        mean, sd = standardised[column].mean(), standardised[column].std()
        standardised[column] = (
            (standardised[column] - mean) / sd if sd > 0 else 0.0
        )
    return standardised


def informative_participants(frame: pd.DataFrame) -> set:
    spread = frame.groupby("va_id")["is_case"].std()
    return set(spread[spread > 0].index)


def fit(frame: pd.DataFrame, columns: list[str], max_iterations: int):
    model = ConditionalLogit(
        frame["is_case"].to_numpy().astype(float),
        frame[columns].to_numpy().astype(float),
        groups=frame["va_id"].to_numpy(),
    )
    return model.fit(method="bfgs", maxiter=max_iterations, disp=False)


def fit_quietly(frame: pd.DataFrame, columns: list[str], max_iterations: int):
    with warnings.catch_warnings():
        warnings.simplefilter("ignore")
        return fit(frame, columns, max_iterations)


def odds_ratio_table(result, columns: list[str], label: str) -> pd.DataFrame:
    params, errors = result.params, result.bse
    z_scores = params / errors
    return pd.DataFrame(
        {
            "specification": label,
            "feature": columns,
            "coef": params,
            "se": errors,
            "z": z_scores,
            "p_value": 2 * (1 - stats.norm.cdf(np.abs(z_scores))),
            "or": np.exp(params),
            "or_ci_lo": np.exp(params - Z_95 * errors),
            "or_ci_hi": np.exp(params + Z_95 * errors),
        }
    )


def fit_specification(
    frame: pd.DataFrame, columns: list[str], label: str
) -> dict:
    complete = frame.dropna(subset=[*columns, "is_case", "va_id"]).copy()
    informative = informative_participants(complete)
    analysis = complete[complete["va_id"].isin(informative)].copy()
    if analysis.empty or analysis["is_case"].sum() == 0:
        return {"specification": label, "error": "no informative stratum"}
    for column in columns:
        mean, sd = analysis[column].mean(), analysis[column].std()
        analysis[column] = (analysis[column] - mean) / sd if sd > 0 else 0.0
    result = fit_quietly(analysis, columns, max_iterations=300)
    table = odds_ratio_table(result, columns, label)
```

```python
    table["n_rows"] = len(analysis)
    table["n_cases"] = int(analysis["is_case"].sum())
    table["n_controls"] = int((analysis["is_case"] == 0).sum())
    table["n_informative_strata"] = len(informative)
    return {"specification": label, "table": table}


def fit_with_diagnostics(
    frame: pd.DataFrame, columns: list[str], label: str
) -> dict:
    complete = frame.dropna(subset=[*columns, "is_case", "va_id"]).copy()
    informative = informative_participants(complete)
    analysis = complete[complete["va_id"].isin(informative)].copy()
    discarded = complete[~complete["va_id"].isin(informative)]
    if analysis.empty or analysis["is_case"].sum() == 0:
        return {
            "label": label,
            "error": "no informative stratum",
            "table": pd.DataFrame(),
            "vif": pd.DataFrame(),
        }
    standardised = zscore(analysis, columns)
    with warnings.catch_warnings(record=True) as caught:
        warnings.simplefilter("always")
        result = fit(standardised, columns, max_iterations=200)
        dropped = [
            str(item.message)
            for item in caught
            if "Dropped" in str(item.message)
        ]
    return {
        "label": label,
        "table": odds_ratio_table(result, columns, label),
        "vif": _vif_table(standardised, columns, label),
        "n_complete_case_rows": int(len(complete)),
        "n_rows_zscored_and_fitted": int(len(analysis)),
        "n_rows_discarded_before_zscoring": int(len(discarded)),
        "n_cases": int(analysis["is_case"].sum()),
        "n_controls": int((analysis["is_case"] == 0).sum()),
        "n_informative_strata": int(len(informative)),
        "n_participants_in_analysis_set": int(analysis["va_id"].nunique()),
        "n_participants_discarded_no_case": int(discarded["va_id"].nunique()),
        "drop_warning": dropped,
        "log_likelihood": float(result.llf),
    }


def _vif_table(
    standardised: pd.DataFrame, columns: list[str], label: str
) -> pd.DataFrame:
    design = standardised[columns].to_numpy().astype(float)
    return pd.DataFrame(
        {
            "specification": label,
            "feature": columns,
            "vif": [
                variance_inflation_factor(design, index)
                for index in range(design.shape[1])
            ],
        }
    )
```

5 src/plotting.py

```python
from __future__ import annotations

import json
from pathlib import Path

import matplotlib as mpl
import numpy as np

PALETTE: dict[str, str] = {
```

```
    "blue": "#0072B2",
    "orange": "#E69F00",
    "green": "#009E73",
    "pink": "#CC79A7",
    "red": "#D55E00",
    "sky": "#56B4E9",
    "yellow": "#F0E442",
    "grey": "#999999",
}
ORDER = ["blue", "orange", "green", "pink", "red", "sky", "yellow", "grey"]
LINESTYLES: list = [
    "-",
    "--",
    "-.",
    ":",
    (0, (3, 1, 1, 1)),
    (0, (5, 1)),
    (0, (1, 1)),
    (0, (3, 5, 1, 5)),
]

WIDTH_FULL = 5.5


def apply_style() -> None:
    mpl.rcParams.update(
        {
            "axes.unicode_minus": True,
            "font.family": "serif",
            "font.size": 10,
            "figure.dpi": 300,
            "savefig.dpi": 300,
            "mathtext.default": "it",
            "mathtext.fontset": "dejavuserif",
            "axes.grid": False,
            "axes.spines.top": False,
            "axes.spines.right": False,
            "legend.frameon": False,
            "axes.titlesize": 10,
        }
    )


def series(index: int) -> dict:
    return {
        "color": PALETTE[ORDER[index % len(ORDER)]],
        "linestyle": LINESTYLES[index % len(LINESTYLES)],
    }


def panel(ax, letter: str) -> None:
    ax.text(
        0.02,
        0.98,
        f"({letter})",
        transform=ax.transAxes,
        fontweight="bold",
        va="top",
        ha="left",
    )


def save(
    fig,
    output_dir: Path,
    name: str,
    *,
    tight: bool = True,
    pad: float = 0.1,
) -> None:
    output_dir.mkdir(parents=True, exist_ok=True)
    keywords = {"bbox_inches": "tight", "pad_inches": pad} if tight else {}
    fig.savefig(output_dir / f"{name}.png", dpi=300, **keywords)
    fig.savefig(output_dir / f"{name}.pdf", **keywords)
    sidecar = output_dir / f"{name}.colors.json"
    sidecar.write_text(
```

```python
        json.dumps(_collect_handles(fig), indent=2), encoding="utf-8"
    )


def _plain(label: str) -> str:
    return label.replace("$", "")


def _collect_handles(fig) -> dict:
    handles: list[dict] = []
    for ax in fig.get_axes():
        handles.extend(_labelled_artists(ax))
        seen = {handle["label"] for handle in handles}
        for container in getattr(ax, "containers", []):
            label = container.get_label()
            if not label or label.startswith("_") or label in seen:
                continue
            entry = {"type": "container", "label": _plain(label)}
            colour = _container_colour(container)
            if colour:
                entry["color"] = colour
            handles.append(entry)
            seen.add(label)
    return {"handles": handles, "n_axes": len(fig.get_axes())}


def _labelled_artists(ax) -> list[dict]:
    found: list[dict] = []
    for line in ax.get_lines():
        label = line.get_label()
        if label and not label.startswith("_"):
            found.append(
                {
                    "type": "line",
                    "label": _plain(label),
                    "color": mpl.colors.to_hex(line.get_color()),
                    "linestyle": str(line.get_linestyle()),
                }
            )
    for patch in ax.patches:
        label = patch.get_label()
        if label and not label.startswith("_"):
            found.append(
                {
                    "type": "patch",
                    "label": _plain(label),
                    "color": mpl.colors.to_hex(patch.get_facecolor()),
                }
            )
    for collection in ax.collections:
        label = collection.get_label()
        if label and not label.startswith("_"):
            found.append({"type": "collection", "label": _plain(label)})
    return found


def _container_colour(container) -> str | None:
    for child in container.get_children():
        if not hasattr(child, "get_facecolor"):
            continue
        face = np.asarray(child.get_facecolor())
        if face.ndim == 2:
            if len(face) == 0:
                continue
            face = face[0]
        return mpl.colors.to_hex(face)
    return None


def forest_figsize(n_rows: int) -> tuple[float, float]:
    return (9.0, max(4.5, n_rows * 0.42))


def adjust_forest(fig) -> None:
    fig.subplots_adjust(left=0.28, right=0.97, top=0.94, bottom=0.10)
```

6 src/build_manifest.py

```
from __future__ import annotations

import io
import json
import logging
import zipfile
from collections import defaultdict
from datetime import datetime

import pandas as pd

import archive
from config import (
    ARCHIVE,
    PROCESSED_DIR,
    TABLES_DIR,
    configure_logging,
    ensure_output_dirs,
)

GROUND_TRUTH_NAME = "falls_groundtruth.csv"
BYTES_PER_GB = 1024**3

logger = logging.getLogger(__name__)


def main() -> None:
    configure_logging()
    ensure_output_dirs()
    with zipfile.ZipFile(ARCHIVE) as zipped:
        infos = zipped.infolist()
        days, bytes_by_kind, unparsed = index_sensor_days(infos)
        falls = read_ground_truth(zipped, infos)
    manifest = build_manifest(days)
    if not falls.empty:
        counts = falls.groupby("va_id").size()
        manifest["falls"] = (
            manifest["pid"].map(counts.to_dict()).fillna(0).astype(int)
        )
        falls.to_csv(PROCESSED_DIR / "falls_groundtruth.csv", index=False)
    manifest.to_csv(TABLES_DIR / "table_cohort_manifest.csv", index=False)
    summary = summarise(manifest, falls, bytes_by_kind, unparsed)
    (PROCESSED_DIR / "manifest_summary.json").write_text(
        json.dumps(summary, indent=2)
    )
    logger.info(json.dumps(summary, indent=2))


def index_sensor_days(
    infos: list[zipfile.ZipInfo],
) -> tuple[dict[str, dict[str, set]], dict[str, int], list[str]]:
    days: dict[str, dict[str, set]] = {
        "imu": defaultdict(set),
        "tof": defaultdict(set),
    }
    bytes_by_kind: dict[str, int] = defaultdict(int)
    unparsed: list[str] = []
    for info in infos:
        if info.is_dir():
            continue
        kind = archive.member_kind(info.filename) or "other"
        bytes_by_kind[kind] += info.file_size
        parsed = archive.participant_and_day(info.filename)
        if not parsed:
            if kind != "other":
                unparsed.append(info.filename)
            continue
        participant, day = parsed
        days["imu" if kind == "imu" else "tof"][participant].add(day)
    return days, bytes_by_kind, unparsed


def read_ground_truth(
    zipped: zipfile.ZipFile, infos: list[zipfile.ZipInfo]
```

```python
) -> pd.DataFrame:
    name = next(
        (
            info.filename
            for info in infos
            if info.filename.lower().endswith(GROUND_TRUTH_NAME)
        ),
        None,
    )
    if name is None:
        logger.warning("%s not found in the archive", GROUND_TRUTH_NAME)
        return pd.DataFrame()
    with zipped.open(name) as handle:
        falls = pd.read_csv(io.BytesIO(handle.read()))
    falls.columns = [column.strip().lower() for column in falls.columns]
    return falls


def build_manifest(days: dict[str, dict[str, set]]) -> pd.DataFrame:
    participants = sorted(set(days["imu"]) | set(days["tof"]), key=int)
    rows = []
    for participant in participants:
        imu_days = sorted(days["imu"].get(participant, ()))
        tof_days = sorted(days["tof"].get(participant, ()))
        all_days = sorted(set(imu_days) | set(tof_days))
        rows.append(
            {
                "participant": f"va_{participant}",
                "pid": int(participant),
                "imu_days": len(imu_days),
                "tof_days": len(tof_days),
                "distinct_days": len(all_days),
                "first_day": all_days[0] if all_days else "",
                "last_day": all_days[-1] if all_days else "",
                "calendar_span_days": _calendar_span(all_days),
            }
        )
    return pd.DataFrame(rows).sort_values("pid")


def _calendar_span(sorted_days: list[str]) -> int:
    if not sorted_days:
        return 0
    first = datetime.strptime(sorted_days[0], "%y%m%d")
    last = datetime.strptime(sorted_days[-1], "%y%m%d")
    return (last - first).days + 1


def summarise(
    manifest: pd.DataFrame,
    falls: pd.DataFrame,
    bytes_by_kind: dict[str, int],
    unparsed: list[str],
) -> dict:
    return {
        "archive_uncompressed_gb": round(
            sum(bytes_by_kind.values()) / BYTES_PER_GB, 2
        ),
        "n_participants_with_data": len(manifest),
        "imu_participant_days": int(manifest["imu_days"].sum()),
        "tof_participant_days": int(manifest["tof_days"].sum()),
        "n_fall_events": int(len(falls)),
        "n_participants_with_falls": (
            int(falls["va_id"].nunique()) if not falls.empty else 0
        ),
        "unparsed_filenames": len(unparsed),
    }


if __name__ == "__main__":
    main()
```

7 src/deduplicate_falls.py

```python
from __future__ import annotations

import json
import logging

import pandas as pd

from config import (
    PROCESSED_DIR,
    TABLES_DIR,
    configure_logging,
    ensure_output_dirs,
)

KEY_COLUMNS = ["va_id", "ts_fall_utc"]
DEDUPLICATION_RULE = (
    "De-duplicated on exact (va_id, ts_fall_utc) pair, keep-first. Two "
    "independent falls sharing the same participant and identical UTC second "
    "are treated as data-entry duplication, not genuine repeated events. "
    "Primary analytic count = de-duplicated; raw count reported alongside "
    "for transparency."
)

logger = logging.getLogger(__name__)


def main() -> None:
    configure_logging()
    ensure_output_dirs()
    falls = load_falls()
    duplicates = duplicate_pairs(falls)
    deduplicated = deduplicate(falls)
    deduplicated.to_csv(PROCESSED_DIR / "falls_deduped.csv", index=False)
    duplicates.to_csv(TABLES_DIR / "table_duplicate_report.csv", index=False)
    summary = {
        "n_raw_rows": len(falls),
        "n_exact_duplicate_pairs": int(len(duplicates)),
        "n_excess_rows_removed": int(duplicates["n_excess_rows"].sum()),
        "n_dedup_events": int(len(deduplicated)),
        "n_participants_raw": int(falls["va_id"].nunique()),
        "n_participants_dedup": int(deduplicated["va_id"].nunique()),
        "rule": DEDUPLICATION_RULE,
    }
    (PROCESSED_DIR / "duplicate_summary.json").write_text(
        json.dumps(summary, indent=2)
    )
    logger.info(json.dumps(summary, indent=2))


def load_falls() -> pd.DataFrame:
    falls = pd.read_csv(PROCESSED_DIR / "falls_groundtruth.csv")
    falls.columns = [column.strip().lower() for column in falls.columns]
    missing = set(KEY_COLUMNS) - set(falls.columns)
    if missing:
        raise ValueError(
            f"falls_groundtruth.csv lacks {sorted(missing)}; rerun "
            "build_manifest.py against the original archive."
        )
    return falls


def duplicate_pairs(falls: pd.DataFrame) -> pd.DataFrame:
    multiplicity = (
        falls.groupby(KEY_COLUMNS)
        .size()
        .reset_index(name="n_rows")
        .sort_values(["n_rows", "va_id"], ascending=[False, True])
    )
    pairs = multiplicity[multiplicity["n_rows"] > 1].copy()
    pairs["n_excess_rows"] = pairs["n_rows"] - 1
    return pairs


def deduplicate(falls: pd.DataFrame) -> pd.DataFrame:
```

```
    unique = falls.drop_duplicates(subset=KEY_COLUMNS, keep="first").copy()
    unique = unique.sort_values(KEY_COLUMNS).reset_index(drop=True)
    unique["event_id"] = [
        f"F{number:03d}" for number in range(1, len(unique) + 1)
    ]
    return unique


if __name__ == "__main__":
    main()
```

8 src/build_window_index.py

```
from __future__ import annotations

import json
import logging
import random
import zipfile
from collections import defaultdict
from datetime import UTC, datetime, timedelta

import pandas as pd

import archive
from config import (
    ARCHIVE,
    PROCESSED_DIR,
    SECONDS_PER_DAY,
    SEED,
    TABLES_DIR,
    TARGET_CONTROLS_PER_CASE,
    configure_logging,
    ensure_output_dirs,
)

WINDOW_SPECIFICATIONS = {
    "primary_5min": (300, 0),
    "alt_2min": (120, 0),
    "alt_8min": (480, 0),
    "lag10_5min": (300, 10),
    "lag30_5min": (300, 30),
    "lag60_5min": (300, 60),
    "lag120_5min": (300, 120),
}
MAX_REACH_SECONDS = max(
    length + lag for length, lag in WINDOW_SPECIFICATIONS.values()
)
NEGATIVE_SPECIFICATIONS = {
    label: spec
    for label, spec in WINDOW_SPECIFICATIONS.items()
    if spec[1] == 0
}
CANDIDATE_MULTIPLIER = 3
NEGATIVE_PARTICIPANT_TARGET = 8
NEGATIVE_CANDIDATE_MULTIPLIER = 3
NEGATIVE_DRAWS_PER_DAY = 4
WASHOUT_BEFORE_SECONDS = 2 * 3600
WASHOUT_AFTER_SECONDS = 24 * 3600
THINNEST_EVENTS_LOGGED = 8

logger = logging.getLogger(__name__)


def main() -> None:
    configure_logging()
    ensure_output_dirs()
    random_generator = random.Random(SEED)
    imu_days, tof_days = scan_sensor_days()
    falls = pd.read_csv(PROCESSED_DIR / "falls_deduped.csv")
    manifest = pd.read_csv(TABLES_DIR / "table_cohort_manifest.csv")
    manifest["pid_str"] = manifest["pid"].astype(str)
    windows, summary = build_windows(
```

```python
        falls, manifest, imu_days, tof_days, random_generator
    )
    windows.to_csv(TABLES_DIR / "table_windows_candidate.csv", index=False)
    (PROCESSED_DIR / "windows_candidate_summary.json").write_text(
        json.dumps(summary, indent=2)
    )
    logger.info(json.dumps(summary, indent=2))
    log_thinnest_events(windows)


def scan_sensor_days() -> tuple[dict[str, set], dict[str, set]]:
    imu_days: dict[str, set] = defaultdict(set)
    tof_days: dict[str, set] = defaultdict(set)
    with zipfile.ZipFile(ARCHIVE) as zipped:
        for info in zipped.infolist():
            if info.is_dir():
                continue
            parsed = archive.participant_and_day(info.filename)
            kind = archive.member_kind(info.filename)
            if not parsed or kind is None:
                continue
            participant, day = parsed
            (imu_days if kind == "imu" else tof_days)[participant].add(day)
    return imu_days, tof_days


def build_windows(
    falls: pd.DataFrame,
    manifest: pd.DataFrame,
    imu_days: dict[str, set],
    tof_days: dict[str, set],
    random_generator: random.Random,
) -> tuple[pd.DataFrame, dict]:
    falls_by_participant: dict[str, list[float]] = defaultdict(list)
    for _, fall in falls.iterrows():
        falls_by_participant[str(int(fall["va_id"]))].append(
            float(fall["ts_fall_utc"])
        )
    case_rows: list[dict] = []
    referent_rows: list[dict] = []
    for _, fall in falls.iterrows():
        participant = str(int(fall["va_id"]))
        case_rows.extend(_case_windows(fall))
        shifts = eligible_referent_shifts(
            float(fall["ts_fall_utc"]),
            imu_days.get(participant, set()),
            falls_by_participant[participant],
        )
        random_generator.shuffle(shifts)
        picked = shifts[: TARGET_CONTROLS_PER_CASE * CANDIDATE_MULTIPLIER]
        referent_rows.extend(
            _referent_windows(fall, picked, tof_days.get(participant, set()))
        )
    negative_rows = unanchored_windows(
        manifest,
        set(falls_by_participant),
        imu_days,
        tof_days,
        random_generator,
    )
    windows = pd.concat(
        [
            pd.DataFrame(case_rows),
            pd.DataFrame(referent_rows),
            pd.DataFrame(negative_rows),
        ],
        ignore_index=True,
        sort=False,
    )
    summary = _summary(
        falls,
        windows,
        len(referent_rows),
        len(negative_rows),
        manifest,
        set(falls_by_participant),
```

```
    )
    return windows, summary


def _case_windows(fall: pd.Series) -> list[dict]:
    fall_time = float(fall["ts_fall_utc"])
    rows = []
    for label, (length, lag) in WINDOW_SPECIFICATIONS.items():
        window_end = fall_time - lag
        rows.append(
            {
                "window_id": f"{fall['event_id']}_case_{label}",
                "event_id": fall["event_id"],
                "va_id": int(fall["va_id"]),
                "is_case": 1,
                "length_label": label,
                "length_s": length,
                "lag_s": lag,
                "window_start_utc": window_end - length,
                "window_end_utc": window_end,
                "anchor_fall_ts": fall_time,
                "clock_hour_utc": _utc_hour(window_end),
            }
        )
    return rows


def eligible_referent_shifts(
    fall_time: float, monitored_days: set, participant_falls: list[float]
) -> list[tuple[str, int]]:
    excluded = _touched_days(fall_time)
    for other_fall in participant_falls:
        excluded |= _touched_days(other_fall)
    fall_day = _day_start(archive.day_string(fall_time))
    shifts = []
    for day in sorted(monitored_days - excluded):
        n_days = round(
            (_day_start(day) - fall_day).total_seconds() / SECONDS_PER_DAY
        )
        shift = n_days * SECONDS_PER_DAY
        shifted_end = fall_time + shift
        if _in_washout(
            shifted_end - MAX_REACH_SECONDS, shifted_end, participant_falls
        ):
            continue
        shifts.append((day, shift))
    return shifts


def _touched_days(fall_time: float) -> set[str]:
    return {
        archive.day_string(fall_time - MAX_REACH_SECONDS),
        archive.day_string(fall_time),
    }


def _day_start(day: str) -> datetime:
    return datetime.strptime(day, "%y%m%d").replace(tzinfo=UTC)


def _in_washout(
    window_start: float, window_end: float, participant_falls: list[float]
) -> bool:
    return any(
        window_end > fall - WASHOUT_BEFORE_SECONDS
        and window_start < fall + WASHOUT_AFTER_SECONDS
        for fall in participant_falls
    )


def _referent_windows(
    fall: pd.Series, picked: list[tuple[str, int]], tof_days: set
) -> list[dict]:
    fall_time = float(fall["ts_fall_utc"])
    rows = []
    for rank, (day, shift) in enumerate(picked):
```

```python
        for label, (length, lag) in WINDOW_SPECIFICATIONS.items():
            window_end = fall_time + shift - lag
            rows.append(
                {
                    "window_id": f"{fall['event_id']}_ctrl{rank:02d}_{label}",
                    "event_id": fall["event_id"],
                    "va_id": int(fall["va_id"]),
                    "is_case": 0,
                    "length_label": label,
                    "length_s": length,
                    "lag_s": lag,
                    "window_start_utc": window_end - length,
                    "window_end_utc": window_end,
                    "anchor_fall_ts": fall_time,
                    "control_day": day,
                    "control_rank": rank,
                    "shift_days": shift // SECONDS_PER_DAY,
                    "has_tof_day": day in tof_days,
                    "clock_hour_utc": _utc_hour(window_end),
                }
            )
    return rows


def _utc_hour(timestamp: float) -> int:
    return datetime.fromtimestamp(timestamp, tz=UTC).hour


def unanchored_windows(
    manifest: pd.DataFrame,
    fall_participants: set[str],
    imu_days: dict[str, set],
    tof_days: dict[str, set],
    random_generator: random.Random,
) -> list[dict]:
    rows: list[dict] = []
    for _, person in manifest.iterrows():
        participant = person["pid_str"]
        monitored = sorted(imu_days.get(participant, set()))
        if participant in fall_participants or not monitored:
            continue
        n_draws = min(
            len(monitored) * NEGATIVE_DRAWS_PER_DAY,
            NEGATIVE_PARTICIPANT_TARGET * NEGATIVE_CANDIDATE_MULTIPLIER,
        )
        for rank in range(n_draws):
            rows.extend(
                _one_unanchored_draw(
                    participant,
                    rank,
                    monitored,
                    tof_days.get(participant, set()),
                    random_generator,
                )
            )
    return rows


def _one_unanchored_draw(
    participant: str,
    rank: int,
    monitored: list[str],
    tof_days: set,
    random_generator: random.Random,
) -> list[dict]:
    day = random_generator.choice(monitored)
    hour = random_generator.randint(0, 23)
    minute = random_generator.randint(0, 59)
    second = random_generator.randint(0, 59)
    window_end = (
        _day_start(day) + timedelta(hours=hour, minutes=minute, seconds=second)
    ).timestamp()
    rows = []
    for label, (length, lag) in NEGATIVE_SPECIFICATIONS.items():
        rows.append(
            {
```

```python
                "window_id": f"NEG_va{participant}_r{rank:02d}_{label}",
                "event_id": f"NEG_va{participant}",
                "va_id": int(participant),
                "is_case": 0,
                "length_label": label,
                "length_s": length,
                "lag_s": lag,
                "window_start_utc": window_end - length,
                "window_end_utc": window_end,
                "anchor_fall_ts": None,
                "control_day": day,
                "control_rank": rank,
                "shift_days": 0,
                "has_tof_day": day in tof_days,
                "clock_hour_utc": hour,
            }
        )
    return rows


def _summary(
    falls: pd.DataFrame,
    windows: pd.DataFrame,
    n_referent_rows: int,
    n_negative_rows: int,
    manifest: pd.DataFrame,
    fall_participants: set[str],
) -> dict:
    return {
        "n_case_events": int(falls.shape[0]),
        "n_case_windows_per_length": int(
            (windows["is_case"] == 1).sum() / len(WINDOW_SPECIFICATIONS)
        ),
        "n_control_candidate_windows_per_length": int(
            n_referent_rows / len(WINDOW_SPECIFICATIONS)
        ),
        "n_negpool_candidate_windows_per_length": int(
            n_negative_rows / len(NEGATIVE_SPECIFICATIONS)
        ),
        "target_controls_per_case": TARGET_CONTROLS_PER_CASE,
        "washout_pre_s": WASHOUT_BEFORE_SECONDS,
        "washout_post_s": WASHOUT_AFTER_SECONDS,
        "window_specs_length_s_lag_s": {
            label: list(spec) for label, spec in WINDOW_SPECIFICATIONS.items()
        },
        "n_fall_participants": int(len(fall_participants)),
        "n_fall_free_participants": int(
            (~manifest["pid_str"].isin(fall_participants)).sum()
        ),
    }


def log_thinnest_events(windows: pd.DataFrame) -> None:
    referents = windows[
        (windows["is_case"] == 0) & (windows["length_label"] == "primary_5min")
    ]
    anchored = referents[referents["anchor_fall_ts"].notna()]
    counts = anchored.groupby("event_id").size().sort_values()
    for event_id, count in counts.head(THINNEST_EVENTS_LOGGED).items():
        logger.info("  %s: %d candidate referent days", event_id, count)


if __name__ == "__main__":
    main()
```

9 src/verify_archive_integrity.py

```python
from __future__ import annotations

import argparse
import json
import logging
import time
```

```python
import zipfile
from pathlib import Path

import pandas as pd

import archive
from config import (
    ARCHIVE,
    PROCESSED_DIR,
    TABLES_DIR,
    configure_logging,
    ensure_output_dirs,
)

READ_CHUNK_BYTES = 1 << 20
PROGRESS_EVERY = 100
READ_ATTEMPTS = 5
MAX_RETRY_DELAY_SECONDS = 30.0
INTEGRITY_NOTE = (
    "Members whose stored CRC does not verify cannot be read. An analysed "
    "window that loses data to one of them is reported as a data-integrity "
    "exclusion, not as an ordinary coverage exclusion."
)

logger = logging.getLogger(__name__)


def main() -> None:
    configure_logging()
    ensure_output_dirs()
    arguments = _parse_arguments()
    needed = referenced_participant_days(arguments.only)
    started = time.time()
    with zipfile.ZipFile(ARCHIVE) as zipped:
        members = members_to_verify(zipped, needed)
        logger.info(
            "%d participant-days referenced; %d archive members to verify",
            len(needed),
            len(members),
        )
        audit = verify_members(zipped, members, started)
    audit.to_csv(TABLES_DIR / "table_archive_integrity.csv", index=False)
    write_summary(audit, len(needed), started)


def _parse_arguments() -> argparse.Namespace:
    parser = argparse.ArgumentParser()
    parser.add_argument(
        "--only", default=None, help="comma-separated participant ids"
    )
    return parser.parse_args()


def referenced_participant_days(only: str | None) -> set[tuple[str, str]]:
    windows = pd.read_csv(TABLES_DIR / "table_windows_candidate.csv")
    windows["pid"] = windows["va_id"].astype(int).astype(str)
    windows["day_start"] = windows["window_start_utc"].apply(
        archive.day_string
    )
    windows["day_end"] = windows["window_end_utc"].apply(archive.day_string)
    if only:
        keep = {value.strip() for value in only.split(",") if value.strip()}
        windows = windows[windows["pid"].isin(keep)]
    needed: set[tuple[str, str]] = set()
    for _, row in windows.iterrows():
        needed.add((row["pid"], row["day_start"]))
        needed.add((row["pid"], row["day_end"]))
    return needed


def members_to_verify(
    zipped: zipfile.ZipFile, needed: set[tuple[str, str]]
) -> list[tuple[str, str, str, str]]:
    index = archive.sensor_file_index(zipped)
    return [
        (kind, participant, day, index[(kind, participant, day)])
```

```
        for (participant, day) in sorted(needed)
        for kind in ("imu", "tof")
        if (kind, participant, day) in index
    ]


def verify_members(
    zipped: zipfile.ZipFile,
    members: list[tuple[str, str, str, str]],
    started: float,
) -> pd.DataFrame:
    rows = []
    for number, (kind, participant, day, name) in enumerate(members, 1):
        status, detail = _read_status(zipped, name)
        rows.append(
            {
                "kind": kind,
                "va_id": int(participant),
                "day": day,
                "member": Path(name).name,
                "declared_size_bytes": zipped.getinfo(name).file_size,
                "status": status,
                "detail": detail,
            }
        )
        if status != "ok":
            logger.warning("%s: %s", status, Path(name).name)
        if number % PROGRESS_EVERY == 0:
            logger.info(
                "  %d/%d verified (%.0fs)",
                number,
                len(members),
                time.time() - started,
            )
    return pd.DataFrame(rows)


def _read_status(zipped: zipfile.ZipFile, name: str) -> tuple[str, str]:
    try:
        _read_to_end(zipped, name)
        return "ok", ""
    except zipfile.BadZipFile as error:
        return "BAD_CRC", str(error)
    except OSError as error:
        logger.warning("read failed for %s (%s); retrying", name, error)
    delay = 1.0
    for attempt in range(1, READ_ATTEMPTS + 1):
        time.sleep(delay)
        delay = min(delay * 2, MAX_RETRY_DELAY_SECONDS)
        try:
            with zipfile.ZipFile(ARCHIVE) as fresh:
                _read_to_end(fresh, name)
            return "ok", ""
        except zipfile.BadZipFile as error:
            return "BAD_CRC", str(error)
        except OSError as error:
            if attempt == READ_ATTEMPTS:
                return "READ_ERROR", f"{type(error).__name__}: {error}"
    return "READ_ERROR", "no attempt made"


def _read_to_end(zipped: zipfile.ZipFile, name: str) -> None:
    with zipped.open(name) as handle:
        while handle.read(READ_CHUNK_BYTES):
            pass


def write_summary(
    audit: pd.DataFrame, n_participant_days: int, started: float
) -> None:
    failed = audit[audit.status != "ok"]
    columns = ["kind", "va_id", "day", "member", "status"]
    summary = {
        "archive": str(ARCHIVE),
        "n_participant_days_referenced": n_participant_days,
        "n_members_verified": int(len(audit)),
```

```
            "n_members_unreadable": int(len(failed)),
            "unreadable_members": failed[columns].to_dict(orient="records"),
            "note": INTEGRITY_NOTE,
            "runtime_s": round(time.time() - started, 1),
        }
        (PROCESSED_DIR / "archive_integrity_summary.json").write_text(
            json.dumps(summary, indent=2)
        )
        logger.info(
            "%d of %d referenced members unreadable", len(failed), len(audit)
        )
        if len(failed):
            logger.warning(failed[columns].to_string(index=False))


if __name__ == "__main__":
    main()
```

10 src/extract_features.py

```
from __future__ import annotations

import argparse
import gc
import io
import json
import logging
import time
import zipfile
from collections import OrderedDict
from pathlib import Path

import numpy as np
import pandas as pd

import archive
import features
from config import (
    ARCHIVE,
    COVERAGE_MIN,
    PROCESSED_DIR,
    TABLES_DIR,
    WORK_DIR,
    configure_logging,
    ensure_output_dirs,
)

CACHE_ENTRIES = 2
CACHE_BYTES = 150 * 2**20
READ_ATTEMPTS = 5
MAX_RETRY_DELAY_SECONDS = 30.0
MIN_ROOM_SAMPLES = 4
DATA_KEYS = {"imu": ("imu_time_utc", "imu_mat"), "tof": ("timestamp", "X")}
CHANNELS = {"imu": 3, "tof": 6}
UNREADABLE_NOTE = (
    "Archive members whose stored CRC does not verify. Windows that depend "
    "on them carry zero samples and are excluded as a data-integrity loss."
)

logger = logging.getLogger(__name__)


def main(argv: list[str] | None = None) -> None:
    configure_logging()
    ensure_output_dirs()
    WORK_DIR.mkdir(parents=True, exist_ok=True)
    arguments = _parse_arguments(argv)
    windows = load_windows()
    participants = sorted(windows["va_id_str"].unique(), key=int)
    with zipfile.ZipFile(ARCHIVE) as zipped:
        index = archive.sensor_file_index(zipped)
        logger.info("file index: %d day-files", len(index))
        parts, unreadable = extract_participants(
```

```
            zipped, index, windows, participants, arguments
        )
    missing = [
        participant
        for participant in participants
        if not _checkpoint(participant).exists()
    ]
    if missing:
        logger.info(
            "%d/%d participants checkpointed; still missing: %s. Re-run to "
            "continue; the feature table is written when all are done.",
            len(parts),
            len(participants),
            ", ".join("va_" + participant for participant in missing),
        )
        return
    table = pd.concat(parts, ignore_index=True, sort=False)
    table.to_csv(TABLES_DIR / "table_features_candidate.csv", index=False)
    write_summary(table, len(participants), unreadable)


def _parse_arguments(argv: list[str] | None) -> argparse.Namespace:
    parser = argparse.ArgumentParser()
    parser.add_argument(
        "--only",
        default=None,
        help="comma-separated participant ids to process this run",
    )
    parser.add_argument(
        "--max",
        type=int,
        default=None,
        help="process at most this many un-checkpointed participants",
    )
    return parser.parse_args(argv)


def load_windows() -> pd.DataFrame:
    windows = pd.read_csv(TABLES_DIR / "table_windows_candidate.csv")
    windows["day_start"] = windows["window_start_utc"].apply(
        archive.day_string
    )
    windows["day_end"] = windows["window_end_utc"].apply(archive.day_string)
    windows["va_id_str"] = windows["va_id"].astype(int).astype(str)
    return windows


def extract_participants(
    zipped: zipfile.ZipFile,
    index: dict[tuple[str, str, str], str],
    windows: pd.DataFrame,
    participants: list[str],
    arguments: argparse.Namespace,
) -> tuple[list[pd.DataFrame], dict[str, str]]:
    wanted = (
        {value.strip() for value in arguments.only.split(",") if value.strip()}
        if arguments.only
        else None
    )
    budget = arguments.max
    parts: list[pd.DataFrame] = []
    unreadable: dict[str, str] = {}
    for participant in participants:
        checkpoint = _checkpoint(participant)
        if checkpoint.exists():
            parts.append(pd.read_parquet(checkpoint))
            continue
        if (wanted is not None and participant not in wanted) or (
            budget is not None and budget <= 0
        ):
            continue
        if budget is not None:
            budget -= 1
        cache = DayCache(zipped, index)
        rows = windows[windows["va_id_str"] == participant]
        result = process_participant(participant, rows, cache)
```

```
        result.to_parquet(checkpoint, index=False)
        parts.append(result)
        unreadable.update(cache.unreadable)
        cache.clear()
        gc.collect()
        logger.info(
            "va_%s: %d rows, %d with inertial coverage >= %.1f",
            participant,
            len(result),
            int((result["imu_coverage_frac"] >= COVERAGE_MIN).sum()),
            COVERAGE_MIN,
        )
    return parts, unreadable


def _checkpoint(participant: str) -> Path:
    return WORK_DIR / f"features_va_{participant}.parquet"


class DayCache:

    def __init__(
        self,
        zipped: zipfile.ZipFile,
        index: dict[tuple[str, str, str], str],
    ) -> None:
        self.zipped = zipped
        self.index = index
        self.unreadable: dict[str, str] = {}
        self._days: OrderedDict = OrderedDict()

    def get(self, kind: str, participant: str, day: str) -> tuple | None:
        key = (kind, participant, day)
        if key in self._days:
            self._days.move_to_end(key)
            return self._days[key]
        value = self._load(kind, participant, day)
        self._days[key] = value
        self._days.move_to_end(key)
        while len(self._days) > CACHE_ENTRIES or (
            len(self._days) > 1 and self._cached_bytes() > CACHE_BYTES
        ):
            self._days.popitem(last=False)
        return value

    def clear(self) -> None:
        self._days.clear()

    def _cached_bytes(self) -> int:
        return sum(
            int(value[0].nbytes) + int(value[1].nbytes)
            for value in self._days.values()
            if value is not None
        )

    def _load(self, kind: str, participant: str, day: str) -> tuple | None:
        name = self.index.get((kind, participant, day))
        if name is None or Path(name).name in self.unreadable:
            return None
        try:
            raw = self._read_member(name)
        except zipfile.BadZipFile as error:
            self.unreadable[Path(name).name] = f"BadZipFile: {error}"
            logger.warning("unreadable member (CRC): %s", name)
            return None
        return _decode_day(raw, kind)

    def _read_member(self, name: str) -> bytes:
        delay = 1.0
        for attempt in range(1, READ_ATTEMPTS + 1):
            try:
                with self.zipped.open(name) as handle:
                    return handle.read()
            except OSError as error:
                if attempt == READ_ATTEMPTS:
                    raise
```

```python
                logger.warning(
                    "read failed for %s (%s); retry %d in %.0fs",
                    name,
                    error,
                    attempt,
                    delay,
                )
                time.sleep(delay)
                delay = min(delay * 2, MAX_RETRY_DELAY_SECONDS)
                self._reopen()
        raise RuntimeError(f"could not read {name}")

    def _reopen(self) -> None:
        try:
            path = self.zipped.filename
            self.zipped.close()
            self.zipped = zipfile.ZipFile(path)
        except OSError:
            logger.warning("could not reopen the archive; keeping handle")


def _decode_day(raw: bytes, kind: str) -> tuple[np.ndarray, np.ndarray]:
    times_key, values_key = DATA_KEYS[kind]
    with np.load(io.BytesIO(raw)) as arrays:
        times = np.asarray(arrays[times_key], dtype=np.float64)
        values = np.asarray(arrays[values_key], dtype=np.float32)
    if len(times) != len(values):
        common = min(len(times), len(values))
        times, values = times[:common], values[:common]
    if times.size and not np.all(np.diff(times) >= 0):
        order = np.argsort(times, kind="stable")
        times, values = times[order], values[order]
    return times, values


def process_participant(
    participant: str, rows: pd.DataFrame, cache: DayCache
) -> pd.DataFrame:
    rows = rows.sort_values(["day_end", "day_start"]).reset_index(drop=True)
    output_rows: list[dict] = []
    positions_by_row: dict[int, np.ndarray] = {}
    for _, row in rows.iterrows():
        start, end = float(row["window_start_utc"]), float(
            row["window_end_utc"]
        )
        days = (row["day_start"], row["day_end"])
        imu_times, imu_values = _window_samples(
            cache, "imu", participant, days, start, end
        )
        tof_times, tof_values = _window_samples(
            cache, "tof", participant, days, start, end
        )
        features_row = dict(row)
        features_row.update(
            features.imu_window_features(
                imu_times.astype(np.float64),
                imu_values.astype(np.float64),
                start,
                end,
            )
        )
        features_row.update(
            features.tof_window_features(
                tof_times.astype(np.float64),
                tof_values.astype(np.float64),
                start,
                end,
            )
        )
        if tof_values.shape[0] >= MIN_ROOM_SAMPLES:
            positions_by_row[len(output_rows)] = tof_values[:, :2].astype(
                np.float64
            )
        output_rows.append(features_row)
    _fill_room_transitions(output_rows, positions_by_row)
    return pd.DataFrame(output_rows)
```

```python


def _window_samples(
    cache: DayCache,
    kind: str,
    participant: str,
    days: tuple[str, str],
    start: float,
    end: float,
) -> tuple[np.ndarray, np.ndarray]:
    day_list = days if days[0] != days[1] else (days[0],)
    times_parts, value_parts = [], []
    for day in day_list:
        loaded = cache.get(kind, participant, day)
        if loaded is None:
            continue
        times, values = loaded
        in_window = (times >= start) & (times < end)
        if len(day_list) == 1 or in_window.any():
            times_parts.append(times[in_window])
            value_parts.append(values[in_window])
    if not times_parts:
        return np.array([]), np.zeros((0, CHANNELS[kind]))
    if len(times_parts) == 1:
        return times_parts[0], value_parts[0]
    return np.concatenate(times_parts), np.concatenate(value_parts)


def _fill_room_transitions(
    output_rows: list[dict], positions_by_row: dict[int, np.ndarray]
) -> None:
    if not positions_by_row:
        return
    room_model = features.fit_room_model(
        np.concatenate(list(positions_by_row.values()), axis=0)
    )
    if room_model is None:
        return
    for row_number, positions in positions_by_row.items():
        row = output_rows[row_number]
        if row.get("tof_n_samples", 0) < MIN_ROOM_SAMPLES:
            continue
        rooms = room_model.predict(positions)
        minutes = (row["window_end_utc"] - row["window_start_utc"]) / 60.0
        row["tof_room_transition_rate"] = (
            float(np.sum(np.diff(rooms) != 0) / minutes)
            if minutes > 0
            else np.nan
        )


def write_summary(
    table: pd.DataFrame, n_participants: int, unreadable: dict[str, str]
) -> None:
    recorded = _integrity_failures()
    for member, detail in unreadable.items():
        recorded.setdefault(member, detail)
    summary = {
        "unreadable_archive_members": recorded,
        "n_unreadable_archive_members": len(recorded),
        "unreadable_note": UNREADABLE_NOTE,
        "n_rows": int(len(table)),
        "n_participants": int(n_participants),
        "imu_coverage_ge_0.9_frac": float(
            (table["imu_coverage_frac"] >= COVERAGE_MIN).mean()
        ),
        "tof_coverage_ge_0.9_frac": float(
            (table["tof_coverage_frac"] >= COVERAGE_MIN).mean()
        ),
        "rows_with_any_tof": int((table["tof_n_samples"] > 0).sum()),
    }
    (PROCESSED_DIR / "feature_extraction_summary.json").write_text(
        json.dumps(summary, indent=2)
    )
    logger.info(json.dumps(summary, indent=2))

```

```python

def _integrity_failures() -> dict[str, str]:
    path = TABLES_DIR / "table_archive_integrity.csv"
    if not path.exists():
        return {}
    audit = pd.read_csv(path)
    return {
        str(row["member"]): f"{row['status']}: {row['detail']}"
        for _, row in audit[audit["status"] != "ok"].iterrows()
    }


if __name__ == "__main__":
    main()
```

11 src/select_windows.py

```python
from __future__ import annotations

import json
import logging

import pandas as pd
from scipy import stats

from config import (
    COVERAGE_MIN,
    NEG_PARTICIPANT_TARGET,
    PRIMARY_LABEL,
    PROCESSED_DIR,
    TABLES_DIR,
    TARGET_CONTROLS_PER_CASE,
    configure_logging,
    ensure_output_dirs,
)

GRAVITY_SCALE_RANGE = (0.8, 1.3)
MIN_ASSOCIATION_ROWS = 10
COVERAGE_CHECK_FEATURES = [
    "accel_dynamic_rms_gravity_removed",
    "accel_mag_fluctuation_rms",
    "jerk_max",
    "dom_freq_hz",
    "spectral_entropy",
    "gait_band_power_frac",
    "posture_transition_rate",
]
EXCLUDED_WINDOW_REASON = (
    "participant excluded: record not on a 1 g scale "
    "(median window-mean magnitude outside 0.8-1.3 g)"
)
COVERAGE_GATE_NOTE = (
    "Applied to cases, matched referents and negative-pool windows alike."
)

logger = logging.getLogger(__name__)


def main() -> None:
    configure_logging()
    ensure_output_dirs()
    candidates = pd.read_csv(TABLES_DIR / "table_features_candidate.csv")
    audit, excluded = calibration_audit(candidates)
    audit.to_csv(TABLES_DIR / "table_calibration_audit.csv", index=False)
    excluded_windows(candidates, excluded).to_csv(
        TABLES_DIR / "table_artifact_windows_excluded.csv", index=False
    )
    n_before = candidates["va_id"].nunique()
    candidates = candidates[~candidates["va_id"].isin(excluded)].copy()
    final, accounting = select_all_labels(candidates)
    final.to_csv(TABLES_DIR / "table_features_final.csv", index=False)
    pd.DataFrame(accounting).to_csv(
        TABLES_DIR / "table_window_selection_by_label.csv", index=False
```

```python
    )
    primary = final[final.length_label == PRIMARY_LABEL]
    association = coverage_feature_association(primary)
    association.to_csv(
        TABLES_DIR / "table_coverage_feature_association.csv", index=False
    )
    referents_per_event(final).to_csv(
        TABLES_DIR / "table_controls_per_event.csv", index=False
    )
    summary = build_summary(
        n_before, excluded, audit, accounting, primary, association
    )
    (PROCESSED_DIR / "window_selection_summary.json").write_text(
        json.dumps(summary, indent=2)
    )
    logger.info(pd.DataFrame(accounting).to_string(index=False))


def calibration_audit(
    candidates: pd.DataFrame,
) -> tuple[pd.DataFrame, list[int]]:
    medians = candidates.groupby("va_id")["accel_mag_mean"].median()
    rows = []
    for participant, median in medians.items():
        within = GRAVITY_SCALE_RANGE[0] <= median <= GRAVITY_SCALE_RANGE[1]
        rows.append(
            {
                "va_id": int(participant),
                "median_accel_mag_mean_g": round(float(median), 4),
                "implied_scale_factor": round(float(median), 2),
                "within_expected_1g_range": bool(within),
                "verdict": (
                    "included"
                    if within
                    else "EXCLUDED: record not on a 1 g scale"
                ),
            }
        )
    audit = pd.DataFrame(rows).sort_values("va_id")
    excluded = audit.loc[~audit["within_expected_1g_range"], "va_id"]
    return audit, [int(participant) for participant in excluded.tolist()]


def excluded_windows(
    candidates: pd.DataFrame, excluded: list[int]
) -> pd.DataFrame:
    columns = [
        "window_id",
        "va_id",
        "event_id",
        "is_case",
        "length_label",
        "accel_mag_mean",
    ]
    removed = candidates.loc[candidates["va_id"].isin(excluded), columns]
    return removed.assign(reason=EXCLUDED_WINDOW_REASON)


def select_all_labels(
    candidates: pd.DataFrame,
) -> tuple[pd.DataFrame, list[dict]]:
    parts, accounting = [], []
    for label in dict.fromkeys(candidates["length_label"]):
        selected, info = select_for_label(candidates, label)
        parts.append(selected)
        accounting.append(info)
    final = (
        pd.concat(parts, ignore_index=True, sort=False)
        .sort_values(["va_id", "event_id", "length_label", "is_case"])
        .reset_index(drop=True)
    )
    return final, accounting


def select_for_label(
    candidates: pd.DataFrame, label: str
```

```
) -> tuple[pd.DataFrame, dict]:
    windows = candidates[candidates["length_label"] == label].copy()
    covered = windows["imu_coverage_frac"] >= COVERAGE_MIN
    event_ids = windows["event_id"].astype(str)
    cases = windows[windows.is_case == 1]
    accepted_cases = windows[(windows.is_case == 1) & covered]
    dropped_cases = cases[~cases.index.isin(accepted_cases.index)]
    referents = windows[
        (windows.is_case == 0) & event_ids.str.startswith("F") & covered
    ]
    referents = referents[
        referents["event_id"].isin(set(accepted_cases["event_id"]))
    ]
    referents = (
        referents.sort_values(["event_id", "control_rank"])
        .groupby("event_id")
        .head(TARGET_CONTROLS_PER_CASE)
    )
    negatives = windows[event_ids.str.startswith("NEG") & covered]
    negatives = (
        negatives.sort_values(["va_id", "control_rank"])
        .groupby("va_id")
        .head(NEG_PARTICIPANT_TARGET)
    )
    selected = pd.concat(
        [accepted_cases, referents, negatives], ignore_index=True, sort=False
    )
    info = {
        "length_label": label,
        "n_cases_candidate": int(len(cases)),
        "n_cases_accepted": int(len(accepted_cases)),
        "n_cases_dropped_low_coverage": int(len(dropped_cases)),
        "dropped_case_event_ids": sorted(dropped_cases["event_id"].tolist()),
        "n_matched_controls": int(len(referents)),
        "n_negpool_windows": int(len(negatives)),
        "controls_per_case": (
            round(len(referents) / len(accepted_cases), 2)
            if len(accepted_cases)
            else None
        ),
        "n_participants": int(selected["va_id"].nunique()),
        "n_participants_with_case": int(
            selected.loc[selected.is_case == 1, "va_id"].nunique()
        ),
    }
    return selected, info


def coverage_feature_association(primary: pd.DataFrame) -> pd.DataFrame:
    rows = []
    for feature in COVERAGE_CHECK_FEATURES:
        if feature not in primary.columns:
            continue
        pairs = primary[["imu_coverage_frac", feature]].dropna()
        if len(pairs) < MIN_ASSOCIATION_ROWS:
            continue
        rho, p_value = stats.spearmanr(
            pairs["imu_coverage_frac"], pairs[feature]
        )
        rows.append(
            {
                "feature": feature,
                "n": int(len(pairs)),
                "spearman_rho": round(float(rho), 4),
                "p_value": float(f"{p_value:.3g}"),
            }
        )
    return pd.DataFrame(rows)


def referents_per_event(final: pd.DataFrame) -> pd.DataFrame:
    referents = final[
        (final.length_label == PRIMARY_LABEL)
        & (final.is_case == 0)
        & final["event_id"].astype(str).str.startswith("F")
    ]
```

```python
    counts = referents.groupby("event_id").size().reset_index()
    counts.columns = ["event_id", "n_controls_accepted"]
    return counts


def build_summary(
    n_before: int,
    excluded: list[int],
    audit: pd.DataFrame,
    accounting: list[dict],
    primary: pd.DataFrame,
    association: pd.DataFrame,
) -> dict:
    return {
        "n_participants_before_calibration_audit": int(n_before),
        "participants_excluded_scale_anomaly": excluded,
        "calibration_audit": audit.to_dict(orient="records"),
        "gravity_scale_ok_range_g": list(GRAVITY_SCALE_RANGE),
        "coverage_min": COVERAGE_MIN,
        "coverage_gate_symmetric": True,
        "coverage_gate_note": COVERAGE_GATE_NOTE,
        "target_controls_per_case": TARGET_CONTROLS_PER_CASE,
        "neg_participant_target": NEG_PARTICIPANT_TARGET,
        "by_label": accounting,
        "coverage_by_arm_accepted_primary": _coverage_by_arm(primary),
        "coverage_feature_association_primary": association.to_dict(
            orient="records"
        ),
    }


def _coverage_by_arm(primary: pd.DataFrame) -> dict:
    case_coverage = primary.loc[
        primary.is_case == 1, "imu_coverage_frac"
    ].dropna()
    referent_coverage = primary.loc[
        primary.is_case == 0, "imu_coverage_frac"
    ].dropna()
    statistic, p_value = stats.mannwhitneyu(
        case_coverage, referent_coverage, alternative="two-sided"
    )
    return {
        "n_case": int(len(case_coverage)),
        "median_case": round(float(case_coverage.median()), 4),
        "n_control": int(len(referent_coverage)),
        "median_control": round(float(referent_coverage.median()), 4),
        "mannwhitney_u": float(statistic),
        "p_value": float(f"{p_value:.3g}"),
    }


if __name__ == "__main__":
    main()
```

12 src/leakage_audit.py

```python
from __future__ import annotations

import logging

import pandas as pd

from config import TABLES_DIR, configure_logging, ensure_output_dirs

FEATURE_FORMULAS = [
    ("accel_mag_mean", "mean(||a(t)||) over the window", "IMU"),
    (
        "accel_mag_fluctuation_rms",
        "RMS of mean-removed ||a(t)||; identical to accel_mag_sd by "
        "construction",
        "IMU",
    ),
    (
```

```python
        "accel_dynamic_rms_gravity_removed",
        "RMS of ||a(t) - g_hat(t)||, gravity estimated per axis by 0.3 Hz "
        "low-pass",
        "IMU",
    ),
    ("jerk_mean", "mean(|d||a||/dt|) over the window", "IMU"),
    ("jerk_max", "max(|d||a||/dt|) over the window", "IMU"),
    (
        "dom_freq_hz",
        "peak-power frequency of the Welch PSD, 0.3-10 Hz band",
        "IMU",
    ),
    (
        "spectral_entropy",
        "normalized Shannon entropy of the PSD, 0.3-10 Hz band",
        "IMU",
    ),
    (
        "gait_band_power_frac",
        "fraction of PSD power in the 0.5-3 Hz gait band",
        "IMU",
    ),
    (
        "posture_transition_rate",
        "count/min of >30-degree orientation-vector deviations",
        "IMU",
    ),
    ("tof_speed_mean", "mean(||v(t)||) from ToF velocity channels", "ToF"),
    (
        "tof_path_length",
        "sum of consecutive-sample ToF position displacements",
        "ToF",
    ),
    ("tof_tortuosity", "ToF path length / net displacement", "ToF"),
    (
        "tof_room_transition_rate",
        "count/min of per-participant k-means cluster-label changes",
        "ToF",
    ),
]
JUSTIFICATION = (
    "Computed purely from the window's raw sensor samples; the window's "
    "is_case label is assigned from its timing relative to a fall timestamp "
    "when the window index is built, before any feature is computed, and is "
    "never used as an input to a feature formula."
)

logger = logging.getLogger(__name__)


def main() -> None:
    configure_logging()
    ensure_output_dirs()
    audit = pd.DataFrame(
        [
            {
                "feature": name,
                "source_sensor": source,
                "formula": formula,
                "derived_from_label": "No",
                "justification": JUSTIFICATION,
            }
            for name, formula, source in FEATURE_FORMULAS
        ]
    )
    audit.to_csv(TABLES_DIR / "table_leakage_audit.csv", index=False)
    mismatches, n_rows = count_label_mismatches()
    logger.info(
        "label consistency: %d mismatches out of %d rows", mismatches, n_rows
    )
    if mismatches:
        raise RuntimeError(
            f"{mismatches} label mismatches between the feature table and "
            "the window index; rebuild both from the same window index."
        )

```

```python
def count_label_mismatches() -> tuple[int, int]:
    index = pd.read_csv(TABLES_DIR / "table_windows_candidate.csv")
    table = pd.read_csv(TABLES_DIR / "table_features_candidate.csv")
    merged = table.merge(
        index[["window_id", "is_case"]],
        on="window_id",
        suffixes=("_feat", "_index"),
    )
    mismatches = int((merged["is_case_feat"] != merged["is_case_index"]).sum())
    return mismatches, len(merged)


if __name__ == "__main__":
    main()
```

13 src/fit_conditional_models.py

```python
from __future__ import annotations

import json
import logging

import numpy as np
import pandas as pd

from conditional_logit import fit_quietly, fit_with_diagnostics, zscore
from config import (
    COVERAGE_MIN,
    IMU_FEATURES,
    LAG_LABELS,
    LENGTH_LABELS,
    METRICS_DIR,
    PRIMARY_LABEL,
    TABLES_DIR,
    TOF_FEATURES,
    configure_logging,
    ensure_output_dirs,
)

IMU_FEATURES_MAGNITUDE_SD = [
    "accel_mag_fluctuation_rms",
    *IMU_FEATURES[1:],
]
MAGNITUDE_SD_SPECIFICATION = "primary_5min_imu_magnitude_sd"
TOF_SPECIFICATION = "primary_5min_imu_tof_complete_case"
SCREEN_FEATURES = [
    "accel_dynamic_rms_gravity_removed",
    "accel_mag_fluctuation_rms",
    "accel_mag_sd",
    "accel_mag_iqr",
    "accel_mag_mean",
    "jerk_mean",
    "jerk_sd",
    "jerk_max",
]
EXCLUSION_REASONS = {
    "accel_mag_mean": (
        "Near-constant by physics: the window-mean magnitude of a worn "
        "accelerometer is dominated by gravity. It carries no within-stratum "
        "movement information once a dynamic-acceleration term is in the "
        "model, while its rare outliers destabilise the fit."
    ),
    "jerk_mean": (
        "Near-duplicate of the dynamic-acceleration term. Its measured "
        "correlation and VIF are in the collinearity screen; the pair is not "
        "separately identifiable at this event count."
    ),
}
SCREEN_NOTE = (
    "The collinearity screen was run after an implausible odds ratio was "
    "observed in a model carrying both dynamic-acceleration terms. It is "
    "post hoc, not pre-specified."
```

```python
)

logger = logging.getLogger(__name__)


def main() -> None:
    configure_logging()
    ensure_output_dirs()
    features = pd.read_csv(TABLES_DIR / "table_features_final.csv")
    write_exclusion_reasons()
    tables, vifs, summaries = fit_all_specifications(features)
    primary_frame = features[features.length_label == PRIMARY_LABEL]
    fit_tof_complete_case(primary_frame, tables, vifs, summaries)
    primary_table = write_specification_tables(tables, vifs)
    write_standardisation_comparison(primary_frame, primary_table)
    write_collinearity_screen(primary_frame)
    summaries["_screen_is_post_hoc"] = SCREEN_NOTE
    (METRICS_DIR / "clr_results_summary.json").write_text(
        json.dumps(summaries, indent=2)
    )
    logger.info("wrote table_clr_all_specifications.csv")


def write_exclusion_reasons() -> None:
    pd.DataFrame(
        [
            {"feature": feature, "reason": reason}
            for feature, reason in EXCLUSION_REASONS.items()
        ]
    ).to_csv(TABLES_DIR / "table_excluded_predictors.csv", index=False)


def fit_all_specifications(
    features: pd.DataFrame,
) -> tuple[list[pd.DataFrame], list[pd.DataFrame], dict]:
    tables: list[pd.DataFrame] = []
    vifs: list[pd.DataFrame] = []
    summaries: dict = {}
    for length_label, name, columns in _specifications(features):
        subset = features[features.length_label == length_label]
        if subset.empty:
            continue
        fitted = fit_with_diagnostics(subset, columns, name)
        if "error" in fitted:
            logger.info("skip %s: %s", name, fitted["error"])
            continue
        _collect(fitted, name, tables, vifs, summaries)
        logger.info(
            "\n=== %s ===\nrows fitted %s (cases %s, controls %s); "
            "discarded before z-scoring %s; informative strata %s\n%s",
            name,
            fitted["n_rows_zscored_and_fitted"],
            fitted["n_cases"],
            fitted["n_controls"],
            fitted["n_rows_discarded_before_zscoring"],
            fitted["n_informative_strata"],
            _rounded(fitted["table"]),
        )
    return tables, vifs, summaries


def _specifications(features: pd.DataFrame) -> list[tuple[str, str, list]]:
    present = set(features["length_label"])
    labels = LAG_LABELS + [x for x in LENGTH_LABELS if x != PRIMARY_LABEL]
    specifications = [
        (label, f"{label}_imu", IMU_FEATURES)
        for label in labels
        if label in present
    ]
    specifications.append(
        (PRIMARY_LABEL, MAGNITUDE_SD_SPECIFICATION, IMU_FEATURES_MAGNITUDE_SD)
    )
    return specifications


def _collect(
```

```python
    fitted: dict,
    name: str,
    tables: list[pd.DataFrame],
    vifs: list[pd.DataFrame],
    summaries: dict,
) -> None:
    tables.append(fitted["table"])
    vifs.append(fitted["vif"])
    summaries[name] = {
        key: value
        for key, value in fitted.items()
        if key not in ("table", "vif")
    }


def _rounded(table: pd.DataFrame) -> str:
    return (
        table.drop(columns=["specification"]).round(4).to_string(index=False)
    )


def fit_tof_complete_case(
    primary_frame: pd.DataFrame,
    tables: list[pd.DataFrame],
    vifs: list[pd.DataFrame],
    summaries: dict,
) -> None:
    complete = primary_frame[
        primary_frame["tof_coverage_frac"].fillna(0) >= COVERAGE_MIN
    ].copy()
    excluded = sorted(set(primary_frame["va_id"]) - set(complete["va_id"]))
    fitted = fit_with_diagnostics(
        complete, IMU_FEATURES + TOF_FEATURES, TOF_SPECIFICATION
    )
    if "error" in fitted:
        return
    _collect(fitted, TOF_SPECIFICATION, tables, vifs, summaries)
    summaries[TOF_SPECIFICATION]["participants_excluded_no_tof"] = [
        int(participant) for participant in excluded
    ]
    fitted["table"].to_csv(
        TABLES_DIR / "table_clr_imu_tof_complete_case.csv", index=False
    )
    logger.info(
        "\n=== IMU + ToF, complete case (participants excluded: %s) ===\n%s",
        excluded,
        _rounded(fitted["table"]),
    )


def write_specification_tables(
    tables: list[pd.DataFrame], vifs: list[pd.DataFrame]
) -> pd.DataFrame:
    combined = pd.concat(tables, ignore_index=True)
    combined.to_csv(
        TABLES_DIR / "table_clr_all_specifications.csv", index=False
    )
    pd.concat(vifs, ignore_index=True).to_csv(
        TABLES_DIR / "table_clr_vif_all_specifications.csv", index=False
    )
    primary_table = combined[combined.specification == "primary_5min_imu"]
    primary_table.to_csv(TABLES_DIR / "table_clr_primary.csv", index=False)
    length_names = [
        f"{label}_imu" for label in LENGTH_LABELS if label != PRIMARY_LABEL
    ]
    combined[combined.specification.isin(length_names)].to_csv(
        TABLES_DIR / "table_clr_sensitivity_lengths.csv", index=False
    )
    combined[combined.specification.str.startswith("lag")].to_csv(
        TABLES_DIR / "table_clr_lag_analysis.csv", index=False
    )
    return primary_table


def write_standardisation_comparison(
    primary_frame: pd.DataFrame, primary_table: pd.DataFrame
```

```python
) -> None:
    all_rows = standardised_over_all_rows(primary_frame, IMU_FEATURES)
    analysis_set = {
        row["feature"]: float(row["or"]) for _, row in primary_table.iterrows()
    }
    rows = [
        {
            "feature": feature,
            "or_standardised_all_rows": round(all_rows[feature], 4),
            "or_standardised_analysis_set": round(analysis_set[feature], 4),
            "ratio": round(all_rows[feature] / analysis_set[feature], 4),
        }
        for feature in IMU_FEATURES
        if feature in all_rows and feature in analysis_set
    ]
    comparison = pd.DataFrame(rows)
    comparison.to_csv(
        TABLES_DIR / "table_zscoring_population_effect.csv", index=False
    )
    logger.info(
        "\n=== odds ratios by standardisation population ===\n%s",
        comparison.to_string(),
    )


def standardised_over_all_rows(
    frame: pd.DataFrame, columns: list[str]
) -> dict[str, float]:
    complete = frame.dropna(subset=[*columns, "is_case", "va_id"]).copy()
    standardised = zscore(complete, columns)
    spread = complete.groupby("va_id")["is_case"].std()
    informative = set(spread[spread > 0].index)
    standardised = standardised[standardised["va_id"].isin(informative)]
    result = fit_quietly(standardised, columns, max_iterations=200)
    return {
        feature: float(np.exp(coefficient))
        for feature, coefficient in zip(columns, result.params, strict=True)
    }


def write_collinearity_screen(primary_frame: pd.DataFrame) -> None:
    columns = [c for c in SCREEN_FEATURES if c in primary_frame.columns]
    correlation = primary_frame[columns].dropna().corr()
    rows = [
        {
            "feature_a": first,
            "feature_b": second,
            "pearson_r": round(float(correlation.loc[first, second]), 4),
        }
        for index, first in enumerate(columns)
        for second in columns[index + 1 :]
    ]
    screen = pd.DataFrame(rows).sort_values(
        "pearson_r", key=abs, ascending=False
    )
    screen.to_csv(TABLES_DIR / "table_collinearity_screen.csv", index=False)
    logger.info(
        "\n=== post-hoc collinearity screen (top pairs) ===\n%s",
        screen.head(10).to_string(index=False),
    )


if __name__ == "__main__":
    main()
```

14 src/jerk_time_base_comparison.py

```python
from __future__ import annotations

import logging

import pandas as pd

```

```python
from conditional_logit import fit_with_diagnostics, informative_participants
from config import (
    IMU_FEATURES,
    PRIMARY_LABEL,
    TABLES_DIR,
    configure_logging,
    ensure_output_dirs,
)

OBSERVED_JERK = "jerk_max_observed_interval"
UNIFORM_JERK = "jerk_max"
OBSERVED_JERK_FEATURES = [
    OBSERVED_JERK if feature == UNIFORM_JERK else feature
    for feature in IMU_FEATURES
]
SPECIFICATION = "primary_5min_imu_observed_interval_jerk"
INFLATION_THRESHOLD = 2.0

logger = logging.getLogger(__name__)


def main() -> None:
    configure_logging()
    ensure_output_dirs()
    candidates = pd.read_csv(TABLES_DIR / "table_features_candidate.csv")
    final = pd.read_csv(TABLES_DIR / "table_features_final.csv")
    primary = final[final.length_label == PRIMARY_LABEL]
    comparison = pd.DataFrame(
        coverage_rows(candidates[candidates.length_label == PRIMARY_LABEL])
        + ratio_rows("conditional-model analysis set", analysis_set(primary))
        + ratio_rows("all accepted primary windows", primary)
    )
    comparison.to_csv(
        TABLES_DIR / "table_jerk_time_base_comparison.csv", index=False
    )
    fitted = fit_with_diagnostics(
        primary, OBSERVED_JERK_FEATURES, SPECIFICATION
    )
    fitted["table"].to_csv(
        TABLES_DIR / "table_clr_observed_interval_jerk.csv", index=False
    )
    logger.info(
        "%s\n%s",
        comparison.to_string(index=False),
        fitted["table"].round(4).to_string(index=False),
    )


def coverage_rows(primary_candidates: pd.DataFrame) -> list[dict]:
    coverage = primary_candidates["imu_coverage_frac"]
    population = "primary candidate windows"
    return [
        _row(population, "n_windows", len(coverage)),
        _row(population, "n_coverage_above_1", int((coverage > 1).sum())),
        _row(
            population, "frac_coverage_above_1", float((coverage > 1).mean())
        ),
        _row(population, "max_coverage_fraction", float(coverage.max())),
    ]


def analysis_set(primary: pd.DataFrame) -> pd.DataFrame:
    complete = primary.dropna(
        subset=[*IMU_FEATURES, OBSERVED_JERK, "is_case", "va_id"]
    )
    return complete[complete["va_id"].isin(informative_participants(complete))]


def ratio_rows(population: str, windows: pd.DataFrame) -> list[dict]:
    both = windows.dropna(subset=[OBSERVED_JERK, UNIFORM_JERK])
    ratio = both[OBSERVED_JERK] / both[UNIFORM_JERK]
    return [
        _row(population, "n_windows", len(ratio)),
        _row(population, "median_ratio", float(ratio.median())),
        _row(
            population,
```

```
            "frac_ratio_above_2",
            float((ratio > INFLATION_THRESHOLD).mean()),
        ),
        _row(population, "max_ratio", float(ratio.max())),
        _row(population, "frac_ratio_above_1", float((ratio > 1).mean())),
    ]


def _row(population: str, quantity: str, value: float) -> dict:
    return {"population": population, "quantity": quantity, "value": value}


if __name__ == "__main__":
    main()
```

15 src/leave_one_participant_out.py

```
from __future__ import annotations

import json
import logging

import matplotlib.pyplot as plt
import numpy as np
import pandas as pd

import plotting
from conditional_logit import fit_with_diagnostics
from config import (
    FEATURE_LABELS,
    FIGURES_DIR,
    IMU_FEATURES,
    METRICS_DIR,
    PRIMARY_LABEL,
    TABLES_DIR,
    configure_logging,
    ensure_output_dirs,
)

FULL_MODEL = "none (full model)"
DOMINANT_PARTICIPANTS = ["va_6", "va_11", "va_14"]
SIGNIFICANCE_LEVEL = 0.05
SUMMARY_NOTE = (
    "Every case-contributing participant is removed in turn. Restricting the "
    "check to the three dominant contributors would understate the "
    "instability, because removing a participant with few events can also "
    "change a feature's significance."
)

logger = logging.getLogger(__name__)


def main() -> None:
    configure_logging()
    ensure_output_dirs()
    features = pd.read_csv(TABLES_DIR / "table_features_final.csv")
    primary = features[features.length_label == PRIMARY_LABEL].copy()
    full, refits = refit_without_each_participant(primary)
    combined = pd.concat([full, refits], ignore_index=True)
    combined.to_csv(TABLES_DIR / "table_lopo_sensitivity.csv", index=False)
    stability = summarise_stability(full, refits)
    stability.to_csv(
        TABLES_DIR / "table_lopo_stability_summary.csv", index=False
    )
    write_summary(primary, stability)
    plot_forest(combined)
    logger.info("wrote table_lopo_sensitivity.csv and fig4_lopo_forest")


def refit_without_each_participant(
    primary: pd.DataFrame,
) -> tuple[pd.DataFrame, pd.DataFrame]:
    fitted = fit_with_diagnostics(primary, IMU_FEATURES, "full_model")
```

```
    full = fitted["table"].copy()
    full["dropped_participant"] = FULL_MODEL
    full = full.assign(
        n_falls_dropped=0,
        n_informative_strata_remaining=fitted["n_informative_strata"],
    )
    cases = primary[primary.is_case == 1]
    falls_by_participant = cases.groupby("va_id").size().to_dict()
    participants = sorted(cases["va_id"].unique())
    logger.info("case-contributing participants: %s", participants)
    refits = []
    for participant in participants:
        refit = fit_with_diagnostics(
            primary[primary.va_id != participant],
            IMU_FEATURES,
            f"drop_va_{participant}",
        )
        table = refit["table"].copy()
        table["dropped_participant"] = f"va_{participant}"
        table["n_falls_dropped"] = falls_by_participant.get(participant, 0)
        table["n_informative_strata_remaining"] = refit["n_informative_strata"]
        refits.append(table)
        logger.info(
            "\n-- drop va_%s (%s events) -- remaining strata: %s\n%s",
            participant,
            falls_by_participant.get(participant, 0),
            refit["n_informative_strata"],
            table[["feature", "or", "or_ci_lo", "or_ci_hi", "p_value"]]
            .round(4)
            .to_string(index=False),
        )
    return full, pd.concat(refits, ignore_index=True)


def summarise_stability(
    full: pd.DataFrame, refits: pd.DataFrame
) -> pd.DataFrame:
    removed = list(dict.fromkeys(refits["dropped_participant"]))
    rows = [
        _feature_stability(feature, full, refits, removed)
        for feature in IMU_FEATURES
    ]
    return pd.DataFrame(rows).sort_values(
        "max_log_or_shift_any_participant", ascending=False
    )


def _feature_stability(
    feature: str, full: pd.DataFrame, refits: pd.DataFrame, removed: list
) -> dict:
    base = full.loc[full.feature == feature].iloc[0]
    base_significant = base["p_value"] < SIGNIFICANCE_LEVEL
    shift_dominant = shift_any = 0.0
    changes_dominant: list[str] = []
    changes_any: list[str] = []
    for participant in removed:
        match = refits[
            (refits.feature == feature)
            & (refits.dropped_participant == participant)
        ]
        if match.empty:
            continue
        row = match.iloc[0]
        shift = abs(np.log(row["or"]) - np.log(base["or"]))
        shift_any = max(shift_any, shift)
        changed = (row["p_value"] < SIGNIFICANCE_LEVEL) != base_significant
        if changed:
            changes_any.append(f"{participant}({int(row['n_falls_dropped'])})")
        if participant in DOMINANT_PARTICIPANTS:
            shift_dominant = max(shift_dominant, shift)
            if changed:
                changes_dominant.append(participant)
    return {
        "feature": feature,
        "full_or": base["or"],
        "full_p": base["p_value"],
```

```python
        "max_log_or_shift_focus3": shift_dominant,
        "significance_flips_when_dropping": ";".join(changes_dominant),
        "max_log_or_shift_any_participant": shift_any,
        "significance_flips_any_participant": ";".join(changes_any),
        "n_participants_whose_removal_flips": len(changes_any),
    }


def write_summary(primary: pd.DataFrame, stability: pd.DataFrame) -> None:
    dominant = stability["significance_flips_when_dropping"] != ""
    any_removal = stability["significance_flips_any_participant"] != ""
    summary = {
        "n_case_participants": len(
            primary.loc[primary.is_case == 1, "va_id"].unique()
        ),
        "n_features_with_significance_flip_on_focus3": int(dominant.sum()),
        "features_with_flip_focus3": stability.loc[
            dominant, "feature"
        ].tolist(),
        "n_features_with_significance_flip_any_participant": int(
            any_removal.sum()
        ),
        "features_with_flip_any_participant": stability.loc[
            any_removal, "feature"
        ].tolist(),
        "flip_detail_any_participant": {
            row["feature"]: row["significance_flips_any_participant"]
            for _, row in stability.iterrows()
            if row["significance_flips_any_participant"]
        },
        "note": SUMMARY_NOTE,
    }
    (METRICS_DIR / "lopo_summary.json").write_text(
        json.dumps(summary, indent=2)
    )
    logger.info(
        "%s of %s features change significance when dropping va_6, va_11 "
        "or va_14.",
        int(dominant.sum()),
        len(IMU_FEATURES),
    )


def plot_forest(combined: pd.DataFrame) -> None:
    plotting.apply_style()
    shown = [FULL_MODEL, *DOMINANT_PARTICIPANTS]
    subset = combined[combined.dropped_participant.isin(shown)]
    n_rows = len(IMU_FEATURES)
    fig, ax = plt.subplots(figsize=plotting.forest_figsize(n_rows))
    positions = np.arange(n_rows)
    offsets = np.linspace(-0.3, 0.3, len(shown))
    for index, dropped in enumerate(shown):
        estimates = (
            subset[subset.dropped_participant == dropped]
            .set_index("feature")
            .reindex(IMU_FEATURES)
        )
        ax.errorbar(
            estimates["or"],
            positions + offsets[index],
            xerr=[
                estimates["or"] - estimates["or_ci_lo"],
                estimates["or_ci_hi"] - estimates["or"],
            ],
            fmt="o",
            color=plotting.series(index)["color"],
            label=_legend_label(dropped),
            capsize=2,
            markersize=4,
        )
    ax.axvline(
        1.0, color=plotting.PALETTE["grey"], linestyle="--", linewidth=1
    )
    ax.set_yticks(positions)
    ax.set_yticklabels([FEATURE_LABELS[name] for name in IMU_FEATURES])
    ax.set_xlabel("odds ratio (95% CI), per-SD, conditional logistic model")
```

```python
    ax.legend(loc="upper right", fontsize=7)
    plotting.adjust_forest(fig)
    plotting.save(fig, FIGURES_DIR, "fig4_lopo_forest", tight=False)
    plt.close(fig)


def _legend_label(dropped: str) -> str:
    if dropped.startswith("none"):
        return "Full model"
    return f"Without {dropped}"


if __name__ == "__main__":
    main()
```

16 src/permutation_control.py

```python
from __future__ import annotations

import json
import logging
import time

import numpy as np
import pandas as pd
from scipy.special import gammaln

from conditional_logit import fit_quietly, zscore
from config import (
    IMU_FEATURES,
    METRICS_DIR,
    PRIMARY_LABEL,
    SEED,
    TABLES_DIR,
    configure_logging,
    ensure_output_dirs,
)

PERMUTATIONS = 500
PROGRESS_EVERY = 50
NULL_PERCENTILE = 95

logger = logging.getLogger(__name__)


def main() -> None:
    configure_logging()
    ensure_output_dirs()
    features = pd.read_csv(TABLES_DIR / "table_features_final.csv")
    primary = (
        features[features.length_label == PRIMARY_LABEL]
        .dropna(subset=[*IMU_FEATURES, "is_case", "va_id"])
        .reset_index(drop=True)
    )
    null_log_likelihood = null_llf(primary)
    observed = 2 * (fit_llf(primary, IMU_FEATURES) - null_log_likelihood)
    logger.info("observed LR statistic: %.4f", observed)
    permuted = permutation_statistics(primary, null_log_likelihood)
    write_permutation_results(observed, permuted)
    necessity = leave_one_feature_out(
        primary, null_log_likelihood, observed, permuted
    )
    necessity.to_csv(TABLES_DIR / "table_lofo_carries_check.csv", index=False)
    logger.info(
        "\n=== leave-one-feature-out ===\n%s",
        necessity.round(4).to_string(index=False),
    )


def null_llf(frame: pd.DataFrame) -> float:
    counts = frame.groupby("va_id")["is_case"].agg(["sum", "count"])
    counts = counts[(counts["sum"] > 0) & (counts["sum"] < counts["count"])]
    return float(
```

```python
        -np.sum(
            _log_binomial(counts["count"].to_numpy(), counts["sum"].to_numpy())
        )
    )


def _log_binomial(n: np.ndarray, k: np.ndarray) -> np.ndarray:
    return gammaln(n + 1) - gammaln(k + 1) - gammaln(n - k + 1)


def fit_llf(frame: pd.DataFrame, columns: list[str]) -> float | None:
    standardised = zscore(frame, columns)
    try:
        result = fit_quietly(standardised, columns, max_iterations=200)
    except (np.linalg.LinAlgError, ValueError):
        return None
    return float(result.llf)


def permutation_statistics(
    frame: pd.DataFrame, null_log_likelihood: float
) -> np.ndarray:
    random_generator = np.random.default_rng(SEED)
    start = time.time()
    statistics = []
    for index in range(PERMUTATIONS):
        permuted = permute_within_strata(frame, random_generator)
        log_likelihood = fit_llf(permuted, IMU_FEATURES)
        if log_likelihood is None:
            continue
        statistics.append(2 * (log_likelihood - null_log_likelihood))
        if (index + 1) % PROGRESS_EVERY == 0:
            logger.info(
                "  permutation %s/%s (%.1fs elapsed)",
                index + 1,
                PERMUTATIONS,
                time.time() - start,
            )
    return np.array(statistics)


def permute_within_strata(
    frame: pd.DataFrame, random_generator: np.random.Generator
) -> pd.DataFrame:
    permuted = frame.copy()
    labels = permuted["is_case"].to_numpy().copy()
    for _, rows in permuted.groupby("va_id").groups.items():
        rows = np.array(rows)
        stratum_labels = permuted.loc[rows, "is_case"].to_numpy().copy()
        random_generator.shuffle(stratum_labels)
        labels[permuted.index.get_indexer(rows)] = stratum_labels
    permuted["is_case"] = labels
    return permuted


def write_permutation_results(observed: float, permuted: np.ndarray) -> None:
    summary = {
        "n_perm_requested": PERMUTATIONS,
        "n_perm_completed": int(len(permuted)),
        "lr_observed": float(observed),
        "lr_perm_mean": float(permuted.mean()),
        "lr_perm_sd": float(permuted.std()),
        "lr_perm_max": float(permuted.max()),
        "p_value": float(
            (np.sum(permuted >= observed) + 1) / (len(permuted) + 1)
        ),
    }
    (METRICS_DIR / "permutation_test.json").write_text(
        json.dumps(summary, indent=2)
    )
    pd.DataFrame({"lr_perm": permuted}).to_csv(
        TABLES_DIR / "table_permutation_null_lr.csv", index=False
    )
    logger.info(
        "\n=== permutation test ===\n%s", json.dumps(summary, indent=2)
    )
```

```python


def leave_one_feature_out(
    frame: pd.DataFrame,
    null_log_likelihood: float,
    observed: float,
    permuted: np.ndarray,
) -> pd.DataFrame:
    threshold = np.percentile(permuted, NULL_PERCENTILE)
    rows = []
    for feature in IMU_FEATURES:
        remaining = [f for f in IMU_FEATURES if f != feature]
        statistic = 2 * (fit_llf(frame, remaining) - null_log_likelihood)
        rows.append(
            {
                "feature_dropped": feature,
                "n_features_remaining": len(remaining),
                "lr_statistic": statistic,
                "lr_full_model": observed,
                "lr_drop_frac_of_full": (
                    statistic / observed if observed else np.nan
                ),
                "within_perm_null_range": bool(statistic <= threshold),
            }
        )
    return pd.DataFrame(rows).sort_values("lr_statistic")


if __name__ == "__main__":
    main()
```

17 src/fit_boosted_model.py

```python
from __future__ import annotations

import json
import logging
from collections.abc import Callable

import matplotlib.pyplot as plt
import numpy as np
import pandas as pd
import xgboost as xgb
from scipy.stats import spearmanr
from sklearn.metrics import average_precision_score, roc_auc_score
from sklearn.model_selection import StratifiedGroupKFold

import plotting
from config import (
    FEATURE_LABELS,
    FIGURES_DIR,
    METRICS_DIR,
    PRIMARY_LABEL,
    SEED,
    TABLES_DIR,
    TOF_FEATURES,
    configure_logging,
    ensure_output_dirs,
)

OUTER_FOLDS = 5
FROZEN_BOOTSTRAP_REPLICATES = 1000
REFIT_BOOTSTRAP_REPLICATES = 200

BOOSTED_IMU_FEATURES = [
    "accel_mag_mean",
    "accel_dynamic_rms_gravity_removed",
    "jerk_mean",
    "jerk_max",
    "dom_freq_hz",
    "spectral_entropy",
    "gait_band_power_frac",
    "posture_transition_rate",
```

```
]
ALL_FEATURES = BOOSTED_IMU_FEATURES + TOF_FEATURES

logger = logging.getLogger(__name__)


def main() -> None:
    configure_logging()
    ensure_output_dirs()
    windows = load_primary_windows()
    features = windows[ALL_FEATURES].astype(float)
    labels = windows["is_case"].astype(int).to_numpy()
    groups = windows["va_id"].to_numpy()
    log_sample(windows, features, labels)
    positive_weight = (len(labels) - labels.sum()) / max(labels.sum(), 1)

    performance = evaluate(features, labels, groups, positive_weight)
    performance["n_rows"] = int(len(windows))
    write_performance(performance, labels, windows)

    shap_table = shap_importance(features, labels, positive_weight)
    shap_table.to_csv(
        TABLES_DIR / "table_gbm_shap_importance.csv", index=False
    )
    logger.info("SHAP mean(|value|) ranking:")
    logger.info(shap_table.round(4).to_string(index=False))
    compare_with_conditional_model(shap_table)
    draw_shap_figure(shap_table)


def load_primary_windows() -> pd.DataFrame:
    final = pd.read_csv(TABLES_DIR / "table_features_final.csv")
    return (
        final[final.length_label == PRIMARY_LABEL]
        .copy()
        .reset_index(drop=True)
    )


def log_sample(
    windows: pd.DataFrame, features: pd.DataFrame, labels: np.ndarray
) -> None:
    logger.info(
        "analytic table: %d rows, %d cases, %d non-cases, %d participants",
        len(windows),
        labels.sum(),
        len(windows) - labels.sum(),
        windows["va_id"].nunique(),
    )
    logger.info("missingness per feature (percent):")
    logger.info((features.isna().mean() * 100).round(1).to_string())


def evaluate(
    features: pd.DataFrame,
    labels: np.ndarray,
    groups: np.ndarray,
    positive_weight: float,
) -> dict:
    n_case_participants = int(len(np.unique(groups[labels == 1])))
    alternative_folds = max(2, min(OUTER_FOLDS, n_case_participants // 2))
    fold_options = [OUTER_FOLDS] + (
        [alternative_folds] if alternative_folds != OUTER_FOLDS else []
    )
    fold_frames, by_fold_count, out_of_fold = [], {}, {}
    for n_splits in fold_options:
        predictions, folds = run_grouped_cv(
            features, labels, groups, positive_weight, n_splits
        )
        fold_frames.append(folds)
        by_fold_count[n_splits] = _fold_count_summary(
            n_splits, folds, labels, predictions
        )
        out_of_fold[n_splits] = predictions
    pd.concat(fold_frames, ignore_index=True).to_csv(
        TABLES_DIR / "table_gbm_cv_folds.csv", index=False
```

```
    )
    performance = _pooled_performance(
        features, labels, groups, positive_weight, out_of_fold[OUTER_FOLDS]
    )
    performance["n_case_participants"] = n_case_participants
    performance["folds"] = fold_frames[0]
    performance["by_n_splits"] = by_fold_count
    return performance


def make_model(positive_weight: float) -> xgb.XGBClassifier:
    return xgb.XGBClassifier(
        n_estimators=200,
        max_depth=3,
        learning_rate=0.05,
        subsample=0.8,
        colsample_bytree=0.8,
        reg_lambda=1.0,
        min_child_weight=3,
        scale_pos_weight=positive_weight,
        eval_metric="aucpr",
        random_state=SEED,
        tree_method="hist",
        missing=np.nan,
    )


def run_grouped_cv(
    features: pd.DataFrame,
    labels: np.ndarray,
    groups: np.ndarray,
    positive_weight: float,
    n_splits: int,
    seed: int = SEED,
) -> tuple[np.ndarray, pd.DataFrame]:
    splitter = StratifiedGroupKFold(
        n_splits=n_splits, shuffle=True, random_state=seed
    )
    out_of_fold = np.full(len(labels), np.nan)
    rows = []
    for fold, (train, test) in enumerate(
        splitter.split(features, labels, groups)
    ):
        model = make_model(positive_weight)
        model.fit(features.iloc[train], labels[train])
        probabilities = model.predict_proba(features.iloc[test])[:, 1]
        out_of_fold[test] = probabilities
        rows.append(
            _fold_row(n_splits, fold, test, labels, groups, probabilities)
        )
    return out_of_fold, pd.DataFrame(rows)


def _fold_row(
    n_splits: int,
    fold: int,
    test: np.ndarray,
    labels: np.ndarray,
    groups: np.ndarray,
    probabilities: np.ndarray,
) -> dict:
    has_both = len(np.unique(labels[test])) > 1
    return {
        "n_splits": n_splits,
        "fold": fold,
        "n_test": len(test),
        "n_test_cases": int(labels[test].sum()),
        "n_test_participants": len(np.unique(groups[test])),
        "auroc": (
            roc_auc_score(labels[test], probabilities) if has_both else np.nan
        ),
        "auprc": (
            average_precision_score(labels[test], probabilities)
            if has_both
            else np.nan
        ),
```

```
        "evaluable": bool(has_both),
    }


def _fold_count_summary(
    n_splits: int,
    folds: pd.DataFrame,
    labels: np.ndarray,
    predictions: np.ndarray,
) -> dict:
    predicted = ~np.isnan(predictions)
    evaluable = folds[folds["evaluable"]]
    has_folds = len(evaluable) > 0
    return {
        "n_splits": n_splits,
        "n_folds_without_any_case": int((~folds["evaluable"]).sum()),
        "fold_auroc_min": (
            float(evaluable["auroc"].min()) if has_folds else None
        ),
        "fold_auroc_max": (
            float(evaluable["auroc"].max()) if has_folds else None
        ),
        "fold_auroc_median": (
            float(evaluable["auroc"].median()) if has_folds else None
        ),
        "pooled_auroc": float(
            roc_auc_score(labels[predicted], predictions[predicted])
        ),
        "pooled_auprc": float(
            average_precision_score(labels[predicted], predictions[predicted])
        ),
    }


def _pooled_performance(
    features: pd.DataFrame,
    labels: np.ndarray,
    groups: np.ndarray,
    positive_weight: float,
    out_of_fold: np.ndarray,
) -> dict:
    predicted = ~np.isnan(out_of_fold)
    observed, scores = labels[predicted], out_of_fold[predicted]
    kept_groups = groups[predicted]
    auroc_interval = cluster_bootstrap_ci(
        observed, scores, kept_groups, roc_auc_score
    )
    auprc_interval = cluster_bootstrap_ci(
        observed, scores, kept_groups, average_precision_score
    )
    logger.info("refitting cluster bootstrap (whole grouped CV per replicate)")
    refit_auroc, refit_auprc = refit_cluster_bootstrap(
        features, labels, groups, positive_weight, OUTER_FOLDS
    )
    return {
        "auroc": float(roc_auc_score(observed, scores)),
        "auprc": float(average_precision_score(observed, scores)),
        "auroc_frozen": auroc_interval,
        "auprc_frozen": auprc_interval,
        "auroc_refit": refit_auroc,
        "auprc_refit": refit_auprc,
    }


def cluster_bootstrap_ci(
    labels: np.ndarray,
    scores: np.ndarray,
    groups: np.ndarray,
    metric: Callable,
    n_boot: int = FROZEN_BOOTSTRAP_REPLICATES,
    seed: int = SEED,
) -> tuple[float, float, int]:
    random_generator = np.random.default_rng(seed)
    participants = np.unique(groups)
    values = []
    for _ in range(n_boot):
```

```python
        sampled = random_generator.choice(
            participants, size=len(participants), replace=True
        )
        rows = np.concatenate([np.where(groups == g)[0] for g in sampled])
        if len(np.unique(labels[rows])) < 2:
            continue
        values.append(metric(labels[rows], scores[rows]))
    low, high = np.percentile(values, [2.5, 97.5])
    return float(low), float(high), int(len(values))


def refit_cluster_bootstrap(
    features: pd.DataFrame,
    labels: np.ndarray,
    groups: np.ndarray,
    positive_weight: float,
    n_splits: int,
    n_boot: int = REFIT_BOOTSTRAP_REPLICATES,
    seed: int = SEED,
) -> tuple[tuple, tuple]:
    random_generator = np.random.default_rng(seed)
    participants = np.unique(groups)
    aurocs: list[float] = []
    auprcs: list[float] = []
    for _ in range(n_boot):
        sampled = random_generator.choice(
            participants, size=len(participants), replace=True
        )
        rows = np.concatenate([np.where(groups == g)[0] for g in sampled])
        replicate_groups, replicate_labels = groups[rows], labels[rows]
        if (
            len(np.unique(replicate_labels)) < 2
            or len(np.unique(replicate_groups[replicate_labels == 1])) < 2
        ):
            continue
        try:
            metrics = _refit_replicate(
                features.iloc[rows],
                replicate_labels,
                replicate_groups,
                positive_weight,
                n_splits,
                int(random_generator.integers(1 << 31)),
            )
        except ValueError:
            continue
        if metrics is not None:
            aurocs.append(metrics[0])
            auprcs.append(metrics[1])
    return _percentile_summary(aurocs), _percentile_summary(auprcs)


def _refit_replicate(
    features: pd.DataFrame,
    labels: np.ndarray,
    groups: np.ndarray,
    positive_weight: float,
    n_splits: int,
    split_seed: int,
) -> tuple[float, float] | None:
    out_of_fold = np.full(len(labels), np.nan)
    splitter = StratifiedGroupKFold(
        n_splits=n_splits, shuffle=True, random_state=split_seed
    )
    for train, test in splitter.split(features, labels, groups):
        if len(np.unique(labels[train])) < 2:
            continue
        model = make_model(positive_weight)
        model.fit(features.iloc[train], labels[train])
        out_of_fold[test] = model.predict_proba(features.iloc[test])[:, 1]
    predicted = ~np.isnan(out_of_fold)
    if not predicted.sum() or len(np.unique(labels[predicted])) <= 1:
        return None
    return (
        roc_auc_score(labels[predicted], out_of_fold[predicted]),
        average_precision_score(labels[predicted], out_of_fold[predicted]),
```

```python
    )


def _percentile_summary(values: list[float]) -> tuple:
    return (
        float(np.percentile(values, 2.5)),
        float(np.percentile(values, 97.5)),
        float(np.median(values)),
        int(len(values)),
    )


def write_performance(
    performance: dict, labels: np.ndarray, windows: pd.DataFrame
) -> None:
    folds = performance["folds"]
    evaluable = folds[folds["evaluable"]]
    auroc_low, auroc_high, n_auroc = performance["auroc_frozen"]
    auprc_low, auprc_high, n_auprc = performance["auprc_frozen"]
    refit_low, refit_high, refit_median, n_refit = performance["auroc_refit"]
    refit_auprc_low, refit_auprc_high = performance["auprc_refit"][:2]
    summary = {
        "n_rows": performance["n_rows"],
        "n_cases": int(labels.sum()),
        "n_controls": int(len(labels) - labels.sum()),
        "n_participants": int(windows["va_id"].nunique()),
        "n_case_participants": performance["n_case_participants"],
        "auroc": performance["auroc"],
        "auprc": performance["auprc"],
        "prevalence": float(labels.mean()),
        "auroc_ci_refit_lo": refit_low,
        "auroc_ci_refit_hi": refit_high,
        "auroc_refit_median": refit_median,
        "n_refit_replicates": n_refit,
        "auprc_ci_refit_lo": refit_auprc_low,
        "auprc_ci_refit_hi": refit_auprc_high,
        "reported_interval": "auroc_ci_refit_lo/hi",
        "reported_interval_note": (
            "Participant-level bootstrap in which the entire grouped "
            "cross-validation is repeated inside every replicate, so the "
            "interval includes model-fitting and fold-assignment variability."
        ),
        "auroc_ci_frozen_lo": auroc_low,
        "auroc_ci_frozen_hi": auroc_high,
        "auprc_ci_frozen_lo": auprc_low,
        "auprc_ci_frozen_hi": auprc_high,
        "n_cluster_boot_replicates_auroc": n_auroc,
        "n_cluster_boot_replicates_auprc": n_auprc,
        "frozen_interval_note": (
            "Resamples participants from a fixed vector of out-of-fold "
            "predictions without refitting. It excludes model-fitting "
            "variability, so it can be narrower than the spread of the fold "
            "estimates."
        ),
        "fold_auroc_min": float(evaluable["auroc"].min()),
        "fold_auroc_max": float(evaluable["auroc"].max()),
        "n_folds_without_any_case": int((~folds["evaluable"]).sum()),
        "fold_note": (
            "With this many case-contributing participants a "
            "participant-grouped split cannot guarantee a case in every "
            "fold; folds without one are undefined and contribute nothing."
        ),
        "by_n_splits": performance["by_n_splits"],
        "independence_note": (
            "This model uses the same recordings, features and labels as the "
            "conditional model, so it is a descriptive check on feature "
            "ranking, not independent confirmation. Any timing or "
            "feature-construction artifact passes through it unchanged."
        ),
    }
    (METRICS_DIR / "gbm_performance.json").write_text(
        json.dumps(summary, indent=2)
    )
    logger.info(json.dumps(summary, indent=2))

```

```python
def shap_importance(
    features: pd.DataFrame, labels: np.ndarray, positive_weight: float
) -> pd.DataFrame:
    final_model = make_model(positive_weight)
    final_model.fit(features, labels)
    contributions = tree_shap_values(final_model, features)
    table = pd.DataFrame(
        {
            "feature": ALL_FEATURES,
            "mean_abs_shap": np.abs(contributions).mean(axis=0),
        }
    )
    return table.sort_values("mean_abs_shap", ascending=False).reset_index(
        drop=True
    )


def tree_shap_values(
    model: xgb.XGBClassifier, features: pd.DataFrame
) -> np.ndarray:
    contributions = model.get_booster().predict(
        xgb.DMatrix(features, feature_names=list(ALL_FEATURES)),
        pred_contribs=True,
    )
    if contributions.shape[1] != len(ALL_FEATURES) + 1:
        raise ValueError(
            f"pred_contribs returned {contributions.shape[1]} columns, "
            f"expected {len(ALL_FEATURES) + 1} (features plus bias)"
        )
    return contributions[:, :-1]


def compare_with_conditional_model(shap_table: pd.DataFrame) -> None:
    conditional = pd.read_csv(TABLES_DIR / "table_clr_primary.csv")
    merged = shap_table.merge(
        conditional[["feature", "z", "or"]], on="feature", how="inner"
    )
    merged["shap_rank"] = merged["mean_abs_shap"].rank(ascending=False)
    merged["clr_rank"] = merged["z"].abs().rank(ascending=False)
    rho, rho_p = spearmanr(merged["shap_rank"], merged["clr_rank"])
    merged.to_csv(TABLES_DIR / "table_gbm_vs_clr_ranking.csv", index=False)
    boosted_only = sorted(
        set(shap_table["feature"]) - set(conditional["feature"])
    )
    logger.info(
        "Spearman rank correlation over %d shared features: rho=%.3f, p=%.4f",
        len(merged),
        rho,
        rho_p,
    )
    (METRICS_DIR / "gbm_vs_clr_ranking.json").write_text(
        json.dumps(
            {
                "spearman_rho": float(rho),
                "spearman_p": float(rho_p),
                "n_features_compared": int(len(merged)),
                "features_in_gbm_only": boosted_only,
            },
            indent=2,
        )
    )


def draw_shap_figure(shap_table: pd.DataFrame) -> None:
    plotting.apply_style()
    fig, ax = plt.subplots(figsize=(plotting.WIDTH_FULL, 4.0))
    order = shap_table.sort_values("mean_abs_shap")
    ax.barh(
        [FEATURE_LABELS[name] for name in order["feature"]],
        order["mean_abs_shap"],
        color=plotting.PALETTE["blue"],
        label="mean |SHAP value|",
    )
    ax.set_xlabel("mean |SHAP value| (log-odds contribution)")
    ax.set_ylim(-0.6, len(order) + 0.4)
    fig.tight_layout()
```

```python
    plotting.save(fig, FIGURES_DIR, "fig5_gbm_shap_importance")
    plt.close(fig)


if __name__ == "__main__":
    main()
```

18 src/referent_and_activity_analyses.py

```python
from __future__ import annotations

import json
import logging

import numpy as np
import pandas as pd
from scipy import stats

from conditional_logit import fit_specification, informative_participants
from config import (
    COVERAGE_MIN,
    IMU_FEATURES,
    LAG_LABELS,
    LAG_SECONDS,
    LENGTH_LABELS,
    METRICS_DIR,
    MOVEMENT_FEATURE,
    PRIMARY_LABEL,
    PROCESSED_DIR,
    SECONDS_PER_DAY,
    TABLES_DIR,
    TARGET_CONTROLS_PER_CASE,
    configure_logging,
)

IMU_WITHOUT_POSTURE = [
    feature for feature in IMU_FEATURES if feature != "posture_transition_rate"
]
PRIMARY_WINDOW_SECONDS = 300
DOCUMENTED_ID_RANGE = (5, 33)
MIN_DAYS_AFTER_FALL = 7

DST_TRANSITIONS_UTC = np.array(
    [
        pd.Timestamp(moment, tz="UTC").timestamp()
        for moment in (
            "2016-03-13 10:00",
            "2016-11-06 09:00",
            "2017-03-12 10:00",
            "2017-11-05 09:00",
            "2018-03-11 10:00",
            "2018-11-04 09:00",
        )
    ]
)

logger = logging.getLogger(__name__)


def main() -> None:
    configure_logging()
    final = pd.read_csv(TABLES_DIR / "table_features_final.csv")
    candidate = pd.read_csv(TABLES_DIR / "table_features_candidate.csv")
    manifest = pd.read_csv(TABLES_DIR / "table_cohort_manifest.csv")
    calibration = pd.read_csv(TABLES_DIR / "table_calibration_audit.csv")
    falls = pd.read_csv(PROCESSED_DIR / "falls_deduped.csv")

    balance, prefall, referent_counts, per_participant = referent_day_tables(
        final
    )
    activity = activity_contrasts(final, candidate)
    lag_activity = lag_within_activity(final)
    capture, capture_summary = fall_capture(candidate)
```

```python
    identifiers, identifier_summary = archive_identifier_coverage(manifest)
    characteristics = participant_characteristics(
        final, manifest, candidate, calibration
    )
    timing = referent_timing(final, falls)
    marker = ambulatory_marker_by_arm(final)

    outputs = {
        "table_referent_day_balance.csv": balance,
        "table_clr_prefall_referents.csv": prefall,
        "table_referent_days_per_participant.csv": per_participant,
        "table_clr_activity_contrast.csv": activity,
        "table_clr_lag_within_activity.csv": lag_activity,
        "table_fall_capture_selection.csv": capture,
        "table_archive_id_coverage.csv": identifiers,
        "table_participant_characteristics.csv": characteristics,
        "table_clr_referent_timing_sensitivity.csv": timing,
        "table_ambulatory_marker_by_arm.csv": marker,
    }
    for name, table in outputs.items():
        table.to_csv(TABLES_DIR / name, index=False)

    summary = build_summary(
        referent_counts,
        balance,
        prefall,
        activity,
        lag_activity,
        timing,
        capture_summary,
        identifier_summary,
    )
    (METRICS_DIR / "referent_activity_summary.json").write_text(
        json.dumps(summary, indent=2), encoding="utf-8"
    )
    log_summary(summary, characteristics, lag_activity)


def ambulatory(windows: pd.DataFrame) -> pd.Series:
    return (windows["posture_transition_rate"] > 0).astype(float)


def ambulatory_marker_by_arm(final: pd.DataFrame) -> pd.DataFrame:
    primary = final[final.length_label == PRIMARY_LABEL]
    analysis = primary[
        primary["va_id"].isin(informative_participants(primary))
    ]
    rows = []
    for arm, is_case in [("case windows", 1), ("referent windows", 0)]:
        windows = analysis[analysis.is_case == is_case]
        moving = ambulatory(windows)
        rows.append(
            {
                "arm": arm,
                "n_windows": int(len(windows)),
                "n_ambulatory": int(moving.sum()),
                "frac_ambulatory": float(moving.mean()),
            }
        )
    return pd.DataFrame(rows)


def referent_day_tables(
    final: pd.DataFrame,
) -> tuple[pd.DataFrame, pd.DataFrame, dict, pd.DataFrame]:
    primary = final[final.length_label == PRIMARY_LABEL].copy()
    analysis = primary[
        primary["va_id"].isin(informative_participants(primary))
    ].copy()
    referents = analysis[analysis.is_case == 0].copy()
    referents["day_side"] = np.where(
        referents.shift_days < 0, "pre-fall", "post-fall"
    )
    balance = _referent_balance(referents)
    per_participant = _referents_per_participant(referents, analysis)

```

```python
    fits = []
    for label, subset in [
        ("primary (all referent days)", primary),
        (
            "pre-fall referent days only",
            primary[(primary.is_case == 1) | (primary.shift_days < 0)],
        ),
        (
            "post-fall referent days only",
            primary[(primary.is_case == 1) | (primary.shift_days > 0)],
        ),
    ]:
        result = fit_specification(subset, IMU_FEATURES, label)
        if "table" in result:
            fits.append(result["table"])
    counts = {
        "n_referents_pre_fall": int((referents.day_side == "pre-fall").sum()),
        "n_referents_post_fall": int(
            (referents.day_side == "post-fall").sum()
        ),
        "n_participants_with_no_pre_fall_referent": int(
            (per_participant.n_referents_pre_fall == 0).sum()
        ),
    }
    return balance, pd.concat(fits, ignore_index=True), counts, per_participant


def _referent_balance(referents: pd.DataFrame) -> pd.DataFrame:
    rows = []
    for column in [
        MOVEMENT_FEATURE,
        "gait_band_power_frac",
        "jerk_max",
        "posture_transition_rate",
    ]:
        by_participant = referents.groupby("va_id")[column]
        within = (
            referents[column] - by_participant.transform("mean")
        ) / by_participant.transform("std")
        before = within[referents.day_side == "pre-fall"].dropna()
        after = within[referents.day_side == "post-fall"].dropna()
        test = stats.mannwhitneyu(after, before)
        rows.append(
            {
                "feature": column,
                "n_pre_fall_referents": int(len(before)),
                "n_post_fall_referents": int(len(after)),
                "mean_within_participant_z_pre": before.mean(),
                "mean_within_participant_z_post": after.mean(),
                "mann_whitney_p": test.pvalue,
            }
        )
    return pd.DataFrame(rows)


def _referents_per_participant(
    referents: pd.DataFrame, analysis: pd.DataFrame
) -> pd.DataFrame:
    counts = (
        referents.groupby(["va_id", "day_side"])
        .size()
        .unstack(fill_value=0)
        .rename(
            columns={
                "pre-fall": "n_referents_pre_fall",
                "post-fall": "n_referents_post_fall",
            }
        )
        .reset_index()
    )
    cases = (
        analysis[analysis.is_case == 1]
        .groupby("va_id")
        .size()
        .rename("n_cases")
    )
```

```python
    return counts.merge(cases, on="va_id", how="left")


def activity_contrasts(
    final: pd.DataFrame, candidate: pd.DataFrame
) -> pd.DataFrame:
    primary = final[final.length_label == PRIMARY_LABEL].copy()
    primary["ambulatory"] = ambulatory(primary)
    moving = primary[primary.ambulatory == 1]
    specifications = [
        ("primary", primary, IMU_FEATURES),
        ("ambulation indicator alone", primary, ["ambulatory"]),
        ("movement term alone", primary, [MOVEMENT_FEATURE]),
        (
            "movement term + ambulation indicator",
            primary,
            [MOVEMENT_FEATURE, "ambulatory"],
        ),
        (
            "full model, ambulation indicator for posture rate",
            primary,
            [*IMU_WITHOUT_POSTURE, "ambulatory"],
        ),
        (
            "restricted to ambulatory windows, both arms",
            moving,
            IMU_WITHOUT_POSTURE,
        ),
        (
            "restricted to ambulatory windows, movement term alone",
            moving,
            [MOVEMENT_FEATURE],
        ),
    ]
    walking_set = walking_referent_set(primary, candidate)
    specifications += [
        ("walking referents, full model", walking_set, IMU_FEATURES),
        (
            "walking referents, movement term alone",
            walking_set,
            [MOVEMENT_FEATURE],
        ),
    ]
    fits = []
    for label, subset, columns in specifications:
        result = fit_specification(subset, columns, label)
        if "table" in result:
            fits.append(result["table"])
    return pd.concat(fits, ignore_index=True)


def walking_referent_set(
    primary: pd.DataFrame, candidate: pd.DataFrame
) -> pd.DataFrame:
    accepted = set(primary.loc[primary.is_case == 1, "event_id"])
    pool = candidate[
        (candidate.length_label == PRIMARY_LABEL)
        & (candidate.imu_coverage_frac >= COVERAGE_MIN)
    ].copy()
    cases = pool[(pool.is_case == 1) & pool.event_id.isin(accepted)]
    referents = pool[(pool.is_case == 0) & pool.event_id.isin(accepted)].copy()
    referents["ambulatory"] = ambulatory(referents)
    walking = (
        referents[referents.ambulatory == 1]
        .sort_values(
            ["event_id", "gait_band_power_frac"], ascending=[True, False]
        )
        .groupby("event_id")
        .head(TARGET_CONTROLS_PER_CASE)
    )
    return pd.concat([cases, walking], ignore_index=True)


def lag_within_activity(final: pd.DataFrame) -> pd.DataFrame:
    rows = []
    for label in LAG_LABELS:
```

```python
        lag = LAG_SECONDS[label]
        windows = final[final.length_label == label].copy()
        windows["ambulatory"] = ambulatory(windows)
        for arm, subset in [
            ("all windows", windows),
            ("ambulatory windows only", windows[windows.ambulatory == 1]),
        ]:
            result = fit_specification(
                subset, IMU_WITHOUT_POSTURE, f"lag {lag} s, {arm}"
            )
            if "table" not in result:
                continue
            table = result["table"]
            row = table[table.feature == MOVEMENT_FEATURE].iloc[0].to_dict()
            row["lag_s"] = lag
            row["arm"] = arm
            rows.append(row)
    return pd.DataFrame(rows)


def fall_capture(candidate: pd.DataFrame) -> tuple[pd.DataFrame, dict]:
    primary = candidate[candidate.length_label == PRIMARY_LABEL]
    cases = primary[primary.is_case == 1]
    referents = primary[
        (primary.is_case == 0)
        & primary.anchor_fall_ts.notna()
        & ~primary.event_id.astype(str).str.startswith("NEG_")
    ]
    rows = [
        _coverage_row(arm, windows)
        for arm, windows in [
            ("case windows", cases),
            ("clock-matched control candidates", referents),
        ]
    ]
    passing = int((cases.imu_coverage_frac >= COVERAGE_MIN).sum())
    pass_rate = float((referents.imu_coverage_frac >= COVERAGE_MIN).mean())
    test = stats.binomtest(
        passing, len(cases), pass_rate, alternative="greater"
    )
    summary = {
        "n_case_windows": int(len(cases)),
        "n_case_windows_passing_coverage": passing,
        "control_candidate_pass_rate": pass_rate,
        "binomial_p_cases_better_covered": float(test.pvalue),
        "n_control_candidates": int(len(referents)),
        "control_candidate_frac_zero_samples": float(
            (referents.imu_n_samples == 0).mean()
        ),
        "interpretation": (
            "Case windows are almost all fully recorded, whereas a "
            "substantial share of the clock-matched referent candidates "
            "from the same participants on other days contain no samples. "
            "The released falls therefore lie in recorded time; the dataset "
            "documentation states no selection rule."
        ),
    }
    return pd.DataFrame(rows), summary


def _coverage_row(arm: str, windows: pd.DataFrame) -> dict:
    return {
        "arm": arm,
        "n_windows": int(len(windows)),
        "frac_coverage_at_least_0.9": float(
            (windows.imu_coverage_frac >= COVERAGE_MIN).mean()
        ),
        "frac_zero_imu_samples": float((windows.imu_n_samples == 0).mean()),
        "median_coverage_fraction": float(windows.imu_coverage_frac.median()),
    }


def archive_identifier_coverage(
    manifest: pd.DataFrame,
) -> tuple[pd.DataFrame, dict]:
    present = set(manifest["pid"].astype(int))
```

```python
    low, high = DOCUMENTED_ID_RANGE
    table = pd.DataFrame(
        [
            {
                "va_id": identifier,
                "participant": f"va_{identifier}",
                "in_archive": identifier in present,
            }
            for identifier in range(low, high + 1)
        ]
    )
    absent = table.loc[~table.in_archive, "participant"].tolist()
    summary = {
        "readme_range": f"va_{low} to va_{high}",
        "n_ids_in_range": int(len(table)),
        "n_in_archive_in_range": int(table.in_archive.sum()),
        "n_absent_in_range": int(len(absent)),
        "absent_ids": absent,
        "archive_ids_outside_range": sorted(
            f"va_{identifier}"
            for identifier in present
            if not low <= identifier <= high
        ),
    }
    return table, summary


def participant_characteristics(
    final: pd.DataFrame,
    manifest: pd.DataFrame,
    candidate: pd.DataFrame,
    calibration: pd.DataFrame,
) -> pd.DataFrame:
    primary = final[final.length_label == PRIMARY_LABEL]
    candidates = candidate[candidate.length_label == PRIMARY_LABEL]
    usable = candidates[candidates.imu_coverage_frac >= COVERAGE_MIN]
    referent = primary.is_case == 0
    unanchored = primary.anchor_fall_ts.isna()
    excluded = calibration.loc[~calibration.within_expected_1g_range, "va_id"]

    table = manifest.rename(columns={"pid": "va_id"}).copy()
    table["analysed"] = ~table.va_id.isin(set(excluded))
    table["n_analysed_falls"] = _count_by_participant(
        table, primary[primary.is_case == 1]
    )
    table["n_matched_referents"] = _count_by_participant(
        table, primary[referent & ~unanchored]
    )
    table["n_unanchored_windows"] = _count_by_participant(
        table, primary[referent & unanchored]
    )
    table["contributes_to_conditional_model"] = table.va_id.isin(
        informative_participants(primary)
    )
    table["frac_windows_with_usable_imu"] = table.va_id.map(
        candidates.groupby("va_id")["imu_coverage_frac"].apply(
            lambda coverage: float((coverage >= COVERAGE_MIN).mean())
        )
    ).round(3)
    table["frac_usable_windows_ambulatory"] = table.va_id.map(
        usable.assign(moving=ambulatory).groupby("va_id")["moving"].mean()
    ).round(3)
    table["has_tof_days"] = table.tof_days > 0
    return table[
        [
            "participant",
            "analysed",
            "imu_days",
            "calendar_span_days",
            "falls",
            "n_analysed_falls",
            "n_matched_referents",
            "n_unanchored_windows",
            "contributes_to_conditional_model",
            "frac_windows_with_usable_imu",
            "frac_usable_windows_ambulatory",
```

```python
            "has_tof_days",
        ]
    ]


def _count_by_participant(
    table: pd.DataFrame, windows: pd.DataFrame
) -> pd.Series:
    counts = windows.groupby("va_id").size()
    return table.va_id.map(counts).fillna(0).astype(int)


def referent_timing(final: pd.DataFrame, falls: pd.DataFrame) -> pd.DataFrame:
    fall_times = (
        falls.groupby("va_id")["ts_fall_utc"]
        .apply(lambda times: np.sort(times.astype(float).to_numpy()))
        .to_dict()
    )
    rows = []
    for label in LAG_LABELS + LENGTH_LABELS[1:]:
        windows = final[final.length_label == label].copy()
        windows["crosses_dst"] = _crosses_daylight_saving(windows)
        subsets = [
            ("all referents", windows),
            (
                "referents on the case's side of daylight saving",
                windows[~windows.crosses_dst],
            ),
        ]
        if label == PRIMARY_LABEL:
            subsets += _fall_history_subsets(windows, fall_times)
        for referent_set, subset in subsets:
            row = _timing_row(label, referent_set, subset, windows)
            if row is not None:
                rows.append(row)
    return pd.DataFrame(rows)


def _crosses_daylight_saving(windows: pd.DataFrame) -> pd.Series:
    crosses = pd.Series(False, index=windows.index)
    anchored = (windows.is_case == 0) & windows.anchor_fall_ts.notna()
    crosses.loc[anchored] = [
        _dst_regime(anchor) != _dst_regime(end)
        for anchor, end in zip(
            windows.loc[anchored, "anchor_fall_ts"],
            windows.loc[anchored, "window_end_utc"],
            strict=False,
        )
    ]
    return crosses


def _dst_regime(timestamp: float) -> int:
    return int((timestamp >= DST_TRANSITIONS_UTC).sum())


def _fall_history_subsets(
    windows: pd.DataFrame, fall_times: dict
) -> list[tuple[str, pd.DataFrame]]:
    windows["days_since_prior_fall"] = [
        (
            _days_since_prior_fall(row, fall_times)
            if (row.is_case == 0 and not pd.isna(row.anchor_fall_ts))
            else np.inf
        )
        for row in windows.itertuples()
    ]
    return [
        (
            "referents before the participant's first fall",
            windows[
                (windows.is_case == 1)
                | np.isinf(windows.days_since_prior_fall)
            ],
        ),
        (
```

```python
            f"referents at least {MIN_DAYS_AFTER_FALL} days after any fall",
            windows[
                (windows.is_case == 1)
                | (windows.days_since_prior_fall >= MIN_DAYS_AFTER_FALL)
            ],
        ),
    ]


def _days_since_prior_fall(row, fall_times: dict) -> float:
    earlier = [
        moment
        for moment in fall_times.get(int(row.va_id), [])
        if moment < row.window_start_utc
    ]
    if not earlier:
        return np.inf
    return (row.window_start_utc - max(earlier)) / SECONDS_PER_DAY


def _timing_row(
    label: str,
    referent_set: str,
    subset: pd.DataFrame,
    windows: pd.DataFrame,
) -> dict | None:
    result = fit_specification(
        subset, IMU_FEATURES, f"{label} | {referent_set}"
    )
    if "table" not in result:
        return None
    table = result["table"]
    row = table[table.feature == MOVEMENT_FEATURE].iloc[0].to_dict()
    row["window_specification"] = label
    row["referent_set"] = referent_set
    row["n_referents_dropped"] = int(
        (windows.is_case == 0).sum() - (subset.is_case == 0).sum()
    )
    return row


def build_summary(
    referent_counts: dict,
    balance: pd.DataFrame,
    prefall: pd.DataFrame,
    activity: pd.DataFrame,
    lag_activity: pd.DataFrame,
    timing: pd.DataFrame,
    capture_summary: dict,
    identifier_summary: dict,
) -> dict:
    activity_specifications = [
        specification
        for specification in activity.specification.unique()
        if MOVEMENT_FEATURE
        in set(activity[activity.specification == specification].feature)
    ]
    return {
        "referent_days": referent_counts,
        "referent_day_activity_test": balance.to_dict(orient="records"),
        "prefall_referents": {
            specification: _estimate(prefall, specification)
            for specification in prefall.specification.unique()
        },
        "activity_contrast": {
            specification: _estimate(activity, specification)
            for specification in activity_specifications
        },
        "ambulation_indicator_alone": _estimate(
            activity, "ambulation indicator alone", "ambulatory"
        ),
        "ambulation_indicator_in_full_model": _estimate(
            activity,
            "full model, ambulation indicator for posture rate",
            "ambulatory",
        ),
```

```
        "lag_within_activity": _lag_activity_summary(lag_activity),
        "fall_capture_selection": capture_summary,
        "archive_id_coverage": identifier_summary,
        "referent_timing_sensitivity": _timing_summary(timing),
        "lag_window_overlap_with_primary": {
            str(lag): (PRIMARY_WINDOW_SECONDS - lag) / PRIMARY_WINDOW_SECONDS
            for lag in list(LAG_SECONDS.values())[1:]
        },
        "lag_overlap_note": (
            "The lagged designs are nested in the primary one, not "
            "independent of it. Differences between the lag estimates are "
            "therefore not interpretable as the association strengthening "
            "or weakening."
        ),
    }


def _estimate(
    table: pd.DataFrame, specification: str, feature: str = MOVEMENT_FEATURE
) -> dict:
    row = table[
        (table.specification == specification) & (table.feature == feature)
    ].iloc[0]
    return {
        "or": float(row["or"]),
        "ci_lo": float(row.or_ci_lo),
        "ci_hi": float(row.or_ci_hi),
        "p": float(row.p_value),
        "n_rows": int(row.n_rows),
        "n_cases": int(row.n_cases),
        "n_controls": int(row.n_controls),
        "n_strata": int(row.n_informative_strata),
    }


def _lag_activity_summary(lag_activity: pd.DataFrame) -> dict:
    return {
        f"{int(row.lag_s)}s | {row.arm}": {
            "or": float(row["or"]),
            "ci_lo": float(row.or_ci_lo),
            "ci_hi": float(row.or_ci_hi),
            "p": float(row.p_value),
            "n_rows": int(row.n_rows),
            "n_cases": int(row.n_cases),
        }
        for _, row in lag_activity.iterrows()
    }


def _timing_summary(timing: pd.DataFrame) -> dict:
    return {
        f"{row.window_specification} | {row.referent_set}": {
            "or": float(row["or"]),
            "ci_lo": float(row.or_ci_lo),
            "ci_hi": float(row.or_ci_hi),
            "p": float(row.p_value),
            "n_rows": int(row.n_rows),
            "n_cases": int(row.n_cases),
            "n_strata": int(row.n_informative_strata),
            "n_referents_dropped": int(row.n_referents_dropped),
        }
        for _, row in timing.iterrows()
    }


def log_summary(
    summary: dict, characteristics: pd.DataFrame, lag_activity: pd.DataFrame
) -> None:
    counts = summary["referent_days"]
    logger.info("participant characteristics : %d rows", len(characteristics))
    logger.info(
        "referent balance            : %d pre / %d post",
        counts["n_referents_pre_fall"],
        counts["n_referents_post_fall"],
    )
    for group in ("prefall_referents", "activity_contrast"):
```

```python
        for name, value in summary[group].items():
            logger.info(
                "  %-44s OR %.2f (%.2f-%.2f) p=%.4f",
                name,
                value["or"],
                value["ci_lo"],
                value["ci_hi"],
                value["p"],
            )
    for _, row in lag_activity.iterrows():
        logger.info(
            "  lag %4d s %-24s OR %.2f (%.2f-%.2f) n=%d",
            int(row.lag_s),
            row.arm,
            row["or"],
            row.or_ci_lo,
            row.or_ci_hi,
            int(row.n_rows),
        )
    logger.info(
        "fall capture binomial p     : %.2e",
        summary["fall_capture_selection"]["binomial_p_cases_better_covered"],
    )


if __name__ == "__main__":
    main()
```

19 src/descriptive_tables.py

```python
from __future__ import annotations

import json
import logging

import matplotlib.pyplot as plt
import numpy as np
import pandas as pd

import plotting
from config import (
    FEATURE_LABELS,
    FIGURES_DIR,
    IMU_FEATURES,
    LAG_SECONDS,
    METRICS_DIR,
    PRIMARY_LABEL,
    PROCESSED_DIR,
    TABLES_DIR,
    TOF_FEATURES,
    configure_logging,
    ensure_output_dirs,
)

PRIMARY_WINDOW_SECONDS = 300
ALTERNATIVE_WINDOW_SECONDS = [120, 480]
SIGNIFICANCE_LEVEL = 0.05

logger = logging.getLogger(__name__)


def main() -> None:
    configure_logging()
    ensure_output_dirs()
    features = pd.read_csv(TABLES_DIR / "table_features_final.csv")
    primary = features[features.length_label == PRIMARY_LABEL].copy()
    descriptive = describe_by_case_status(primary)
    descriptive.to_csv(
        TABLES_DIR / "table_descriptive_by_case.csv", index=False
    )
    design = design_summary()
    (METRICS_DIR / "design_summary.json").write_text(
        json.dumps(design, indent=2)
```

```
    )
    plot_primary_forest(pd.read_csv(TABLES_DIR / "table_clr_primary.csv"))
    logger.info(
        "wrote table_descriptive_by_case.csv, design_summary.json and "
        "fig1_clr_primary_forest"
    )


def describe_by_case_status(primary: pd.DataFrame) -> pd.DataFrame:
    rows = []
    for feature in IMU_FEATURES + TOF_FEATURES:
        cases = primary.loc[primary.is_case == 1, feature].dropna()
        referents = primary.loc[primary.is_case == 0, feature].dropna()
        rows.append(
            {
                "feature": feature,
                "n_case": int(len(cases)),
                "case_mean": cases.mean(),
                "case_sd": cases.std(),
                "n_control": int(len(referents)),
                "control_mean": referents.mean(),
                "control_sd": referents.std(),
            }
        )
    return pd.DataFrame(rows)


def design_summary() -> dict:
    duplicates = _read_json("duplicate_summary.json")
    selection = _read_json("window_selection_summary.json")
    candidates = _read_json("windows_candidate_summary.json")
    primary = next(
        block
        for block in selection["by_label"]
        if block["length_label"] == PRIMARY_LABEL
    )
    return {
        "n_fall_rows_raw": duplicates["n_raw_rows"],
        "n_fall_events_deduped_primary": duplicates["n_dedup_events"],
        "n_excess_duplicate_rows_removed": duplicates["n_excess_rows_removed"],
        "n_participants_with_falls": duplicates["n_participants_dedup"],
        "n_fall_free_participants": candidates["n_fall_free_participants"],
        "primary_case_window_s": PRIMARY_WINDOW_SECONDS,
        "sensitivity_window_lengths_s": ALTERNATIVE_WINDOW_SECONDS,
        "target_controls_per_case": selection["target_controls_per_case"],
        "achieved_control_to_case_ratio_primary": primary["controls_per_case"],
        "n_case_events_accepted_primary": primary["n_cases_accepted"],
        "n_case_events_dropped_low_coverage": primary[
            "n_cases_dropped_low_coverage"
        ],
        "dropped_case_event_ids": primary["dropped_case_event_ids"],
        "n_matched_controls_primary": primary["n_matched_controls"],
        "n_participants_after_calibration_audit": primary["n_participants"],
        "participants_excluded_scale_anomaly": selection[
            "participants_excluded_scale_anomaly"
        ],
        "coverage_gate_symmetric": selection["coverage_gate_symmetric"],
        "lag_specifications_s": list(LAG_SECONDS.values()),
        "washout_pre_s": candidates["washout_pre_s"],
        "washout_post_s": candidates["washout_post_s"],
        "coverage_gate_min_frac": selection["coverage_min"],
    }


def _read_json(name: str) -> dict:
    return json.loads((PROCESSED_DIR / name).read_text())


def plot_primary_forest(primary_table: pd.DataFrame) -> None:
    plotting.apply_style()
    ordered = primary_table.sort_values("or")
    n_rows = len(ordered)
    fig, ax = plt.subplots(figsize=plotting.forest_figsize(n_rows))
    positions = np.arange(n_rows)
    significant = ordered["p_value"] < SIGNIFICANCE_LEVEL
    ax.errorbar(
```

```python
        ordered["or"],
        positions,
        xerr=[
            ordered["or"] - ordered["or_ci_lo"],
            ordered["or_ci_hi"] - ordered["or"],
        ],
        fmt="none",
        ecolor=plotting.PALETTE["grey"],
        capsize=3,
    )
    for mask, colour, label in [
        (significant, plotting.PALETTE["red"], "$p$ < 0.05"),
        (~significant, plotting.PALETTE["blue"], "$p$ ≥ 0.05"),
    ]:
        if mask.any():
            ax.scatter(
                ordered["or"][mask],
                positions[mask.to_numpy()],
                color=colour,
                zorder=3,
                label=label,
            )
    ax.axvline(
        1.0,
        color=plotting.PALETTE["grey"],
        linestyle="--",
        linewidth=1,
        label="null (OR = 1)",
    )
    ax.set_yticks(positions)
    ax.set_yticklabels([FEATURE_LABELS[name] for name in ordered["feature"]])
    ax.set_xlabel("odds ratio (95% CI), per-SD, conditional logistic model")
    ax.legend(loc="lower right", fontsize=7)
    plotting.adjust_forest(fig)
    plotting.save(fig, FIGURES_DIR, "fig1_clr_primary_forest", tight=False)
    plt.close(fig)


if __name__ == "__main__":
    main()
```

20 src/make_lag_figure.py

```python
from __future__ import annotations

import logging

import matplotlib.pyplot as plt
import numpy as np
import pandas as pd

import plotting
from config import (
    FIGURES_DIR,
    LAG_LABELS,
    LAG_SECONDS,
    TABLES_DIR,
    configure_logging,
)

PANELS = [
    (
        "accel_dynamic_rms_gravity_removed",
        "Dynamic acceleration RMS, gravity-removed",
    ),
    ("jerk_max", "Peak jerk"),
]
LOG_TICKS = [0.5, 1, 2, 4, 8]

logger = logging.getLogger(__name__)


def main() -> None:
```

```python
    configure_logging()
    estimates = pd.read_csv(TABLES_DIR / "table_clr_all_specifications.csv")
    plotting.apply_style()
    fig, axes = plt.subplots(1, 2, figsize=(plotting.WIDTH_FULL * 2, 4.2))
    for ax, (feature, title), letter in zip(axes, PANELS, "ab", strict=False):
        lag_rows = lag_estimates(estimates, feature)
        draw_panel(ax, lag_rows, title, letter)
    plotting.save(fig, FIGURES_DIR, "fig2_lag_analysis", pad=0.2)
    plt.close(fig)
    logger.info("wrote fig2_lag_analysis")


def lag_estimates(estimates: pd.DataFrame, feature: str) -> np.ndarray:
    rows = []
    for label in LAG_LABELS:
        match = estimates[
            (estimates.specification == f"{label}_imu")
            & (estimates.feature == feature)
        ]
        if len(match):
            row = match.iloc[0]
            rows.append(
                (
                    LAG_SECONDS[label],
                    row["or"],
                    row["or_ci_lo"],
                    row["or_ci_hi"],
                    row["p_value"],
                )
            )
    return np.array(rows, dtype=float)


def draw_panel(ax, lag_rows: np.ndarray, title: str, letter: str) -> None:
    lags, odds_ratios = lag_rows[:, 0], lag_rows[:, 1]
    low, high = lag_rows[:, 2], lag_rows[:, 3]
    significant = lag_rows[:, 4] < 0.05
    ax.errorbar(
        lags,
        odds_ratios,
        yerr=[odds_ratios - low, high - odds_ratios],
        color=plotting.PALETTE["blue"],
        linestyle="-",
        marker="o",
        markersize=5,
        capsize=3,
        label="Odds ratio per SD (95% CI)",
        zorder=2,
    )
    if significant.any():
        ax.scatter(
            lags[significant],
            odds_ratios[significant],
            s=70,
            color=plotting.PALETTE["red"],
            zorder=3,
            edgecolors="none",
            label="$p$ < 0.05",
        )
    ax.axhline(
        1.0,
        color=plotting.PALETTE["grey"],
        linestyle="--",
        linewidth=1,
        label="No association (OR = 1)",
    )
    ax.set_yscale("log")
    ax.set_yticks(LOG_TICKS)
    ax.set_yticklabels([f"{tick:g}" for tick in LOG_TICKS])
    ax.set_xticks(lags)
    ax.set_xlabel("Lag (s): how far before the annotated fall the window ends")
    ax.set_ylabel("Odds ratio per SD")
    ax.set_title(title, fontsize=9)
    ax.legend(loc="upper right", fontsize=7)
    plotting.panel(ax, letter)

```

```python

if __name__ == "__main__":
    main()
```

21 src/make_referent_activity_figure.py

```python
from __future__ import annotations

import logging

import matplotlib.pyplot as plt
import numpy as np
import pandas as pd

import plotting
from config import FIGURES_DIR, MOVEMENT_FEATURE, TABLES_DIR, configure_logging

REFERENT_PANEL = [
    ("primary (all referent days)", "Referents as drawn"),
    ("pre-fall referent days only", "Pre-fall referent days only"),
    ("post-fall referent days only", "Post-fall referent days only"),
]
ACTIVITY_PANEL = [
    ("primary", "Primary model"),
    (
        "full model, ambulation indicator for posture rate",
        "Adjusted for ambulation",
    ),
    (
        "restricted to ambulatory windows, both arms",
        "Ambulatory windows only",
    ),
    ("walking referents, full model", "Walking referents"),
]
LOG_TICKS = [0.5, 1, 2, 4, 8]

logger = logging.getLogger(__name__)


def main() -> None:
    configure_logging()
    referents = pd.read_csv(TABLES_DIR / "table_clr_prefall_referents.csv")
    activity = pd.read_csv(TABLES_DIR / "table_clr_activity_contrast.csv")
    plotting.apply_style()
    fig, axes = plt.subplots(1, 2, figsize=(plotting.WIDTH_FULL * 2, 4.2))
    for ax, table, panel_rows, letter, title in [
        (axes[0], referents, REFERENT_PANEL, "a", "Referent day selection"),
        (
            axes[1],
            activity,
            ACTIVITY_PANEL,
            "b",
            "Holding activity comparable",
        ),
    ]:
        estimates = movement_estimates(table, panel_rows)
        labels = [label for _, label in panel_rows]
        draw_panel(ax, estimates, labels, letter, title)
    fig.subplots_adjust(
        left=0.20, right=0.97, wspace=0.55, bottom=0.16, top=0.90
    )
    plotting.save(
        fig, FIGURES_DIR, "fig3_referent_activity_sensitivity", tight=False
    )
    plt.close(fig)
    logger.info("wrote fig3_referent_activity_sensitivity")


def movement_estimates(
    table: pd.DataFrame, panel_rows: list[tuple[str, str]]
) -> np.ndarray:
    rows = []
    for specification, _ in panel_rows:
```

```python
        row = table[
            (table.specification == specification)
            & (table.feature == MOVEMENT_FEATURE)
        ].iloc[0]
        rows.append(
            (row["or"], row["or_ci_lo"], row["or_ci_hi"], row["p_value"])
        )
    return np.array(rows, dtype=float)


def draw_panel(
    ax, estimates: np.ndarray, labels: list[str], letter: str, title: str
) -> None:
    positions = np.arange(len(estimates))[::-1]
    odds_ratios, low, high = estimates[:, 0], estimates[:, 1], estimates[:, 2]
    significant = estimates[:, 3] < 0.05
    for mask, colour, label in [
        (~significant, plotting.PALETTE["grey"], "$p$ ≥ 0.05"),
        (significant, plotting.PALETTE["blue"], "$p$ < 0.05"),
    ]:
        ax.errorbar(
            odds_ratios[mask],
            positions[mask],
            xerr=[(odds_ratios - low)[mask], (high - odds_ratios)[mask]],
            fmt="o",
            markersize=5,
            capsize=3,
            color=colour,
            linestyle="none",
            label=label,
        )
    ax.axvline(
        1.0,
        color=plotting.PALETTE["grey"],
        linestyle="--",
        linewidth=1,
        label="No association (OR = 1)",
    )
    ax.set_yticks(positions)
    ax.set_yticklabels(labels, fontsize=8)
    ax.set_ylim(-1.7, len(estimates) - 0.3)
    ax.set_xscale("log")
    ax.set_xticks(LOG_TICKS)
    ax.set_xticklabels([f"{tick:g}" for tick in LOG_TICKS])
    ax.set_xlabel("Odds ratio per SD, movement intensity")
    ax.set_title(title, fontsize=9)
    ax.legend(loc="lower right", fontsize=7)
    plotting.panel(ax, letter)


if __name__ == "__main__":
    main()
```

22 scripts/run_all.py

```python
from __future__ import annotations

import logging
import subprocess
import sys
from pathlib import Path

logger = logging.getLogger(__name__)

SOURCE_DIR = Path(__file__).resolve().parents[1] / "src"

STEPS = [
    "build_manifest.py",
    "deduplicate_falls.py",
    "build_window_index.py",
    "verify_archive_integrity.py",
    "extract_features.py",
    "select_windows.py",
```

```
    "leakage_audit.py",
    "fit_conditional_models.py",
    "jerk_time_base_comparison.py",
    "leave_one_participant_out.py",
    "permutation_control.py",
    "fit_boosted_model.py",
    "descriptive_tables.py",
    "make_lag_figure.py",
    "referent_and_activity_analyses.py",
    "make_referent_activity_figure.py",
]


def main() -> None:
    logging.basicConfig(level=logging.INFO, format="%(message)s")
    first = sys.argv[1] if len(sys.argv) > 1 else STEPS[0]
    for step in STEPS[STEPS.index(first) :]:
        logger.info("\n%s\n%s\n%s", "=" * 70, step, "=" * 70)
        result = subprocess.run(
            [sys.executable, str(SOURCE_DIR / step)],
            cwd=str(SOURCE_DIR),
            check=False,
        )
        if result.returncode != 0:
            logger.error("FAILED at %s (exit %s)", step, result.returncode)
            sys.exit(result.returncode)
    logger.info("\nAll steps completed.")


if __name__ == "__main__":
    main()
```
