# Supplementary material for: Movement Intensity in the Minutes Before a Fall: A Lag-Controlled, Activity-Matched Time-Stratified Case-Crossover Analysis of Home Monitoring in Multiple Sclerosis
Source: J Clin Med. 2026 Oct 26;15(21):8323. doi: 10.3390/jcm15218323 (PMC13764606; doi:10.3390/jcm15218323)
Supplement: Supplementary file 1 [file jcm-15-08323-s001.zip › Supplement_S2_Tables.pdf]

Supplementary Tables

This file accompanies the manuscript *Movement Intensity in the Minutes Before a Fall: A Lag-Controlled, Activity-Matched Time-Stratified Case-Crossover Analysis of Home Monitoring in Multiple Sclerosis* as Supplementary File S2. Every table is generated from the result files written by the analysis code in Supplementary File S1, and the source file is named in each caption. Column names are those of the source file; numeric values are rounded to four significant figures and are otherwise unchanged. A blank cell means the value is not defined or was not recorded for that row. Tables S7 to S9 are window-level tables; S2 gives their column dictionaries, and Supplementary File S3 holds them in full.

Table S1. Cohort manifest: monitored days, calendar span and observed fall count for each of the 26 participants in the archive, as measured from the archive index (Section 2.1). (source: `table_cohort_manifest.csv`).

participant	pid	imu_days	tof_days	distinct_days	first_day	last_day	calendar_span_days	falls
va_4	4	25	26	26	161007	161107	32	0
va_5	5	60	60	60	161011	161212	63	0
va_6	6	49	52	52	161013	161213	62	16
va_7	7	44	0	44	161021	161220	61	0
va_8	8	40	40	40	161025	161209	46	0
va_9	9	56	56	56	161027	161222	57	2
va_10	10	66	0	66	161101	170105	66	0
va_11	11	63	63	63	161103	170105	64	15
va_12	12	60	59	60	161110	170108	60	0
va_13	13	57	65	65	170418	170623	67	2
va_14	14	55	62	62	170419	170622	65	9
va_15	15	49	65	65	170420	170623	65	0
va_16	16	22	22	22	170421	170519	29	1
va_17	17	24	28	28	170426	170529	34	0
va_18	18	53	61	61	170427	170627	62	0
va_19	19	32	44	44	170428	170629	63	0
va_20	20	47	61	61	170503	170706	65	1
va_21	21	37	43	43	170504	170627	55	2
va_22	22	41	54	54	170505	170708	65	0
va_23	23	56	57	57	171116	180111	57	0
va_24	24	41	43	43	171122	180204	75	1
va_25	25	57	58	58	171121	180125	66	0
va_26	26	63	64	64	171128	180213	78	2
va_28	28	46	48	48	171212	180213	64	0
va_30	30	45	47	47	171214	180219	68	1
va_33	33	74	74	74	171219	180302	74	2

Table S2. Archive-integrity verification: every archive member referenced by the analysis was read in full and its stored checksum verified before feature extraction (Section 2.9). Of the 1279 members checked, 1 failed. (source: `table_archive_integrity.csv`).

kind	va_id	day	member	declared_size_bytes	status	detail
imu	10	161102	161102_va_10_motio_19_raw.npz	20404998	ok	
imu	10	161111	161111_va_10_motio_19_raw.npz	15270057	ok	
imu	10	161112	161112_va_10_motio_19_raw.npz	18750718	ok	
imu	10	161115	161115_va_10_motio_19_raw.npz	16577946	ok	
imu	10	161116	161116_va_10_motio_19_raw.npz	9856421	ok	
imu	10	161118	161118_va_10_motio_19_raw.npz	19753716	ok	
imu	10	161124	161124_va_10_motio_19_raw.npz	15781958	ok	
imu	10	161125	161125_va_10_motio_19_raw.npz	16916140	ok	
imu	10	161128	161128_va_10_motio_19_raw.npz	18478552	ok	
imu	10	161201	161201_va_10_motio_19_raw.npz	15283951	ok	
imu	10	161202	161202_va_10_motio_19_raw.npz	19875240	ok	
imu	10	161208	161208_va_10_motio_19_raw.npz	14458289	ok	
imu	10	161213	161213_va_10_motio_19_raw.npz	12864227	ok	
imu	10	161218	161218_va_10_motio_19_raw.npz	17328499	ok	
imu	10	161222	161222_va_10_motio_19_raw.npz	15415640	ok	
imu	10	161229	161229_va_10_motio_19_raw.npz	15022691	ok	
imu	10	161230	161230_va_10_motio_19_raw.npz	14564690	ok	
imu	10	161231	161231_va_10_motio_19_raw.npz	14314155	ok	
imu	10	170103	170103_va_10_motio_19_raw.npz	11580526	ok	
imu	10	170104	170104_va_10_motio_19_raw.npz	14259203	ok	
imu	11	161103	161103_va_11_motio_20_raw.npz	6730509	ok	
tof	11	161103	161103_va_11_motio_20_context_raw.npz	715835	ok	
imu	11	161104	161104_va_11_motio_20_raw.npz	28823323	ok	
tof	11	161104	161104_va_11_motio_20_context_raw.npz	12759379	ok	
imu	11	161105	161105_va_11_motio_20_raw.npz	28392424	ok	
tof	11	161105	161105_va_11_motio_20_context_raw.npz	6859720	ok	
imu	11	161106	161106_va_11_motio_20_raw.npz	28185768	ok	
tof	11	161106	161106_va_11_motio_20_context_raw.npz	10482806	ok	
imu	11	161107	161107_va_11_motio_20_raw.npz	27662044	ok	
tof	11	161107	161107_va_11_motio_20_context_raw.npz	10134564	ok	

kind	va_id	day	member	declared_size_bytes	status	detail
imu	11	161108	161108_va_11_motio_20_raw.npz	30922557	ok	
tof	11	161108	161108_va_11_motio_20_context_raw.npz	14870131	ok	
imu	11	161109	161109_va_11_motio_20_raw.npz	29490095	ok	
tof	11	161109	161109_va_11_motio_20_context_raw.npz	11743301	ok	
imu	11	161110	161110_va_11_motio_20_raw.npz	27381115	ok	
tof	11	161110	161110_va_11_motio_20_context_raw.npz	13033889	ok	
imu	11	161111	161111_va_11_motio_20_raw.npz	27034069	ok	
tof	11	161111	161111_va_11_motio_20_context_raw.npz	12923997	ok	
imu	11	161112	161112_va_11_motio_20_raw.npz	27150683	ok	
tof	11	161112	161112_va_11_motio_20_context_raw.npz	10698136	ok	
imu	11	161113	161113_va_11_motio_20_raw.npz	25312782	ok	
tof	11	161113	161113_va_11_motio_20_context_raw.npz	6980232	ok	
imu	11	161114	161114_va_11_motio_20_raw.npz	28558696	ok	
tof	11	161114	161114_va_11_motio_20_context_raw.npz	10419817	ok	
imu	11	161115	161115_va_11_motio_20_raw.npz	28043493	ok	
tof	11	161115	161115_va_11_motio_20_context_raw.npz	10834262	ok	
imu	11	161116	161116_va_11_motio_20_raw.npz	18233221	ok	
tof	11	161116	161116_va_11_motio_20_context_raw.npz	10974538	ok	
imu	11	161117	161117_va_11_motio_20_raw.npz	26949733	ok	
tof	11	161117	161117_va_11_motio_20_context_raw.npz	12970428	ok	
imu	11	161118	161118_va_11_motio_20_raw.npz	25994171	ok	
tof	11	161118	161118_va_11_motio_20_context_raw.npz	12926918	ok	
imu	11	161119	161119_va_11_motio_20_raw.npz	29011082	ok	
tof	11	161119	161119_va_11_motio_20_context_raw.npz	10655414	ok	
imu	11	161120	161120_va_11_motio_20_raw.npz	31313049	ok	
tof	11	161120	161120_va_11_motio_20_context_raw.npz	12820878	ok	
imu	11	161121	161121_va_11_motio_20_raw.npz	28594996	ok	
tof	11	161121	161121_va_11_motio_20_context_raw.npz	12566598	ok	
imu	11	161122	161122_va_11_motio_20_raw.npz	25937018	ok	
tof	11	161122	161122_va_11_motio_20_context_raw.npz	10805966	ok	
imu	11	161123	161123_va_11_motio_20_raw.npz	29412751	ok	
tof	11	161123	161123_va_11_motio_20_context_raw.npz	10250362	ok	
imu	11	161124	161124_va_11_motio_20_raw.npz	28315635	ok	
tof	11	161124	161124_va_11_motio_20_context_raw.npz	10055355	ok	
imu	11	161125	161125_va_11_motio_20_raw.npz	24975221	ok	
tof	11	161125	161125_va_11_motio_20_context_raw.npz	7873328	ok	
imu	11	161126	161126_va_11_motio_20_raw.npz	26947256	ok	
tof	11	161126	161126_va_11_motio_20_context_raw.npz	9232842	ok	
imu	11	161127	161127_va_11_motio_20_raw.npz	28320096	ok	
tof	11	161127	161127_va_11_motio_20_context_raw.npz	11370269	ok	
imu	11	161128	161128_va_11_motio_20_raw.npz	26072648	ok	
tof	11	161128	161128_va_11_motio_20_context_raw.npz	12625683	ok	
imu	11	161129	161129_va_11_motio_20_raw.npz	25567355	ok	
tof	11	161129	161129_va_11_motio_20_context_raw.npz	11273673	ok	
imu	11	161130	161130_va_11_motio_20_raw.npz	28742186	ok	
tof	11	161130	161130_va_11_motio_20_context_raw.npz	8569425	ok	
imu	11	161201	161201_va_11_motio_20_raw.npz	23562843	ok	
tof	11	161201	161201_va_11_motio_20_context_raw.npz	8112120	ok	
imu	11	161202	161202_va_11_motio_20_raw.npz	25168460	ok	
tof	11	161202	161202_va_11_motio_20_context_raw.npz	12795547	ok	
imu	11	161203	161203_va_11_motio_20_raw.npz	27986942	ok	
tof	11	161203	161203_va_11_motio_20_context_raw.npz	12556568	ok	
imu	11	161204	161204_va_11_motio_20_raw.npz	27190314	ok	
tof	11	161204	161204_va_11_motio_20_context_raw.npz	7975640	ok	
imu	11	161205	161205_va_11_motio_20_raw.npz	28226282	ok	
tof	11	161205	161205_va_11_motio_20_context_raw.npz	10540036	ok	
imu	11	161206	161206_va_11_motio_20_raw.npz	29002032	ok	
tof	11	161206	161206_va_11_motio_20_context_raw.npz	11830005	ok	
imu	11	161207	161207_va_11_motio_20_raw.npz	25719481	ok	
tof	11	161207	161207_va_11_motio_20_context_raw.npz	8505696	ok	
imu	11	161208	161208_va_11_motio_20_raw.npz	28559667	ok	
tof	11	161208	161208_va_11_motio_20_context_raw.npz	10769078	ok	
imu	11	161209	161209_va_11_motio_20_raw.npz	27752277	ok	
tof	11	161209	161209_va_11_motio_20_context_raw.npz	12532068	ok	
imu	11	161210	161210_va_11_motio_20_raw.npz	28588867	ok	
tof	11	161210	161210_va_11_motio_20_context_raw.npz	12105899	ok	
imu	11	161212	161212_va_11_motio_20_raw.npz	28397741	ok	
tof	11	161212	161212_va_11_motio_20_context_raw.npz	10556243	ok	
imu	11	161214	161214_va_11_motio_20_raw.npz	11647915	ok	
tof	11	161214	161214_va_11_motio_20_context_raw.npz	4067078	ok	
imu	11	161215	161215_va_11_motio_20_raw.npz	28277699	ok	
tof	11	161215	161215_va_11_motio_20_context_raw.npz	13378258	ok	
imu	11	161216	161216_va_11_motio_20_raw.npz	24730057	ok	
tof	11	161216	161216_va_11_motio_20_context_raw.npz	12295949	ok	
imu	11	161217	161217_va_11_motio_20_raw.npz	28442244	ok	
tof	11	161217	161217_va_11_motio_20_context_raw.npz	12414759	ok	
imu	11	161218	161218_va_11_motio_20_raw.npz	23481883	ok	
tof	11	161218	161218_va_11_motio_20_context_raw.npz	11522538	ok	
imu	11	161219	161219_va_11_motio_20_raw.npz	27217874	ok	
tof	11	161219	161219_va_11_motio_20_context_raw.npz	13092214	ok	
imu	11	161220	161220_va_11_motio_20_raw.npz	27309810	ok	
tof	11	161220	161220_va_11_motio_20_context_raw.npz	8868331	ok	
imu	11	161221	161221_va_11_motio_20_raw.npz	28629912	ok	
tof	11	161221	161221_va_11_motio_20_context_raw.npz	11132711	ok	
imu	11	161222	161222_va_11_motio_20_raw.npz	27933162	ok	
tof	11	161222	161222_va_11_motio_20_context_raw.npz	11276356	ok	

kind	va_id	day	member	declared_size_bytes	status	detail
imu	11	161223	161223_va_11_motio_20_raw.npz	27199992	ok	
tof	11	161223	161223_va_11_motio_20_context_raw.npz	10583846	ok	
imu	11	161224	161224_va_11_motio_20_raw.npz	26422508	ok	
tof	11	161224	161224_va_11_motio_20_context_raw.npz	10767325	ok	
imu	11	161225	161225_va_11_motio_20_raw.npz	26417477	ok	
tof	11	161225	161225_va_11_motio_20_context_raw.npz	12037312	ok	
imu	11	161226	161226_va_11_motio_20_raw.npz	24012754	ok	
tof	11	161226	161226_va_11_motio_20_context_raw.npz	8646773	ok	
imu	11	161227	161227_va_11_motio_20_raw.npz	26573918	ok	
tof	11	161227	161227_va_11_motio_20_context_raw.npz	12153911	ok	
imu	11	161228	161228_va_11_motio_20_raw.npz	26979315	ok	
tof	11	161228	161228_va_11_motio_20_context_raw.npz	11204652	ok	
imu	11	161229	161229_va_11_motio_20_raw.npz	26852437	ok	
tof	11	161229	161229_va_11_motio_20_context_raw.npz	11829525	ok	
imu	11	161230	161230_va_11_motio_20_raw.npz	28539760	ok	
tof	11	161230	161230_va_11_motio_20_context_raw.npz	11790658	ok	
imu	11	161231	161231_va_11_motio_20_raw.npz	28191241	ok	
tof	11	161231	161231_va_11_motio_20_context_raw.npz	10026268	ok	
imu	11	170101	170101_va_11_motio_20_raw.npz	28610344	ok	
tof	11	170101	170101_va_11_motio_20_context_raw.npz	11078538	ok	
imu	11	170102	170102_va_11_motio_20_raw.npz	27909630	ok	
tof	11	170102	170102_va_11_motio_20_context_raw.npz	8717558	ok	
imu	11	170103	170103_va_11_motio_20_raw.npz	29641410	ok	
tof	11	170103	170103_va_11_motio_20_context_raw.npz	10301857	ok	
imu	11	170105	170105_va_11_motio_20_raw.npz	20823420	ok	
tof	11	170105	170105_va_11_motio_20_context_raw.npz	6042439	ok	
imu	12	161112	161112_va_12_motio_21_raw.npz	24502317	ok	
tof	12	161112	161112_va_12_motio_21_context_raw.npz	5968436	ok	
imu	12	161113	161113_va_12_motio_21_raw.npz	21173057	ok	
tof	12	161113	161113_va_12_motio_21_context_raw.npz	7212303	ok	
imu	12	161114	161114_va_12_motio_21_raw.npz	24231853	ok	
tof	12	161114	161114_va_12_motio_21_context_raw.npz	7134473	ok	
imu	12	161117	161117_va_12_motio_21_raw.npz	21663933	ok	
tof	12	161117	161117_va_12_motio_21_context_raw.npz	6801256	ok	
imu	12	161118	161118_va_12_motio_21_raw.npz	23351356	ok	
tof	12	161118	161118_va_12_motio_21_context_raw.npz	7892637	ok	
imu	12	161205	161205_va_12_motio_21_raw.npz	12060624	ok	
tof	12	161205	161205_va_12_motio_21_context_raw.npz	2084072	ok	
imu	12	161208	161208_va_12_motio_21_raw.npz	22279189	ok	
tof	12	161208	161208_va_12_motio_21_context_raw.npz	7236145	ok	
imu	12	161211	161211_va_12_motio_21_raw.npz	23842733	ok	
tof	12	161211	161211_va_12_motio_21_context_raw.npz	4979805	ok	
imu	12	161212	161212_va_12_motio_21_raw.npz	22796579	ok	
tof	12	161212	161212_va_12_motio_21_context_raw.npz	6343576	ok	
imu	12	161214	161214_va_12_motio_21_raw.npz	24506018	ok	
tof	12	161214	161214_va_12_motio_21_context_raw.npz	6702361	ok	
imu	12	161215	161215_va_12_motio_21_raw.npz	20852047	ok	
tof	12	161215	161215_va_12_motio_21_context_raw.npz	9017758	ok	
imu	12	161216	161216_va_12_motio_21_raw.npz	24741699	ok	
tof	12	161216	161216_va_12_motio_21_context_raw.npz	6104316	ok	
imu	12	161223	161223_va_12_motio_21_raw.npz	22232693	ok	
tof	12	161223	161223_va_12_motio_21_context_raw.npz	8180912	ok	
imu	12	161225	161225_va_12_motio_21_raw.npz	24571918	ok	
tof	12	161225	161225_va_12_motio_21_context_raw.npz	10040911	ok	
imu	12	161226	161226_va_12_motio_21_raw.npz	23243664	ok	
tof	12	161226	161226_va_12_motio_21_context_raw.npz	4742896	ok	
imu	12	161227	161227_va_12_motio_21_raw.npz	24114195	ok	
tof	12	161227	161227_va_12_motio_21_context_raw.npz	7427880	ok	
imu	12	161229	161229_va_12_motio_21_raw.npz	21321652	ok	
tof	12	161229	161229_va_12_motio_21_context_raw.npz	6040171	ok	
imu	12	161231	161231_va_12_motio_21_raw.npz	23540884	ok	
tof	12	161231	161231_va_12_motio_21_context_raw.npz	6191370	ok	
imu	12	170103	170103_va_12_motio_21_raw.npz	24241318	ok	
tof	12	170103	170103_va_12_motio_21_context_raw.npz	7373592	ok	
imu	12	170106	170106_va_12_motio_21_raw.npz	23337531	ok	
tof	12	170106	170106_va_12_motio_21_context_raw.npz	8712603	ok	
imu	12	170107	170107_va_12_motio_21_raw.npz	20142666	ok	
tof	12	170107	170107_va_12_motio_21_context_raw.npz	8969743	ok	
imu	13	170418	170418_va_13_motio_18_raw.npz	8718442	ok	
tof	13	170418	170418_va_13_motio_18_context_raw.npz	3920011	ok	
imu	13	170420	170420_va_13_motio_18_raw.npz	15342253	ok	
tof	13	170420	170420_va_13_motio_18_context_raw.npz	4427301	ok	
imu	13	170421	170421_va_13_motio_18_raw.npz	19604213	ok	
tof	13	170421	170421_va_13_motio_18_context_raw.npz	4799927	ok	
imu	13	170424	170424_va_13_motio_18_raw.npz	18506550	ok	
tof	13	170424	170424_va_13_motio_18_context_raw.npz	6616753	ok	
imu	13	170425	170425_va_13_motio_18_raw.npz	22601595	ok	
tof	13	170425	170425_va_13_motio_18_context_raw.npz	3923391	ok	
imu	13	170428	170428_va_13_motio_18_raw.npz	25070123	ok	
tof	13	170428	170428_va_13_motio_18_context_raw.npz	7125129	ok	
imu	13	170429	170429_va_13_motio_18_raw.npz	22523047	ok	
tof	13	170429	170429_va_13_motio_18_context_raw.npz	7845362	ok	
imu	13	170501	170501_va_13_motio_18_raw.npz	16327921	ok	
tof	13	170501	170501_va_13_motio_18_context_raw.npz	2855879	ok	
imu	13	170506	170506_va_13_motio_18_raw.npz	13951584	ok	
tof	13	170506	170506_va_13_motio_18_context_raw.npz	4638437	ok	

kind	va_id	day	member	declared_size_bytes	status	detail
imu	13	170507	170507_va_13_motio_18_raw.npz	5136771	ok	
tof	13	170507	170507_va_13_motio_18_context_raw.npz	657339	ok	
imu	13	170510	170510_va_13_motio_18_raw.npz	13836616	ok	
tof	13	170510	170510_va_13_motio_18_context_raw.npz	3572263	ok	
imu	13	170513	170513_va_13_motio_18_raw.npz	15427280	ok	
tof	13	170513	170513_va_13_motio_18_context_raw.npz	4740927	ok	
imu	13	170515	170515_va_13_motio_18_raw.npz	19071875	ok	
tof	13	170515	170515_va_13_motio_18_context_raw.npz	6897939	ok	
imu	13	170516	170516_va_13_motio_18_raw.npz	17547051	ok	
tof	13	170516	170516_va_13_motio_18_context_raw.npz	6102922	ok	
imu	13	170518	170518_va_13_motio_18_raw.npz	12245400	ok	
tof	13	170518	170518_va_13_motio_18_context_raw.npz	4031057	ok	
imu	13	170520	170520_va_13_motio_18_raw.npz	19340686	ok	
tof	13	170520	170520_va_13_motio_18_context_raw.npz	4813408	ok	
imu	13	170522	170522_va_13_motio_18_raw.npz	21635612	ok	
tof	13	170522	170522_va_13_motio_18_context_raw.npz	4339072	ok	
imu	13	170524	170524_va_13_motio_18_raw.npz	23302538	ok	
tof	13	170524	170524_va_13_motio_18_context_raw.npz	5508115	ok	
imu	13	170526	170526_va_13_motio_18_raw.npz	17232381	ok	
tof	13	170526	170526_va_13_motio_18_context_raw.npz	3493664	ok	
imu	13	170527	170527_va_13_motio_18_raw.npz	23121168	ok	
tof	13	170527	170527_va_13_motio_18_context_raw.npz	2583366	ok	
imu	13	170531	170531_va_13_motio_18_raw.npz	13940464	ok	
tof	13	170531	170531_va_13_motio_18_context_raw.npz	1041089	ok	
imu	13	170601	170601_va_13_motio_18_raw.npz	24868783	ok	
tof	13	170601	170601_va_13_motio_18_context_raw.npz	8552349	ok	
imu	13	170602	170602_va_13_motio_18_raw.npz	24008592	ok	
tof	13	170602	170602_va_13_motio_18_context_raw.npz	5520290	ok	
imu	13	170603	170603_va_13_motio_18_raw.npz	19453583	ok	
tof	13	170603	170603_va_13_motio_18_context_raw.npz	10136784	ok	
imu	13	170604	170604_va_13_motio_18_raw.npz	21912010	ok	
tof	13	170604	170604_va_13_motio_18_context_raw.npz	5235156	ok	
imu	13	170608	170608_va_13_motio_18_raw.npz	20762634	ok	
tof	13	170608	170608_va_13_motio_18_context_raw.npz	5402995	ok	
imu	13	170610	170610_va_13_motio_18_raw.npz	8452329	ok	
tof	13	170610	170610_va_13_motio_18_context_raw.npz	3828062	ok	
imu	13	170611	170611_va_13_motio_18_raw.npz	4787870	ok	
tof	13	170611	170611_va_13_motio_18_context_raw.npz	1898103	ok	
imu	13	170614	170614_va_13_motio_18_raw.npz	22575477	ok	
tof	13	170614	170614_va_13_motio_18_context_raw.npz	4641298	ok	
imu	13	170616	170616_va_13_motio_18_raw.npz	22025322	ok	
tof	13	170616	170616_va_13_motio_18_context_raw.npz	3399921	ok	
imu	13	170617	170617_va_13_motio_18_raw.npz	9336542	ok	
tof	13	170617	170617_va_13_motio_18_context_raw.npz	2898295	ok	
imu	14	170419	170419_va_14_motio_19_raw.npz	8445583	ok	
tof	14	170419	170419_va_14_motio_19_context_raw.npz	4411746	ok	
imu	14	170420	170420_va_14_motio_19_raw.npz	28501615	ok	
tof	14	170420	170420_va_14_motio_19_context_raw.npz	13463625	ok	
imu	14	170421	170421_va_14_motio_19_raw.npz	16958133	ok	
tof	14	170421	170421_va_14_motio_19_context_raw.npz	5873455	ok	
imu	14	170422	170422_va_14_motio_19_raw.npz	27775126	ok	
tof	14	170422	170422_va_14_motio_19_context_raw.npz	13530995	ok	
imu	14	170424	170424_va_14_motio_19_raw.npz	29112020	ok	
tof	14	170424	170424_va_14_motio_19_context_raw.npz	9750395	ok	
imu	14	170428	170428_va_14_motio_19_raw.npz	25124382	ok	
tof	14	170428	170428_va_14_motio_19_context_raw.npz	12745022	ok	
imu	14	170429	170429_va_14_motio_19_raw.npz	23438689	ok	
tof	14	170429	170429_va_14_motio_19_context_raw.npz	12868834	ok	
imu	14	170430	170430_va_14_motio_19_raw.npz	26502112	ok	
tof	14	170430	170430_va_14_motio_19_context_raw.npz	11516732	ok	
imu	14	170501	170501_va_14_motio_19_raw.npz	29404987	ok	
tof	14	170501	170501_va_14_motio_19_context_raw.npz	6517790	ok	
imu	14	170502	170502_va_14_motio_19_raw.npz	28380813	ok	
tof	14	170502	170502_va_14_motio_19_context_raw.npz	8867238	ok	
imu	14	170503	170503_va_14_motio_19_raw.npz	28012058	ok	
tof	14	170503	170503_va_14_motio_19_context_raw.npz	13595365	ok	
imu	14	170504	170504_va_14_motio_19_raw.npz	26320604	ok	
tof	14	170504	170504_va_14_motio_19_context_raw.npz	9476041	ok	
imu	14	170505	170505_va_14_motio_19_raw.npz	11399637	ok	
tof	14	170505	170505_va_14_motio_19_context_raw.npz	12205451	ok	
imu	14	170508	170508_va_14_motio_19_raw.npz	21757921	ok	
tof	14	170508	170508_va_14_motio_19_context_raw.npz	6552796	ok	
imu	14	170509	170509_va_14_motio_19_raw.npz	24345614	ok	
tof	14	170509	170509_va_14_motio_19_context_raw.npz	12019414	ok	
imu	14	170511	170511_va_14_motio_19_raw.npz	26714924	ok	
tof	14	170511	170511_va_14_motio_19_context_raw.npz	13224347	ok	
imu	14	170512	170512_va_14_motio_19_raw.npz	16691351	ok	
tof	14	170512	170512_va_14_motio_19_context_raw.npz	7501390	ok	
imu	14	170513	170513_va_14_motio_19_raw.npz	27704727	ok	
tof	14	170513	170513_va_14_motio_19_context_raw.npz	12278807	ok	
imu	14	170514	170514_va_14_motio_19_raw.npz	30206304	ok	
tof	14	170514	170514_va_14_motio_19_context_raw.npz	14999565	ok	
imu	14	170516	170516_va_14_motio_19_raw.npz	27776186	ok	
tof	14	170516	170516_va_14_motio_19_context_raw.npz	10831898	ok	
imu	14	170517	170517_va_14_motio_19_raw.npz	27008247	ok	
tof	14	170517	170517_va_14_motio_19_context_raw.npz	11701700	ok	

kind	va_id	day	member	declared_size_bytes	status	detail
imu	14	170518	170518_va_14_motio_19_raw.npz	10621109	ok	
tof	14	170518	170518_va_14_motio_19_context_raw.npz	4054046	ok	
imu	14	170519	170519_va_14_motio_19_raw.npz	23234735	ok	
tof	14	170519	170519_va_14_motio_19_context_raw.npz	5773975	ok	
imu	14	170520	170520_va_14_motio_19_raw.npz	16983333	ok	
tof	14	170520	170520_va_14_motio_19_context_raw.npz	9207600	ok	
imu	14	170522	170522_va_14_motio_19_raw.npz	29106417	ok	
tof	14	170522	170522_va_14_motio_19_context_raw.npz	13363322	ok	
imu	14	170523	170523_va_14_motio_19_raw.npz	24109094	ok	
tof	14	170523	170523_va_14_motio_19_context_raw.npz	8764674	ok	
imu	14	170524	170524_va_14_motio_19_raw.npz	22248048	ok	
tof	14	170524	170524_va_14_motio_19_context_raw.npz	10881987	ok	
imu	14	170525	170525_va_14_motio_19_raw.npz	25502474	ok	
tof	14	170525	170525_va_14_motio_19_context_raw.npz	13277302	ok	
imu	14	170526	170526_va_14_motio_19_raw.npz	25132478	ok	
tof	14	170526	170526_va_14_motio_19_context_raw.npz	12338352	ok	
imu	14	170527	170527_va_14_motio_19_raw.npz	24998606	ok	
tof	14	170527	170527_va_14_motio_19_context_raw.npz	11727933	ok	
imu	14	170528	170528_va_14_motio_19_raw.npz	31213663	ok	
tof	14	170528	170528_va_14_motio_19_context_raw.npz	11531904	ok	
imu	14	170529	170529_va_14_motio_19_raw.npz	26996536	ok	
tof	14	170529	170529_va_14_motio_19_context_raw.npz	10445515	ok	
imu	14	170530	170530_va_14_motio_19_raw.npz	9528750	ok	
tof	14	170530	170530_va_14_motio_19_context_raw.npz	4534456	ok	
imu	14	170531	170531_va_14_motio_19_raw.npz	21903470	ok	
tof	14	170531	170531_va_14_motio_19_context_raw.npz	8433868	ok	
imu	14	170602	170602_va_14_motio_19_raw.npz	21429621	ok	
tof	14	170602	170602_va_14_motio_19_context_raw.npz	9862756	ok	
imu	14	170603	170603_va_14_motio_19_raw.npz	21493414	ok	
tof	14	170603	170603_va_14_motio_19_context_raw.npz	9116996	ok	
imu	14	170604	170604_va_14_motio_19_raw.npz	27339368	ok	
tof	14	170604	170604_va_14_motio_19_context_raw.npz	13547139	ok	
imu	14	170605	170605_va_14_motio_19_raw.npz	18225733	ok	
tof	14	170605	170605_va_14_motio_19_context_raw.npz	8759816	ok	
imu	14	170606	170606_va_14_motio_19_raw.npz	30505800	ok	
tof	14	170606	170606_va_14_motio_19_context_raw.npz	10341558	ok	
imu	14	170607	170607_va_14_motio_19_raw.npz	28262191	ok	
tof	14	170607	170607_va_14_motio_19_context_raw.npz	13956459	ok	
imu	14	170608	170608_va_14_motio_19_raw.npz	32116056	ok	
tof	14	170608	170608_va_14_motio_19_context_raw.npz	15360446	ok	
imu	14	170609	170609_va_14_motio_19_raw.npz	1560186	ok	
tof	14	170609	170609_va_14_motio_19_context_raw.npz	608365	ok	
imu	14	170612	170612_va_14_motio_19_raw.npz	7158205	ok	
tof	14	170612	170612_va_14_motio_19_context_raw.npz	4262220	ok	
imu	14	170613	170613_va_14_motio_19_raw.npz	22359370	ok	
tof	14	170613	170613_va_14_motio_19_context_raw.npz	13337403	ok	
imu	14	170614	170614_va_14_motio_19_raw.npz	24070569	ok	
tof	14	170614	170614_va_14_motio_19_context_raw.npz	11593044	ok	
imu	14	170615	170615_va_14_motio_19_raw.npz	27752575	ok	
tof	14	170615	170615_va_14_motio_19_context_raw.npz	13161778	ok	
imu	14	170616	170616_va_14_motio_19_raw.npz	25916404	ok	
tof	14	170616	170616_va_14_motio_19_context_raw.npz	9973438	ok	
imu	14	170617	170617_va_14_motio_19_raw.npz	18652393	ok	
tof	14	170617	170617_va_14_motio_19_context_raw.npz	10880426	ok	
imu	14	170620	170620_va_14_motio_19_raw.npz	19631788	ok	
tof	14	170620	170620_va_14_motio_19_context_raw.npz	8589964	ok	
imu	14	170621	170621_va_14_motio_19_raw.npz	21433085	ok	
tof	14	170621	170621_va_14_motio_19_context_raw.npz	8586327	ok	
imu	14	170622	170622_va_14_motio_19_raw.npz	23172129	ok	
tof	14	170622	170622_va_14_motio_19_context_raw.npz	10791743	ok	
imu	15	170420	170420_va_15_motio_20_raw.npz	10011381	ok	
tof	15	170420	170420_va_15_motio_20_context_raw.npz	4397382	ok	
imu	15	170425	170425_va_15_motio_20_raw.npz	28115363	ok	
tof	15	170425	170425_va_15_motio_20_context_raw.npz	5952632	ok	
imu	15	170427	170427_va_15_motio_20_raw.npz	3654486	ok	
tof	15	170427	170427_va_15_motio_20_context_raw.npz	4729710	ok	
imu	15	170502	170502_va_15_motio_20_raw.npz	11350603	ok	
tof	15	170502	170502_va_15_motio_20_context_raw.npz	3180735	ok	
imu	15	170511	170511_va_15_motio_20_raw.npz	11672051	ok	
tof	15	170511	170511_va_15_motio_20_context_raw.npz	5147607	ok	
imu	15	170514	170514_va_15_motio_20_raw.npz	23891949	ok	
tof	15	170514	170514_va_15_motio_20_context_raw.npz	5191239	ok	
imu	15	170518	170518_va_15_motio_20_raw.npz	11829938	ok	
tof	15	170518	170518_va_15_motio_20_context_raw.npz	5416546	ok	
imu	15	170519	170519_va_15_motio_20_raw.npz	11145726	ok	
tof	15	170519	170519_va_15_motio_20_context_raw.npz	5803638	ok	
imu	15	170520	170520_va_15_motio_20_raw.npz	11638234	ok	
tof	15	170520	170520_va_15_motio_20_context_raw.npz	3482021	ok	
imu	15	170526	170526_va_15_motio_20_raw.npz	12956009	ok	
tof	15	170526	170526_va_15_motio_20_context_raw.npz	4065071	ok	
imu	15	170602	170602_va_15_motio_20_raw.npz	13905661	ok	
tof	15	170602	170602_va_15_motio_20_context_raw.npz	6433792	ok	
imu	15	170604	170604_va_15_motio_20_raw.npz	15115535	ok	
tof	15	170604	170604_va_15_motio_20_context_raw.npz	766829	ok	
imu	15	170605	170605_va_15_motio_20_raw.npz	9594133	ok	
tof	15	170605	170605_va_15_motio_20_context_raw.npz	5001175	ok	

kind	va_id	day	member	declared_size_bytes	status	detail
imu	15	170608	170608_va_15_motio_20_raw.npz	12120624	ok	
tof	15	170608	170608_va_15_motio_20_context_raw.npz	7949564	ok	
imu	15	170609	170609_va_15_motio_20_raw.npz	12301775	ok	
tof	15	170609	170609_va_15_motio_20_context_raw.npz	5410614	ok	
imu	15	170611	170611_va_15_motio_20_raw.npz	11277986	ok	
tof	15	170611	170611_va_15_motio_20_context_raw.npz	7312463	ok	
imu	15	170613	170613_va_15_motio_20_raw.npz	11861546	ok	
tof	15	170613	170613_va_15_motio_20_context_raw.npz	8779333	ok	
imu	15	170614	170614_va_15_motio_20_raw.npz	12811824	ok	
tof	15	170614	170614_va_15_motio_20_context_raw.npz	5625946	ok	
imu	15	170620	170620_va_15_motio_20_raw.npz	12671944	ok	
tof	15	170620	170620_va_15_motio_20_context_raw.npz	3816217	ok	
imu	16	170421	170421_va_16_motio_13_raw.npz	7364972	ok	
tof	16	170421	170421_va_16_motio_13_context_raw.npz	2639426	ok	
imu	16	170423	170423_va_16_motio_13_raw.npz	13414935	ok	
tof	16	170423	170423_va_16_motio_13_context_raw.npz	4934992	ok	
imu	16	170424	170424_va_16_motio_13_raw.npz	20171336	ok	
tof	16	170424	170424_va_16_motio_13_context_raw.npz	6823450	ok	
imu	16	170426	170426_va_16_motio_13_raw.npz	21646188	ok	
tof	16	170426	170426_va_16_motio_13_context_raw.npz	7169667	ok	
imu	16	170427	170427_va_16_motio_13_raw.npz	17373226	ok	
tof	16	170427	170427_va_16_motio_13_context_raw.npz	4575761	ok	
imu	16	170428	170428_va_16_motio_13_raw.npz	17442243	ok	
tof	16	170428	170428_va_16_motio_13_context_raw.npz	5150954	ok	
imu	16	170429	170429_va_16_motio_13_raw.npz	17093105	ok	
tof	16	170429	170429_va_16_motio_13_context_raw.npz	4976342	ok	
imu	16	170430	170430_va_16_motio_13_raw.npz	6256579	ok	
tof	16	170430	170430_va_16_motio_13_context_raw.npz	3061262	ok	
imu	16	170501	170501_va_16_motio_13_raw.npz	14649885	ok	
tof	16	170501	170501_va_16_motio_13_context_raw.npz	4350335	ok	
imu	16	170502	170502_va_16_motio_13_raw.npz	8209894	ok	
tof	16	170502	170502_va_16_motio_13_context_raw.npz	2439092	ok	
imu	16	170510	170510_va_16_motio_13_raw.npz	12566924	ok	
tof	16	170510	170510_va_16_motio_13_context_raw.npz	4428443	ok	
imu	16	170511	170511_va_16_motio_13_raw.npz	22328790	ok	
tof	16	170511	170511_va_16_motio_13_context_raw.npz	8468090	ok	
imu	16	170512	170512_va_16_motio_13_raw.npz	18584346	ok	
tof	16	170512	170512_va_16_motio_13_context_raw.npz	8134453	ok	
imu	16	170513	170513_va_16_motio_13_raw.npz	10786683	ok	
tof	16	170513	170513_va_16_motio_13_context_raw.npz	3530377	ok	
imu	16	170514	170514_va_16_motio_13_raw.npz	16683741	ok	
tof	16	170514	170514_va_16_motio_13_context_raw.npz	4792596	ok	
imu	16	170515	170515_va_16_motio_13_raw.npz	16469830	ok	
tof	16	170515	170515_va_16_motio_13_context_raw.npz	6374794	ok	
imu	16	170516	170516_va_16_motio_13_raw.npz	15636820	ok	
tof	16	170516	170516_va_16_motio_13_context_raw.npz	5182963	ok	
imu	16	170517	170517_va_16_motio_13_raw.npz	6281472	ok	
tof	16	170517	170517_va_16_motio_13_context_raw.npz	2327876	ok	
imu	16	170518	170518_va_16_motio_13_raw.npz	13473114	ok	
tof	16	170518	170518_va_16_motio_13_context_raw.npz	2730777	ok	
imu	17	170428	170428_va_17_motio_10_raw.npz	16765440	ok	
tof	17	170428	170428_va_17_motio_10_context_raw.npz	1882877	ok	
imu	17	170430	170430_va_17_motio_10_raw.npz	14729315	ok	
tof	17	170430	170430_va_17_motio_10_context_raw.npz	3071982	ok	
imu	17	170502	170502_va_17_motio_10_raw.npz	15396836	ok	
tof	17	170502	170502_va_17_motio_10_context_raw.npz	1826090	ok	
imu	17	170503	170503_va_17_motio_10_raw.npz	17408289	ok	
tof	17	170503	170503_va_17_motio_10_context_raw.npz	2685291	ok	
imu	17	170504	170504_va_17_motio_10_raw.npz	3960000	ok	
tof	17	170504	170504_va_17_motio_10_context_raw.npz	1585342	ok	
imu	17	170509	170509_va_17_motio_10_raw.npz	152737	ok	
tof	17	170509	170509_va_17_motio_10_context_raw.npz	670031	ok	
imu	17	170510	170510_va_17_motio_10_raw.npz	4185874	ok	
tof	17	170510	170510_va_17_motio_10_context_raw.npz	2476564	ok	
imu	17	170511	170511_va_17_motio_10_raw.npz	2430955	ok	
tof	17	170511	170511_va_17_motio_10_context_raw.npz	1069208	ok	
imu	17	170512	170512_va_17_motio_10_raw.npz	4815480	ok	
tof	17	170512	170512_va_17_motio_10_context_raw.npz	1684934	ok	
imu	17	170514	170514_va_17_motio_10_raw.npz	23059069	ok	
tof	17	170514	170514_va_17_motio_10_context_raw.npz	6991191	ok	
imu	17	170516	170516_va_17_motio_10_raw.npz	9097309	ok	
tof	17	170516	170516_va_17_motio_10_context_raw.npz	2162032	ok	
imu	17	170517	170517_va_17_motio_10_raw.npz	5944949	ok	
tof	17	170517	170517_va_17_motio_10_context_raw.npz	2973473	ok	
imu	17	170518	170518_va_17_motio_10_raw.npz	14349792	ok	
tof	17	170518	170518_va_17_motio_10_context_raw.npz	2584351	ok	
imu	17	170520	170520_va_17_motio_10_raw.npz	6995144	ok	
tof	17	170520	170520_va_17_motio_10_context_raw.npz	1426512	ok	
imu	17	170521	170521_va_17_motio_10_raw.npz	22854519	ok	
tof	17	170521	170521_va_17_motio_10_context_raw.npz	6329741	ok	
imu	17	170522	170522_va_17_motio_10_raw.npz	11041417	ok	
tof	17	170522	170522_va_17_motio_10_context_raw.npz	2300327	ok	
imu	17	170523	170523_va_17_motio_10_raw.npz	4182190	ok	
tof	17	170523	170523_va_17_motio_10_context_raw.npz	1259012	ok	
imu	18	170427	170427_va_18_motio_11_raw.npz	8153563	ok	
tof	18	170427	170427_va_18_motio_11_context_raw.npz	3206946	ok	

kind	va_id	day	member	declared_size_bytes	status	detail
imu	18	170428	170428_va_18_motio_11_raw.npz	22308115	ok	
tof	18	170428	170428_va_18_motio_11_context_raw.npz	10586825	ok	
imu	18	170429	170429_va_18_motio_11_raw.npz	23193599	ok	
tof	18	170429	170429_va_18_motio_11_context_raw.npz	9869717	ok	
imu	18	170430	170430_va_18_motio_11_raw.npz	23022348	ok	
tof	18	170430	170430_va_18_motio_11_context_raw.npz	8549552	ok	
imu	18	170508	170508_va_18_motio_11_raw.npz	26203710	ok	
tof	18	170508	170508_va_18_motio_11_context_raw.npz	13301877	ok	
imu	18	170514	170514_va_18_motio_11_raw.npz	18462971	ok	
tof	18	170514	170514_va_18_motio_11_context_raw.npz	6624862	ok	
imu	18	170515	170515_va_18_motio_11_raw.npz	26499914	ok	
tof	18	170515	170515_va_18_motio_11_context_raw.npz	8163496	ok	
imu	18	170517	170517_va_18_motio_11_raw.npz	30840893	ok	
tof	18	170517	170517_va_18_motio_11_context_raw.npz	14568774	ok	
imu	18	170518	170518_va_18_motio_11_raw.npz	2246813	ok	
tof	18	170518	170518_va_18_motio_11_context_raw.npz	1381306	ok	
imu	18	170523	170523_va_18_motio_11_raw.npz	11681053	ok	
tof	18	170523	170523_va_18_motio_11_context_raw.npz	2256908	ok	
imu	18	170529	170529_va_18_motio_11_raw.npz	16535285	ok	
tof	18	170529	170529_va_18_motio_11_context_raw.npz	5646160	ok	
imu	18	170530	170530_va_18_motio_11_raw.npz	11013558	ok	
tof	18	170530	170530_va_18_motio_11_context_raw.npz	3842697	ok	
imu	18	170603	170603_va_18_motio_11_raw.npz	22401526	ok	
tof	18	170603	170603_va_18_motio_11_context_raw.npz	7578667	ok	
imu	18	170605	170605_va_18_motio_11_raw.npz	22048793	ok	
tof	18	170605	170605_va_18_motio_11_context_raw.npz	7254071	ok	
imu	18	170606	170606_va_18_motio_11_raw.npz	21909189	ok	
tof	18	170606	170606_va_18_motio_11_context_raw.npz	8915870	ok	
imu	18	170609	170609_va_18_motio_11_raw.npz	21634364	ok	
tof	18	170609	170609_va_18_motio_11_context_raw.npz	5284862	ok	
imu	18	170612	170612_va_18_motio_11_raw.npz	21385743	ok	
tof	18	170612	170612_va_18_motio_11_context_raw.npz	8852352	ok	
imu	18	170615	170615_va_18_motio_11_raw.npz	23471009	ok	
tof	18	170615	170615_va_18_motio_11_context_raw.npz	8233329	ok	
imu	18	170616	170616_va_18_motio_11_raw.npz	19933205	ok	
tof	18	170616	170616_va_18_motio_11_context_raw.npz	7906470	ok	
imu	18	170627	170627_va_18_motio_11_raw.npz	9518003	ok	
tof	18	170627	170627_va_18_motio_11_context_raw.npz	2251567	ok	
imu	19	170430	170430_va_19_motio_14_raw.npz	6695355	ok	
tof	19	170430	170430_va_19_motio_14_context_raw.npz	2254429	ok	
imu	19	170502	170502_va_19_motio_14_raw.npz	18835785	ok	
tof	19	170502	170502_va_19_motio_14_context_raw.npz	2447419	ok	
imu	19	170504	170504_va_19_motio_14_raw.npz	12623209	ok	
tof	19	170504	170504_va_19_motio_14_context_raw.npz	5545095	ok	
imu	19	170505	170505_va_19_motio_14_raw.npz	2748742	ok	
tof	19	170505	170505_va_19_motio_14_context_raw.npz	1769542	ok	
imu	19	170508	170508_va_19_motio_14_raw.npz	13628205	ok	
tof	19	170508	170508_va_19_motio_14_context_raw.npz	898160	ok	
imu	19	170510	170510_va_19_motio_14_raw.npz	7391413	ok	
tof	19	170510	170510_va_19_motio_14_context_raw.npz	203581	ok	
imu	19	170511	170511_va_19_motio_14_raw.npz	5736106	ok	
tof	19	170511	170511_va_19_motio_14_context_raw.npz	90333	ok	
imu	19	170514	170514_va_19_motio_14_raw.npz	26759505	ok	
tof	19	170514	170514_va_19_motio_14_context_raw.npz	14459936	ok	
imu	19	170524	170524_va_19_motio_14_raw.npz	21914298	ok	
tof	19	170524	170524_va_19_motio_14_context_raw.npz	11106342	ok	
imu	19	170606	170606_va_19_motio_14_raw.npz	13883807	ok	
tof	19	170606	170606_va_19_motio_14_context_raw.npz	1991919	ok	
imu	19	170607	170607_va_19_motio_14_raw.npz	5418243	ok	
tof	19	170607	170607_va_19_motio_14_context_raw.npz	1866691	ok	
imu	19	170609	170609_va_19_motio_14_raw.npz	28978484	ok	
tof	19	170609	170609_va_19_motio_14_context_raw.npz	16391882	ok	
imu	19	170612	170612_va_19_motio_14_raw.npz	4163266	ok	
tof	19	170612	170612_va_19_motio_14_context_raw.npz	1776027	ok	
imu	19	170615	170615_va_19_motio_14_raw.npz	20231704	ok	
tof	19	170615	170615_va_19_motio_14_context_raw.npz	10809726	ok	
imu	19	170628	170628_va_19_motio_14_raw.npz	29416190	ok	
tof	19	170628	170628_va_19_motio_14_context_raw.npz	16127634	ok	
imu	19	170629	170629_va_19_motio_14_raw.npz	8116804	ok	
tof	19	170629	170629_va_19_motio_14_context_raw.npz	3958813	ok	
imu	20	170503	170503_va_20_motio_16_raw.npz	9358744	ok	
tof	20	170503	170503_va_20_motio_16_context_raw.npz	2526314	ok	
imu	20	170504	170504_va_20_motio_16_raw.npz	10338753	ok	
tof	20	170504	170504_va_20_motio_16_context_raw.npz	10789354	ok	
imu	20	170510	170510_va_20_motio_16_raw.npz	6188538	ok	
tof	20	170510	170510_va_20_motio_16_context_raw.npz	2637668	ok	
imu	20	170512	170512_va_20_motio_16_raw.npz	7761862	ok	
tof	20	170512	170512_va_20_motio_16_context_raw.npz	3930479	ok	
imu	20	170515	170515_va_20_motio_16_raw.npz	2632726	ok	
tof	20	170515	170515_va_20_motio_16_context_raw.npz	1707179	ok	
imu	20	170516	170516_va_20_motio_16_raw.npz	10524956	ok	
tof	20	170516	170516_va_20_motio_16_context_raw.npz	3280021	ok	
imu	20	170530	170530_va_20_motio_16_raw.npz	15056382	ok	
tof	20	170530	170530_va_20_motio_16_context_raw.npz	7923457	ok	
imu	20	170531	170531_va_20_motio_16_raw.npz	18261350	ok	
tof	20	170531	170531_va_20_motio_16_context_raw.npz	10092027	ok	

kind	va_id	day	member	declared_size_bytes	status	detail
imu	20	170606	170606_va_20_motio_16_raw.npz	4978835	ok	
tof	20	170606	170606_va_20_motio_16_context_raw.npz	5234278	ok	
imu	20	170607	170607_va_20_motio_16_raw.npz	22069759	ok	
tof	20	170607	170607_va_20_motio_16_context_raw.npz	6282138	ok	
imu	20	170608	170608_va_20_motio_16_raw.npz	11756647	ok	
tof	20	170608	170608_va_20_motio_16_context_raw.npz	6587469	ok	
imu	20	170613	170613_va_20_motio_16_raw.npz	19222657	ok	
tof	20	170613	170613_va_20_motio_16_context_raw.npz	7524414	ok	
imu	20	170616	170616_va_20_motio_16_raw.npz	20837329	ok	
tof	20	170616	170616_va_20_motio_16_context_raw.npz	8381824	ok	
imu	20	170622	170622_va_20_motio_16_raw.npz	20734376	ok	
tof	20	170622	170622_va_20_motio_16_context_raw.npz	10203224	ok	
imu	20	170629	170629_va_20_motio_16_raw.npz	13350194	ok	
tof	20	170629	170629_va_20_motio_16_context_raw.npz	4911319	ok	
imu	20	170701	170701_va_20_motio_16_raw.npz	19955546	ok	
tof	20	170701	170701_va_20_motio_16_context_raw.npz	8403413	ok	
imu	20	170704	170704_va_20_motio_16_raw.npz	14442100	ok	
tof	20	170704	170704_va_20_motio_16_context_raw.npz	3615384	ok	
imu	20	170705	170705_va_20_motio_16_raw.npz	18570503	ok	
tof	20	170705	170705_va_20_motio_16_context_raw.npz	8380935	ok	
imu	20	170706	170706_va_20_motio_16_raw.npz	18787351	ok	
tof	20	170706	170706_va_20_motio_16_context_raw.npz	10962028	ok	
imu	21	170505	170505_va_21_motio_17_raw.npz	15762878	ok	
tof	21	170505	170505_va_21_motio_17_context_raw.npz	4356189	ok	
imu	21	170508	170508_va_21_motio_17_raw.npz	19134118	ok	
tof	21	170508	170508_va_21_motio_17_context_raw.npz	6875419	ok	
imu	21	170509	170509_va_21_motio_17_raw.npz	8470637	ok	
tof	21	170509	170509_va_21_motio_17_context_raw.npz	3976852	ok	
imu	21	170514	170514_va_21_motio_17_raw.npz	6540116	ok	
tof	21	170514	170514_va_21_motio_17_context_raw.npz	647455	ok	
imu	21	170517	170517_va_21_motio_17_raw.npz	9567409	ok	
tof	21	170517	170517_va_21_motio_17_context_raw.npz	3906002	ok	
imu	21	170518	170518_va_21_motio_17_raw.npz	16492137	ok	
tof	21	170518	170518_va_21_motio_17_context_raw.npz	5685387	ok	
imu	21	170519	170519_va_21_motio_17_raw.npz	18653862	ok	
tof	21	170519	170519_va_21_motio_17_context_raw.npz	5760425	ok	
imu	21	170521	170521_va_21_motio_17_raw.npz	20606153	ok	
tof	21	170521	170521_va_21_motio_17_context_raw.npz	6848275	ok	
imu	21	170522	170522_va_21_motio_17_raw.npz	7313594	ok	
tof	21	170522	170522_va_21_motio_17_context_raw.npz	3528565	ok	
imu	21	170523	170523_va_21_motio_17_raw.npz	12078942	ok	
tof	21	170523	170523_va_21_motio_17_context_raw.npz	5143508	ok	
imu	21	170526	170526_va_21_motio_17_raw.npz	3232607	ok	
tof	21	170526	170526_va_21_motio_17_context_raw.npz	1594828	ok	
imu	21	170529	170529_va_21_motio_17_raw.npz	13474709	ok	
tof	21	170529	170529_va_21_motio_17_context_raw.npz	3926916	ok	
imu	21	170530	170530_va_21_motio_17_raw.npz	9750412	ok	
tof	21	170530	170530_va_21_motio_17_context_raw.npz	3138188	ok	
imu	21	170531	170531_va_21_motio_17_raw.npz	19123339	ok	
tof	21	170531	170531_va_21_motio_17_context_raw.npz	5033456	ok	
imu	21	170601	170601_va_21_motio_17_raw.npz	12915885	ok	
tof	21	170601	170601_va_21_motio_17_context_raw.npz	4087579	ok	
imu	21	170602	170602_va_21_motio_17_raw.npz	12490460	ok	
tof	21	170602	170602_va_21_motio_17_context_raw.npz	4521222	ok	
imu	21	170603	170603_va_21_motio_17_raw.npz	20930085	ok	
tof	21	170603	170603_va_21_motio_17_context_raw.npz	3811775	ok	
imu	21	170606	170606_va_21_motio_17_raw.npz	17850990	ok	
tof	21	170606	170606_va_21_motio_17_context_raw.npz	7607637	ok	
imu	21	170608	170608_va_21_motio_17_raw.npz	15811759	ok	
tof	21	170608	170608_va_21_motio_17_context_raw.npz	5664384	ok	
imu	21	170609	170609_va_21_motio_17_raw.npz	16494714	ok	
tof	21	170609	170609_va_21_motio_17_context_raw.npz	3983601	ok	
imu	21	170610	170610_va_21_motio_17_raw.npz	20970673	ok	
tof	21	170610	170610_va_21_motio_17_context_raw.npz	7594576	ok	
imu	21	170612	170612_va_21_motio_17_raw.npz	15275152	ok	
tof	21	170612	170612_va_21_motio_17_context_raw.npz	6345457	ok	
imu	21	170613	170613_va_21_motio_17_raw.npz	11851830	ok	
tof	21	170613	170613_va_21_motio_17_context_raw.npz	5220791	ok	
imu	21	170615	170615_va_21_motio_17_raw.npz	9177649	ok	
tof	21	170615	170615_va_21_motio_17_context_raw.npz	4266449	ok	
imu	21	170616	170616_va_21_motio_17_raw.npz	20752060	ok	
tof	21	170616	170616_va_21_motio_17_context_raw.npz	5688114	ok	
imu	21	170617	170617_va_21_motio_17_raw.npz	6691589	ok	
tof	21	170617	170617_va_21_motio_17_context_raw.npz	2727881	ok	
imu	21	170627	170627_va_21_motio_17_raw.npz	228132	ok	
tof	21	170627	170627_va_21_motio_17_context_raw.npz	408194	ok	
imu	22	170508	170508_va_22_motio_15_raw.npz	10083860	ok	
tof	22	170508	170508_va_22_motio_15_context_raw.npz	5039917	ok	
imu	22	170511	170511_va_22_motio_15_raw.npz	17970283	ok	
tof	22	170511	170511_va_22_motio_15_context_raw.npz	9164167	ok	
imu	22	170512	170512_va_22_motio_15_raw.npz	13633990	ok	
tof	22	170512	170512_va_22_motio_15_context_raw.npz	10694904	ok	
imu	22	170515	170515_va_22_motio_15_raw.npz	20923613	ok	
tof	22	170515	170515_va_22_motio_15_context_raw.npz	13451313	ok	
imu	22	170516	170516_va_22_motio_15_raw.npz	25647846	ok	
tof	22	170516	170516_va_22_motio_15_context_raw.npz	13106852	ok	

kind	va_id	day	member	declared_size_bytes	status	detail
imu	22	170517	170517_va_22_motio_15_raw.npz	20959181	ok	
tof	22	170517	170517_va_22_motio_15_context_raw.npz	8953067	ok	
imu	22	170518	170518_va_22_motio_15_raw.npz	19058694	ok	
tof	22	170518	170518_va_22_motio_15_context_raw.npz	9659095	ok	
imu	22	170522	170522_va_22_motio_15_raw.npz	14588636	ok	
tof	22	170522	170522_va_22_motio_15_context_raw.npz	8698421	ok	
imu	22	170525	170525_va_22_motio_15_raw.npz	19841805	ok	
tof	22	170525	170525_va_22_motio_15_context_raw.npz	4353815	ok	
imu	22	170527	170527_va_22_motio_15_raw.npz	20793403	ok	
tof	22	170527	170527_va_22_motio_15_context_raw.npz	8303470	ok	
imu	22	170528	170528_va_22_motio_15_raw.npz	27383140	ok	
tof	22	170528	170528_va_22_motio_15_context_raw.npz	14344424	ok	
imu	22	170531	170531_va_22_motio_15_raw.npz	24605387	ok	
tof	22	170531	170531_va_22_motio_15_context_raw.npz	9851664	ok	
imu	22	170601	170601_va_22_motio_15_raw.npz	9565609	ok	
tof	22	170601	170601_va_22_motio_15_context_raw.npz	8459587	ok	
imu	22	170603	170603_va_22_motio_15_raw.npz	23901985	ok	
tof	22	170603	170603_va_22_motio_15_context_raw.npz	7585884	ok	
imu	22	170604	170604_va_22_motio_15_raw.npz	25749766	ok	
tof	22	170604	170604_va_22_motio_15_context_raw.npz	9378087	ok	
imu	22	170605	170605_va_22_motio_15_raw.npz	20562325	ok	
tof	22	170605	170605_va_22_motio_15_context_raw.npz	10194811	ok	
imu	22	170608	170608_va_22_motio_15_raw.npz	2990419	ok	
tof	22	170608	170608_va_22_motio_15_context_raw.npz	2130042	ok	
imu	22	170702	170702_va_22_motio_15_raw.npz	7431938	ok	
tof	22	170702	170702_va_22_motio_15_context_raw.npz	3774632	ok	
imu	22	170704	170704_va_22_motio_15_raw.npz	18731790	ok	
tof	22	170704	170704_va_22_motio_15_context_raw.npz	8281835	ok	
imu	22	170707	170707_va_22_motio_15_raw.npz	8780538	ok	
tof	22	170707	170707_va_22_motio_15_context_raw.npz	5196191	ok	
imu	23	171117	171117_va_23_motio_10_raw.npz	23208755	ok	
tof	23	171117	171117_va_23_motio_10_context_raw.npz	8275898	ok	
imu	23	171122	171122_va_23_motio_10_raw.npz	22214001	ok	
tof	23	171122	171122_va_23_motio_10_context_raw.npz	9108839	ok	
imu	23	171124	171124_va_23_motio_10_raw.npz	19521514	ok	
tof	23	171124	171124_va_23_motio_10_context_raw.npz	4468786	ok	
imu	23	171127	171127_va_23_motio_10_raw.npz	20141474	ok	
tof	23	171127	171127_va_23_motio_10_context_raw.npz	8792600	ok	
imu	23	171128	171128_va_23_motio_10_raw.npz	26205116	ok	
tof	23	171128	171128_va_23_motio_10_context_raw.npz	13762729	ok	
imu	23	171129	171129_va_23_motio_10_raw.npz	16240038	ok	
tof	23	171129	171129_va_23_motio_10_context_raw.npz	6720318	ok	
imu	23	171201	171201_va_23_motio_10_raw.npz	26916827	ok	
tof	23	171201	171201_va_23_motio_10_context_raw.npz	9549516	ok	
imu	23	171202	171202_va_23_motio_10_raw.npz	16533729	ok	
tof	23	171202	171202_va_23_motio_10_context_raw.npz	8665650	ok	
imu	23	171203	171203_va_23_motio_10_raw.npz	29420483	ok	
tof	23	171203	171203_va_23_motio_10_context_raw.npz	11225210	ok	
imu	23	171205	171205_va_23_motio_10_raw.npz	18925970	ok	
tof	23	171205	171205_va_23_motio_10_context_raw.npz	9341239	ok	
imu	23	171210	171210_va_23_motio_10_raw.npz	19462097	ok	
tof	23	171210	171210_va_23_motio_10_context_raw.npz	7688060	ok	
imu	23	171211	171211_va_23_motio_10_raw.npz	28991659	ok	
tof	23	171211	171211_va_23_motio_10_context_raw.npz	11934084	ok	
imu	23	171213	171213_va_23_motio_10_raw.npz	17953943	ok	
tof	23	171213	171213_va_23_motio_10_context_raw.npz	7813477	ok	
imu	23	171216	171216_va_23_motio_10_raw.npz	22911076	ok	
tof	23	171216	171216_va_23_motio_10_context_raw.npz	9666810	ok	
imu	23	171221	171221_va_23_motio_10_raw.npz	17427611	ok	
tof	23	171221	171221_va_23_motio_10_context_raw.npz	8398337	ok	
imu	23	171223	171223_va_23_motio_10_raw.npz	18013175	ok	
tof	23	171223	171223_va_23_motio_10_context_raw.npz	9157620	ok	
imu	23	171225	171225_va_23_motio_10_raw.npz	31437541	ok	
tof	23	171225	171225_va_23_motio_10_context_raw.npz	16339936	ok	
imu	23	171231	171231_va_23_motio_10_raw.npz	23091180	ok	
tof	23	171231	171231_va_23_motio_10_context_raw.npz	10815365	ok	
imu	23	180106	180106_va_23_motio_10_raw.npz	24425045	ok	
tof	23	180106	180106_va_23_motio_10_context_raw.npz	10755729	ok	
imu	23	180109	180109_va_23_motio_10_raw.npz	19692415	ok	
tof	23	180109	180109_va_23_motio_10_context_raw.npz	9654061	ok	
imu	23	180110	180110_va_23_motio_10_raw.npz	19469051	ok	
tof	23	180110	180110_va_23_motio_10_context_raw.npz	7543378	ok	
imu	24	171122	171122_va_24_motio_11_raw.npz	13621784	ok	
tof	24	171122	171122_va_24_motio_11_context_raw.npz	3087666	ok	
imu	24	171123	171123_va_24_motio_11_raw.npz	15099282	ok	
tof	24	171123	171123_va_24_motio_11_context_raw.npz	3757421	ok	
imu	24	171125	171125_va_24_motio_11_raw.npz	14715517	ok	
tof	24	171125	171125_va_24_motio_11_context_raw.npz	5843393	ok	
imu	24	171204	171204_va_24_motio_11_raw.npz	13660489	ok	
tof	24	171204	171204_va_24_motio_11_context_raw.npz	2974585	ok	
imu	24	171211	171211_va_24_motio_11_raw.npz	9347025	ok	
tof	24	171211	171211_va_24_motio_11_context_raw.npz	2450501	ok	
imu	24	171214	171214_va_24_motio_11_raw.npz	4197952	ok	
tof	24	171214	171214_va_24_motio_11_context_raw.npz	56777	ok	
imu	24	171220	171220_va_24_motio_11_raw.npz	28362285	ok	
tof	24	171220	171220_va_24_motio_11_context_raw.npz	6940124	ok	

kind	va_id	day	member	declared_size_bytes	status	detail
imu	24	180105	180105_va_24_motio_11_raw.npz	7409693	ok	
tof	24	180105	180105_va_24_motio_11_context_raw.npz	1054153	ok	
imu	24	180106	180106_va_24_motio_11_raw.npz	15745921	BAD_CRC	Bad CRC-32 for file 'ohsu-ms-fall-data/ms_ohsu_raw_imu_data_04152018/180106_va_24_motio_11_raw.npz'
tof	24	180106	180106_va_24_motio_11_context_raw.npz	2673097	ok	
imu	24	180107	180107_va_24_motio_11_raw.npz	5145619	ok	
tof	24	180107	180107_va_24_motio_11_context_raw.npz	314437	ok	
imu	24	180108	180108_va_24_motio_11_raw.npz	12776508	ok	
tof	24	180108	180108_va_24_motio_11_context_raw.npz	2826661	ok	
imu	24	180109	180109_va_24_motio_11_raw.npz	22690796	ok	
tof	24	180109	180109_va_24_motio_11_context_raw.npz	11664730	ok	
imu	24	180121	180121_va_24_motio_11_raw.npz	11423794	ok	
tof	24	180121	180121_va_24_motio_11_context_raw.npz	2886219	ok	
imu	24	180122	180122_va_24_motio_11_raw.npz	21887740	ok	
tof	24	180122	180122_va_24_motio_11_context_raw.npz	7126399	ok	
imu	24	180123	180123_va_24_motio_11_raw.npz	17151836	ok	
tof	24	180123	180123_va_24_motio_11_context_raw.npz	7912496	ok	
imu	24	180127	180127_va_24_motio_11_raw.npz	11872620	ok	
tof	24	180127	180127_va_24_motio_11_context_raw.npz	821585	ok	
imu	24	180128	180128_va_24_motio_11_raw.npz	9372270	ok	
tof	24	180128	180128_va_24_motio_11_context_raw.npz	1385496	ok	
imu	24	180129	180129_va_24_motio_11_raw.npz	8040477	ok	
tof	24	180129	180129_va_24_motio_11_context_raw.npz	166391	ok	
imu	24	180130	180130_va_24_motio_11_raw.npz	14075034	ok	
tof	24	180130	180130_va_24_motio_11_context_raw.npz	1637831	ok	
imu	25	171123	171123_va_25_motio_13_raw.npz	25766122	ok	
tof	25	171123	171123_va_25_motio_13_context_raw.npz	6080611	ok	
imu	25	171125	171125_va_25_motio_13_raw.npz	30461549	ok	
tof	25	171125	171125_va_25_motio_13_context_raw.npz	12008384	ok	
imu	25	171126	171126_va_25_motio_13_raw.npz	24925926	ok	
tof	25	171126	171126_va_25_motio_13_context_raw.npz	9072433	ok	
imu	25	171127	171127_va_25_motio_13_raw.npz	17270342	ok	
tof	25	171127	171127_va_25_motio_13_context_raw.npz	4133415	ok	
imu	25	171130	171130_va_25_motio_13_raw.npz	15362856	ok	
tof	25	171130	171130_va_25_motio_13_context_raw.npz	5541992	ok	
imu	25	171205	171205_va_25_motio_13_raw.npz	21692538	ok	
tof	25	171205	171205_va_25_motio_13_context_raw.npz	7573332	ok	
imu	25	171212	171212_va_25_motio_13_raw.npz	19341085	ok	
tof	25	171212	171212_va_25_motio_13_context_raw.npz	5969179	ok	
imu	25	171213	171213_va_25_motio_13_raw.npz	18970313	ok	
tof	25	171213	171213_va_25_motio_13_context_raw.npz	4832163	ok	
imu	25	171226	171226_va_25_motio_13_raw.npz	18667566	ok	
tof	25	171226	171226_va_25_motio_13_context_raw.npz	6675497	ok	
imu	25	171227	171227_va_25_motio_13_raw.npz	20117752	ok	
tof	25	171227	171227_va_25_motio_13_context_raw.npz	7340245	ok	
imu	25	171229	171229_va_25_motio_13_raw.npz	15152503	ok	
tof	25	171229	171229_va_25_motio_13_context_raw.npz	4938572	ok	
imu	25	180101	180101_va_25_motio_13_raw.npz	21768543	ok	
tof	25	180101	180101_va_25_motio_13_context_raw.npz	6938534	ok	
imu	25	180110	180110_va_25_motio_13_raw.npz	745814	ok	
tof	25	180110	180110_va_25_motio_13_context_raw.npz	404442	ok	
imu	25	180111	180111_va_25_motio_13_raw.npz	967567	ok	
tof	25	180111	180111_va_25_motio_13_context_raw.npz	519259	ok	
imu	25	180114	180114_va_25_motio_13_raw.npz	4417180	ok	
tof	25	180114	180114_va_25_motio_13_context_raw.npz	2438256	ok	
imu	25	180115	180115_va_25_motio_13_raw.npz	14422769	ok	
tof	25	180115	180115_va_25_motio_13_context_raw.npz	5666152	ok	
imu	25	180117	180117_va_25_motio_13_raw.npz	15160831	ok	
tof	25	180117	180117_va_25_motio_13_context_raw.npz	5808260	ok	
imu	25	180120	180120_va_25_motio_13_raw.npz	16223592	ok	
tof	25	180120	180120_va_25_motio_13_context_raw.npz	3845715	ok	
imu	25	180124	180124_va_25_motio_13_raw.npz	12082799	ok	
tof	25	180124	180124_va_25_motio_13_context_raw.npz	2228379	ok	
imu	26	171128	171128_va_26_motio_14_raw.npz	7334770	ok	
tof	26	171128	171128_va_26_motio_14_context_raw.npz	913885	ok	
imu	26	171201	171201_va_26_motio_14_raw.npz	6366551	ok	
tof	26	171201	171201_va_26_motio_14_context_raw.npz	986131	ok	
imu	26	171202	171202_va_26_motio_14_raw.npz	16175277	ok	
tof	26	171202	171202_va_26_motio_14_context_raw.npz	1958361	ok	
imu	26	171203	171203_va_26_motio_14_raw.npz	21367504	ok	
tof	26	171203	171203_va_26_motio_14_context_raw.npz	5524888	ok	
imu	26	171204	171204_va_26_motio_14_raw.npz	23597947	ok	
tof	26	171204	171204_va_26_motio_14_context_raw.npz	4044185	ok	
imu	26	171205	171205_va_26_motio_14_raw.npz	17435706	ok	
tof	26	171205	171205_va_26_motio_14_context_raw.npz	3752234	ok	
imu	26	171208	171208_va_26_motio_14_raw.npz	25372133	ok	
tof	26	171208	171208_va_26_motio_14_context_raw.npz	4189660	ok	
imu	26	171209	171209_va_26_motio_14_raw.npz	20125284	ok	
tof	26	171209	171209_va_26_motio_14_context_raw.npz	3368556	ok	
imu	26	171210	171210_va_26_motio_14_raw.npz	15240260	ok	
tof	26	171210	171210_va_26_motio_14_context_raw.npz	2946864	ok	

kind	va_id	day	member	declared_size_bytes	status	detail
imu	26	171211	171211_va_26_motio_14_raw.npz	14640248	ok	
tof	26	171211	171211_va_26_motio_14_context_raw.npz	1814525	ok	
imu	26	171215	171215_va_26_motio_14_raw.npz	21998571	ok	
tof	26	171215	171215_va_26_motio_14_context_raw.npz	3303035	ok	
imu	26	171216	171216_va_26_motio_14_raw.npz	13446925	ok	
tof	26	171216	171216_va_26_motio_14_context_raw.npz	4540227	ok	
imu	26	171217	171217_va_26_motio_14_raw.npz	33924879	ok	
tof	26	171217	171217_va_26_motio_14_context_raw.npz	9323445	ok	
imu	26	171219	171219_va_26_motio_14_raw.npz	6518575	ok	
tof	26	171219	171219_va_26_motio_14_context_raw.npz	1054941	ok	
imu	26	180103	180103_va_26_motio_14_raw.npz	16760821	ok	
tof	26	180103	180103_va_26_motio_14_context_raw.npz	2574907	ok	
imu	26	180104	180104_va_26_motio_14_raw.npz	12869185	ok	
tof	26	180104	180104_va_26_motio_14_context_raw.npz	686758	ok	
imu	26	180105	180105_va_26_motio_14_raw.npz	22139046	ok	
tof	26	180105	180105_va_26_motio_14_context_raw.npz	3770108	ok	
imu	26	180106	180106_va_26_motio_14_raw.npz	21055168	ok	
tof	26	180106	180106_va_26_motio_14_context_raw.npz	2988886	ok	
imu	26	180108	180108_va_26_motio_14_raw.npz	12737213	ok	
tof	26	180108	180108_va_26_motio_14_context_raw.npz	1751573	ok	
imu	26	180109	180109_va_26_motio_14_raw.npz	20132422	ok	
tof	26	180109	180109_va_26_motio_14_context_raw.npz	2909730	ok	
imu	26	180111	180111_va_26_motio_14_raw.npz	27443321	ok	
tof	26	180111	180111_va_26_motio_14_context_raw.npz	2954290	ok	
imu	26	180113	180113_va_26_motio_14_raw.npz	20215295	ok	
tof	26	180113	180113_va_26_motio_14_context_raw.npz	2420405	ok	
imu	26	180114	180114_va_26_motio_14_raw.npz	24468180	ok	
tof	26	180114	180114_va_26_motio_14_context_raw.npz	3615753	ok	
imu	26	180115	180115_va_26_motio_14_raw.npz	21237606	ok	
tof	26	180115	180115_va_26_motio_14_context_raw.npz	3450875	ok	
imu	26	180118	180118_va_26_motio_14_raw.npz	22186011	ok	
tof	26	180118	180118_va_26_motio_14_context_raw.npz	5557536	ok	
imu	26	180124	180124_va_26_motio_14_raw.npz	18474490	ok	
tof	26	180124	180124_va_26_motio_14_context_raw.npz	3433721	ok	
imu	26	180127	180127_va_26_motio_14_raw.npz	20580159	ok	
tof	26	180127	180127_va_26_motio_14_context_raw.npz	4042337	ok	
imu	26	180128	180128_va_26_motio_14_raw.npz	21524262	ok	
tof	26	180128	180128_va_26_motio_14_context_raw.npz	5379431	ok	
imu	26	180131	180131_va_26_motio_14_raw.npz	20921651	ok	
tof	26	180131	180131_va_26_motio_14_context_raw.npz	3764886	ok	
imu	26	180202	180202_va_26_motio_14_raw.npz	23032065	ok	
tof	26	180202	180202_va_26_motio_14_context_raw.npz	4055909	ok	
imu	26	180203	180203_va_26_motio_14_raw.npz	19360660	ok	
tof	26	180203	180203_va_26_motio_14_context_raw.npz	2738606	ok	
imu	26	180204	180204_va_26_motio_14_raw.npz	22191402	ok	
tof	26	180204	180204_va_26_motio_14_context_raw.npz	3148830	ok	
imu	26	180205	180205_va_26_motio_14_raw.npz	14911574	ok	
tof	26	180205	180205_va_26_motio_14_context_raw.npz	3616917	ok	
imu	26	180207	180207_va_26_motio_14_raw.npz	9657569	ok	
tof	26	180207	180207_va_26_motio_14_context_raw.npz	1193646	ok	
imu	28	171226	171226_va_28_motio_16_raw.npz	5000492	ok	
tof	28	171226	171226_va_28_motio_16_context_raw.npz	2432373	ok	
imu	28	171228	171228_va_28_motio_16_raw.npz	18177033	ok	
tof	28	171228	171228_va_28_motio_16_context_raw.npz	6884849	ok	
imu	28	171230	171230_va_28_motio_16_raw.npz	7279680	ok	
tof	28	171230	171230_va_28_motio_16_context_raw.npz	3184524	ok	
imu	28	171231	171231_va_28_motio_16_raw.npz	9229268	ok	
tof	28	171231	171231_va_28_motio_16_context_raw.npz	1725600	ok	
imu	28	180104	180104_va_28_motio_16_raw.npz	3218193	ok	
tof	28	180104	180104_va_28_motio_16_context_raw.npz	939727	ok	
imu	28	180112	180112_va_28_motio_16_raw.npz	22321965	ok	
tof	28	180112	180112_va_28_motio_16_context_raw.npz	8177874	ok	
imu	28	180113	180113_va_28_motio_16_raw.npz	7328285	ok	
tof	28	180113	180113_va_28_motio_16_context_raw.npz	2153248	ok	
imu	28	180117	180117_va_28_motio_16_raw.npz	19781575	ok	
tof	28	180117	180117_va_28_motio_16_context_raw.npz	8807240	ok	
imu	28	180119	180119_va_28_motio_16_raw.npz	19218630	ok	
tof	28	180119	180119_va_28_motio_16_context_raw.npz	7741716	ok	
imu	28	180123	180123_va_28_motio_16_raw.npz	13655959	ok	
tof	28	180123	180123_va_28_motio_16_context_raw.npz	4827434	ok	
imu	28	180124	180124_va_28_motio_16_raw.npz	15467266	ok	
tof	28	180124	180124_va_28_motio_16_context_raw.npz	4499074	ok	
imu	28	180125	180125_va_28_motio_16_raw.npz	16508723	ok	
tof	28	180125	180125_va_28_motio_16_context_raw.npz	5743081	ok	
imu	28	180128	180128_va_28_motio_16_raw.npz	482897	ok	
tof	28	180128	180128_va_28_motio_16_context_raw.npz	139940	ok	
imu	28	180202	180202_va_28_motio_16_raw.npz	22854691	ok	
tof	28	180202	180202_va_28_motio_16_context_raw.npz	3477008	ok	
imu	28	180207	180207_va_28_motio_16_raw.npz	11544600	ok	
tof	28	180207	180207_va_28_motio_16_context_raw.npz	4496203	ok	
imu	28	180208	180208_va_28_motio_16_raw.npz	11057377	ok	
tof	28	180208	180208_va_28_motio_16_context_raw.npz	4388414	ok	
imu	28	180209	180209_va_28_motio_16_raw.npz	14102087	ok	
tof	28	180209	180209_va_28_motio_16_context_raw.npz	2854181	ok	
imu	28	180210	180210_va_28_motio_16_raw.npz	20971556	ok	
tof	28	180210	180210_va_28_motio_16_context_raw.npz	3731879	ok	

kind	va_id	day	member	declared_size_bytes	status	detail
imu	28	180211	180211_va_28_motio_16_raw.npz	8817122	ok	
tof	28	180211	180211_va_28_motio_16_context_raw.npz	1738614	ok	
imu	28	180212	180212_va_28_motio_16_raw.npz	14581972	ok	
tof	28	180212	180212_va_28_motio_16_context_raw.npz	5276195	ok	
imu	28	180213	180213_va_28_motio_16_raw.npz	7097627	ok	
tof	28	180213	180213_va_28_motio_16_context_raw.npz	3701422	ok	
imu	30	171214	171214_va_30_motio_18_raw.npz	3647736	ok	
tof	30	171214	171214_va_30_motio_18_context_raw.npz	965055	ok	
imu	30	171215	171215_va_30_motio_18_raw.npz	5118810	ok	
tof	30	171215	171215_va_30_motio_18_context_raw.npz	3069643	ok	
imu	30	171219	171219_va_30_motio_18_raw.npz	19252189	ok	
tof	30	171219	171219_va_30_motio_18_context_raw.npz	6513095	ok	
imu	30	171221	171221_va_30_motio_18_raw.npz	19536706	ok	
tof	30	171221	171221_va_30_motio_18_context_raw.npz	7619952	ok	
imu	30	171223	171223_va_30_motio_18_raw.npz	20506969	ok	
tof	30	171223	171223_va_30_motio_18_context_raw.npz	9530789	ok	
imu	30	171224	171224_va_30_motio_18_raw.npz	18376273	ok	
tof	30	171224	171224_va_30_motio_18_context_raw.npz	10539004	ok	
imu	30	171226	171226_va_30_motio_18_raw.npz	4064373	ok	
tof	30	171226	171226_va_30_motio_18_context_raw.npz	1719484	ok	
imu	30	171230	171230_va_30_motio_18_raw.npz	13961344	ok	
tof	30	171230	171230_va_30_motio_18_context_raw.npz	7075828	ok	
imu	30	180116	180116_va_30_motio_18_raw.npz	28392803	ok	
tof	30	180116	180116_va_30_motio_18_context_raw.npz	10213460	ok	
imu	30	180118	180118_va_30_motio_18_raw.npz	16294589	ok	
tof	30	180118	180118_va_30_motio_18_context_raw.npz	1677002	ok	
imu	30	180119	180119_va_30_motio_18_raw.npz	15857960	ok	
tof	30	180119	180119_va_30_motio_18_context_raw.npz	8282233	ok	
imu	30	180121	180121_va_30_motio_18_raw.npz	15462680	ok	
tof	30	180121	180121_va_30_motio_18_context_raw.npz	5267433	ok	
imu	30	180126	180126_va_30_motio_18_raw.npz	29121457	ok	
tof	30	180126	180126_va_30_motio_18_context_raw.npz	14688077	ok	
imu	30	180204	180204_va_30_motio_18_raw.npz	8791637	ok	
tof	30	180204	180204_va_30_motio_18_context_raw.npz	2276705	ok	
imu	30	180206	180206_va_30_motio_18_raw.npz	20692185	ok	
tof	30	180206	180206_va_30_motio_18_context_raw.npz	4301416	ok	
imu	30	180214	180214_va_30_motio_18_raw.npz	10527121	ok	
tof	30	180214	180214_va_30_motio_18_context_raw.npz	1605071	ok	
imu	30	180215	180215_va_30_motio_18_raw.npz	14266796	ok	
tof	30	180215	180215_va_30_motio_18_context_raw.npz	285217	ok	
imu	30	180217	180217_va_30_motio_18_raw.npz	4869245	ok	
tof	30	180217	180217_va_30_motio_18_context_raw.npz	2742	ok	
imu	30	180219	180219_va_30_motio_18_raw.npz	28488879	ok	
tof	30	180219	180219_va_30_motio_18_context_raw.npz	13480601	ok	
imu	33	171219	171219_va_33_motio_17_raw.npz	2331685	ok	
tof	33	171219	171219_va_33_motio_17_context_raw.npz	822824	ok	
imu	33	171220	171220_va_33_motio_17_raw.npz	24048304	ok	
tof	33	171220	171220_va_33_motio_17_context_raw.npz	8873085	ok	
imu	33	171221	171221_va_33_motio_17_raw.npz	17215538	ok	
tof	33	171221	171221_va_33_motio_17_context_raw.npz	4088498	ok	
imu	33	171225	171225_va_33_motio_17_raw.npz	11022605	ok	
tof	33	171225	171225_va_33_motio_17_context_raw.npz	3597561	ok	
imu	33	171227	171227_va_33_motio_17_raw.npz	18059017	ok	
tof	33	171227	171227_va_33_motio_17_context_raw.npz	8323283	ok	
imu	33	171231	171231_va_33_motio_17_raw.npz	15704951	ok	
tof	33	171231	171231_va_33_motio_17_context_raw.npz	3557464	ok	
imu	33	180102	180102_va_33_motio_17_raw.npz	13941987	ok	
tof	33	180102	180102_va_33_motio_17_context_raw.npz	7424111	ok	
imu	33	180103	180103_va_33_motio_17_raw.npz	19061851	ok	
tof	33	180103	180103_va_33_motio_17_context_raw.npz	6871647	ok	
imu	33	180104	180104_va_33_motio_17_raw.npz	16926064	ok	
tof	33	180104	180104_va_33_motio_17_context_raw.npz	4095861	ok	
imu	33	180108	180108_va_33_motio_17_raw.npz	19772462	ok	
tof	33	180108	180108_va_33_motio_17_context_raw.npz	8967287	ok	
imu	33	180111	180111_va_33_motio_17_raw.npz	17115228	ok	
tof	33	180111	180111_va_33_motio_17_context_raw.npz	5914719	ok	
imu	33	180114	180114_va_33_motio_17_raw.npz	19463886	ok	
tof	33	180114	180114_va_33_motio_17_context_raw.npz	7266622	ok	
imu	33	180116	180116_va_33_motio_17_raw.npz	15927136	ok	
tof	33	180116	180116_va_33_motio_17_context_raw.npz	4029576	ok	
imu	33	180117	180117_va_33_motio_17_raw.npz	21356045	ok	
tof	33	180117	180117_va_33_motio_17_context_raw.npz	7476033	ok	
imu	33	180122	180122_va_33_motio_17_raw.npz	18689844	ok	
tof	33	180122	180122_va_33_motio_17_context_raw.npz	5663650	ok	
imu	33	180124	180124_va_33_motio_17_raw.npz	15574112	ok	
tof	33	180124	180124_va_33_motio_17_context_raw.npz	6576814	ok	
imu	33	180125	180125_va_33_motio_17_raw.npz	18755164	ok	
tof	33	180125	180125_va_33_motio_17_context_raw.npz	8656839	ok	
imu	33	180127	180127_va_33_motio_17_raw.npz	6854067	ok	
tof	33	180127	180127_va_33_motio_17_context_raw.npz	2037555	ok	
imu	33	180128	180128_va_33_motio_17_raw.npz	14344400	ok	
tof	33	180128	180128_va_33_motio_17_context_raw.npz	3726441	ok	
imu	33	180131	180131_va_33_motio_17_raw.npz	13022315	ok	
tof	33	180131	180131_va_33_motio_17_context_raw.npz	5519433	ok	
imu	33	180201	180201_va_33_motio_17_raw.npz	12766696	ok	
tof	33	180201	180201_va_33_motio_17_context_raw.npz	5095779	ok	

kind	va_id	day	member	declared_size_bytes	status	detail
imu	33	180202	180202_va_33_motio_17_raw.npz	15386686	ok	
tof	33	180202	180202_va_33_motio_17_context_raw.npz	5134143	ok	
imu	33	180204	180204_va_33_motio_17_raw.npz	10947938	ok	
tof	33	180204	180204_va_33_motio_17_context_raw.npz	6089770	ok	
imu	33	180205	180205_va_33_motio_17_raw.npz	20119957	ok	
tof	33	180205	180205_va_33_motio_17_context_raw.npz	8363983	ok	
imu	33	180207	180207_va_33_motio_17_raw.npz	24585512	ok	
tof	33	180207	180207_va_33_motio_17_context_raw.npz	7723624	ok	
imu	33	180210	180210_va_33_motio_17_raw.npz	17479849	ok	
tof	33	180210	180210_va_33_motio_17_context_raw.npz	6154273	ok	
imu	33	180213	180213_va_33_motio_17_raw.npz	16866895	ok	
tof	33	180213	180213_va_33_motio_17_context_raw.npz	2676853	ok	
imu	33	180215	180215_va_33_motio_17_raw.npz	20732496	ok	
tof	33	180215	180215_va_33_motio_17_context_raw.npz	4226021	ok	
imu	33	180217	180217_va_33_motio_17_raw.npz	3562308	ok	
tof	33	180217	180217_va_33_motio_17_context_raw.npz	2401	ok	
imu	33	180221	180221_va_33_motio_17_raw.npz	20486972	ok	
tof	33	180221	180221_va_33_motio_17_context_raw.npz	8255494	ok	
imu	33	180228	180228_va_33_motio_17_raw.npz	15775413	ok	
tof	33	180228	180228_va_33_motio_17_context_raw.npz	5541582	ok	
imu	33	180301	180301_va_33_motio_17_raw.npz	17163713	ok	
tof	33	180301	180301_va_33_motio_17_context_raw.npz	7660845	ok	
imu	33	180302	180302_va_33_motio_17_raw.npz	9210818	ok	
tof	33	180302	180302_va_33_motio_17_context_raw.npz	4800907	ok	
imu	4	161008	161008_va_4_motio_11_raw.npz	19823300	ok	
tof	4	161008	161008_va_4_motio_11_context_raw.npz	6633177	ok	
imu	4	161011	161011_va_4_motio_11_raw.npz	1250945	ok	
tof	4	161011	161011_va_4_motio_11_context_raw.npz	468991	ok	
imu	4	161012	161012_va_4_motio_11_raw.npz	23061276	ok	
tof	4	161012	161012_va_4_motio_11_context_raw.npz	2132803	ok	
imu	4	161014	161014_va_4_motio_11_raw.npz	18825998	ok	
tof	4	161014	161014_va_4_motio_11_context_raw.npz	6230580	ok	
imu	4	161015	161015_va_4_motio_11_raw.npz	36456468	ok	
tof	4	161015	161015_va_4_motio_11_context_raw.npz	14014909	ok	
imu	4	161016	161016_va_4_motio_11_raw.npz	23083819	ok	
tof	4	161016	161016_va_4_motio_11_context_raw.npz	5309012	ok	
imu	4	161017	161017_va_4_motio_11_raw.npz	12980244	ok	
tof	4	161017	161017_va_4_motio_11_context_raw.npz	3858868	ok	
imu	4	161020	161020_va_4_motio_11_raw.npz	8742571	ok	
tof	4	161020	161020_va_4_motio_11_context_raw.npz	1012105	ok	
imu	4	161021	161021_va_4_motio_11_raw.npz	8804175	ok	
tof	4	161021	161021_va_4_motio_11_context_raw.npz	4117080	ok	
imu	4	161022	161022_va_4_motio_11_raw.npz	14289074	ok	
tof	4	161022	161022_va_4_motio_11_context_raw.npz	4746756	ok	
imu	4	161025	161025_va_4_motio_11_raw.npz	15166381	ok	
tof	4	161025	161025_va_4_motio_11_context_raw.npz	912226	ok	
imu	4	161026	161026_va_4_motio_11_raw.npz	24403573	ok	
tof	4	161026	161026_va_4_motio_11_context_raw.npz	3891183	ok	
imu	4	161029	161029_va_4_motio_11_raw.npz	17491043	ok	
tof	4	161029	161029_va_4_motio_11_context_raw.npz	2828852	ok	
imu	4	161030	161030_va_4_motio_11_raw.npz	17426640	ok	
tof	4	161030	161030_va_4_motio_11_context_raw.npz	3110212	ok	
imu	4	161103	161103_va_4_motio_11_raw.npz	17694668	ok	
tof	4	161103	161103_va_4_motio_11_context_raw.npz	4257335	ok	
imu	4	161104	161104_va_4_motio_11_raw.npz	14942012	ok	
tof	4	161104	161104_va_4_motio_11_context_raw.npz	3386711	ok	
imu	4	161106	161106_va_4_motio_11_raw.npz	8746819	ok	
tof	4	161106	161106_va_4_motio_11_context_raw.npz	1927211	ok	
imu	4	161107	161107_va_4_motio_11_raw.npz	6303452	ok	
tof	4	161107	161107_va_4_motio_11_context_raw.npz	107883	ok	
imu	5	161014	161014_va_5_motio_15_raw.npz	1731739	ok	
tof	5	161014	161014_va_5_motio_15_context_raw.npz	40824	ok	
imu	5	161015	161015_va_5_motio_15_raw.npz	20992535	ok	
tof	5	161015	161015_va_5_motio_15_context_raw.npz	438974	ok	
imu	5	161018	161018_va_5_motio_15_raw.npz	18706559	ok	
tof	5	161018	161018_va_5_motio_15_context_raw.npz	794756	ok	
imu	5	161020	161020_va_5_motio_15_raw.npz	19043565	ok	
tof	5	161020	161020_va_5_motio_15_context_raw.npz	348876	ok	
imu	5	161021	161021_va_5_motio_15_raw.npz	19357995	ok	
tof	5	161021	161021_va_5_motio_15_context_raw.npz	574206	ok	
imu	5	161028	161028_va_5_motio_15_raw.npz	21537119	ok	
tof	5	161028	161028_va_5_motio_15_context_raw.npz	699901	ok	
imu	5	161030	161030_va_5_motio_15_raw.npz	21668146	ok	
tof	5	161030	161030_va_5_motio_15_context_raw.npz	1212916	ok	
imu	5	161101	161101_va_5_motio_15_raw.npz	18754461	ok	
tof	5	161101	161101_va_5_motio_15_context_raw.npz	1209070	ok	
imu	5	161102	161102_va_5_motio_15_raw.npz	19129430	ok	
tof	5	161102	161102_va_5_motio_15_context_raw.npz	1084620	ok	
imu	5	161104	161104_va_5_motio_15_raw.npz	13858634	ok	
tof	5	161104	161104_va_5_motio_15_context_raw.npz	491967	ok	
imu	5	161105	161105_va_5_motio_15_raw.npz	12247774	ok	
tof	5	161105	161105_va_5_motio_15_context_raw.npz	453886	ok	
imu	5	161106	161106_va_5_motio_15_raw.npz	17782724	ok	
tof	5	161106	161106_va_5_motio_15_context_raw.npz	807631	ok	
imu	5	161113	161113_va_5_motio_15_raw.npz	16988928	ok	
tof	5	161113	161113_va_5_motio_15_context_raw.npz	1337602	ok	

kind	va_id	day	member	declared_size_bytes	status	detail
imu	5	161116	161116__va__5__motio__15__raw.npz	18140208	ok	
tof	5	161116	161116__va__5__motio__15__context__raw.npz	810666	ok	
imu	5	161119	161119__va__5__motio__15__raw.npz	12200373	ok	
tof	5	161119	161119__va__5__motio__15__context__raw.npz	668094	ok	
imu	5	161124	161124__va__5__motio__15__raw.npz	11250936	ok	
tof	5	161124	161124__va__5__motio__15__context__raw.npz	410508	ok	
imu	5	161126	161126__va__5__motio__15__raw.npz	4489069	ok	
tof	5	161126	161126__va__5__motio__15__context__raw.npz	28382	ok	
imu	5	161128	161128__va__5__motio__15__raw.npz	8040070	ok	
tof	5	161128	161128__va__5__motio__15__context__raw.npz	528921	ok	
imu	5	161202	161202__va__5__motio__15__raw.npz	20119004	ok	
tof	5	161202	161202__va__5__motio__15__context__raw.npz	571942	ok	
imu	5	161203	161203__va__5__motio__15__raw.npz	18247548	ok	
tof	5	161203	161203__va__5__motio__15__context__raw.npz	2370682	ok	
imu	6	161014	161014__va__6__motio__14__raw.npz	21480962	ok	
tof	6	161014	161014__va__6__motio__14__context__raw.npz	2942674	ok	
imu	6	161015	161015__va__6__motio__14__raw.npz	4265870	ok	
tof	6	161015	161015__va__6__motio__14__context__raw.npz	1410019	ok	
imu	6	161017	161017__va__6__motio__14__raw.npz	9039775	ok	
tof	6	161017	161017__va__6__motio__14__context__raw.npz	2093274	ok	
imu	6	161018	161018__va__6__motio__14__raw.npz	16692421	ok	
tof	6	161018	161018__va__6__motio__14__context__raw.npz	2315697	ok	
imu	6	161019	161019__va__6__motio__14__raw.npz	29325378	ok	
tof	6	161019	161019__va__6__motio__14__context__raw.npz	6339673	ok	
imu	6	161020	161020__va__6__motio__14__raw.npz	21795526	ok	
tof	6	161020	161020__va__6__motio__14__context__raw.npz	3051399	ok	
imu	6	161021	161021__va__6__motio__14__raw.npz	19863558	ok	
tof	6	161021	161021__va__6__motio__14__context__raw.npz	3058919	ok	
imu	6	161022	161022__va__6__motio__14__raw.npz	2185305	ok	
tof	6	161022	161022__va__6__motio__14__context__raw.npz	182068	ok	
imu	6	161024	161024__va__6__motio__14__raw.npz	11532813	ok	
tof	6	161024	161024__va__6__motio__14__context__raw.npz	1740120	ok	
imu	6	161025	161025__va__6__motio__14__raw.npz	23538633	ok	
tof	6	161025	161025__va__6__motio__14__context__raw.npz	10246091	ok	
imu	6	161026	161026__va__6__motio__14__raw.npz	1305454	ok	
tof	6	161026	161026__va__6__motio__14__context__raw.npz	146346	ok	
imu	6	161027	161027__va__6__motio__14__raw.npz	21137487	ok	
tof	6	161027	161027__va__6__motio__14__context__raw.npz	782158	ok	
imu	6	161028	161028__va__6__motio__14__raw.npz	9282397	ok	
tof	6	161028	161028__va__6__motio__14__context__raw.npz	2256577	ok	
imu	6	161029	161029__va__6__motio__14__raw.npz	18636878	ok	
tof	6	161029	161029__va__6__motio__14__context__raw.npz	5018312	ok	
imu	6	161030	161030__va__6__motio__14__raw.npz	17238708	ok	
tof	6	161030	161030__va__6__motio__14__context__raw.npz	3960289	ok	
imu	6	161031	161031__va__6__motio__14__raw.npz	16990936	ok	
tof	6	161031	161031__va__6__motio__14__context__raw.npz	2485735	ok	
imu	6	161101	161101__va__6__motio__14__raw.npz	11756440	ok	
tof	6	161101	161101__va__6__motio__14__context__raw.npz	2349234	ok	
imu	6	161102	161102__va__6__motio__14__raw.npz	12916156	ok	
tof	6	161102	161102__va__6__motio__14__context__raw.npz	5208394	ok	
imu	6	161103	161103__va__6__motio__14__raw.npz	17716124	ok	
tof	6	161103	161103__va__6__motio__14__context__raw.npz	3641925	ok	
imu	6	161104	161104__va__6__motio__14__raw.npz	17571980	ok	
tof	6	161104	161104__va__6__motio__14__context__raw.npz	6290610	ok	
imu	6	161105	161105__va__6__motio__14__raw.npz	13347768	ok	
tof	6	161105	161105__va__6__motio__14__context__raw.npz	1687202	ok	
imu	6	161106	161106__va__6__motio__14__raw.npz	16652886	ok	
tof	6	161106	161106__va__6__motio__14__context__raw.npz	4429774	ok	
imu	6	161107	161107__va__6__motio__14__raw.npz	19246181	ok	
tof	6	161107	161107__va__6__motio__14__context__raw.npz	5958030	ok	
imu	6	161108	161108__va__6__motio__14__raw.npz	7599224	ok	
tof	6	161108	161108__va__6__motio__14__context__raw.npz	5645846	ok	
imu	6	161109	161109__va__6__motio__14__raw.npz	23028813	ok	
tof	6	161109	161109__va__6__motio__14__context__raw.npz	9195257	ok	
imu	6	161110	161110__va__6__motio__14__raw.npz	18734503	ok	
tof	6	161110	161110__va__6__motio__14__context__raw.npz	4229332	ok	
imu	6	161111	161111__va__6__motio__14__raw.npz	16886353	ok	
tof	6	161111	161111__va__6__motio__14__context__raw.npz	5196819	ok	
imu	6	161112	161112__va__6__motio__14__raw.npz	227539	ok	
tof	6	161112	161112__va__6__motio__14__context__raw.npz	2758111	ok	
imu	6	161113	161113__va__6__motio__14__raw.npz	19493694	ok	
tof	6	161113	161113__va__6__motio__14__context__raw.npz	2413250	ok	
imu	6	161114	161114__va__6__motio__14__raw.npz	9031834	ok	
tof	6	161114	161114__va__6__motio__14__context__raw.npz	1373527	ok	
imu	6	161115	161115__va__6__motio__14__raw.npz	8979250	ok	
tof	6	161115	161115__va__6__motio__14__context__raw.npz	4603430	ok	
imu	6	161116	161116__va__6__motio__14__raw.npz	16237157	ok	
tof	6	161116	161116__va__6__motio__14__context__raw.npz	4173018	ok	
imu	6	161117	161117__va__6__motio__14__raw.npz	11086342	ok	
tof	6	161117	161117__va__6__motio__14__context__raw.npz	1382658	ok	
imu	6	161118	161118__va__6__motio__14__raw.npz	8512937	ok	
tof	6	161118	161118__va__6__motio__14__context__raw.npz	2451287	ok	
imu	6	161119	161119__va__6__motio__14__raw.npz	6470523	ok	
tof	6	161119	161119__va__6__motio__14__context__raw.npz	168463	ok	
imu	6	161120	161120__va__6__motio__14__raw.npz	1251568	ok	
tof	6	161120	161120__va__6__motio__14__context__raw.npz	892095	ok	

kind	va_id	day	member	declared_size_bytes	status	detail
imu	6	161122	161122_va_6_motio_14_raw.npz	10155105	ok	
tof	6	161122	161122_va_6_motio_14_context_raw.npz	4671671	ok	
imu	6	161123	161123_va_6_motio_14_raw.npz	4120659	ok	
tof	6	161123	161123_va_6_motio_14_context_raw.npz	2687875	ok	
imu	6	161124	161124_va_6_motio_14_raw.npz	5139580	ok	
tof	6	161124	161124_va_6_motio_14_context_raw.npz	308454	ok	
imu	6	161125	161125_va_6_motio_14_raw.npz	6046069	ok	
tof	6	161125	161125_va_6_motio_14_context_raw.npz	1234751	ok	
imu	6	161129	161129_va_6_motio_14_raw.npz	2571632	ok	
tof	6	161129	161129_va_6_motio_14_context_raw.npz	244800	ok	
imu	6	161130	161130_va_6_motio_14_raw.npz	8609063	ok	
tof	6	161130	161130_va_6_motio_14_context_raw.npz	43202	ok	
imu	6	161201	161201_va_6_motio_14_raw.npz	10301538	ok	
tof	6	161201	161201_va_6_motio_14_context_raw.npz	615918	ok	
imu	6	161206	161206_va_6_motio_14_raw.npz	772938	ok	
tof	6	161206	161206_va_6_motio_14_context_raw.npz	576543	ok	
imu	6	161207	161207_va_6_motio_14_raw.npz	13958545	ok	
tof	6	161207	161207_va_6_motio_14_context_raw.npz	2014455	ok	
imu	6	161211	161211_va_6_motio_14_raw.npz	8340041	ok	
tof	6	161211	161211_va_6_motio_14_context_raw.npz	74752	ok	
imu	6	161212	161212_va_6_motio_14_raw.npz	3712553	ok	
tof	6	161212	161212_va_6_motio_14_context_raw.npz	68096	ok	
imu	6	161213	161213_va_6_motio_14_raw.npz	11456918	ok	
tof	6	161213	161213_va_6_motio_14_context_raw.npz	263041	ok	
imu	7	161025	161025_va_7_motio_16_raw.npz	22940396	ok	
imu	7	161101	161101_va_7_motio_16_raw.npz	10544357	ok	
imu	7	161105	161105_va_7_motio_16_raw.npz	20534651	ok	
imu	7	161114	161114_va_7_motio_16_raw.npz	14712456	ok	
imu	7	161115	161115_va_7_motio_16_raw.npz	9879300	ok	
imu	7	161116	161116_va_7_motio_16_raw.npz	7525157	ok	
imu	7	161117	161117_va_7_motio_16_raw.npz	20442240	ok	
imu	7	161120	161120_va_7_motio_16_raw.npz	2331585	ok	
imu	7	161121	161121_va_7_motio_16_raw.npz	13288614	ok	
imu	7	161122	161122_va_7_motio_16_raw.npz	14249802	ok	
imu	7	161125	161125_va_7_motio_16_raw.npz	11440771	ok	
imu	7	161202	161202_va_7_motio_16_raw.npz	17432598	ok	
imu	7	161205	161205_va_7_motio_16_raw.npz	10433160	ok	
imu	7	161209	161209_va_7_motio_16_raw.npz	8489280	ok	
imu	7	161211	161211_va_7_motio_16_raw.npz	23787691	ok	
imu	7	161212	161212_va_7_motio_16_raw.npz	15599984	ok	
imu	7	161220	161220_va_7_motio_16_raw.npz	1379762	ok	
imu	8	161027	161027_va_8_motio_17_raw.npz	23658377	ok	
tof	8	161027	161027_va_8_motio_17_context_raw.npz	3621695	ok	
imu	8	161028	161028_va_8_motio_17_raw.npz	25408955	ok	
tof	8	161028	161028_va_8_motio_17_context_raw.npz	8991324	ok	
imu	8	161102	161102_va_8_motio_17_raw.npz	18552222	ok	
tof	8	161102	161102_va_8_motio_17_context_raw.npz	3450960	ok	
imu	8	161104	161104_va_8_motio_17_raw.npz	16718364	ok	
tof	8	161104	161104_va_8_motio_17_context_raw.npz	8028123	ok	
imu	8	161105	161105_va_8_motio_17_raw.npz	19215755	ok	
tof	8	161105	161105_va_8_motio_17_context_raw.npz	6584479	ok	
imu	8	161108	161108_va_8_motio_17_raw.npz	16517060	ok	
tof	8	161108	161108_va_8_motio_17_context_raw.npz	5849834	ok	
imu	8	161111	161111_va_8_motio_17_raw.npz	18522092	ok	
tof	8	161111	161111_va_8_motio_17_context_raw.npz	10321680	ok	
imu	8	161112	161112_va_8_motio_17_raw.npz	14908420	ok	
tof	8	161112	161112_va_8_motio_17_context_raw.npz	7675646	ok	
imu	8	161115	161115_va_8_motio_17_raw.npz	10097774	ok	
tof	8	161115	161115_va_8_motio_17_context_raw.npz	4460177	ok	
imu	8	161117	161117_va_8_motio_17_raw.npz	3867975	ok	
tof	8	161117	161117_va_8_motio_17_context_raw.npz	2550721	ok	
imu	8	161123	161123_va_8_motio_17_raw.npz	9527203	ok	
tof	8	161123	161123_va_8_motio_17_context_raw.npz	5317020	ok	
imu	8	161125	161125_va_8_motio_17_raw.npz	20471630	ok	
tof	8	161125	161125_va_8_motio_17_context_raw.npz	10164301	ok	
imu	8	161126	161126_va_8_motio_17_raw.npz	18829612	ok	
tof	8	161126	161126_va_8_motio_17_context_raw.npz	9927016	ok	
imu	8	161128	161128_va_8_motio_17_raw.npz	5982337	ok	
tof	8	161128	161128_va_8_motio_17_context_raw.npz	2018133	ok	
imu	8	161129	161129_va_8_motio_17_raw.npz	23545762	ok	
tof	8	161129	161129_va_8_motio_17_context_raw.npz	9490128	ok	
imu	8	161203	161203_va_8_motio_17_raw.npz	13472751	ok	
tof	8	161203	161203_va_8_motio_17_context_raw.npz	4358777	ok	
imu	8	161207	161207_va_8_motio_17_raw.npz	16782871	ok	
tof	8	161207	161207_va_8_motio_17_context_raw.npz	7518353	ok	
imu	9	161027	161027_va_9_motio_18_raw.npz	2554080	ok	
tof	9	161027	161027_va_9_motio_18_context_raw.npz	1395943	ok	
imu	9	161028	161028_va_9_motio_18_raw.npz	18306234	ok	
tof	9	161028	161028_va_9_motio_18_context_raw.npz	5252286	ok	
imu	9	161029	161029_va_9_motio_18_raw.npz	4587447	ok	
tof	9	161029	161029_va_9_motio_18_context_raw.npz	2202955	ok	
imu	9	161031	161031_va_9_motio_18_raw.npz	24445641	ok	
tof	9	161031	161031_va_9_motio_18_context_raw.npz	9533154	ok	
imu	9	161101	161101_va_9_motio_18_raw.npz	18745537	ok	
tof	9	161101	161101_va_9_motio_18_context_raw.npz	6580695	ok	
imu	9	161104	161104_va_9_motio_18_raw.npz	18119436	ok	

kind	va_id	day	member	declared_size_bytes	status	detail
tof	9	161104	161104_va_9_motio_18_context_raw.npz	7612969	ok	
imu	9	161110	161110_va_9_motio_18_raw.npz	22997522	ok	
tof	9	161110	161110_va_9_motio_18_context_raw.npz	6087092	ok	
imu	9	161111	161111_va_9_motio_18_raw.npz	21544615	ok	
tof	9	161111	161111_va_9_motio_18_context_raw.npz	7398472	ok	
imu	9	161112	161112_va_9_motio_18_raw.npz	482719	ok	
tof	9	161112	161112_va_9_motio_18_context_raw.npz	8704014	ok	
imu	9	161113	161113_va_9_motio_18_raw.npz	21746035	ok	
tof	9	161113	161113_va_9_motio_18_context_raw.npz	10214880	ok	
imu	9	161114	161114_va_9_motio_18_raw.npz	21166930	ok	
tof	9	161114	161114_va_9_motio_18_context_raw.npz	5814001	ok	
imu	9	161115	161115_va_9_motio_18_raw.npz	23324269	ok	
tof	9	161115	161115_va_9_motio_18_context_raw.npz	3952088	ok	
imu	9	161116	161116_va_9_motio_18_raw.npz	17125634	ok	
tof	9	161116	161116_va_9_motio_18_context_raw.npz	6100727	ok	
imu	9	161120	161120_va_9_motio_18_raw.npz	22346971	ok	
tof	9	161120	161120_va_9_motio_18_context_raw.npz	9000447	ok	
imu	9	161122	161122_va_9_motio_18_raw.npz	24270746	ok	
tof	9	161122	161122_va_9_motio_18_context_raw.npz	8610731	ok	
imu	9	161123	161123_va_9_motio_18_raw.npz	12550782	ok	
tof	9	161123	161123_va_9_motio_18_context_raw.npz	6303825	ok	
imu	9	161124	161124_va_9_motio_18_raw.npz	16816340	ok	
tof	9	161124	161124_va_9_motio_18_context_raw.npz	7043087	ok	
imu	9	161125	161125_va_9_motio_18_raw.npz	19695178	ok	
tof	9	161125	161125_va_9_motio_18_context_raw.npz	10853819	ok	
imu	9	161127	161127_va_9_motio_18_raw.npz	12338163	ok	
tof	9	161127	161127_va_9_motio_18_context_raw.npz	5878633	ok	
imu	9	161128	161128_va_9_motio_18_raw.npz	19368055	ok	
tof	9	161128	161128_va_9_motio_18_context_raw.npz	6402426	ok	
imu	9	161130	161130_va_9_motio_18_raw.npz	17853655	ok	
tof	9	161130	161130_va_9_motio_18_context_raw.npz	8447996	ok	
imu	9	161201	161201_va_9_motio_18_raw.npz	17435056	ok	
tof	9	161201	161201_va_9_motio_18_context_raw.npz	4733678	ok	
imu	9	161202	161202_va_9_motio_18_raw.npz	18599073	ok	
tof	9	161202	161202_va_9_motio_18_context_raw.npz	5901234	ok	
imu	9	161207	161207_va_9_motio_18_raw.npz	21303402	ok	
tof	9	161207	161207_va_9_motio_18_context_raw.npz	8342657	ok	
imu	9	161208	161208_va_9_motio_18_raw.npz	17626312	ok	
tof	9	161208	161208_va_9_motio_18_context_raw.npz	6958213	ok	
imu	9	161209	161209_va_9_motio_18_raw.npz	23679593	ok	
tof	9	161209	161209_va_9_motio_18_context_raw.npz	5777347	ok	
imu	9	161211	161211_va_9_motio_18_raw.npz	20768963	ok	
tof	9	161211	161211_va_9_motio_18_context_raw.npz	7490627	ok	
imu	9	161214	161214_va_9_motio_18_raw.npz	6744633	ok	
tof	9	161214	161214_va_9_motio_18_context_raw.npz	1990498	ok	
imu	9	161218	161218_va_9_motio_18_raw.npz	16373150	ok	
tof	9	161218	161218_va_9_motio_18_context_raw.npz	6526703	ok	
imu	9	161221	161221_va_9_motio_18_raw.npz	16689587	ok	
tof	9	161221	161221_va_9_motio_18_context_raw.npz	6411402	ok	
imu	9	161222	161222_va_9_motio_18_raw.npz	20224253	ok	
tof	9	161222	161222_va_9_motio_18_context_raw.npz	10321297	ok	

Table S2 note. A member that fails verification cannot be read. Windows that depend on it carry no samples and are removed by the coverage gate, and the loss is counted as a data-integrity exclusion.

Table S3. Duplicate-event report: fall records sharing participant and timestamp that were collapsed as exact duplicates, with the number of excess rows removed (4 affected records). (source: `table_duplicate_report.csv`).

va_id	ts_fall_utc	n_rows	n_excess_rows
6	1478745482	3	2
6	1479009643	2	1
6	1481667773	2	1
11	1483483987	2	1

Table S4. Participant-level calibration audit: median window-mean acceleration magnitude per participant, the implied scale factor, and the exclusion verdict (Section 2.5). A participant whose median falls outside 0.8 to 1.3 g is excluded whole; 1 of 26 participants were excluded on this criterion. (source: `table_calibration_audit.csv`).

va_id	median_accel_mag_mean_g	implied_scale_factor	within_expected_1g_range	verdict
4	4.057	4.06	False	EXCLUDED: record not on a 1 g scale
5	0.9986	1	True	included
6	1.002	1	True	included
7	0.9998	1	True	included
8	1.003	1	True	included
9	0.996	1	True	included
10	1.005	1	True	included

va_id	median_accel_mag_mean_g	implied_scale_factor	within_expected_1g_range	verdict
11	1.006	1.01	True	included
12	1.008	1.01	True	included
13	1.012	1.01	True	included
14	1.005	1.01	True	included
15	1.012	1.01	True	included
16	1.006	1.01	True	included
17	1.012	1.01	True	included
18	0.9956	1	True	included
19	0.9922	0.99	True	included
20	0.9956	1	True	included
21	1.007	1.01	True	included
22	1.002	1	True	included
23	1.013	1.01	True	included
24	1.026	1.03	True	included
25	1.003	1	True	included
26	1.011	1.01	True	included
28	1.001	1	True	included
30	0.9999	1	True	included
33	1.003	1	True	included

Table S4 note. The audit is applied per participant because an off-scale record is off-scale throughout, not in isolated windows.

Table S5. Windows removed by the calibration exclusion: every candidate window of the participant excluded in Table S4, with its window-mean acceleration magnitude (72 windows). (source: `table_artifact_windows_excluded.csv`).

window_id	va_id	event_id	is_case	length_label	accel_mag_mean	reason
NEG_va4_r01_primary_5min	4	NEG_va4	0	primary_5min	4.071	participant excluded: record not on a 1 g scale (median window-mean magnitude outside 0.8-1.3 g)
NEG_va4_r01_alt_2min	4	NEG_va4	0	alt_2min	4.072	participant excluded: record not on a 1 g scale (median window-mean magnitude outside 0.8-1.3 g)
NEG_va4_r01_alt_8min	4	NEG_va4	0	alt_8min	4.071	participant excluded: record not on a 1 g scale (median window-mean magnitude outside 0.8-1.3 g)
NEG_va4_r08_primary_5min	4	NEG_va4	0	primary_5min		participant excluded: record not on a 1 g scale (median window-mean magnitude outside 0.8-1.3 g)
NEG_va4_r08_alt_2min	4	NEG_va4	0	alt_2min		participant excluded: record not on a 1 g scale (median window-mean magnitude outside 0.8-1.3 g)
NEG_va4_r08_alt_8min	4	NEG_va4	0	alt_8min		participant excluded: record not on a 1 g scale (median window-mean magnitude outside 0.8-1.3 g)
NEG_va4_r22_primary_5min	4	NEG_va4	0	primary_5min		participant excluded: record not on a 1 g scale (median window-mean magnitude outside 0.8-1.3 g)
NEG_va4_r22_alt_2min	4	NEG_va4	0	alt_2min		participant excluded: record not on a 1 g scale (median window-mean magnitude outside 0.8-1.3 g)
NEG_va4_r22_alt_8min	4	NEG_va4	0	alt_8min		participant excluded: record not on a 1 g scale (median window-mean magnitude outside 0.8-1.3 g)
NEG_va4_r16_primary_5min	4	NEG_va4	0	primary_5min	4.084	participant excluded: record not on a 1 g scale (median window-mean magnitude outside 0.8-1.3 g)
NEG_va4_r16_alt_2min	4	NEG_va4	0	alt_2min	4.159	participant excluded: record not on a 1 g scale (median window-mean magnitude outside 0.8-1.3 g)
NEG_va4_r16_alt_8min	4	NEG_va4	0	alt_8min	4.106	participant excluded: record not on a 1 g scale (median window-mean magnitude outside 0.8-1.3 g)
NEG_va4_r18_primary_5min	4	NEG_va4	0	primary_5min		participant excluded: record not on a 1 g scale (median window-mean magnitude outside 0.8-1.3 g)
NEG_va4_r18_alt_2min	4	NEG_va4	0	alt_2min		participant excluded: record not on a 1 g scale (median window-mean magnitude outside 0.8-1.3 g)
NEG_va4_r18_alt_8min	4	NEG_va4	0	alt_8min		participant excluded: record not on a 1 g scale (median window-mean magnitude outside 0.8-1.3 g)
NEG_va4_r20_primary_5min	4	NEG_va4	0	primary_5min		participant excluded: record not on a 1 g scale (median window-mean magnitude outside 0.8-1.3 g)
NEG_va4_r20_alt_2min	4	NEG_va4	0	alt_2min		participant excluded: record not on a 1 g scale (median window-mean magnitude outside 0.8-1.3 g)
NEG_va4_r20_alt_8min	4	NEG_va4	0	alt_8min		participant excluded: record not on a 1 g scale (median window-mean magnitude outside 0.8-1.3 g)
NEG_va4_r03_primary_5min	4	NEG_va4	0	primary_5min	4.076	participant excluded: record not on a 1 g scale (median window-mean magnitude outside 0.8-1.3 g)
NEG_va4_r03_alt_2min	4	NEG_va4	0	alt_2min	4.02	participant excluded: record not on a 1 g scale (median window-mean magnitude outside 0.8-1.3 g)
NEG_va4_r03_alt_8min	4	NEG_va4	0	alt_8min	4.07	participant excluded: record not on a 1 g scale (median window-mean magnitude outside 0.8-1.3 g)
NEG_va4_r10_primary_5min	4	NEG_va4	0	primary_5min	4.023	participant excluded: record not on a 1 g scale (median window-mean magnitude outside 0.8-1.3 g)
NEG_va4_r10_alt_2min	4	NEG_va4	0	alt_2min	4.023	participant excluded: record not on a 1 g scale (median window-mean magnitude outside 0.8-1.3 g)
NEG_va4_r10_alt_8min	4	NEG_va4	0	alt_8min	4.023	participant excluded: record not on a 1 g scale (median window-mean magnitude outside 0.8-1.3 g)
NEG_va4_r23_primary_5min	4	NEG_va4	0	primary_5min		participant excluded: record not on a 1 g scale (median window-mean magnitude outside 0.8-1.3 g)
NEG_va4_r23_alt_2min	4	NEG_va4	0	alt_2min		participant excluded: record not on a 1 g scale (median window-mean magnitude outside 0.8-1.3 g)
NEG_va4_r23_alt_8min	4	NEG_va4	0	alt_8min		participant excluded: record not on a 1 g scale (median window-mean magnitude outside 0.8-1.3 g)
NEG_va4_r15_primary_5min	4	NEG_va4	0	primary_5min		participant excluded: record not on a 1 g scale (median window-mean magnitude outside 0.8-1.3 g)
NEG_va4_r15_alt_2min	4	NEG_va4	0	alt_2min		participant excluded: record not on a 1 g scale (median window-mean magnitude outside 0.8-1.3 g)
NEG_va4_r15_alt_8min	4	NEG_va4	0	alt_8min		participant excluded: record not on a 1 g scale (median window-mean magnitude outside 0.8-1.3 g)
NEG_va4_r06_primary_5min	4	NEG_va4	0	primary_5min		participant excluded: record not on a 1 g scale (median window-mean magnitude outside 0.8-1.3 g)
NEG_va4_r06_alt_2min	4	NEG_va4	0	alt_2min		participant excluded: record not on a 1 g scale (median window-mean magnitude outside 0.8-1.3 g)
NEG_va4_r06_alt_8min	4	NEG_va4	0	alt_8min		participant excluded: record not on a 1 g scale (median window-mean magnitude outside 0.8-1.3 g)
NEG_va4_r11_primary_5min	4	NEG_va4	0	primary_5min	4.008	participant excluded: record not on a 1 g scale (median window-mean magnitude outside 0.8-1.3 g)
NEG_va4_r11_alt_2min	4	NEG_va4	0	alt_2min	3.957	participant excluded: record not on a 1 g scale (median window-mean magnitude outside 0.8-1.3 g)
NEG_va4_r11_alt_8min	4	NEG_va4	0	alt_8min	4.034	participant excluded: record not on a 1 g scale (median window-mean magnitude outside 0.8-1.3 g)
NEG_va4_r14_primary_5min	4	NEG_va4	0	primary_5min		participant excluded: record not on a 1 g scale (median window-mean magnitude outside 0.8-1.3 g)
NEG_va4_r14_alt_2min	4	NEG_va4	0	alt_2min		participant excluded: record not on a 1 g scale (median window-mean magnitude outside 0.8-1.3 g)
NEG_va4_r14_alt_8min	4	NEG_va4	0	alt_8min		participant excluded: record not on a 1 g scale (median window-mean magnitude outside 0.8-1.3 g)
NEG_va4_r12_primary_5min	4	NEG_va4	0	primary_5min		participant excluded: record not on a 1 g scale (median window-mean magnitude outside 0.8-1.3 g)
NEG_va4_r12_alt_2min	4	NEG_va4	0	alt_2min		participant excluded: record not on a 1 g scale (median window-mean magnitude outside 0.8-1.3 g)
NEG_va4_r12_alt_8min	4	NEG_va4	0	alt_8min		participant excluded: record not on a 1 g scale (median window-mean magnitude outside 0.8-1.3 g)
NEG_va4_r13_primary_5min	4	NEG_va4	0	primary_5min		participant excluded: record not on a 1 g scale (median window-mean magnitude outside 0.8-1.3 g)
NEG_va4_r13_alt_2min	4	NEG_va4	0	alt_2min		participant excluded: record not on a 1 g scale (median window-mean magnitude outside 0.8-1.3 g)
NEG_va4_r13_alt_8min	4	NEG_va4	0	alt_8min		participant excluded: record not on a 1 g scale (median window-mean magnitude outside 0.8-1.3 g)
NEG_va4_r09_primary_5min	4	NEG_va4	0	primary_5min		participant excluded: record not on a 1 g scale (median window-mean magnitude outside 0.8-1.3 g)
NEG_va4_r09_alt_2min	4	NEG_va4	0	alt_2min		participant excluded: record not on a 1 g scale (median window-mean magnitude outside 0.8-1.3 g)
NEG_va4_r09_alt_8min	4	NEG_va4	0	alt_8min		participant excluded: record not on a 1 g scale (median window-mean magnitude outside 0.8-1.3 g)
NEG_va4_r21_primary_5min	4	NEG_va4	0	primary_5min		participant excluded: record not on a 1 g scale (median window-mean magnitude outside 0.8-1.3 g)
NEG_va4_r21_alt_2min	4	NEG_va4	0	alt_2min		participant excluded: record not on a 1 g scale (median window-mean magnitude outside 0.8-1.3 g)
NEG_va4_r21_alt_8min	4	NEG_va4	0	alt_8min		participant excluded: record not on a 1 g scale (median window-mean magnitude outside 0.8-1.3 g)
NEG_va4_r00_primary_5min	4	NEG_va4	0	primary_5min		participant excluded: record not on a 1 g scale (median window-mean magnitude outside 0.8-1.3 g)
NEG_va4_r00_alt_2min	4	NEG_va4	0	alt_2min		participant excluded: record not on a 1 g scale (median window-mean magnitude outside 0.8-1.3 g)
NEG_va4_r00_alt_8min	4	NEG_va4	0	alt_8min		participant excluded: record not on a 1 g scale (median window-mean magnitude outside 0.8-1.3 g)

window_id	va_id	event_id	is_case	length_label	accel_mag_mean	reason
NEG_va4_r17_primary_5min	4	NEG_va4	0	primary_5min		participant excluded: record not on a 1 g scale (median window-mean magnitude outside 0.8-1.3 g)
NEG_va4_r17_alt_2min	4	NEG_va4	0	alt_2min		participant excluded: record not on a 1 g scale (median window-mean magnitude outside 0.8-1.3 g)
NEG_va4_r17_alt_8min	4	NEG_va4	0	alt_8min		participant excluded: record not on a 1 g scale (median window-mean magnitude outside 0.8-1.3 g)
NEG_va4_r04_primary_5min	4	NEG_va4	0	primary_5min		participant excluded: record not on a 1 g scale (median window-mean magnitude outside 0.8-1.3 g)
NEG_va4_r04_alt_2min	4	NEG_va4	0	alt_2min		participant excluded: record not on a 1 g scale (median window-mean magnitude outside 0.8-1.3 g)
NEG_va4_r04_alt_8min	4	NEG_va4	0	alt_8min		participant excluded: record not on a 1 g scale (median window-mean magnitude outside 0.8-1.3 g)
NEG_va4_r05_primary_5min	4	NEG_va4	0	primary_5min	4.059	participant excluded: record not on a 1 g scale (median window-mean magnitude outside 0.8-1.3 g)
NEG_va4_r05_alt_2min	4	NEG_va4	0	alt_2min	4.059	participant excluded: record not on a 1 g scale (median window-mean magnitude outside 0.8-1.3 g)
NEG_va4_r05_alt_8min	4	NEG_va4	0	alt_8min	4.057	participant excluded: record not on a 1 g scale (median window-mean magnitude outside 0.8-1.3 g)
NEG_va4_r07_primary_5min	4	NEG_va4	0	primary_5min		participant excluded: record not on a 1 g scale (median window-mean magnitude outside 0.8-1.3 g)
NEG_va4_r07_alt_2min	4	NEG_va4	0	alt_2min		participant excluded: record not on a 1 g scale (median window-mean magnitude outside 0.8-1.3 g)
NEG_va4_r07_alt_8min	4	NEG_va4	0	alt_8min		participant excluded: record not on a 1 g scale (median window-mean magnitude outside 0.8-1.3 g)
NEG_va4_r19_primary_5min	4	NEG_va4	0	primary_5min		participant excluded: record not on a 1 g scale (median window-mean magnitude outside 0.8-1.3 g)
NEG_va4_r19_alt_2min	4	NEG_va4	0	alt_2min		participant excluded: record not on a 1 g scale (median window-mean magnitude outside 0.8-1.3 g)
NEG_va4_r19_alt_8min	4	NEG_va4	0	alt_8min		participant excluded: record not on a 1 g scale (median window-mean magnitude outside 0.8-1.3 g)
NEG_va4_r02_primary_5min	4	NEG_va4	0	primary_5min	4.019	participant excluded: record not on a 1 g scale (median window-mean magnitude outside 0.8-1.3 g)
NEG_va4_r02_alt_2min	4	NEG_va4	0	alt_2min	4.022	participant excluded: record not on a 1 g scale (median window-mean magnitude outside 0.8-1.3 g)
NEG_va4_r02_alt_8min	4	NEG_va4	0	alt_8min	4.016	participant excluded: record not on a 1 g scale (median window-mean magnitude outside 0.8-1.3 g)

Table S6. Window-extraction summary: candidate windows extracted and participants covered, across every window specification (Section 2.3). (source: `table_features_candidate.csv`).

quantity	value
candidate windows extracted (all lengths and lags)	7525
window specifications	7
participants with extracted windows	26
rows with IMU coverage ≥ 0.9	5168
rows with ToF coverage ≥ 0.9	3995
rows with any ToF samples	5626
archive members unreadable (checksum failure)	1

Table S6 note. Counts are computed from the candidate window table; the coverage figures and the archive-integrity count come from the summary written by the extraction step.

Table S7. Candidate window index: every candidate window across every window specification, including the four lag designs, before feature computation (Section 2.3). The full table (7525 rows, 15 columns) is sheet `window_index` of Supplementary File S3; the column dictionary below gives each column's type, completeness and range (source: `table_windows_candidate.csv`).

Column	Type	N present	N missing	Mean	Min	Max
window_id	text/id	7525	0	7525 distinct values		
event_id	text/id	7525	0	63 distinct values		
va_id	numeric	7525	0	13.46	4	33
is_case	numeric	7525	0	0.04558	0	1
length_label	text/id	7525	0	7 distinct values		
length_s	numeric	7525	0	300	120	480
lag_s	numeric	7525	0	27.22	0	120
window_start_utc	numeric	7525	0	1.49e+09	1.476e+09	1.52e+09
window_end_utc	numeric	7525	0	1.49e+09	1.476e+09	1.52e+09
anchor_fall_ts	numeric	6517	1008	1.489e+09	1.478e+09	1.518e+09
clock_hour_utc	numeric	7525	0	10.52	0	23
control_day	numeric	7182	343	1.664e+05	161000	180300
control_rank	numeric	7182	343	8.921	0	23
shift_days	numeric	7182	343	2.755	-64	68
has_tof_day	text/id	7182	343	2 distinct values		

Table S8. Candidate window feature table: the IMU and ToF features (Section 2.4) for every candidate window, before coverage filtering and referent matching, with the per-window sampling-interval diagnostics. The full table (7525 rows, 46 columns) is sheet `candidate_features` of Supplementary File S3; the column dictionary below gives each column's type, completeness and range (source: `table_features_candidate.csv`).

Column	Type	N present	N missing	Mean	Min	Max
window_id	text/id	7525	0	7525 distinct values		
event_id	text/id	7525	0	63 distinct values		
va_id	numeric	7525	0	13.46	4	33
is_case	numeric	7525	0	0.04558	0	1
length_label	text/id	7525	0	7 distinct values		
length_s	numeric	7525	0	300	120	480
lag_s	numeric	7525	0	27.22	0	120
window_start_utc	numeric	7525	0	1.49e+09	1.476e+09	1.52e+09
window_end_utc	numeric	7525	0	1.49e+09	1.476e+09	1.52e+09
anchor_fall_ts	numeric	6517	1008	1.489e+09	1.478e+09	1.518e+09
clock_hour_utc	numeric	7525	0	10.52	0	23
control_day	numeric	7182	343	1.664e+05	161000	180300
control_rank	numeric	7182	343	8.921	0	23
shift_days	numeric	7182	343	2.755	-64	68
has_tof_day	text/id	7182	343	2 distinct values		
day_start	numeric	7525	0	1.664e+05	161000	180300
day_end	numeric	7525	0	1.664e+05	161000	180300
va_id_str	numeric	7525	0	13.46	4	33

Column	Type	N present	N missing	Mean	Min	Max
imu_n_samples	numeric	7525	0	1.062e+04	0	30640
imu_coverage_frac	numeric	7525	0	0.7089	0	1.427
imu_dt_median_s	numeric	5337	2188	0.02	0.02	0.02
imu_dt_min_s	numeric	5337	2188	0.009119	0	0.01999
imu_dt_frac_below_ half_nominal	numeric	5337	2188	0.00264	0	0.3
imu_dt_frac_nonpositive	numeric	5337	2188	0.002124	0	0.2991
jerk_max_observed_ interval	numeric	5331	2194	37	0.2837	436
resampled_valid_frac	numeric	5337	2188	0.9891	0.5005	1
accel_mag_mean	numeric	5331	2194	1.016	0.4691	4.159
accel_mag_sd	numeric	5331	2194	0.04001	0.0008481	0.9349
accel_mag_iqr	numeric	5331	2194	0.01474	0.001134	0.9037
accel_mag_fluctuation_ rms	numeric	5331	2194	0.04001	0.0008481	0.9349
accel_dynamic_rms_ gravity_removed	numeric	5331	2194	0.06826	0.001691	1.486
jerk_mean	numeric	5331	2194	0.5624	0.03415	14.57
jerk_sd	numeric	5331	2194	1.325	0.04282	31.34
jerk_max	numeric	5331	2194	29.21	0.1621	377.5
dom_freq_hz	numeric	5337	2188	3.109	0.375	10
spectral_entropy	numeric	5331	2194	0.9387	0.5921	0.9992
gait_band_power_frac	numeric	5331	2194	0.3491	0.01857	0.888
posture_transition_rate	numeric	5337	2188	0.7845	0	22
tof_n_samples	numeric	7525	0	675.6	0	2176
tof_coverage_frac	numeric	7525	0	0.5632	0	1.515
tof_speed_mean	numeric	4246	3279	0.1616	0	2.461
tof_speed_sd	numeric	4246	3279	0.1461	0	2
tof_path_length	numeric	4246	3279	38.12	0	293.9
tof_net_displacement	numeric	4246	3279	1.967	0	16.7
tof_tortuosity	numeric	2820	4705	37.94	1	417.7
tof_room_transition_rate	numeric	5615	1910	0.5509	0	19.12

Column	Type	N present	N missing	Mean	Min	Max

Table S9. Final window feature table: the feature set of Table S8 for the windows retained after the coverage gate and referent matching (Section 3.1), from which every analytic sample is drawn. The full table (2597 rows, 46 columns) is sheet `final_features` of Supplementary File S3; the column dictionary below gives each column's type, completeness and range (source: `table_features_final.csv`).

Column	Type	N present	N missing	Mean	Min	Max
window_id	text/id	2597	0	2597 distinct values		
event_id	text/id	2597	0	62 distinct values		
va_id	numeric	2597	0	13.5	5	33
is_case	numeric	2597	0	0.1286	0	1
length_label	text/id	2597	0	7 distinct values		
length_s	numeric	2597	0	299	120	480
lag_s	numeric	2597	0	27.85	0	120
window_start_utc	numeric	2597	0	1.49e+09	1.476e+09	1.52e+09
window_end_utc	numeric	2597	0	1.49e+09	1.476e+09	1.52e+09
anchor_fall_ts	numeric	2316	281	1.489e+09	1.478e+09	1.518e+09
clock_hour_utc	numeric	2597	0	10.61	0	23
control_day	numeric	2263	334	1.664e+05	161000	180200
control_rank	numeric	2263	334	4.422	0	23
shift_days	numeric	2263	334	2.152	-61	68
has_tof_day	text/id	2263	334	2 distinct values		
day_start	numeric	2597	0	1.664e+05	161000	180200
day_end	numeric	2597	0	1.664e+05	161000	180200
va_id_str	numeric	2597	0	13.5	5	33
imu_n_samples	numeric	2597	0	1.496e+04	5501	30640
imu_coverage_frac	numeric	2597	0	1.001	0.9033	1.427
imu_dt_median_s	numeric	2597	0	0.02	0.02	0.02
imu_dt_min_s	numeric	2597	0	0.008813	0	0.01999
imu_dt_frac_below_half_nominal	numeric	2597	0	0.002788	0	0.3
imu_dt_frac_nonpositive	numeric	2597	0	0.002326	0	0.2991
jerk_max_observed_interval	numeric	2597	0	41.21	0.2837	369.9
resampled_valid_frac	numeric	2597	0	0.9979	0.8972	1
accel_mag_mean	numeric	2597	0	1.004	0.9688	1.072
accel_mag_sd	numeric	2597	0	0.04078	0.0008481	0.3429
accel_mag_iqr	numeric	2597	0	0.01457	0.001134	0.4136
accel_mag_fluctuation_rms	numeric	2597	0	0.04078	0.0008481	0.3429
accel_dynamic_rms_gravity_removed	numeric	2597	0	0.07066	0.001691	0.4808
jerk_mean	numeric	2597	0	0.5718	0.03415	6.865
jerk_sd	numeric	2597	0	1.37	0.04282	9.863
jerk_max	numeric	2597	0	32.25	0.1621	295.4
dom_freq_hz	numeric	2597	0	3.129	0.375	10
spectral_entropy	numeric	2597	0	0.9384	0.6217	0.999
gait_band_power_frac	numeric	2597	0	0.3477	0.01857	0.888
posture_transition_rate	numeric	2597	0	0.8783	0	22
tof_n_samples	numeric	2597	0	936.1	0	2176
tof_coverage_frac	numeric	2597	0	0.7828	0	1.515
tof_speed_mean	numeric	2038	559	0.1726	0.00787	1.35
tof_speed_sd	numeric	2038	559	0.1528	0	1.048
tof_path_length	numeric	2038	559	40.79	0	261.9
tof_net_displacement	numeric	2038	559	2.147	0	16.7
tof_tortuosity	numeric	1426	1171	36.28	1.059	417.7
tof_room_transition_rate	numeric	2543	54	0.6314	0	19.12

Table S10. Per-specification selection accounting: for each window specification, the candidate cases, the cases accepted, the cases dropped by the coverage gate with their event identifiers, the matched referents retained, and the resulting referents per case (Section 3.1). (source: `table_window_selection_by_label.csv`).

length__label	n__cases__candidate	n__cases__accepted	n__cases__dropped__low__coverage	dropped__case__event__ids	n__matched__controls	n__negpool__windows	controls__per__case	n__participants	n__participants__with__case
primary__5min	49	48	1	['F027']	285	94	5.94	25	12
alt__2min	49	48	1	['F032']	288	94	6	25	12
alt__8min	49	47	2	['F015', 'F027']	276	93	5.87	25	12
lag10__5min	49	48	1	['F027']	285	0	5.94	12	12
lag30__5min	49	48	1	['F027']	284	0	5.92	12	12
lag60__5min	49	48	1	['F027']	286	0	5.96	12	12
lag120__5min	49	47	2	['F015', 'F027']	278	0	5.91	12	12

Table S10 note. The coverage gate is applied identically to case, referent and negative-pool windows, so that no arm of the contrast is filtered on a quantity that tracks wear and movement.

Table S11. Coverage-versus-feature association: Spearman correlation between window coverage and each analyzed feature among accepted primary windows (Section 2.5). (source: `table_coverage_feature_association.csv`).

feature	n	spearman_rho	p_value
accel_dynamic_rms_gravity_removed	427	0.0149	0.759
accel_mag_fluctuation_rms	427	0.0151	0.756
jerk_max	427	0.037	0.446
dom_freq_hz	427	0.0243	0.617
spectral_entropy	427	0.0207	0.67
gait_band_power_frac	427	0.0404	0.405
posture_transition_rate	427	0.0566	0.243

Table S12. Referents accepted per case event: the number of matched referent windows retained for each of the 48 analyzed fall events (Section 3.1). (source: `table_controls_per_event.csv`).

event_id	n_controls_accepted
F001	6
F002	6
F003	6
F004	6
F005	6
F006	6
F007	6
F008	6
F009	6
F010	6
F011	6
F012	6
F013	6
F014	6
F015	6
F016	5
F017	6
F018	6
F019	6
F020	6
F021	6
F022	6
F023	6
F024	6
F025	6
F026	6
F028	6
F029	6
F030	6
F031	6
F032	4
F033	6
F034	6
F035	6
F036	6
F037	6
F038	6
F039	6
F040	6
F041	6
F042	6
F043	6
F044	6
F045	6
F046	6
F047	6
F048	6
F049	6

Table S13. Excluded-predictor audit: candidate predictors dropped from the primary conditional model, with the reason for each (Section 2.5). (source: `table_excluded_predictors.csv`).

feature	reason
accel_mag_mean	Near-constant by physics: the window-mean magnitude of a worn accelerometer is dominated by gravity. It carries no within-stratum movement information once a dynamic-acceleration term is in the model, while its rare outliers destabilise the fit.
jerk_mean	Near-duplicate of the dynamic-acceleration term. Its measured correlation and VIF are in the collinearity screen; the pair is not separately identifiable at this event count.

Table S14. Post-hoc collinearity screen: every pairwise Pearson correlation among the candidate amplitude and jerk features, in long form (Section 2.5). (source: `table_collinearity_screen.csv`).

feature_a	feature_b	pearson_r
accel_mag_fluctuation_rms	accel_mag_sd	1
accel_mag_fluctuation_rms	jerk_sd	0.9856
accel_mag_sd	jerk_sd	0.9856
accel_dynamic_rms_gravity_removed	accel_mag_ fluctuation_rms	0.9839
accel_dynamic_rms_gravity_removed	accel_mag_sd	0.9839
accel_dynamic_rms_gravity_removed	jerk_sd	0.9737
accel_mag_sd	jerk_mean	0.9503
accel_mag_fluctuation_rms	jerk_mean	0.9503
jerk_mean	jerk_sd	0.934
accel_dynamic_rms_gravity_removed	jerk_mean	0.9177
accel_mag_iqr	jerk_mean	0.8379
jerk_sd	jerk_max	0.7733
accel_dynamic_rms_gravity_removed	jerk_max	0.7233
accel_mag_fluctuation_rms	jerk_max	0.7063
accel_mag_sd	jerk_max	0.7063
accel_mag_fluctuation_rms	accel_mag_iqr	0.6986
accel_mag_sd	accel_mag_iqr	0.6986
accel_mag_iqr	jerk_sd	0.6512
accel_dynamic_rms_gravity_removed	accel_mag_iqr	0.6497
jerk_mean	jerk_max	0.5642
accel_mag_mean	jerk_mean	0.4954
accel_dynamic_rms_gravity_removed	accel_mag_mean	0.4925
accel_mag_fluctuation_rms	accel_mag_mean	0.489
accel_mag_sd	accel_mag_mean	0.489
accel_mag_mean	jerk_sd	0.4849
accel_mag_iqr	accel_mag_mean	0.4012
accel_mag_mean	jerk_max	0.3415
accel_mag_iqr	jerk_max	0.2882

Table S14 note. The screen was run after an implausible odds ratio was observed in a model containing both dynamic-acceleration terms, and is therefore post hoc.

Table S15. Primary conditional logistic regression, full precision: coefficient, standard error, z-statistic, p-value, odds ratio and 95 % confidence interval (Section 3.2). (source: `table_clr_primary.csv`).

specification	feature	coef	se	z	p_value	or	or_ci_lo	or_ci_hi
primary_5min_imu	accel_dynamic_rms_gravity_removed	1.116	0.312	3.578	0.0003459	3.054	1.657	5.628
primary_5min_imu	jerk_max	0.2551	0.1958	1.303	0.1926	1.291	0.8793	1.894
primary_5min_imu	dom_freq_hz	0.07015	0.2876	0.2439	0.8073	1.073	0.6105	1.885
primary_5min_imu	spectral_entropy	0.536	0.2678	2.001	0.04534	1.709	1.011	2.889
primary_5min_imu	gait_band_power_frac	0.6247	0.3421	1.826	0.06786	1.868	0.9552	3.652
primary_5min_imu	posture_transition_rate	0.1303	0.1666	0.7818	0.4343	1.139	0.8217	1.579

Table S16. Variance-inflation factors for the primary model. (source: `table_clr_vif_all_specifications.csv`).

feature	vif
accel_dynamic_rms_gravity_removed	3.322
jerk_max	2.194
dom_freq_hz	1.617
spectral_entropy	1.766
gait_band_power_frac	2.035
posture_transition_rate	1.543

Table S16 note. Rows for specification `primary_5min_imu`.

specification	feature	coef	se	z	p_value	or	or_ci_lo	or_ci_hi

Table S17. Lag analysis: the primary conditional model refitted on case windows ending 10, 30, 60 and 120 s before the annotated fall time, with the same lag applied to the referent arm (Section 2.7, Section 3.3). (source: `table_clr_lag_analysis.csv`).

specification	feature	coef	se	z	p_value	or	or_ci_lo	or_ci_hi
lag10_5min_imu	accel_dynamic_rms_gravity_removed	1.192	0.3068	3.884	0.0001027	3.293	1.805	6.009
lag10_5min_imu	jerk_max	-0.1305	0.2187	-0.5968	0.5507	0.8777	0.5718	1.347
lag10_5min_imu	dom_freq_hz	0.1299	0.253	0.5135	0.6076	1.139	0.6935	1.87
lag10_5min_imu	spectral_entropy	0.5312	0.2505	2.12	0.03399	1.701	1.041	2.78
lag10_5min_imu	gait_band_power_frac	0.5309	0.3115	1.704	0.08835	1.701	0.9234	3.132
lag10_5min_imu	posture_transition_rate	0.09403	0.1616	0.5818	0.5607	1.099	0.8003	1.508
lag30_5min_imu	accel_dynamic_rms_gravity_removed	1.322	0.3247	4.071	4.689e-05	3.75	1.984	7.087
lag30_5min_imu	jerk_max	-0.354	0.2449	-1.445	0.1484	0.7019	0.4343	1.134
lag30_5min_imu	dom_freq_hz	0.1539	0.2326	0.6616	0.5082	1.166	0.7394	1.84
lag30_5min_imu	spectral_entropy	0.8453	0.2739	3.086	0.002029	2.329	1.361	3.984
lag30_5min_imu	gait_band_power_frac	0.6923	0.3032	2.283	0.02242	1.998	1.103	3.62
lag30_5min_imu	posture_transition_rate	0.04784	0.1555	0.3076	0.7584	1.049	0.7734	1.423
lag60_5min_imu	accel_dynamic_rms_gravity_removed	0.8792	0.3033	2.899	0.003742	2.409	1.33	4.365
lag60_5min_imu	jerk_max	-0.08565	0.2426	-0.353	0.7241	0.9179	0.5706	1.477
lag60_5min_imu	dom_freq_hz	-0.07392	0.2457	-0.3008	0.7636	0.9287	0.5738	1.503
lag60_5min_imu	spectral_entropy	0.6778	0.2661	2.547	0.01088	1.969	1.169	3.318
lag60_5min_imu	gait_band_power_frac	0.4622	0.2995	1.543	0.1228	1.588	0.8827	2.855
lag60_5min_imu	posture_transition_rate	0.09703	0.1624	0.5974	0.5502	1.102	0.8015	1.515
lag120_5min_imu	accel_dynamic_rms_gravity_removed	0.2738	0.2796	0.9795	0.3273	1.315	0.7602	2.275
lag120_5min_imu	jerk_max	0.2113	0.2121	0.9963	0.3191	1.235	0.8152	1.872
lag120_5min_imu	dom_freq_hz	-0.2282	0.2378	-0.9597	0.3372	0.796	0.4995	1.268
lag120_5min_imu	spectral_entropy	0.4015	0.2617	1.534	0.125	1.494	0.8945	2.495
lag120_5min_imu	gait_band_power_frac	0.1328	0.2819	0.4709	0.6377	1.142	0.6572	1.984
lag120_5min_imu	posture_transition_rate	0.2072	0.1529	1.355	0.1755	1.23	0.9116	1.66

Table S17 note. The lag is applied to both arms, so every lagged design is within-participant and time-stratified. The lagged designs are nested in the primary one, sharing 96.7 % to 60.0 % of its span, so differences among their point estimates are not interpreted.

Table S18. Conditional-model estimates for every specification fitted: the primary model, the four lag designs, both alternative window lengths, the acceleration-magnitude feature specification and the IMU plus ToF complete-case model (Section 3.6). (source: `table_clr_all_specifications.csv`).

specification	feature	coef	se	z	p_value	or	or_ci_lo	or_ci_hi
primary_5min_imu	accel_dynamic_rms_gravity_removed	1.116	0.312	3.578	0.0003459	3.054	1.657	5.628
primary_5min_imu	jerk_max	0.2551	0.1958	1.303	0.1926	1.291	0.8793	1.894
primary_5min_imu	dom_freq_hz	0.07015	0.2876	0.2439	0.8073	1.073	0.6105	1.885
primary_5min_imu	spectral_entropy	0.536	0.2678	2.001	0.04534	1.709	1.011	2.889
primary_5min_imu	gait_band_power_frac	0.6247	0.3421	1.826	0.06786	1.868	0.9552	3.652
primary_5min_imu	posture_transition_rate	0.1303	0.1666	0.7818	0.4343	1.139	0.8217	1.579
lag10_5min_imu	accel_dynamic_rms_gravity_removed	1.192	0.3068	3.884	0.0001027	3.293	1.805	6.009
lag10_5min_imu	jerk_max	-0.1305	0.2187	-0.5968	0.5507	0.8777	0.5718	1.347
lag10_5min_imu	dom_freq_hz	0.1299	0.253	0.5135	0.6076	1.139	0.6935	1.87
lag10_5min_imu	spectral_entropy	0.5312	0.2505	2.12	0.03399	1.701	1.041	2.78
lag10_5min_imu	gait_band_power_frac	0.5309	0.3115	1.704	0.08835	1.701	0.9234	3.132
lag10_5min_imu	posture_transition_rate	0.09403	0.1616	0.5818	0.5607	1.099	0.8003	1.508
lag30_5min_imu	accel_dynamic_rms_gravity_removed	1.322	0.3247	4.071	4.689e-05	3.75	1.984	7.087
lag30_5min_imu	jerk_max	-0.354	0.2449	-1.445	0.1484	0.7019	0.4343	1.134
lag30_5min_imu	dom_freq_hz	0.1539	0.2326	0.6616	0.5082	1.166	0.7394	1.84
lag30_5min_imu	spectral_entropy	0.8453	0.2739	3.086	0.002029	2.329	1.361	3.984
lag30_5min_imu	gait_band_power_frac	0.6923	0.3032	2.283	0.02242	1.998	1.103	3.62
lag30_5min_imu	posture_transition_rate	0.04784	0.1555	0.3076	0.7584	1.049	0.7734	1.423
lag60_5min_imu	accel_dynamic_rms_gravity_removed	0.8792	0.3033	2.899	0.003742	2.409	1.33	4.365
lag60_5min_imu	jerk_max	-0.08565	0.2426	-0.353	0.7241	0.9179	0.5706	1.477
lag60_5min_imu	dom_freq_hz	-0.07392	0.2457	-0.3008	0.7636	0.9287	0.5738	1.503
lag60_5min_imu	spectral_entropy	0.6778	0.2661	2.547	0.01088	1.969	1.169	3.318
lag60_5min_imu	gait_band_power_frac	0.4622	0.2995	1.543	0.1228	1.588	0.8827	2.855
lag60_5min_imu	posture_transition_rate	0.09703	0.1624	0.5974	0.5502	1.102	0.8015	1.515
lag120_5min_imu	accel_dynamic_rms_gravity_removed	0.2738	0.2796	0.9795	0.3273	1.315	0.7602	2.275
lag120_5min_imu	jerk_max	0.2113	0.2121	0.9963	0.3191	1.235	0.8152	1.872
lag120_5min_imu	dom_freq_hz	-0.2282	0.2378	-0.9597	0.3372	0.796	0.4995	1.268
lag120_5min_imu	spectral_entropy	0.4015	0.2617	1.534	0.125	1.494	0.8945	2.495
lag120_5min_imu	gait_band_power_frac	0.1328	0.2819	0.4709	0.6377	1.142	0.6572	1.984
lag120_5min_imu	posture_transition_rate	0.2072	0.1529	1.355	0.1755	1.23	0.9116	1.66
alt_2min_imu	accel_dynamic_rms_gravity_removed	1.359	0.3448	3.942	8.092e-05	3.893	1.98	7.651
alt_2min_imu	jerk_max	0.3355	0.2196	1.528	0.1266	1.399	0.9094	2.151
alt_2min_imu	dom_freq_hz	-0.03683	0.3321	-0.1109	0.9117	0.9638	0.5027	1.848
alt_2min_imu	spectral_entropy	0.6025	0.2869	2.1	0.03574	1.827	1.041	3.205
alt_2min_imu	gait_band_power_frac	0.8302	0.3865	2.148	0.03171	2.294	1.075	4.892
alt_2min_imu	posture_transition_rate	0.2616	0.2202	1.188	0.2349	1.299	0.8436	2
alt_8min_imu	accel_dynamic_rms_gravity_removed	0.5723	0.2887	1.983	0.04741	1.772	1.007	3.121
alt_8min_imu	jerk_max	0.4968	0.2043	2.431	0.01504	1.644	1.101	2.453
alt_8min_imu	dom_freq_hz	0.05121	0.2696	0.19	0.8493	1.053	0.6205	1.785
alt_8min_imu	spectral_entropy	0.4282	0.2827	1.514	0.13	1.534	0.8816	2.671
alt_8min_imu	gait_band_power_frac	0.5815	0.3354	1.734	0.08294	1.789	0.927	3.452
alt_8min_imu	posture_transition_rate	0.1366	0.1851	0.7381	0.4604	1.146	0.7976	1.648
primary_5min_imu_magnitude_sd	accel_mag_fluctuation_rms	0.8696	0.2918	2.981	0.002876	2.386	1.347	4.227
primary_5min_imu_magnitude_sd	jerk_max	0.3251	0.1991	1.633	0.1024	1.384	0.9371	2.045
primary_5min_imu_magnitude_sd	dom_freq_hz	0.03499	0.2832	0.1235	0.9017	1.036	0.5944	1.804
primary_5min_imu_magnitude_sd	spectral_entropy	0.5589	0.2804	1.993	0.04625	1.749	1.009	3.03
primary_5min_imu_magnitude_sd	gait_band_power_frac	0.6793	0.3374	2.014	0.04405	1.973	1.018	3.821
primary_5min_imu_magnitude_sd	posture_transition_rate	0.2719	0.1571	1.73	0.08356	1.312	0.9646	1.786
primary_5min_imu_tof_complete_case	accel_dynamic_rms_gravity_removed	2.738	0.7359	3.721	0.0001987	15.46	3.654	65.38
primary_5min_imu_tof_complete_case	jerk_max	-0.08039	0.3141	-0.256	0.798	0.9228	0.4986	1.708
primary_5min_imu_tof_complete_case	dom_freq_hz	-0.2185	0.413	-0.529	0.5968	0.8037	0.3577	1.806
primary_5min_imu_tof_complete_case	spectral_entropy	-0.2661	0.3008	-0.8847	0.3763	0.7663	0.425	1.382
primary_5min_imu_tof_complete_case	gait_band_power_frac	-0.01274	0.4243	-0.03003	0.976	0.9873	0.4298	2.268
primary_5min_imu_tof_complete_case	posture_transition_rate	-0.004617	0.2589	-0.01783	0.9858	0.9954	0.5993	1.653

specification	feature	coef	se	z	p_value	or	or_ci_lo	or_ci_hi
primary_5min_imu_tof_complete_case	tof_speed_mean	-0.9614	0.5421	-1.774	0.07612	0.3823	0.1321	1.106
primary_5min_imu_tof_complete_case	tof_path_length	-0.7508	0.538	-1.396	0.1628	0.472	0.1645	1.355
primary_5min_imu_tof_complete_case	tof_tortuosity	-0.4983	0.4367	-1.141	0.2539	0.6076	0.2582	1.43
primary_5min_imu_tof_complete_case	tof_room_transition_rate	0.1617	0.3211	0.5036	0.6146	1.176	0.6265	2.206

Table S19. Variance-inflation factors for every specification in Table S18. (source: `table_clr_vif_all_specifications.csv`).

specification	feature	vif
primary_5min_imu	accel_dynamic_rms_gravity_removed	3.322
primary_5min_imu	jerk_max	2.194
primary_5min_imu	dom_freq_hz	1.617
primary_5min_imu	spectral_entropy	1.766
primary_5min_imu	gait_band_power_frac	2.035
primary_5min_imu	posture_transition_rate	1.543
lag10_5min_imu	accel_dynamic_rms_gravity_removed	3.422
lag10_5min_imu	jerk_max	2.421
lag10_5min_imu	dom_freq_hz	1.676
lag10_5min_imu	spectral_entropy	1.769
lag10_5min_imu	gait_band_power_frac	2.142
lag10_5min_imu	posture_transition_rate	1.56
lag30_5min_imu	accel_dynamic_rms_gravity_removed	3.508
lag30_5min_imu	jerk_max	2.49
lag30_5min_imu	dom_freq_hz	1.593
lag30_5min_imu	spectral_entropy	1.639
lag30_5min_imu	gait_band_power_frac	1.917
lag30_5min_imu	posture_transition_rate	1.474
lag60_5min_imu	accel_dynamic_rms_gravity_removed	3.416
lag60_5min_imu	jerk_max	2.594
lag60_5min_imu	dom_freq_hz	1.599
lag60_5min_imu	spectral_entropy	1.635
lag60_5min_imu	gait_band_power_frac	1.934
lag60_5min_imu	posture_transition_rate	1.534
lag120_5min_imu	accel_dynamic_rms_gravity_removed	3.138
lag120_5min_imu	jerk_max	2.403
lag120_5min_imu	dom_freq_hz	1.567
lag120_5min_imu	spectral_entropy	1.722
lag120_5min_imu	gait_band_power_frac	2.011
lag120_5min_imu	posture_transition_rate	1.337
alt_2min_imu	accel_dynamic_rms_gravity_removed	3.291
alt_2min_imu	jerk_max	2.26
alt_2min_imu	dom_freq_hz	1.839
alt_2min_imu	spectral_entropy	1.506
alt_2min_imu	gait_band_power_frac	2.086
alt_2min_imu	posture_transition_rate	1.687
alt_8min_imu	accel_dynamic_rms_gravity_removed	2.884
alt_8min_imu	jerk_max	2.157
alt_8min_imu	dom_freq_hz	1.582
alt_8min_imu	spectral_entropy	1.736
alt_8min_imu	gait_band_power_frac	2.086
alt_8min_imu	posture_transition_rate	1.495
primary_5min_imu_magnitude_sd	accel_mag_fluctuation_rms	3.076
primary_5min_imu_magnitude_sd	jerk_max	2.234
primary_5min_imu_magnitude_sd	dom_freq_hz	1.621
primary_5min_imu_magnitude_sd	spectral_entropy	1.915
primary_5min_imu_magnitude_sd	gait_band_power_frac	2.063
primary_5min_imu_magnitude_sd	posture_transition_rate	1.355
primary_5min_imu_tof_complete_case	accel_dynamic_rms_gravity_removed	5.811
primary_5min_imu_tof_complete_case	jerk_max	2.278
primary_5min_imu_tof_complete_case	dom_freq_hz	1.892
primary_5min_imu_tof_complete_case	spectral_entropy	1.633
primary_5min_imu_tof_complete_case	gait_band_power_frac	2.449
primary_5min_imu_tof_complete_case	posture_transition_rate	1.813
primary_5min_imu_tof_complete_case	tof_speed_mean	1.698
primary_5min_imu_tof_complete_case	tof_path_length	2.877
primary_5min_imu_tof_complete_case	tof_tortuosity	1.187
primary_5min_imu_tof_complete_case	tof_room_transition_rate	1.694

Table S20. Alternative window lengths: the primary conditional model refitted on 2-minute and 8-minute windows in place of the primary 5-minute window (Section 2.3). (source: `table_clr_sensitivity_lengths.csv`).

specification	feature	coef	se	z	p_value	or	or_ci_lo	or_ci_hi
alt_2min_imu	accel_dynamic_rms_gravity_removed	1.359	0.3448	3.942	8.092e-05	3.893	1.98	7.651
alt_2min_imu	jerk_max	0.3355	0.2196	1.528	0.1266	1.399	0.9094	2.151
alt_2min_imu	dom_freq_hz	-0.03683	0.3321	-0.1109	0.9117	0.9638	0.5027	1.848
alt_2min_imu	spectral_entropy	0.6025	0.2869	2.1	0.03574	1.827	1.041	3.205
alt_2min_imu	gait_band_power_frac	0.8302	0.3865	2.148	0.03171	2.294	1.075	4.892
alt_2min_imu	posture_transition_rate	0.2616	0.2202	1.188	0.2349	1.299	0.8436	2
alt_8min_imu	accel_dynamic_rms_gravity_removed	0.5723	0.2887	1.983	0.04741	1.772	1.007	3.121
alt_8min_imu	jerk_max	0.4968	0.2043	2.431	0.01504	1.644	1.101	2.453
alt_8min_imu	dom_freq_hz	0.05121	0.2696	0.19	0.8493	1.053	0.6205	1.785
alt_8min_imu	spectral_entropy	0.4282	0.2827	1.514	0.13	1.534	0.8816	2.671
alt_8min_imu	gait_band_power_frac	0.5815	0.3354	1.734	0.08294	1.789	0.927	3.452
alt_8min_imu	posture_transition_rate	0.1366	0.1851	0.7381	0.4604	1.146	0.7976	1.648

Table S21. IMU plus ToF complete-case model: conditional logistic regression including the time-of-flight features alongside the IMU features, restricted to windows with complete data on both sensors. (source: `table_clr_imu_tof_complete_case.csv`).

specification	feature	coef	se	z	p_value	or	or_ci_lo	or_ci_hi
primary_5min_imu_tof_complete_case	accel_dynamic_rms_gravity_removed	2.738	0.7359	3.721	0.0001987	15.46	3.654	65.38
primary_5min_imu_tof_complete_case	jerk_max	-0.08039	0.3141	-0.256	0.798	0.9228	0.4986	1.708
primary_5min_imu_tof_complete_case	dom_freq_hz	-0.2185	0.413	-0.529	0.5968	0.8037	0.3577	1.806
primary_5min_imu_tof_complete_case	spectral_entropy	-0.2661	0.3008	-0.8847	0.3763	0.7663	0.425	1.382
primary_5min_imu_tof_complete_case	gait_band_power_frac	-0.01274	0.4243	-0.03003	0.976	0.9873	0.4298	2.268
primary_5min_imu_tof_complete_case	posture_transition_rate	-0.004617	0.2589	-0.01783	0.9858	0.9954	0.5993	1.653
primary_5min_imu_tof_complete_case	tof_speed_mean	-0.9614	0.5421	-1.774	0.07612	0.3823	0.1321	1.106
primary_5min_imu_tof_complete_case	tof_path_length	-0.7508	0.538	-1.396	0.1628	0.472	0.1645	1.355
primary_5min_imu_tof_complete_case	tof_tortuosity	-0.4983	0.4367	-1.141	0.2539	0.6076	0.2582	1.43
primary_5min_imu_tof_complete_case	tof_room_transition_rate	0.1617	0.3211	0.5036	0.6146	1.176	0.6265	2.206

Table S22. Variance-inflation factors for the IMU plus ToF complete-case model. (source: `table_clr_vif_all_specifications.csv`).

feature	vif
accel_dynamic_rms_gravity_removed	5.811
jerk_max	2.278
dom_freq_hz	1.892
spectral_entropy	1.633
gait_band_power_frac	2.449
posture_transition_rate	1.813
tof_speed_mean	1.698
tof_path_length	2.877
tof_tortuosity	1.187
tof_room_transition_rate	1.694

Table S22 note. Rows for specification `primary_5min_imu_tof_complete_case`.

Table S23. Standardization-population comparison: the primary model's odds ratios with features z-scored over every row of the primary window table, against z-scoring over the rows that enter the conditional likelihood (Section 3.6). (source: `table_zscoring_population_effect.csv`).

feature	or_standardised_all_rows	or_standardised_analysis_set	ratio
accel_dynamic_rms_gravity_removed	2.998	3.054	0.982
jerk_max	1.269	1.291	0.9833
dom_freq_hz	1.072	1.073	0.999
spectral_entropy	1.78	1.709	1.042
gait_band_power_frac	1.846	1.868	0.9885
posture_transition_rate	1.13	1.139	0.9919

Table S23 note. The analysis standardises after strata with no case are removed, so the rows that set the per-SD scale are the rows that contribute to the likelihood.

Table S24. Leave-one-participant-out sensitivity: the primary model refitted after removing, in turn, each case-contributing participant (13 fits including the full model), with the falls dropped and the informative strata remaining at each step (Section 3.5). (source: `table_lopo_sensitivity.csv`).

specification	feature	coef	se	z	p_value	or	or_ci_lo	or_ci_hi	dropped_participant	n_falls_dropped	n_informative_strata_remaining
full_model	accel_dynamic_rms_gravity_removed	1.116	0.312	3.578	0.0003459	3.054	1.657	5.628	none (full model)	0	12
full_model	jerk_max	0.2551	0.1958	1.303	0.1926	1.291	0.8793	1.894	none (full model)	0	12
full_model	dom_freq_hz	0.07015	0.2876	0.2439	0.8073	1.073	0.6105	1.885	none (full model)	0	12
full_model	spectral_entropy	0.536	0.2678	2.001	0.04534	1.709	1.011	2.889	none (full model)	0	12
full_model	gait_band_power_frac	0.6247	0.3421	1.826	0.06786	1.868	0.9552	3.652	none (full model)	0	12
full_model	posture_transition_rate	0.1303	0.1666	0.7818	0.4343	1.139	0.8217	1.579	none (full model)	0	12
drop_va_6	accel_dynamic_rms_gravity_removed	1.406	0.4196	3.351	0.0008048	4.081	1.793	9.289	va_6	12	11
drop_va_6	jerk_max	0.1487	0.2297	0.6473	0.5174	1.16	0.7397	1.82	va_6	12	11
drop_va_6	dom_freq_hz	-0.2846	0.3948	-0.7209	0.471	0.7523	0.347	1.631	va_6	12	11
drop_va_6	spectral_entropy	0.6175	0.3799	1.625	0.1041	1.854	0.8807	3.904	va_6	12	11
drop_va_6	gait_band_power_frac	0.6354	0.4647	1.367	0.1715	1.888	0.7593	4.693	va_6	12	11
drop_va_6	posture_transition_rate	0.02306	0.1983	0.1163	0.9074	1.023	0.6938	1.509	va_6	12	11
drop_va_9	accel_dynamic_rms_gravity_removed	1.088	0.3144	3.461	0.0005381	2.968	1.603	5.496	va_9	2	11
drop_va_9	jerk_max	0.2567	0.1981	1.296	0.195	1.293	0.8767	1.906	va_9	2	11
drop_va_9	dom_freq_hz	0.07796	0.2829	0.2755	0.7829	1.081	0.6209	1.882	va_9	2	11
drop_va_9	spectral_entropy	0.5406	0.2679	2.018	0.04361	1.717	1.016	2.903	va_9	2	11
drop_va_9	gait_band_power_frac	0.5544	0.3386	1.638	0.1015	1.741	0.8966	3.38	va_9	2	11
drop_va_9	posture_transition_rate	0.139	0.1625	0.8552	0.3924	1.149	0.8357	1.58	va_9	2	11
drop_va_11	accel_dynamic_rms_gravity_removed	1.17	0.3718	3.147	0.001651	3.222	1.555	6.676	va_11	13	11
drop_va_11	jerk_max	0.2993	0.2444	1.225	0.2206	1.349	0.8356	2.178	va_11	13	11
drop_va_11	dom_freq_hz	0.2481	0.3288	0.7546	0.4505	1.282	0.6728	2.441	va_11	13	11
drop_va_11	spectral_entropy	0.8131	0.354	2.297	0.02162	2.255	1.127	4.513	va_11	13	11
drop_va_11	gait_band_power_frac	0.8078	0.4295	1.881	0.06004	2.243	0.9664	5.205	va_11	13	11
drop_va_11	posture_transition_rate	0.1299	0.1997	0.6505	0.5154	1.139	0.7699	1.684	va_11	13	11
drop_va_13	accel_dynamic_rms_gravity_removed	1.157	0.3152	3.671	0.0002412	3.182	1.715	5.901	va_13	2	11
drop_va_13	jerk_max	0.2768	0.2045	1.354	0.1759	1.319	0.8834	1.969	va_13	2	11
drop_va_13	dom_freq_hz	0.1034	0.2953	0.3502	0.7262	1.109	0.6216	1.978	va_13	2	11
drop_va_13	spectral_entropy	0.293	0.2487	1.178	0.2388	1.34	0.8232	2.183	va_13	2	11
drop_va_13	gait_band_power_frac	0.4877	0.3295	1.48	0.1388	1.629	0.8538	3.106	va_13	2	11
drop_va_13	posture_transition_rate	0.06748	0.171	0.3946	0.6932	1.07	0.7651	1.496	va_13	2	11
drop_va_14	accel_dynamic_rms_gravity_removed	0.9108	0.3524	2.585	0.009742	2.486	1.246	4.96	va_14	9	11
drop_va_14	jerk_max	0.2886	0.2183	1.322	0.1862	1.335	0.87	2.047	va_14	9	11
drop_va_14	dom_freq_hz	0.1103	0.3067	0.3596	0.7191	1.117	0.6121	2.037	va_14	9	11
drop_va_14	spectral_entropy	0.5236	0.2977	1.759	0.07859	1.688	0.9419	3.025	va_14	9	11
drop_va_14	gait_band_power_frac	0.777	0.3712	2.094	0.0363	2.175	1.051	4.502	va_14	9	11
drop_va_14	posture_transition_rate	0.1987	0.1928	1.031	0.3026	1.22	0.836	1.78	va_14	9	11
drop_va_16	accel_dynamic_rms_gravity_removed	1.213	0.3214	3.773	0.0001615	3.362	1.791	6.313	va_16	1	11
drop_va_16	jerk_max	0.229	0.198	1.157	0.2474	1.257	0.853	1.854	va_16	1	11
drop_va_16	dom_freq_hz	0.03113	0.2932	0.1062	0.9154	1.032	0.5807	1.833	va_16	1	11
drop_va_16	spectral_entropy	0.5907	0.272	2.172	0.02989	1.805	1.059	3.077	va_16	1	11
drop_va_16	gait_band_power_frac	0.5342	0.3453	1.547	0.1219	1.706	0.8671	3.357	va_16	1	11
drop_va_16	posture_transition_rate	0.127	0.1669	0.7611	0.4466	1.135	0.8187	1.575	va_16	1	11
drop_va_20	accel_dynamic_rms_gravity_removed	1.142	0.3183	3.586	0.0003353	3.131	1.678	5.843	va_20	1	11
drop_va_20	jerk_max	0.2427	0.1985	1.223	0.2215	1.275	0.8639	1.881	va_20	1	11
drop_va_20	dom_freq_hz	0.1811	0.287	0.6309	0.5281	1.198	0.6829	2.103	va_20	1	11
drop_va_20	spectral_entropy	0.5124	0.2664	1.924	0.05441	1.669	0.9904	2.814	va_20	1	11
drop_va_20	gait_band_power_frac	0.6436	0.3465	1.857	0.06325	1.903	0.9651	3.754	va_20	1	11
drop_va_20	posture_transition_rate	0.2337	0.1775	1.317	0.1879	1.263	0.8921	1.789	va_20	1	11
drop_va_21	accel_dynamic_rms_gravity_removed	1.207	0.3232	3.735	0.0001878	3.344	1.775	6.301	va_21	2	11
drop_va_21	jerk_max	0.1818	0.2025	0.8979	0.3692	1.199	0.8065	1.784	va_21	2	11
drop_va_21	dom_freq_hz	0.07262	0.2929	0.2479	0.8042	1.075	0.6056	1.909	va_21	2	11
drop_va_21	spectral_entropy	0.5931	0.2778	2.135	0.03275	1.81	1.05	3.119	va_21	2	11
drop_va_21	gait_band_power_frac	0.6691	0.3501	1.911	0.05596	1.953	0.9831	3.878	va_21	2	11
drop_va_21	posture_transition_rate	0.1246	0.17	0.733	0.4636	1.133	0.8117	1.581	va_21	2	11
drop_va_24	accel_dynamic_rms_gravity_removed	1.107	0.3069	3.607	0.0003101	3.025	1.658	5.521	va_24	1	11
drop_va_24	jerk_max	0.2659	0.1923	1.383	0.1668	1.305	0.8949	1.902	va_24	1	11
drop_va_24	dom_freq_hz	0.06218	0.2879	0.216	0.829	1.064	0.6053	1.871	va_24	1	11
drop_va_24	spectral_entropy	0.5431	0.2679	2.027	0.04269	1.721	1.018	2.91	va_24	1	11
drop_va_24	gait_band_power_frac	0.612	0.3448	1.775	0.07593	1.844	0.9381	3.625	va_24	1	11
drop_va_24	posture_transition_rate	0.07835	0.1682	0.4659	0.6413	1.081	0.7778	1.504	va_24	1	11
drop_va_26	accel_dynamic_rms_gravity_removed	0.9511	0.3025	3.145	0.001663	2.589	1.431	4.683	va_26	2	11
drop_va_26	jerk_max	0.308	0.2009	1.533	0.1252	1.361	0.9178	2.017	va_26	2	11
drop_va_26	dom_freq_hz	0.05201	0.3112	0.1671	0.8673	1.053	0.5724	1.939	va_26	2	11
drop_va_26	spectral_entropy	0.4762	0.2688	1.771	0.07649	1.61	0.9506	2.727	va_26	2	11
drop_va_26	gait_band_power_frac	0.6271	0.3514	1.784	0.07435	1.872	0.9402	3.728	va_26	2	11

specification	feature	coef	se	z	p_value	or	or_ci_lo	or_ci_hi	dropped_participant	n_falls_dropped	n_informative_strata_remaining
drop_va_26	posture_transition_rate	0.188	0.1732	1.085	0.2777	1.207	0.8595	1.695	va_26	2	11
drop_va_30	accel_dynamic_rms_gravity_removed	1.096	0.3149	3.482	0.0004985	2.993	1.615	5.548	va_30	1	11
drop_va_30	jerk_max	0.2588	0.1965	1.317	0.1879	1.295	0.8813	1.904	va_30	1	11
drop_va_30	dom_freq_hz	0.07639	0.2826	0.2703	0.7869	1.079	0.6203	1.878	va_30	1	11
drop_va_30	spectral_entropy	0.53	0.2686	1.973	0.04846	1.699	1.004	2.876	va_30	1	11
drop_va_30	gait_band_power_frac	0.6263	0.3429	1.827	0.06777	1.871	0.9553	3.663	va_30	1	11
drop_va_30	posture_transition_rate	0.1345	0.1683	0.7992	0.4241	1.144	0.8226	1.591	va_30	1	11
drop_va_33	accel_dynamic_rms_gravity_removed	1.134	0.3174	3.572	0.0003538	3.108	1.668	5.79	va_33	2	11
drop_va_33	jerk_max	0.2485	0.1989	1.249	0.2117	1.282	0.8681	1.893	va_33	2	11
drop_va_33	dom_freq_hz	0.04211	0.2943	0.1431	0.8862	1.043	0.5858	1.857	va_33	2	11
drop_va_33	spectral_entropy	0.5505	0.273	2.016	0.04375	1.734	1.016	2.961	va_33	2	11
drop_va_33	gait_band_power_frac	0.6227	0.3482	1.788	0.0737	1.864	0.942	3.688	va_33	2	11
drop_va_33	posture_transition_rate	0.1089	0.1687	0.6453	0.5187	1.115	0.8011	1.552	va_33	2	11

Table S25. Leave-one-participant-out stability summary: per-feature maximum shift in log odds ratio across all single-participant removals, and how many removals change the feature's significance. (source: `table_lopo_stability_summary.csv`).

feature	full_or	full_p	max_log_or_shift_focus3	significance_flips_when_dropping	max_log_or_shift_any_participant	significance_flips_any_participant	n_participants_whose_removal_flips
dom_freq_hz	1.073	0.8073	0.3547		0.3547		0
accel_dynamic_rms_gravity_removed	3.054	0.0003459	0.29		0.29		0
spectral_entropy	1.709	0.04534	0.2771	va_6;va_14	0.2771	va_6(12);va_13(2);va_14(9);va_20(1);va_26(2)	5
gait_band_power_frac	1.868	0.06786	0.183	va_14	0.183	va_14(9)	1
posture_transition_rate	1.139	0.4343	0.1072		0.1072		0
jerk_max	1.291	0.1926	0.1064		0.1064		0

Table S26. Permutation-control null distribution: the model likelihood-ratio statistic under within-stratum permutations of the case labels. (source: `table_permutation_null_lr.csv`).

lr_perm
4.607
9.336
5.05
17.96
5.334
9.627
3.042
8.997
3.175
3.783
23.14
5.357
2.634
7.906
11.8
4.325
5.612
3.117
2.254
4.086
9.908
6.448
1.898
7.776
2.756
7.288
6.211
4.003
8.774
4.13
5.528
0.8488
6.205
4.047
2.779
2.303
8.308
10.33
3.892
11.21
7.167
1.354
1.445
4.105
2.227
5.82
6.187
5.484
5.427
8.205
9.69
4.808
5.393
3.421
10.44
2.208
5.84
6.143
8.008
3.286
6.471
6.208
3.868
5.741
13.71
1.185

lr_perm
3.909
5.815
7.667
5.166
3.674
3.344
2.237
6.585
8.262
4.259
2.569
5.735
2
1.093
6.101
11.64
7.434
10.57
2.135
24.25
9.163
3.271
8.841
7.453
9.025
3.882
6.505
8.237
4.45
2.707
1.368
4.497
3.859
10.04
6.575
3.475
3.931
3.613
7.843
6.293
6.101
6.707
3.79
4.104
2.668
5.216
2.2
8.424
4.451
6.105
2.635
10.29
4.701
6.819
3.927
2.79
8.582
6.109
9.094
2.405
2.901
9.319
7.947
6.889
4.226
1.715
3.805
5.452

lr_perm
11.38
7.568
9.733
12.07
4.507
8.74
13.22
4.338
3.979
8.217
1.313
7.502
6.888
1.869
6.254
6.489
2.066
1.362
8.012
4.015
4.233
17.24
10.87
2.335
13.14
5.547
3.709
3.209
2.216
1.223
2.164
3.368
9.283
4.602
6.684
6.694
3.288
9.272
4.469
4.427
4.669
9.088
6.22
2.915
5.618
4.275
11.07
2.918
12.07
4.897
6.292
7.677
2.325
8.812
2.318
4.606
4.139
0.7242
1.686
2.857
5.15
9.126
8.514
5.42
11.55
10.92
6.163
3.334

lr_perm
2.893
5.447
13.77
6.646
7.787
18.41
9.811
15.18
0.9433
4.57
8.436
4.255
3.762
9.983
3.788
6.097
3.893
7.642
2.925
14.41
6.376
4.152
2.145
10.69
5.705
3.277
10.25
6.987
13.52
3.451
3.548
6.78
3.352
7.93
7.201
9.715
4.081
7.202
2.746
3.118
3.357
6.172
6.786
4.187
6.959
7.374
3.287
7.589
6.833
6.32
8.647
2.866
5.437
4.668
6.808
2.553
5.126
4.299
2.924
6.45
6.997
8.124
7.208
5.271
5.812
1.483
5.108
3.086

lr_perm
8.854
8.39
7.576
2.236
8.68
5.602
8.142
6.783
4.906
14.14
11.7
6.639
7.64
10.58
1.906
4.7
2.133
4.365
7.949
15.13
7.773
6.401
7.279
5.978
5.14
17.05
4.925
8.695
5.323
2.3
12.35
5.073
11.61
4.937
4.479
5.466
6.484
12.19
7.621
5.005
2.085
2.742
7.916
6.425
7.13
5.15
7.516
6.378
8.414
1.991
1.706
3.396
6.266
19.37
7.128
9.1
9.244
3.143
7.005
10.47
2.037
1.359
7.696
4.37
7.642
7.563
5.518
6.853

lr_perm
5.359
4.04
17.53
5.579
9.707
6.42
10.1
6.486
3.13
1.74
8.43
3.576
5.118
4.676
9.19
7.14
2.514
9.771
3.092
9.866
5.526
1.063
3.398
3.533
6.138
4.88
5.399
4.282
8.331
2.188
2.072
2.865
7.456
7.38
4.824
10.33
3.159
5.254
0.4372
6.987
4.707
7.554
6.127
5.912
1.419
4.743
11.63
9.201
5.251
1.703
7.941
9.215
5.772
9.128
10.32
8.072
11.08
4.355
7.631
4.767
19.7
5.049
7.821
5.871
7.415
7.154
6.135
7.263

lr_perm
17.72
2.608
4.315
1.696
1.643
4.688
2.87
14.38
10.76
4.675
3.444
1.363
9.263
16.54
3.8
3.707
4.592
5.22
1.253
12.42
3.872
4.799
0.793
11.43
4.126
5.139
4.437
1.234
7.362
5.493
11
8.345
13.97
11.67
4.801
2.488
1.706
5.253
5.089
6.065
8.286
1.658
4.483
4.06
7.618
2.592
6.94
2.215
2.684
6.684
5.233
8.661
4.91
1.711
7.19
6.295
4.373
7.759
14.36
3.477
13.26
3.148
16.38
7.863
6.048
6.219
2.892
1.257

lr_perm
12.63
7.568
5.813
11.25
5.067
8.411
3.598
3.028
9.47
6.557
2.734
2.122
8.46
8.677
7.753
3.048
14.56
3.119
3.504
3.608
6.743
3.013
11.24
4.712
10.36
4.567

Table S27. Leave-one-feature-out check: the model likelihood-ratio statistic after dropping each feature from the primary model in turn, against the full-model statistic. (source: `table_lofo_carries_check.csv`).

feature_dropped	n_features_remaining	lr_statistic	lr_full_model	lr_drop_frac_of_full	within_perm_null_range
accel_dynamic_rms_gravity_removed	5	53.75	67.69	0.7941	False
spectral_entropy	5	63.13	67.69	0.9325	False
gait_band_power_frac	5	64.1	67.69	0.9469	False
jerk_max	5	66.01	67.69	0.9751	False
posture_transition_rate	5	67.08	67.69	0.9909	False
dom_freq_hz	5	67.64	67.69	0.9991	False

Table S28. Leakage audit: source sensor and formula for each predictor, with confirmation that every feature is computed from raw sensor samples within its own window and never from the label or its timing (Section 2.6). (source: `table_leakage_audit.csv`).

feature	source_sensor	formula	derived_from_label	justification
accel_mag_mean	IMU	mean(		a(t)
accel_mag_fluctuation_rms	IMU	RMS of mean-removed		a(t)
accel_dynamic_rms_gravity_removed	IMU	RMS of		a(t) - g_hat(t)
jerk_mean	IMU	mean(	d	
jerk_max	IMU	max(	d	
dom_freq_hz	IMU	peak-power frequency of the Welch PSD, 0.3-10 Hz band	No	Computed purely from the window's raw sensor samples; the window's is_case label is assigned from its timing relative to a fall timestamp when the window index is built, before any feature is computed, and is never used as an input to a feature formula.
spectral_entropy	IMU	normalized Shannon entropy of the PSD, 0.3-10 Hz band	No	Computed purely from the window's raw sensor samples; the window's is_case label is assigned from its timing relative to a fall timestamp when the window index is built, before any feature is computed, and is never used as an input to a feature formula.
gait_band_power_frac	IMU	fraction of PSD power in the 0.5-3 Hz gait band	No	Computed purely from the window's raw sensor samples; the window's is_case label is assigned from its timing relative to a fall timestamp when the window index is built, before any feature is computed, and is never used as an input to a feature formula.
posture_transition_rate	IMU	count/min of >30-degree orientation-vector deviations	No	Computed purely from the window's raw sensor samples; the window's is_case label is assigned from its timing relative to a fall timestamp when the window index is built, before any feature is computed, and is never used as an input to a feature formula.
tof_speed_mean	ToF	mean(		v(t)
tof_path_length	ToF	sum of consecutive-sample ToF position displacements	No	Computed purely from the window's raw sensor samples; the window's is_case label is assigned from its timing relative to a fall timestamp when the window index is built, before any feature is computed, and is never used as an input to a feature formula.
tof_tortuosity	ToF	ToF path length / net displacement	No	Computed purely from the window's raw sensor samples; the window's is_case label is assigned from its timing relative to a fall timestamp when the window index is built, before any feature is computed, and is never used as an input to a feature formula.
tof_room_transition_rate	ToF	count/min of per-participant k-means cluster-label changes	No	Computed purely from the window's raw sensor samples; the window's is_case label is assigned from its timing relative to a fall timestamp when the window index is built, before any feature is computed, and is never used as an input to a feature formula.

Table S29. Descriptive statistics by case and referent status: mean and SD of each feature in case windows and in referent windows, before model fitting. (source: `table_descriptive_by_case.csv`).

feature	n_case	case_mean	case_sd	n_control	control_mean	control_sd
accel_dynamic_rms_gravity_removed	48	0.1496	0.06685	379	0.05923	0.0712
jerk_max	48	82.03	55.35	379	26.14	39.66
dom_freq_hz	48	2.406	1.308	379	3.132	1.95
spectral_entropy	48	0.9288	0.04683	379	0.9402	0.05327
gait_band_power_frac	48	0.4159	0.13	379	0.3437	0.1376
posture_transition_rate	48	2.133	2.449	379	0.6607	1.374
tof_speed_mean	39	0.2312	0.1035	293	0.1673	0.1782
tof_path_length	39	61.25	30.84	293	38.73	34.27
tof_tortuosity	39	19.24	17.84	193	43.88	59.07
tof_room_transition_rate	48	1.208	1.752	362	0.5873	1.589

Table S30. Gradient-boosted model cross-validation folds: test-set size, case count, participant count and discrimination for each fold of the participant-grouped cross-validation (5 folds; Section 3.7). (source: `table_gbm_cv_folds.csv`).

n_splits	fold	n_test	n_test_cases	n_test_participants	auroc	auprc	evaluable
5	0	14	2	1	0.6667	0.2667	True
5	1	8	0	1			False
5	2	39	3	5	0.6481	0.1313	True
5	3	155	18	8	0.8025	0.2782	True
5	4	211	25	10	0.8247	0.3319	True

Table S30 note. A fold containing no case has no defined AUROC and contributes nothing. With few case-contributing participants a participant-grouped split cannot guarantee a case in every fold.

Table S31. Gradient-boosted model TreeSHAP importance: mean absolute SHAP attribution for every IMU and ToF feature. (source: `table_gbm_shap_importance.csv`).

feature	mean_abs_shap
jerk_max	1.292
accel_dynamic_rms_ gravity_removed	1.05
tof_tortuosity	0.7084
posture_transition_rate	0.6429
gait_band_power_frac	0.4631
tof_speed_mean	0.3232
tof_room_transition_rate	0.2017
tof_path_length	0.1917
accel_mag_mean	0.1795
dom_freq_hz	0.1485
spectral_entropy	0.1298
jerk_mean	0.06988

Table S32. Gradient-boosted versus conditional-model feature ranking: mean absolute SHAP attribution, conditional-model z-statistic and odds ratio, and the rank each model assigns to each feature (Section 3.7). (source: `table_gbm_vs_clr_ranking.csv`).

feature	mean_abs_shap	z	or	shap_rank	clr_rank
jerk_max	1.292	1.303	1.291	1	4
accel_dynamic_rms_gravity_removed	1.05	3.578	3.054	2	1
posture_transition_rate	0.6429	0.7818	1.139	3	5
gait_band_power_frac	0.4631	1.826	1.868	4	3
dom_freq_hz	0.1485	0.2439	1.073	5	6
spectral_entropy	0.1298	2.001	1.709	6	2

Table S32 note. The two models draw on the same recordings, features and labels but not the same windows: the boosted model is fitted on all 427 primary windows from 25 participants, and the conditional model on the 333 in informative strata. The comparison describes what a structurally different learner attends to; it is not independent confirmation.

Table S33. Participant characteristics as the public release permits them to be described: monitored days, calendar span, fall records, analyzed falls, matched referents, unanchored windows, usable-coverage fraction, and ambulatory fraction for each of the 26 archived participants (Section 2.2, Table 2). (source: `table_participant_characteristics.csv`).

participant	analysed	imu_days	calendar_span_days	falls	n_analysed_falls	n_matched_referents	n_unanchored_windows	contributes_to_conditional_model	frac_windows_with_usable_imu	frac_usable_windows_ambulatory	has_tof_days
va_4	False	25	32	0	0	0	0	False	0.292	0.429	True
va_5	True	60	63	0	0	0	7	False	0.292	0.857	True
va_6	True	49	62	16	12	72	0	True	0.636	0.497	True
va_7	True	44	61	0	0	0	8	False	0.5	0.417	False
va_8	True	40	46	0	0	0	8	False	0.583	0.429	True
va_9	True	56	57	2	2	12	0	True	0.868	0.333	True
va_10	True	66	66	0	0	0	8	False	0.333	0.5	False
va_11	True	63	64	15	13	77	0	True	0.85	0.624	True
va_12	True	60	60	0	0	0	8	False	0.583	0.429	True
va_13	True	57	67	2	2	12	0	True	0.842	0.688	True
va_14	True	55	65	9	9	52	0	True	0.737	0.27	True
va_15	True	49	65	0	0	0	6	False	0.25	0.333	True
va_16	True	22	29	1	1	6	0	True	0.684	0.385	True
va_17	True	24	34	0	0	0	6	False	0.25	0.667	True
va_18	True	53	62	0	0	0	7	False	0.292	0.429	True
va_19	True	32	63	0	0	0	8	False	0.375	0.111	True
va_20	True	47	65	1	1	6	0	True	0.632	0.583	True
va_21	True	37	55	2	2	12	0	True	0.5	0.632	True
va_22	True	41	65	0	0	0	8	False	0.5	0.25	True
va_23	True	56	57	0	0	0	8	False	0.667	0.188	True
va_24	True	41	75	1	1	6	0	True	0.526	0.3	True
va_25	True	57	66	0	0	0	8	False	0.5	0.25	True
va_26	True	63	78	2	2	12	0	True	0.684	0.885	True
va_28	True	46	64	0	0	0	4	False	0.167	0	True
va_30	True	45	68	1	1	6	0	True	0.737	0.286	True
va_33	True	74	74	2	2	12	0	True	0.711	0.593	True

Table S33 note. The release carries inertial and position signal and a fall-timestamp file only. It contains no age, sex, disease duration, phenotype, disability score, medication or assistive-device information, so none appears here.

Table S34. Referent-day activity balance: for each movement feature, the mean within-participant standardized value of referents on days before their anchoring fall and of referents on days after it, with a Mann-Whitney test of the difference (Section 2.3, Section 3.4). (source: `table_referent_day_balance.csv`).

feature	n_pre_fall_referents	n_post_fall_referents	mean_within_participant_z_pre	mean_within_participant_z_post	mann_whitney_p
accel_dynamic_rms_gravity_removed	141	144	0.1782	-0.1745	0.002617
gait_band_power_frac	141	144	0.02415	-0.02365	0.4604
jerk_max	141	144	0.1614	-0.1581	0.004256
posture_transition_rate	141	144	0.1016	-0.09951	0.2904

Table S34 note. Standardizing within participant stops a participant who contributes many referents from creating the difference through their own activity level. Features differing at $p < 0.05$: `accel_dynamic_rms_gravity_removed`, `jerk_max`.

Table S35. Referent days per participant, split by whether the referent day precedes or follows its anchoring fall, with the case count for each participant (Section 3.4). (source: `table_referent_days_per_participant.csv`).

va_id	n_referents_post_fall	n_referents_pre_fall	n_cases
6	17	55	12
9	1	11	2
11	33	44	13
13	12	0	2
14	43	9	9
16	5	1	1
20	6	0	1
21	5	7	2
24	6	0	1
26	4	8	2
30	5	1	1
33	7	5	2

Table S35 note. In total, 3 participants contribute no pre-fall referent, so the pre-fall-only refit in Table S36 loses strata as well as events.

Table S36. Pre-fall referent sensitivity analysis: the primary conditional model refitted on referents from days preceding the fall only, and from days following it only, against the model on referents as drawn (Section 3.4, Table 5, Figure 3A). (source: `table_clr_prefall_referents.csv`).

specification	feature	coef	se	z	p_value	or	or_ci_lo	or_ci_hi	n_rows	n_cases	n_controls	n_informative_strata
primary (all referent days)	accel_dynamic_rms_gravity_removed	1.116	0.312	3.578	0.0003459	3.054	1.657	5.628	333	48	285	12
primary (all referent days)	jerk_max	0.2551	0.1958	1.303	0.1926	1.291	0.8793	1.894	333	48	285	12
primary (all referent days)	dom_freq_hz	0.07015	0.2876	0.2439	0.8073	1.073	0.6105	1.885	333	48	285	12
primary (all referent days)	spectral_entropy	0.536	0.2678	2.001	0.04534	1.709	1.011	2.889	333	48	285	12
primary (all referent days)	gait_band_power_frac	0.6247	0.3421	1.826	0.06786	1.868	0.9552	3.652	333	48	285	12
primary (all referent days)	posture_transition_rate	0.1303	0.1666	0.7818	0.4343	1.139	0.8217	1.579	333	48	285	12
pre-fall referent days only	accel_dynamic_rms_gravity_removed	1.242	0.3707	3.35	0.000809	3.462	1.674	7.16	185	44	141	9
pre-fall referent days only	jerk_max	0.1547	0.24	0.6447	0.5191	1.167	0.7293	1.869	185	44	141	9
pre-fall referent days only	dom_freq_hz	0.2822	0.3048	0.9258	0.3546	1.326	0.7296	2.41	185	44	141	9
pre-fall referent days only	spectral_entropy	0.436	0.2762	1.579	0.1144	1.546	0.9001	2.657	185	44	141	9
pre-fall referent days only	gait_band_power_frac	0.6622	0.3755	1.764	0.07778	1.939	0.929	4.047	185	44	141	9
pre-fall referent days only	posture_transition_rate	0.09074	0.2247	0.4037	0.6864	1.095	0.7049	1.701	185	44	141	9
post-fall referent days only	accel_dynamic_rms_gravity_removed	1.079	0.4313	2.501	0.01239	2.94	1.263	6.848	192	48	144	12
post-fall referent days only	jerk_max	0.6602	0.3098	2.131	0.03311	1.935	1.054	3.552	192	48	144	12
post-fall referent days only	dom_freq_hz	-0.04185	0.3487	-0.12	0.9045	0.959	0.4842	1.9	192	48	144	12
post-fall referent days only	spectral_entropy	0.3541	0.3939	0.899	0.3687	1.425	0.6584	3.084	192	48	144	12
post-fall referent days only	gait_band_power_frac	0.5084	0.4562	1.114	0.2651	1.663	0.68	4.066	192	48	144	12
post-fall referent days only	posture_transition_rate	0.2491	0.2516	0.99	0.3222	1.283	0.7835	2.1	192	48	144	12

Table S36 note. The washout clears the 24 h after a fall but not day +2 onward, so referents can follow a fall. Table S34 compares the activity of such referents with that of pre-fall referents.

Table S37. Activity-matched contrasts: the movement term estimated with an ambulation marker as covariate, with both arms restricted to ambulatory windows, and against referents drawn from the participant's own walking periods, each as a full model and, where applicable, with the movement term fitted alone (Section 2.8, Section 3.4, Table 5, Figure 3B). (source: `table_clr_activity_contrast.csv`).

specification	feature	coef	se	z	p_value	or	or_ci_lo	or_ci_hi	n_rows	n_cases	n_controls	n_informative_strata
primary	accel_dynamic_rms_gravity_removed	1.116	0.312	3.578	0.0003459	3.054	1.657	5.628	333	48	285	12
primary	jerk_max	0.2551	0.1958	1.303	0.1926	1.291	0.8793	1.894	333	48	285	12
primary	dom_freq_hz	0.07015	0.2876	0.2439	0.8073	1.073	0.6105	1.885	333	48	285	12
primary	spectral_entropy	0.536	0.2678	2.001	0.04534	1.709	1.011	2.889	333	48	285	12
primary	gait_band_power_frac	0.6247	0.3421	1.826	0.06786	1.868	0.9552	3.652	333	48	285	12
primary	posture_transition_rate	0.1303	0.1666	0.7818	0.4343	1.139	0.8217	1.579	333	48	285	12
ambulation indicator alone	ambulatory	1.739	0.3694	4.709	2.492e-06	5.694	2.761	11.75	333	48	285	12
movement term alone	accel_dynamic_rms_gravity_removed	1.286	0.199	6.466	1.01e-10	3.62	2.451	5.346	333	48	285	12
movement term + ambulation indicator	accel_dynamic_rms_gravity_removed	0.9659	0.233	4.146	3.383e-05	2.627	1.664	4.147	333	48	285	12
movement term + ambulation indicator	ambulatory	1.254	0.382	3.283	0.001029	3.504	1.657	7.41	333	48	285	12
full model, ambulation indicator for posture rate	accel_dynamic_rms_gravity_removed	0.8849	0.3144	2.815	0.004882	2.423	1.308	4.486	333	48	285	12
full model, ambulation indicator for posture rate	jerk_max	0.2222	0.1966	1.13	0.2584	1.249	0.8495	1.836	333	48	285	12
full model, ambulation indicator for posture rate	dom_freq_hz	0.1175	0.3003	0.3914	0.6955	1.125	0.6243	2.026	333	48	285	12
full model, ambulation indicator for posture rate	spectral_entropy	0.4396	0.2624	1.676	0.09378	1.552	0.9282	2.596	333	48	285	12
full model, ambulation indicator for posture rate	gait_band_power_frac	0.53	0.3275	1.618	0.1056	1.699	0.8942	3.228	333	48	285	12
full model, ambulation indicator for posture rate	ambulatory	1.131	0.3861	2.93	0.003387	3.1	1.454	6.608	333	48	285	12
restricted to ambulatory windows, both arms	accel_dynamic_rms_gravity_removed	0.7459	0.2996	2.489	0.01279	2.108	1.172	3.793	173	46	127	12
restricted to ambulatory windows, both arms	jerk_max	0.2763	0.2274	1.215	0.2243	1.318	0.8442	2.059	173	46	127	12
restricted to ambulatory windows, both arms	dom_freq_hz	0.1888	0.2613	0.7224	0.4701	1.208	0.7237	2.016	173	46	127	12
restricted to ambulatory windows, both arms	spectral_entropy	0.5098	0.288	1.771	0.07663	1.665	0.9469	2.928	173	46	127	12
restricted to ambulatory windows, both arms	gait_band_power_frac	0.6745	0.3478	1.94	0.05243	1.963	0.9929	3.881	173	46	127	12
restricted to ambulatory windows, movement term alone	accel_dynamic_rms_gravity_removed	0.8518	0.2392	3.561	0.0003698	2.344	1.467	3.746	173	46	127	12
walking referents, full model	accel_dynamic_rms_gravity_removed	0.3227	0.2425	1.331	0.1833	1.381	0.8584	2.221	266	48	218	12
walking referents, full model	jerk_max	0.4002	0.1936	2.067	0.03872	1.492	1.021	2.181	266	48	218	12
walking referents, full model	dom_freq_hz	0.007887	0.235	0.03357	0.9732	1.008	0.6359	1.598	266	48	218	12
walking referents, full model	spectral_entropy	0.2995	0.2794	1.072	0.2837	1.349	0.7803	2.333	266	48	218	12
walking referents, full model	gait_band_power_frac	0.1562	0.3178	0.4914	0.6231	1.169	0.627	2.18	266	48	218	12
walking referents, full model	posture_transition_rate	0.1835	0.1645	1.115	0.2647	1.201	0.8703	1.658	266	48	218	12
walking referents, movement term alone	accel_dynamic_rms_gravity_removed	0.5482	0.1723	3.182	0.001464	1.73	1.234	2.425	266	48	218	12

Table S37 note. The walking referents are selected on the gait-band power fraction and the posture-transition rate, which are model predictors. Those predictors then carry little within-stratum contrast, which attenuates the movement term through their collinearity, so the single-predictor fit on the same referent set is reported beside the full model.

Table S38. The lag family refitted within ambulatory windows: the movement term at each lag with both arms unrestricted and with both arms restricted to ambulatory windows (Section 2.8, Section 3.4, Table 5). (source: `table_clr_lag_within_activity.csv`).

specification	feature	coef	se	z	p_value	or	or_ci_lo	or_ci_hi	n_rows	n_cases	n_controls	n_informative_strata	lag_s	arm
lag 0 s, all windows	accel_dynamic_rms_gravity_removed	1.211	0.2899	4.176	2.966e-05	3.356	1.901	5.924	333	48	285	12	0	all windows
lag 0 s, ambulatory windows only	accel_dynamic_rms_gravity_removed	0.7459	0.2996	2.489	0.01279	2.108	1.172	3.793	173	46	127	12	0	ambulatory windows only
lag 10 s, all windows	accel_dynamic_rms_gravity_removed	1.249	0.2917	4.28	1.87e-05	3.485	1.968	6.174	333	48	285	12	10	all windows
lag 10 s, ambulatory windows only	accel_dynamic_rms_gravity_removed	0.8649	0.3117	2.775	0.005528	2.375	1.289	4.375	175	43	132	12	10	ambulatory windows only
lag 30 s, all windows	accel_dynamic_rms_gravity_removed	1.354	0.307	4.412	1.023e-05	3.875	2.123	7.071	332	48	284	12	30	all windows
lag 30 s, ambulatory windows only	accel_dynamic_rms_gravity_removed	0.9188	0.3204	2.868	0.004131	2.506	1.338	4.696	180	41	139	12	30	ambulatory windows only
lag 60 s, all windows	accel_dynamic_rms_gravity_removed	0.9202	0.2938	3.132	0.001735	2.51	1.411	4.464	334	48	286	12	60	all windows
lag 60 s, ambulatory windows only	accel_dynamic_rms_gravity_removed	0.425	0.3185	1.335	0.182	1.53	0.8194	2.855	180	40	140	11	60	ambulatory windows only
lag 120 s, all windows	accel_dynamic_rms_gravity_removed	0.3713	0.2648	1.402	0.1609	1.45	0.8626	2.436	325	47	278	12	120	all windows
lag 120 s, ambulatory windows only	accel_dynamic_rms_gravity_removed	-0.119	0.3144	-0.3784	0.7052	0.8878	0.4794	1.644	162	33	129	11	120	ambulatory windows only

Table S38 note. The posture-transition rate is dropped from the predictor set in both arms because it defines the restriction, so every row is a five-predictor model and the unrestricted rows are not the six-predictor model of Table 3. The five designs are nested in one another, so the ordering of their point estimates is not interpreted.

Table S39. Fall-capture comparison: inertial coverage of case windows against clock-matched referent candidates from the same participants on other days (Section 2.1, Table 5 footnote). (source: `table_fall_capture_selection.csv`).

arm	n_windows	frac_coverage_at_least_0.9	frac_zero_imu_samples	median_coverage_fraction
case windows	49	0.9796	0	1
clock-matched control candidates	882	0.72	0.2313	1

Table S39 note. Among the referent candidates, 23.1% contain no inertial samples, against 0.0% of case windows. The comparison arm holds only candidates anchored to a fall; the unanchored windows of fall-free participants are not clock-matched and are excluded.

Table S40. Referent-timing refits: the movement term with referents on the far side of a daylight-saving change from their case removed, for every window specification, and, for the primary window, with referents restricted to those preceding the participant's first fall or falling at least seven days after any fall (Section 2.3, Section 3.4). (source: `table_clr_referent_timing_sensitivity.csv`).

specification	feature	coef	se	z	p_value	or	or_ci_lo	or_ci_hi	n_rows	n_cases	n_controls	n_informative_strata	window_specification	referent_set	n_referents_dropped
primary_5min	all referents	accel_dynamic_rms_gravity_removed	1.116	0.312	3.578	0.0003459	3.054	1.657	5.628	333	48	285	12	primary_5min	all referents
primary_5min	referents on the case's side of daylight saving	accel_dynamic_rms_gravity_removed	1.083	0.3416	3.171	0.001519	2.955	1.512	5.772	264	48	216	12	primary_5min	referents on the case's side of daylight saving
primary_5min	referents before the participant's first fall	accel_dynamic_rms_gravity_removed	1.528	0.5379	2.841	0.004498	4.61	1.606	13.23	105	31	74	8	primary_5min	referents before the participant's first fall
primary_5min	referents at least 7 days after any fall	accel_dynamic_rms_gravity_removed	1.331	0.3641	3.655	0.0002576	3.783	1.853	7.722	210	48	162	12	primary_5min	referents at least 7 days after any fall
lag10_5min	all referents	accel_dynamic_rms_gravity_removed	1.192	0.3068	3.884	0.0001027	3.293	1.805	6.009	333	48	285	12	lag10_5min	all referents
lag10_5min	referents on the case's side of daylight saving	accel_dynamic_rms_gravity_removed	1.193	0.3392	3.516	0.0004386	3.296	1.695	6.408	264	48	216	12	lag10_5min	referents on the case's side of daylight saving
lag30_5min	all referents	accel_dynamic_rms_gravity_removed	1.322	0.3247	4.071	4.689e-05	3.75	1.984	7.087	332	48	284	12	lag30_5min	all referents
lag30_5min	referents on the case's side of daylight saving	accel_dynamic_rms_gravity_removed	1.341	0.3654	3.669	0.0002433	3.822	1.868	7.824	264	48	216	12	lag30_5min	referents on the case's side of daylight saving
lag60_5min	all referents	accel_dynamic_rms_gravity_removed	0.8792	0.3033	2.899	0.003742	2.409	1.33	4.365	334	48	286	12	lag60_5min	all referents
lag60_5min	referents on the case's side of daylight saving	accel_dynamic_rms_gravity_removed	0.9362	0.3355	2.791	0.00526	2.55	1.321	4.922	264	48	216	12	lag60_5min	referents on the case's side of daylight saving
lag120_5min	all referents	accel_dynamic_rms_gravity_removed	0.2738	0.2796	0.9795	0.3273	1.315	0.7602	2.275	325	47	278	12	lag120_5min	all referents
lag120_5min	referents on the case's side of daylight saving	accel_dynamic_rms_gravity_removed	0.306	0.2934	1.043	0.297	1.358	0.7641	2.413	263	47	216	12	lag120_5min	referents on the case's side of daylight saving
alt_2min	all referents	accel_dynamic_rms_gravity_removed	1.359	0.3448	3.942	8.092e-05	3.893	1.98	7.651	336	48	288	12	alt_2min	all referents
alt_2min	referents on the case's side of daylight saving	accel_dynamic_rms_gravity_removed	1.286	0.3795	3.389	0.0007006	3.619	1.72	7.615	267	48	219	12	alt_2min	referents on the case's side of daylight saving
alt_8min	all referents	accel_dynamic_rms_gravity_removed	0.5723	0.2887	1.983	0.04741	1.772	1.007	3.121	323	47	276	12	alt_8min	all referents
alt_8min	referents on the case's side of daylight saving	accel_dynamic_rms_gravity_removed	0.5401	0.306	1.765	0.07757	1.716	0.9421	3.126	263	47	216	12	alt_8min	referents on the case's side of daylight saving

Table S40 note. Referents are the case window shifted by whole multiples of 86,400 s in the archive's UTC time base, so a referent across a daylight-saving change is one hour from its case in local clock time. Matching in local time would require redrawing the referent windows.

Table S41. Archive identifier coverage: every participant identifier in the range the dataset documentation gives (va_5 to va_33), and whether a recording for it is present in the public archive (Section 2.1). (source: `table_archive_id_coverage.csv`).

va_id	participant	in_archive
5	va_5	True
6	va_6	True
7	va_7	True
8	va_8	True
9	va_9	True
10	va_10	True
11	va_11	True
12	va_12	True
13	va_13	True
14	va_14	True
15	va_15	True
16	va_16	True
17	va_17	True
18	va_18	True
19	va_19	True
20	va_20	True
21	va_21	True
22	va_22	True
23	va_23	True
24	va_24	True
25	va_25	True
26	va_26	True
27	va_27	False
28	va_28	True
29	va_29	False
30	va_30	True
31	va_31	False
32	va_32	False
33	va_33	True

Table S42. Sampling time base: coverage fractions above 1 among primary candidate windows, and peak jerk computed by dividing by the observed sampling interval relative to peak jerk on the uniform 50 Hz grid (Section 2.4, Section 3.6). (source: `table_jerk_time_base_comparison.csv`).

population	quantity	value
primary candidate windows	n_windows	1267
primary candidate windows	n_coverage_above_1	148
primary candidate windows	frac_coverage_above_1	0.1168
primary candidate windows	max_coverage_fraction	1.36
conditional-model analysis set	n_windows	333
conditional-model analysis set	median_ratio	1.214
conditional-model analysis set	frac_ratio_above_2	0.07808
conditional-model analysis set	max_ratio	5.978
conditional-model analysis set	frac_ratio_above_1	0.982
all accepted primary windows	n_windows	427
all accepted primary windows	median_ratio	1.229
all accepted primary windows	frac_ratio_above_2	0.08899
all accepted primary windows	max_ratio	7.595
all accepted primary windows	frac_ratio_above_1	0.9813

Table S43. Primary conditional model refitted with peak jerk computed from the observed sampling interval in place of peak jerk on the uniform grid (Section 3.6). (source: `table_clr_observed_interval_jerk.csv`).

specification	feature	coef	se	z	p_value	or	or_ci_lo	or_ci_hi
primary_5min_imu_observed_interval_jerk	accel_dynamic_rms_gravity_removed	0.9557	0.3068	3.115	0.001842	2.6	1.425	4.745
primary_5min_imu_observed_interval_jerk	jerk_max_observed_interval	0.4438	0.194	2.288	0.02215	1.559	1.066	2.28
primary_5min_imu_observed_interval_jerk	dom_freq_hz	0.06687	0.2923	0.2287	0.8191	1.069	0.6028	1.896
primary_5min_imu_observed_interval_jerk	spectral_entropy	0.5086	0.2708	1.878	0.06032	1.663	0.9782	2.827
primary_5min_imu_observed_interval_jerk	gait_band_power_frac	0.6475	0.3498	1.851	0.06413	1.911	0.9627	3.793
primary_5min_imu_observed_interval_jerk	posture_transition_rate	0.131	0.1698	0.7714	0.4405	1.14	0.8172	1.59

Table S44. Ambulation marker by arm: windows with at least one posture transition, among the case and referent windows entering the conditional model (Section 2.4). (source: `table_ambulatory_marker_by_arm.csv`).

arm	n_windows	n_ambulatory	frac_ambulatory
case windows	48	46	0.9583
referent windows	285	127	0.4456
